# A fine-tuned β-catenin regulation during proliferation of corneal endothelial cells revealed using proteomics analysis

**DOI:** 10.1038/s41598-020-70800-w

**Published:** 2020-08-14

**Authors:** Eleonora Maurizi, Davide Schiroli, Roberta Zini, Anna Limongelli, Raffaela Mistò, Claudio Macaluso, Graziella Pellegrini

**Affiliations:** 1 Centre for Regenerative Medicine “S. Ferrari”, Department of Life Sciences, University of Modena and Reggio Emilia, Modena, Italy; 2 Transfusion Medicine Unit, Azienda USL-IRCCS, Reggio Emilia, Italy; 3 Eye Bank of Monza, San Gerardo Hospital, Monza, Italy; 4 Department of Medicine and Surgery, Dentistry Center, University of Parma, Parma, Italy

**Keywords:** Biochemistry, Cell biology, Molecular biology

## Abstract

Corneal endothelial (CE) dysfunction is the main indication for corneal transplantation, an invasive procedure with several limitations. Developing novel strategies to re-activate CE regenerative capacity is, therefore, of fundamental importance. This goal has proved to be challenging as corneal endothelial cells (CEnC) are blocked in the G0/G1 phase of the cell cycle in vivo and, albeit retaining proliferative capacity in vitro, this is further hindered by endothelial-to-mesenchymal transition. Herein we investigated the mechanisms regulating CEnC proliferation in vitro*.* Comparing the proteome of non-proliferating (in vivo—G0/G1) and proliferating (in vitro—G2/M) rabbit CEnC (rCEnC), 77 proteins, out of 3,328 identified, were differentially expressed in the two groups (*p* < 0.005). Literature and Gene Ontology analysis revealed β-catenin and transforming growth factor (TGF-β) pathways to be correlated with the identified proteins. Treatment of rCEnC with a β-catenin activator and inhibitor showed that β-catenin activation was necessary during rCEnC proliferation, but not sufficient for its induction. Furthermore, both pro-proliferative activity of basic fibroblast growth factor and anti-proliferative effects of TGF-β were regulated through β-catenin. Overall, these results provide novel insights into the molecular basis underlying the proliferation process that CEnC re-activate in vitro*,* consolidating the role of β-catenin and TGF-β*.*

## Introduction

The corneal endothelium (CE) is a monolayer of cells localised in the innermost segment of the cornea, regulating solutes transport from and to the aqueous humor (pump-leak hypothesis)^1^. Corneal endothelial cells (CEnC) are generally considered non-dividing in vivo^2^ and arrested in the G0/G1 phase of the cell cycle^3,4^. The presence of anti-proliferative factors in the aqueous humor, the stress-induced premature senescence, and the contact inhibition between cells are the main events triggering the expression of cell cycle regulators that induce CEnC mitotic block^2^. For this reason, as CEnC density decreases by 0.6% each year^5,6^, cell loss is compensated through migration and cell enlargement^2,7^. When, as a consequence of pathologies or surgical treatments, endothelial cell density falls below 500 cells/mm^2^, cell enlargement is no longer able to compensate for the passive leaking^7^. In this case, the impaired CE results in corneal swelling and, if untreated, in the subsequent severe loss of corneal transparency, ultimately leading to blindness.

CEnC dysfunction is the underlying cause of about 40% of all corneal transplants performed worldwide^7^. Corneal grafts, despite decisive advancements in the last decades, are still complex and invasive procedures, with some severe limitations, including immune graft rejection, graft failure, and a general scarcity of donor corneas^7^.

Alternative clinical procedures have been recently proposed in order to increase the number of patients that can be treated and to improve their quality of life. Currently, descemetorhexis is the only therapeutic approach that avoids allogeneic transplantation. This treatment involves the removal of the central CEnC to directly stimulate migration of the patient’s own peripheral CEnC towards the central cornea. Unfortunately, although promising, the procedure can be applied only in those cases that have preserved a high density of CEnC in the peripheral cornea (i.e., in some cases of Fuchs endothelial dystrophy), and the rate of success is still low, requiring a long time for the post-operative recovery^7^.

Interestingly, successful studies have recently promoted the development of a cell-based therapy^8,9^, which may be considered as a less invasive substitute for a corneal graft. Since induced mitosis has been observed in cultured CEnC^10^, heterologous cell transplantation to the patient’s anterior chamber turned out to be a promising strategy. However, despite CEnC expansion in vitro has been thoroughly investigated^11,12^, only in 2018 Kinoshita’s group demonstrated for the first time that injected CEnC from young donors are able to repopulate the cornea of a cohort of patients^8^. Two factors, in particular, have made CEnC culture in vitro very challenging: their reduced proliferative capacity, exacerbated over passages by replicative senescence^12^, and the propensity to spontaneously lose their morphology through an endothelial-to-mesenchymal transition (EnMT)^13^.

For implementation and efficient substitution of corneal graft in clinical practice, these or other new approaches need to be investigated. First, it is necessary to acquire deeper knowledge on how to regulate and circumvent the mitotic block. One prominent approach aiming to reach this goal was focused on the examination of the role of several growth factors. Among them, basic fibroblast growth factor (bFGF)^14–16^, insulin-like growth factor (IGF)^16^ and hepatocyte growth factor (HGF)^16^, all present in the aqueous humor, stimulate CEnC proliferation, while transforming growth factor beta (TGF-β), which is also found in the aqueous humor, induces CEnC mitotic block^17,18^. Intracellular pathways, activated by these growth factors, are shown not to act in parallel but rather to build a network of cross-regulation: bFGF promotes cellular proliferation in CEnC through PI3K/Akt^4,19–21^, while its activity is counteracted by the presence of TGF-β^19,22^. Nevertheless, in CEnC, the complexity of pathway regulation is not limited to the cross-talk between them but also implies the alternative functions, which the same pathway can overtake in different cellular conditions. For instance, TGF-β activation has been linked either to CEnC maturation or to EnMT^23^, while bFGF, although promoting proliferation^19^, was also found to be involved in EnMT^24,25^. Intriguingly, canonical Wnt signalling and consequent β-catenin nuclear internalization, triggered by bFGF together with EDTA-mediated cell junction disruption, promotes both an increase in CEnC proliferation and in EnMT^26^. In accordance with the aforementioned study, β-catenin in CEnC was described to promote cellular proliferation through the expression of cyclin D1^27^, but also to regulate EnMT^28^. Taken together, these elements suggest that Wnt/β-catenin pathway may promote proliferation and, in parallel, regulate a sensitive balance between the maintenance of the polygonal morphology and the transformation to the fibroblastic phenotype (EnMT). Any variation in mechanotransduction, medium components or donor’s cell characteristics can impair this delicate equilibrium and induce development of one fate rather than the other.

Nevertheless, the role of this and other factors orchestrating CEnC proliferation while maintaining the correct morphology has to be further elucidated.

Similar to recent studies on novel eye surface cellular mechanisms^29,30^, we used here a hypothesis-free approach to compare the proteome of rabbit CEnC (rCEnC) isolated from the corneal tissue with the proteome of rCEnC grown in culture. This analysis allowed the identification of 3,328 rabbit proteins, 77 of which were differentially regulated at the two conditions with a *p* < 0.005. Several of these proteins were found to be associated with common intracellular pathways, in particular Wnt/β-catenin and TGF-β, which were further investigated in cultured rCEnC. Nuclear translocation of β-catenin was observed in mitotic cells while TGF-β was shown to arrest cells in the G2/M phase of the cell cycle as well as block β-catenin nuclear translocation. Moreover, β-catenin pathway inhibition significantly decreased cellular proliferation. By contrast, we did not observe any pro-proliferative effect upon direct Wnt pathway activation. In summary, our results suggested that β-catenin is essential, but not sufficient to overcome the mitotic block. In parallel, we confirmed that bFGF has a pro-proliferative effect on CEnC and that its activity is mediated by β-catenin activation and counteracted by TGF-β stimulation. Overall, the results provide new insights into the role of β-catenin in corneal endothelial proliferation in vitro, after cell–cell disruption and growth factors stimulation (bFGF and TGF-β).

### Results

#### Identification of cell cycle phases characterising rabbit CEnC in vitro and ex vivo

**Figure 1:**
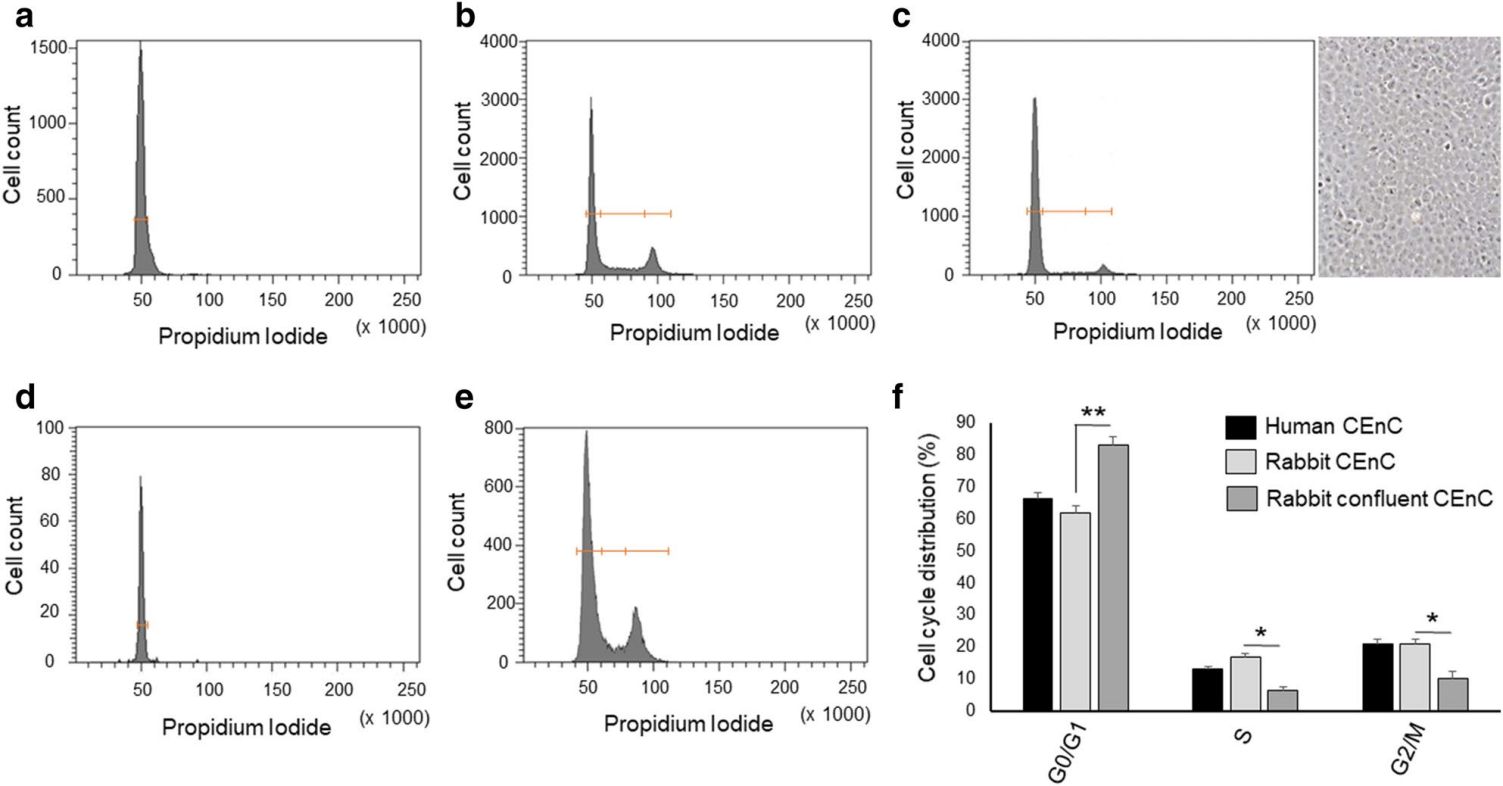
Comparison of the cell cycle distribution of corneal endothelial cells in vitro and ex vivo, in rabbit and human: cells distribution in the three different phases of the cell cycle identified by Propidium Iodide flow cytofluorimetric analysis and plotted in a representative graph for each condition. In (**a**), the graph shows rabbit CEnC from the ex vivo tissue, the peak represents the G0/G1 phase, while in (**b**), the graph identifies three different peaks for the cultured rCEnC, representing the G0/G1, S, and G2/M phases, from left to right. In (**c**), we can observe how confluent rCEnC decrease their proliferating component (G2/M). On the right, a representative image of a confluent rCEnC culture, obtained with the Axiovert 40C inverted microscope (Zeiss), objective 5x. Similarly, in (**d**), the graph shows a unique peak for the ex vivo human CE cells (G0/G1), while the graph (**e**) exhibits the distribution of the human CEnC in all three different phases of the cell cycle. Panel (**f**) represents a bar chart of Propidium Iodide flow cytofluorimetric analysis comparing human and rabbit CEnC at 60% confluence with confluent rabbit CEnC. Experiments were performed n = 3. Results are presented as mean ± Standard Error (SE). T-test was performed *p < 0.05, **p < 0.01.

rCEnC growing in vitro were compared with ex vivo rCEnC directly isolated from the Descemet’s membrane, using flow cytometry and PI staining, in order to assess the cell cycle status of rCEnC in the experimental samples. Rabbit CE ex vivo exhibited a unique defined peak, representing cells in the G0/G1 phase of the cell cycle (Fig. 1a). rCEnC expanded in vitro were distributed instead among different phases of the cell cycle, depending on cell culture confluence: at 60% confluence, 20% of the population was in G2/M phases of the cell cycle (Fig. 1b), while confluent cells exhibited prevalence of the cells in G0/G1, similarly to those isolated from the tissue (Fig. 1c). Human CEnC showed a similar behaviour: all of them were found to be in the G0/G1 phase when isolated from the tissue and directly analysed (Fig. 1d), while they were redistributed in the three different phases with percentages similar to the rCEnC, when harvested at 60% confluence (Fig. 1e). Quantifying cell cycle distribution of both human and rabbit CEnC at 60% confluence we confirmed that human and rabbit CEnC, under the same culture condition, have a comparable rate of proliferating cells and indicated that the rabbit CE is an appropriate model for studying cell cycle regulation (Fig. 1f). Interestingly, human CEnC at higher passages (P4), presenting with an elongated morphology^13^, showed a significantly increased population of cells in G2/M phase compared to the previously analysed human CEnC (P2) (Supplementary Figure S1).

#### Proteomic analysis

Proteomic comparison of rabbit CE and cultured rCEnC was performed in order to elucidate differentially regulated pathways between tissue and actively proliferating cultured cells. The proteomic analysis, carried out in a total of 30 samples, identified 3,328 proteins from the complete rabbit 20,190,508 database. The expression levels of these proteins were highly reproducible within the biological replicates under examination. This allowed the selection of proteins with a significant difference between the tissue and the cultured rCEnC: 77 proteins with a *p* value < 0.005, 19 of which with a *p* value < 0.001 (heat map in Supplementary Figure S2). Among those with a *p* value < 0.005, 16 proteins were found to be upregulated, while 61 proteins were downregulated in in vitro rCEnC as compared to the ex vivo rCEnC from the tissue (Tables 1, 2). A full protein dataset is available in supplementary material (Supplementary Table S1).

**Table 1:** List of proteins from proteomic analysis which were over-expressed in rCEnC isolated and cultured in vitro if compared with the rCEnC isolated from the tissue, with a *p* value < 0.005 and < 0.001.

General function	Protein name	Protein symbol	Uniprot	*p* value
**Up-regulated in rCEnC**				
Trafficking	AP-2 complex subunit beta	AP2B1	G1SL02	< 0.005
Actin polymerization	Actin-related protein 2/3 complex subunit 3	ARPC3	G1T277	< 0.001
Chaperone	DnaJ homolog subfamily A member 1	DNAJA1	G1SML9	< 0.005
Chaperone	DnaJ homolog subfamily A member 2	DNAJA2	G1SMM5	< 0.005
Nucleolar protein	Nucleolar protein 16	NOP16	G1T4W0	< 0.005
Phospholipase	Cytosolic phospholipase A2	PLA2G4A	G1T7T8	< 0.005
RNA processing	DNA-directed RNA polymerases I and III subunit RPAC1	POLR1C	G1SP24	< 0.005
Proteasome	Proteasome subunit beta type-7	PSMB7	G1SWK8	< 0.005
Proteasome	26S proteasome non-ATPase regulatory subunit 2	PSMD2	G1SSA2	< 0.005
Proteasome	26S proteasome non-ATPase regulatory subunit 3	PSMD3	G1TP15	< 0.005
Proteasome	26S proteasome non-ATPase regulatory subunit 6	PSMD6	G1U115	< 0.005
Proteasome	26S proteasome non-ATPase regulatory subunit 7	PSMD7	G1SVT4	< 0.005
Proteasome	26S proteasome non-ATPase regulatory subunit 12	PSMD12	G1T6D4	< 0.005
Replication factor	Replication factor C subunit 2	RFC2	G1TNA6	< 0.001
Trafficking	Protein transport protein Sec24C	SEC24C	G1TZQ5	< 0.005
RNA processing	Heterogeneous nuclear ribonucleoprotein Q	SYNCRIP	G1SDN4	< 0.005

**Table 2:** List of proteins from proteomic analysis which were down-regulated in rCEnC isolated and cultured in vitro if compared with the rCEnC isolated from the tissue, with a *p* value < 0.005 and < 0.001.

General function	Protein name	Protein symbol	Uniprot	*p* value
**Down-regulated in rCEnC**				
Amino-acid biosynthesis	S-adenosylhomocysteine hydrolase-like protein 1	AHCYL1	G1SXT1	< 0.005
Cotransporter regulator	Adenosylhomocysteinase 3	AHCYL2	G1T1W6	< 0.001
Sodium pump	Sodium/potassium-transporting ATPase subunit beta-1	ATP1B1	Q9TT37	< 0.005
calcium homeostasis	Plasma membrane calcium-transporting ATPase 4	ATP2B4	G1SR77	< 0.005
Glycosylation	Beta-1,3-glucosyltransferase	B3GLCT	G1T852	< 0.001
Chaperone	B-cell receptor-associated protein 31	BCAP31	G1TER3	< 0.005
Trafficking	BET1 homolog	BET1	G1TCU9	< 0.005
Uncharacterized	Protein CASC4	CASC4	G1SNS3	< 0.005
Cell proliferation, EMT	CDKN2A-interacting protein ( CDKN2AIP)	CARF	G1U8Q6	< 0.001
kinetochore attachment	Chromosome alignment-maintaining phosphoprotein 1	CHAMP1	G1SFQ2	< 0.005
Redox reactions	CDGSH iron-sulphur domain-containing protein 1	CISD1	G1SIP6	< 0.001
Cell adhesion/proliferation	Cytoskeleton-associated protein 4	CKAP4	G1SCT0	< 0.005
Collagen component	Collagen alpha-3(IV) chain	COL4A3	G1SCM1	< 0.005
Collagen component	Collagen alpha-6(IV) chain	COL4A6	G1T049	< 0.005
Crystallin/Chaperone like	Alpha-crystallin B chain	CRYAB	G1T4F9	< 0.005
Transcriptional regulation	Homeobox protein cut-like 1	CUX1	G1T9V2	< 0.001
ER homoeostasis	DDRGK domain-containing protein 1	DDRGK1	G1T3Q2	< 0.005
Chaperone	DnaJ homolog subfamily C member 1	DNAJC1	G1TIJ7	< 0.005
tRNA processing	Probable glutamate–tRNA ligase, mitochondrial	EARS2	G1SV59	< 0.005
soluble TGF-β co-receptor	Endoglin	ENG	G1SSF2	< 0.005
cytoskeletal function	Protein 4.1 ( protein 4.1 R)	EPB41	G1SRE6	< 0.005
cytoskeletal function	Band 4.1-like protein 2 ( protein 4.1 G)	EPB41L2	G1SG55	< 0.001
Tumour suppressor	Band 4.1-like protein 3	EPB41L3	G1TGX6	< 0.005
Tight junction	Junctional adhesion molecule A	F11R/JAMA	G1U305	< 0.001
Trafficking	Golgi SNAP receptor complex member 1	GOSR1	G1SG37	< 0.005
Glutathione peroxidase	Glutathione peroxidase 1	GPX1	P11909	< 0.005
phenylalanine catabolism	Homogentisate 1,2-dioxygenase	HGD	G1SWC2	< 0.005
Cellular pathway regulation	Homeodomain-interacting protein kinase 1	HIPK1	G1SKE7	< 0.005
Histone component	Histone H2B type 1-B	HIST1H2BB	G1SSZ8	< 0.005
Chaperone	Heat shock-related 70 kDa protein 2	HSPA2	G1T1V9	< 0.005
Inflammation	T-cell immunomodulatory protein	ITFG1	G1TA79	< 0.005
Cell-ECM interaction	Integrin beta-5	ITGB5	G1T7R4	< 0.005
RNA processing (neuronal)	KH domain, RNA-binding, signal transduction-associated 1	KHDRBS1	G1STI3	< 0.005
Trafficking	Kinectin	KTN1	G1SZR7	< 0.001
ECM Crosslink	Lysyl oxidase homolog 3	LOXL3	G1U7Y3	< 0.005
Immunosurveillance	Prolow-density lipoprotein receptor-related protein 1	LRP1/CD91	G1T4Z1	< 0.005
Trafficking	MGARP	MGARP	G1SR45	< 0.005
Tight junction	MAGUK p55 subfamily member 7	MPP7	G1T781	< 0.001
Uncertain	Myelin expression factor 2	MYEF2	G1T4L9	< 0.001
Amino-acid metabolism	Omega-amidase NIT2	NIT2	G1TXN1	< 0.005
Gene regulation	Pre-B-cell leukemia TF-interacting protein 1 ( PBXIP1)	HPIP	G1TB19	< 0.005
cell growth control	Polymerase delta-interacting protein 3	POLDIP3	G1TAY8	< 0.005
RNA processing	Paraspeckle component 1	PSPC1	G1SQF9	< 0.005
Transcriptional activator	Transcriptional activator protein Pur-alpha	PURA	G1T0N5	< 0.005
Trafficking	Ras-related protein Rab-39A	RAB39A	G1TZG4	< 0.001
Ribosome function	Ribosome-binding protein 1	RRBP1	G1U7C5	< 0.005
Trafficking	Secretory carrier-associated membrane protein 1	SCAMP1	G1SE20	< 0.001
Cell migration, proliferation	SPARC	SPARC	P36233	< 0.005
Cell migration, proliferation	SPARC-like protein 1	SPARCL1	G1SQE6	< 0.001
Trafficking	Signal recognition particle 54 kDa protein	SRP54	G1STX4	< 0.005
ER Ca^2+^ sensor	Stromal interaction molecule 1	STIM1	G1T594	< 0.005
Cell adhesion	Sushi, vWF A, EGF and pentraxin domain protein 1	SVEP1	G1TB95	< 0.005
RNA processing	Transcription elongation regulator 1	TCERG1	G1SQH2	< 0.005
Ribosome maturation	Treacle protein	TCOF1	G1SKJ8	< 0.005
Iron transport	Serotransferrin	TF	G1STF7	< 0.005
Uncertain	Thymocyte nuclear protein 1	THYN1	G1T302	< 0.005
Trafficking	Transmembrane emp24 domain-containing protein 3	TMED3	G1TJ80	< 0.005
nuclear chaperoning	Lamina-associated polypeptide 2, isoform alpha	TMPO	G1T3V5	< 0.001
Trafficking	Vesicle-associated membrane protein 2	VAMP2	G1TLZ2	< 0.005
Trafficking	Vesicle-associated membrane protein 4	VAMP4	G1TIV8	< 0.005
RNA processing	Zinc finger C3H1 domain-containing protein	ZFC3H1	G1U537	< 0.005

#### Gene ontology and literature analysis

Gene ontology analysis (Table 3) of the proteomic results revealed that some specific biological processes were significantly dysregulated, especially those involved in the formation of the proteasome complex, in the negative regulation of canonical Wnt signalling pathway, in the endoplasmic reticulum function, and in cellular trafficking (GO:0000502, GO:0090090, GO:0005789, GO:0006888, GO:0031201, GO:0031032 and KEGG pathway 04141). Other processes found in the gene ontology analysis, listed in Table 3, were not significantly modulated (*p* > 0.05).

**Table 3:** Gene ontology analysis using David database (https://david.ncifcrf.gov/) of the proteins identified in the proteomic study, Biological processes (GO terms or KEGG pathways) having a *p* value < 0.05 are significantly dysregulated.

Enrichment score	GO terms	Proteins	*p*
4.01	GO: 0000502-proteasome complex GO: 0090090-negative regulation of canonical Wnt signalling pathway	PSMD7, PSMD2, PSMD12, PSMD3, PSMD6, PSMB7	0.001 0.025
3.6	GO: 0005789-endoplasmic reticulum membrane	BCAP31, BET1, DDRGK1, DNAJC1, SEC24C, AHCYL1, B3GLCT, CKAP4, KTN1, PLA2G4A, STIM1, TMPO, TMED3	0.0089
2.33	GO: 0006888-endoplasmic reticulum to Golgi vesicle-mediated transport GO: 0031201-SNARE complex	BCAP31, BET1, SEC24C, GOSR1, TMED3, VAMP4 BET1, GOSR1, VAMP2, VAMP4	0.025 0.035
2.22	GO: 0031032-actomyosin structure organization	F11R, EPB41L2, EPB41L3, EPB41	0.0095
2.22	GO: 0005200-structural constituent of the cytoskeleton, GO: 0005856-cytoskeleton GO: 0003779-actin binding	ARPC3, EPB41L2, EPB41L3, EPB41 CKAP4, EPB41L2, EPB41L3, EPB41 EPB41L2, EPB41L3, EPB41	4.6E-1, 7.6E-1,9.9E-1
1.93	GO: 0051082-unfolded protein binding GO: 0006457-protein folding GO: 0051087-chaperone binding	DNAJA1, DNAJA2, CRYAB, HSPA2 DNAJA1, DNAJA2, CRYAB, DNAJC1 DNAJA1, DNAJA2, DNAJC1	4.6E-1, 5.7E-1, 7.2E-1
1.23	GO: 0005509-calcium ion binding	LRP1, SPARCL1, PLA2G4A, SPARC, STIM1, SVEP1	9.4E-1
1.14	GO: 0030198-extracellular matrix organization GO: 0007155-cell adhesion	F11R, COL4A3, COL4A6, ITGB5, SPARC, F11R, COL4A3, COL4A6, ITGB5, ATP1B1, ENG, SVEP1	2.2E-1, 2.8E-1
1.13	GO:0003676-nucleic acid binding	POLDIP3, CHAMP1, MYEF2, PSPC1, SYNCRIP	1.0E0
1.08	GO: 0006874-cellular calcium ion homeostasis	ATP1B1, ATP2B4, STIM1	6.7E-1
0.89	GO: 0098609-cell–cell adhesion GO: 0098641-cadherin binding involved in cell–cell adhesion	F11R, KTN1, MPP7, TMPO	8.7E-1, 8.8E-1

Enrichment	KEGG pathway	Proteins	*p*
1.93	04141: protein processing in endoplasmic reticulum	BCAP31, DNAJA1, DNAJA2, DNAJC1, SEC24C, CRYAB, CKAP4, HSPA2, RRBP1	0.00038

Literature analysis showed that some of these proteins have been previously associated with CE function and dysfunction (Supplementary Table S2) and that several of them have a role in multiple fundamental intracellular pathways (Table 4). More specifically, based on the proteomic results, we found numerous proteins differentially expressed in proliferating in vitro versus ex vivo cells, which were involved in β-catenin and TGF-β pathways.

**Table 4:** List of proteins from the proteomic analysis found to be involved, as an effector or as a regulator, in different cellular pathways: Wnt/β-catenin, AKT, TGF-β and NF-kB.

Pathway	Protein name	Protein Symbol	*p* value
**Upregulated in rCEnC**			
β-catenin^83^	Actin-related protein 2/3 complex subunit 3	ARPC3	< 0.001
AKT^84^	Protein transport protein Sec24C	SEC24C	< 0.005
**Downregulated in rCEnC**			
β-catenin^36^	CDKN2A-interacting protein	CDKN2AIP/CARF	< 0.001
β-catenin^35^, TGF-β^47^	Homeobox protein cut-like 1	CUX1	< 0.001
NF-kB^85^	DDRGK domain-containing protein 1	DDRGK1	< 0.005
TGF-β^44,45^, β-catenin^34^	Endoglin	ENG	< 0.005
β-catenin^39^	Protein 4.1	EPB41/protein 4.1 R	< 0.005
β-catenin^39^	Band 4.1-like protein 2	EPB41L2/protein 4.1 G	< 0.001
β-catenin^31,70^	Homeodomain-interacting protein kinase 1	HIPK1	< 0.005
β-catenin^33^	Integrin beta-5	ITGB5	< 0.005
NF-kB^86^, β-catenin^32^	KH domain-containing, RNA-binding, signal transduction-associated protein 1	KHDRBS1/SAM68	< 0.005
TGF-β1^51^	Prolow-density lipoprotein receptor-related protein 1	LRP1/CD91	< 0.005
AKT^87^, TGF-β^52^	Pre-B-cell leukaemia transcription factor-interacting protein 1	PBXIP1/HPIP	< 0.005
TGF-β1^50^	Paraspeckle component 1	PSPC1	< 0.005
AKT^88^	Ras-related protein Rab-39A	RAB39A	< 0.001
β-catenin^38^	SPARC	SPARC/Osteonectin	< 0.005
β-catenin^37^	SPARC-like protein 1	SPARCL1	< 0.001
AKT^89^	Stromal interaction molecule 1	STIM1	< 0.005

Several proteins involved in promoting the Wnt/β-catenin pathway were significantly downregulated in cultured rCEnC when compared with the tissue-derived counterpart. For instance, HIPK1 was previously shown to induce Wnt/β-catenin signalling^31^. KHDRBS1 promotes proliferation through the same pathway^32^, while ITGB5 inhibits β-catenin degradation by the proteasome, leading to Wnt/β-catenin pathway activation as well^33^. Similarly, Endoglin (ENG)^34^, CUX^35^, CARF^36^, SPARCL1^37^, and SPARC^38^ were proved to trigger the Wnt/β-catenin pathway. Downregulation of proteins 4.1R (EPB41) and 4.1G (EPB41L2), which associates β-catenin to the cell membrane in gastric epithelium^39^, may play a role in cytoplasmic β-catenin translocation. Moreover, DAVID analysis predicted a negative regulation of the canonical Wnt signalling pathway (Table 3), identifying some proteasome proteins that were upregulated in rCEnC (PSMD7, PSMD2, PSMD12, PSMD3, PSMD6, and PSMB7). The PSMD are components of the 19S subcomplex, known to be involved in β-catenin/Wnt signalling; PSMD7 and PSMD2 in particular are found to be directly associated with the degradation of β-catenin from the proteasome^40^.

Although Wnt/β-catenin is involved in CE development^41^ and in CE mesenchymal transformation^26,28,42^, the mechanism of β-catenin pathway downregulation observed here is not completely clear and it will be further dissected in the next sections.

Multiple proteins that were found downregulated in cultured rCEnC are also involved in TGF-β pathway regulation. Endoglin, previously shown to be expressed in CEnC^43^, is an auxiliary receptor for TGF-β, controlling proliferation and quiescence^44^, in particular in the endothelium^45^, where it also regulates the EMT^46^. CUX1, a protein with a role in regulating cell cycle progression^47^, EMT^48^, and repressing E-cadherin^49^, is proved to be a target of TGF-β pathway^47^ and PSPC1 potentiates TGF-β autocrine signalling^50^. Similarly, expression of LRP1, a cell receptor involved in the clearance of growth factors, including TGF-β^51^ is increased upon stimulation of TGF-β1^51^, and HPIP is a downstream factor of TGF-β1 stimulation, promoting EMT in A549 cells^52^.

These results show that Wnt/β-catenin modulators and TGF-β effectors are downregulated in culture-derived proliferating rCEnC and, in accordance with previous reports, demonstrate a fine-tuned regulation involved in promoting CEnC proliferation while still suppressing the EnMT.

#### β-Catenin cellular localization

**Figure 2:**
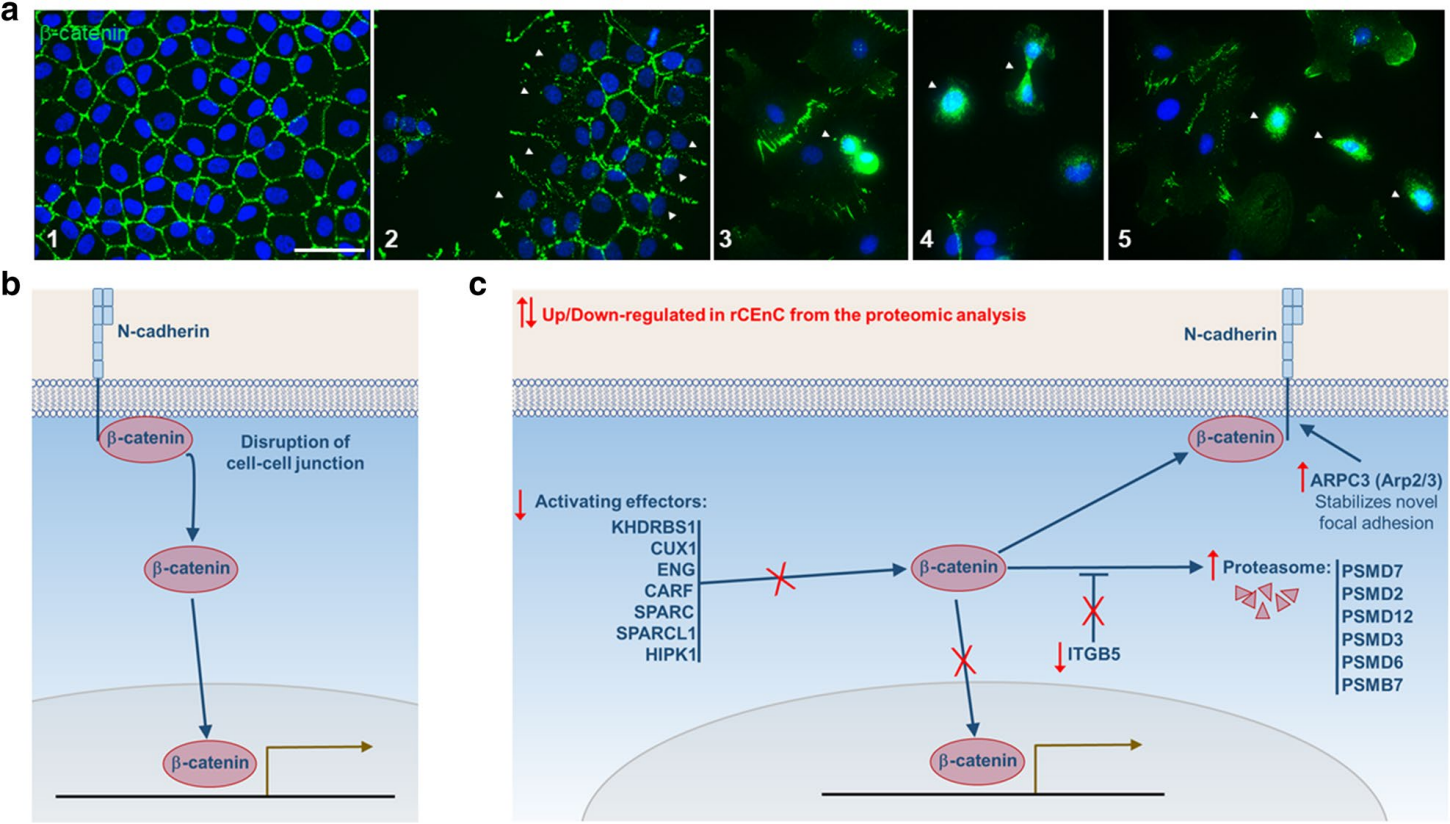
Regulation of β-catenin in cultured rCEnC. (**a**) Immunofluorescence of rCEnC in culture: DAPI in blue, β-catenin in green. White arrows indicate in: (2) the lack of β-catenin staining in external cells not having any contact with other cells, (3, 4, 5) β-catenin nuclear localization in duplicating and isolated cells. Confluent cells (1) showed a complete membrane localisation of β-catenin. Scale bar 50 µM. (**b**, **c**) Scheme representing the possible role of β-catenin in ex vivo CE and in cultured cells and their interaction with some proteins identified to be dysregulated from the proteomic analysis, as suggested by literature analysis (Table 4). (**b**) Hypothetic representation of the initial phase of CEnC proliferation in which β-catenin is released from the membrane (where it interacts with N-cadherin) and moves to the nucleus in order to promote proliferation. (**c**) A second phase, when β-catenin is no more promoting cellular proliferation and is (i) degraded from the proteasome, or (ii) Inactivated through the downregulation of β-catenin effectors, or (iii) moved back to the membrane (once the cell–cell junctions are re-established).

Staining of rCEnC showed that β-catenin is mainly located in the plasma membrane when cultured cells are confluent and reconstitute the tissue-like alveolar structure (Fig. 2a, panel 1). Nevertheless, the external cells of the colonies, not presenting any cell–cell contact, do not show β-catenin on the plasma membrane (Fig. 2a, panel 2). Most importantly, cytoplasmic and nuclear translocation of β-catenin from the plasma membrane was observed in the dividing or in some isolated cells (Fig. 2a, panels 3–5). These data, together with available literature, suggest a complex mechanism containing two phases. In the first phase, β-catenin is released from the cell-junctions, moving eventually to the nucleus for regulation of the gene expression. In the second phase, it is degraded or transferred back to the membrane, while its activators are downregulated, as observed in the proteomic results (Fig. 2b,c).

We suggest this hypothesis based on the observation of β-catenin translocation in the first phase, which can also explain why it was quickly degraded and subsequently downregulated.

#### Variability in the cell cycle and β-catenin distribution after treatments with bFGF and TGF-β

In order to increase or diminish the number of cells in the G2/M phase of the cell cycle and analyse in parallel the effect on β-catenin nuclear translocation as well as the maintenance of a corneal endothelial phenotype, we treated rCEnC with different compounds.

**Figure 3:**
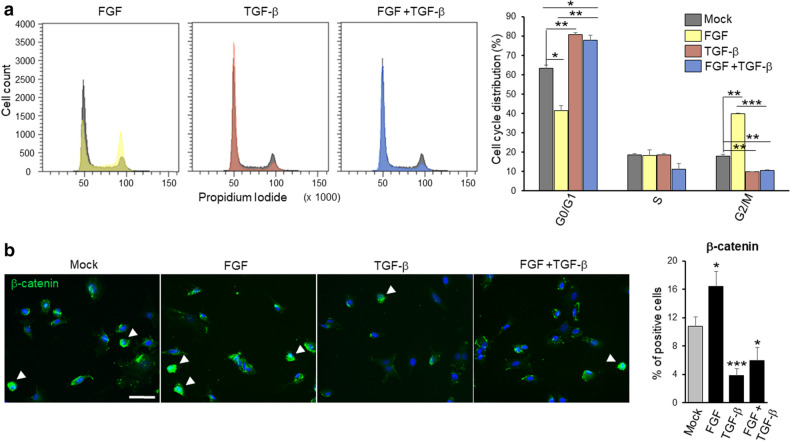
Effect of bFGF and TGF-β on rCEnC (**a**) the panel on the left shows representative graphs of cell cycle distribution identified by Propidium Iodide flow cytofluorimetric analysis of the treatments with bFGF, TGF-β and bFGF + TGF-β, respectively. For each graph, the coloured peaks represent the distribution after the treatment, while the dark grey peaks indicate the distribution of cells in the mock sample. In the right panel, a bar chart shows the quantification of all the replicates (n = 3), comparing the different treatments and the mock control in the three phases of the cell cycle. Colours are maintained constant in both peak and bar charts. They refer to the three treatments and the mock control, as summarised in the top right corner of the figure. The y-axis represents the mean percentage of cells in each phase of the cell cycle. Results are presented as mean ± SE. T-test was performed *p < 0.05, **p < 0.01, ***p < 0.001. (**b**) The panel on the left shows representative immunofluorescence images of β-catenin (green) in the four conditions, the same listed in (**a**). White arrows indicate the cells in which β-catenin translocated to the nuclei. In blue DAPI, scale bar 50 µM. In the right panel, the bar chart shows the percentage of cells in which β-catenin moved to the nuclei as a mean of 12 fields (n = 3 biological replicates) for each condition. Results are presented as mean ± SE. T-test was performed n.s. non-significant, *p < 0.05, **p < 0.01, ***p < 0.001.

Initially, bFGF was used as a positive control for proliferation induction in CEnC^19^ and its cross-regulations with the Wnt pathway, as observed in other cellular models^53^ and in CEnC^26^. Moreover, bFGF signalling was shown to downregulate GSK-3β activity through a mechanism involving Akt, which in turn activates β-catenin response^54^. As expected, rCEnC treated with 20 ng/mL of bFGF showed a significant reduction in the number of non-proliferating cells and a consequent increase in the G2/M population (Fig. 3a).

In parallel, TGF-β was tested on rCEnC as it correlates with multiple proteins that emerged from the proteomic analysis and has an ascertained role in reducing CEnC proliferation^17,18,55–58^. Herein, TGF-β-treated cells presented a significant decrease in G2/M phase of the cell cycle as it was detected by using cytofluorimetric analysis, confirming the anti-proliferative effect of TGF-β on CEnC. When TGF-β was added to bFGF-stimulated cells, we observed a distribution comparable to the TGF-β alone treatment, proving the TGF-β interference with the bFGF-activated pathway (Fig. 3a).

The same treatments were analysed for β-catenin expression by immunocytochemistry. We detected a significant increase of β-catenin nuclear translocation in bFGF-treated cells, while the cytoplasmic signal was significantly reduced both in TGF-β and TGF-β + bFGF-treated rCEnC when compared to the control (Fig. 3b). These data collectively proved that the activity of bFGF was mediated by an increased translocation of β-catenin to the nucleus, which is inhibited by TGF-β, in accordance with the previous literature^19^.

**Figure 4:**
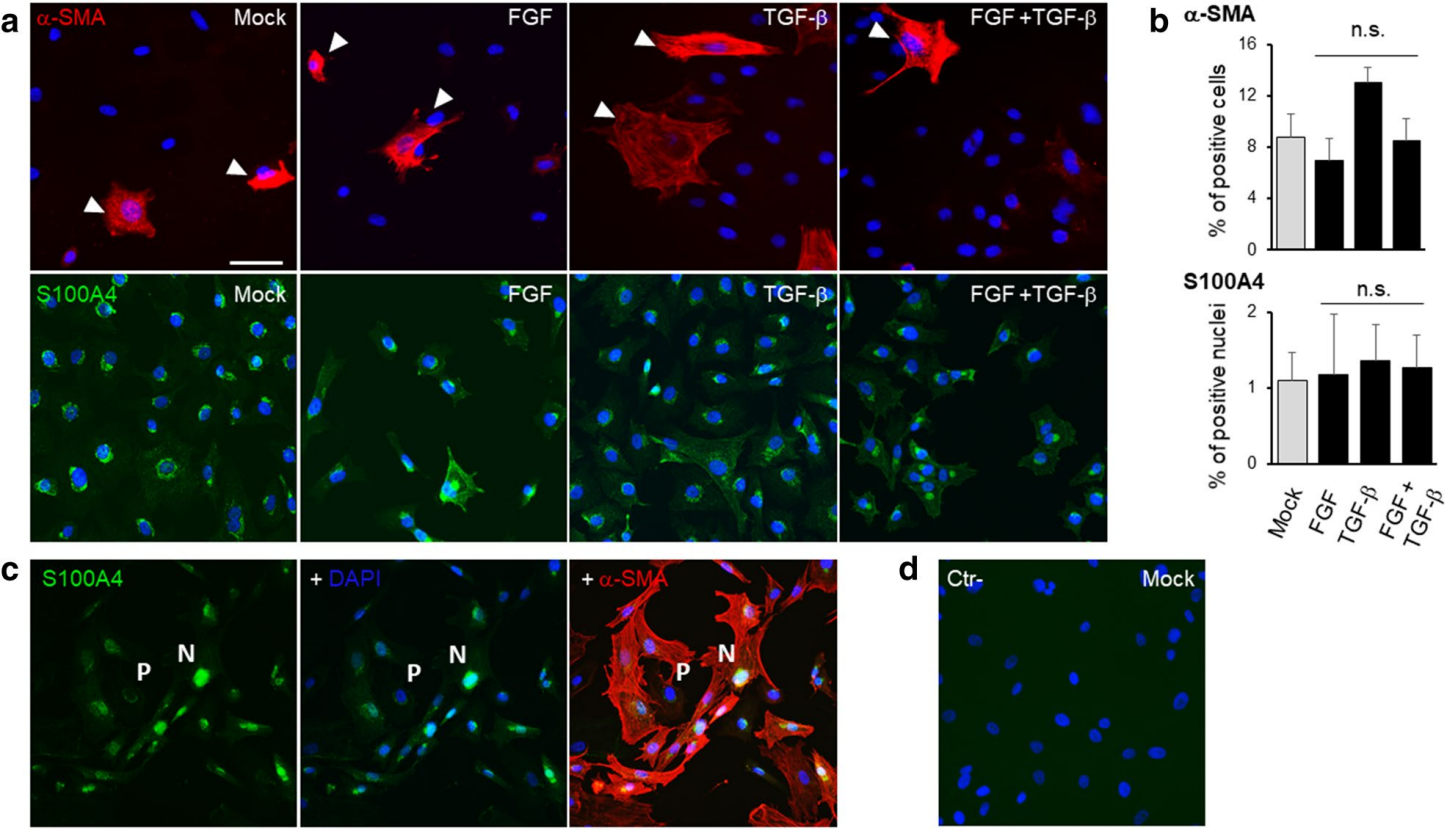
EnMT investigation upon bFGF and TGF-β on rCEnC. (**a**) The panel shows representative immunofluorescence images of α-SMA (red, first row) and S100A4 (green, second row) in rCEnC treated with Mock control, bFGF, TGF-β and bFGF + TGF-β, respectively. White arrows indicate the cells positive for α-SMA. In blue DAPI, scale bar 50 µM for all the images. (**b**) The bar chart on the right shows the percentage of cells positive for α-SMA and the percentage of cells in which S100A4 moved to the nuclei as a mean of 12 fields (n = 3 biological replicates) for each condition. Results are presented as mean ± SE. T-test was performed n.s. non-significant. (**c**) The panel illustrates a representative image of a double immunostaining with S100A4 in green, DAPI in blue and α-SMA in red of rCEnC at a high passage number (P10). Letters P, perinuclear, and N, nuclear, underlie the different localization of S100A4 staining, corresponding to a low and high α-SMA positivity, respectively. (d) The panel shows a secondary only control on Mock rCEnC, used as a negative control with DAPI in blue.

The studies by immunofluorescence on α-SMA (Fig. 4), a marker of EMT, did not show any significant evidence of mesenchymal transformation. The amount of α-SMA-positive cells was not modified by bFGF treatment, as similarly assessed by Tseng and Heur groups^26,59^. Although TGF-β can induce EnMT^42,60^ and differently from what observed by Tseng et al*.*^26^, the treatment of rCEnC with either TGF-β and TGF-β + bFGF did not show any significant increase in α-SMA expression. Nevertheless, in the Tseng et al*.* study, the treatment lasted 3 days and was followed by EDTA dissociation, likely provoking a more sustained stimulation of β-catenin as well as other cellular responses.

The data obtained with α-SMA were corroborated by immunostaining with an early marker of EMT, S100A4^61,62^, expressed within the cytoplasm by human adult CEnC *in vivo*^63^. Conversely, S100A4 expression was observed in the nucleus when CEnC underwent EnMT^26^. Similarly to what observed for α-SMA, we did not detect any significant variation in S100A4 expression between the treatments with bFGF, TGF-β, TGF-β + bFGF and the Mock control (Fig. 4a,b). In each treatment tested S100A4 presented as mainly cytoplasmic and/or perinuclear. As a positive control we used a rCEnC strain with a high number of passages (P10) which showed an elongated phenotype. In this condition S100A4 was localized in the nuclei of the majority of rCEnC, which were also showing a high α-SMA positivity (Fig. 4c). This result confirm that both proteins may be considered as valid markers for EnMT.

Altogether, these results showed that bFGF and TGF-β treatments did not cause any mesenchymal transformation on rCEnC at 24 h, although they were able to interfere with β-catenin and activate or inhibit proliferation. Further experiments in the following section, using small molecules targeting β-catenin pathways, helped to reveal a possible role of this crosstalk in rCEnC maintenance and propagation.

#### Variation in the cell cycle phases and β-catenin distribution after treatments with Wnt activators/inhibitors

**Figure 5:**
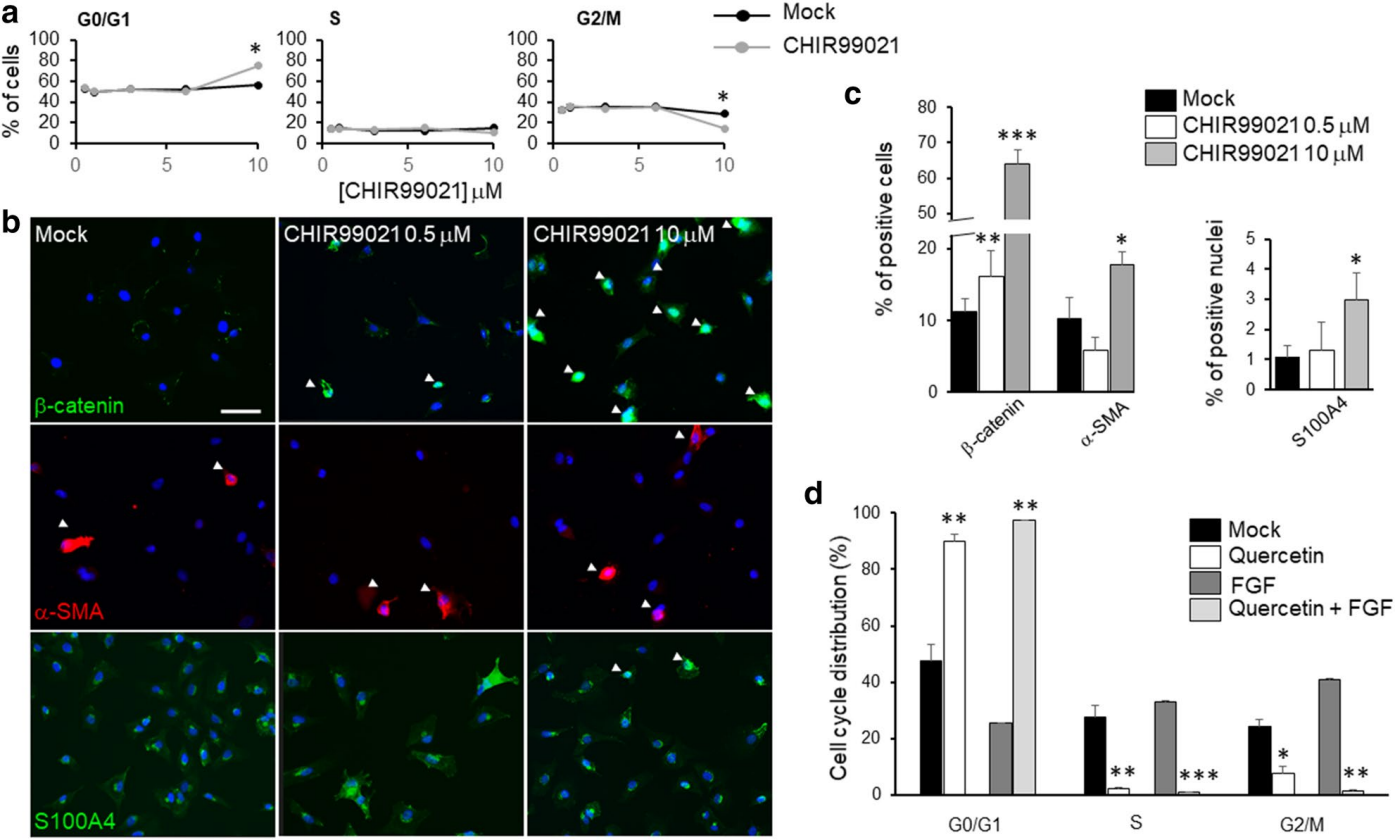
Effects of β-catenin activator/inhibitor. (**a**) Percentage of cells in the three phases of the cell cycle identified by Propidium Iodide flow cytofluorimetric analysis, mock is represented with black lines and dots, treated (CHIR99021 at different concentrations) with grey lines and dots. Experiments were performed n = 3 at 0.5, 1 and 10 µM, twice at 3 and 6 µM. Results are presented as mean ± SE. T-test was performed *p < 0.05, **p < 0.01. (**b**) Representative immunofluorescence images of rCEnC treated with CHIR99021 at 0.5 and 10 µM, in comparison with mock control in the left panel. The top row shows expression of β-catenin (green), the second row is α-SMA (red), the third is S100A4 (green) while blue is DAPI. White arrows indicate cells in which β-catenin translocated to the nuclei and cells that express α-SMA. Scale bar 50 µM. The panel (**c**) on the right shows a bar chart for the quantification of the immunofluorescence analysis (in % of cells) of cells having β-catenin in the nuclei, expressing α-SMA or presenting S100A4 in the nuclei (12 fields and n = 3 replicates). Results are presented as mean ± Standard Error (SE). T-test was performed *p < 0.05, **p < 0.01, ***p < 0.001. (**d**) Represents a bar chart of Propidium Iodide flow cytofluorimetric analysis for quercetin treatment of rCEnC. Experiments were performed n = 3. Results are presented as mean ± SE. T-test was performed *p < 0.05, **p < 0.01, ***p < 0.001.

CHIR99021 was previously described to inhibit GSK-3β^64^, thereby stabilizing cytoplasmic β-catenin and eventually promoting its nuclear translocation. On the basis of CHIR99021 IC50 (0.04 µM)^64^, the treatment was initially tested in a range of concentrations between 0.05 and 10 µM. The distribution in the cell cycle phases was not statistically different for all the CHIR99021 concentrations tested except for 10 µM. This concentration produced a significant decrease of cells in the G2/M phase of the cell cycle in comparison with untreated cells (Fig. 5a), although promoting a consistent β-catenin nuclear translocation (Fig. 5b,c). Interestingly, CHIR99021 at 0.5 µM, despite not showing any significant difference in cell phases distribution, revealed an increase in cytoplasmic and nuclear β-catenin if compared with the untreated cells (Fig. 5b,c). Collectively these results suggest that CHIR99021, although able to promote β-catenin migration to the nuclei, did not cause an increase in rCEnC proliferation. Conversely, at high concentration (10 µM), CHIR99021 decreased rCEnC proliferation. This unexpected effect might be due to a negative feedback regulation of β-catenin, once overactivated. The possibility of β-catenin feedback regulation was also previously proposed by Hirata‐Tominaga et al*.*^28^. CHIR99021 concentrations of 0.5 and 10 µM were also tested at 12 and 48 h: while at 12 h both concentrations did not show any effect on the cell cycle of rCEnC, at 48 h only 10 µM CHIR99021 elicits an effect not significantly different to what observed at 24 h (data not shown). Moreover, a vitality assay using calcein AM/Propidium Iodide staining was performed to assess if CHIR99021, at the concentrations used for the experiments on rCEnC, demonstrated a cytotoxic effect. However, we could not observe any Propidium Iodide positive cell nuclei, confirming that CHIR99021 and DMSO, at all the concentration tested under the same experimental conditions used in this study, did not induce cell death (Supplementary Figure S3).

In addition, we observed an increased amount of α-SMA positive cells and nuclear S100A4 at 10 µM CHIR99021 (Fig. 5b,c), suggesting that activation of β-catenin nuclear translocation over a certain limit may induce EnMT. Similar results were previously obtained via disrupting CEnC junctions with EDTA and with prolonged addition of TGF-β (2 days)^26^. Under these conditions, rCEnC might thus interfere with the β-catenin pathway to avoid the loss of hexagonal morphology and the mesenchymal transformation.

Next, we tested an inhibitor of β-catenin pathway: quercetin^65,66^, previously proven to inhibit the Wnt/β-catenin signalling by interfering with β-catenin nuclear translocation^67^. The 25 µM concentration was selected for our study. Quercetin was shown here to completely abolish the rCEnC populations both in S and G2/M phases, maintaining the cells in the G0/G1 phase of the cell cycle (Fig. 5d). Similar results were obtained by treating rCEnC with both bFGF and quercetin (Fig. 5d), highlighting how the latter was also able to knockdown the proliferative effect of bFGF. These data suggest that quercetin may act, at least in part, via the same pathway activated by bFGF.

Overall, the presented results show how β-catenin appears necessary but not sufficient to promote proliferation of rCEnC. The β-catenin pathway is activated by the disruption of cell–cell junction and β-catenin expression is increased by bFGF, while it is inhibited by the presence of TGF-β. However, β-catenin is quickly degraded by cells, underlining the sophisticated regulation of this specific pathway in the corneal endothelium, with the likely effect to avoid EnMT.

### Discussion

Several studies have been carried out with the aim to characterize how CEnC regulate their proliferation, mainly focusing on the role of specific growth factors (EGF^68^, bFGF^19^, and TGF-β^55,56^) and the final downstream effectors, in particular those involved in the regulation of the cell cycle (cyclins, p16, p21, and p27^2,57^). Herein we propose a hypothesis-free approach, starting directly from the analysis of the CE proteome through a correlation between ex vivo non-proliferating rCEnC and in vitro proliferating rCEnC. Using rabbits farmed in standard conditions (controlled environment, slaughtered at the same age) and correlating the two eyes of the same individual, we were able to minimize the inter-individual variations. Moreover, although cellular division was observed in rabbit CE in vivo^69^, human and rabbit CEnC showed a similar cell cycle distribution, either when isolated from the tissue or when cultured in vitro. For this reason, rCEnC were selected as a model to study proliferative mechanisms that are utilised by the cells when expanded in vitro. The proteomic analysis allowed the selection of 77 proteins, out of the 3,328 identified, with a significant differential expression between the two groups in comparison (*p* < 0.005). A targeted dissection of each identified protein and their related intracellular routes, enabled to propose a correlation between specific pathways and rCEnC expansion in vitro.

For instance, several proteins promoting activation of the Wnt/β-catenin pathway, including HIPK1^31,70^, KHDRBS1^32^, ITGB5^33^, Endoglin (ENG)^34^, CUX^35^, CARF^36^, SPARCL1^37^, and SPARC^38^, were all significantly reduced in cultured rCEnC. Negative regulation of the canonical Wnt signalling pathway was further confirmed by upregulation of some proteasome proteins (PSMD7, PSMD2, PSMD12, PSMD3, PSMD6, and PSMB7; Table 3)^40^.

Beside Wnt/β-catenin, multiple proteins involved in the TGF-β signalling were found to be downregulated. In particular, the expression of TGF-β effectors like Endoglin^44–46^, CUX1^47–49^, LRP1^51^, HPIP^52^, and PSPC1^50^ was significantly reduced in rCEnC.

Taken together, the proteomic characterization suggested that Wnt/β-catenin and TGF-β might have a key function in proliferating rCEnC (Table 4) and garnered therefore an increasing attention in this study.

Proteins of the TGF-β family are cytokines activated upon different stimulation (i.e., injury or mechanical stress)^5^. In the cornea, TGF-βs (1, -2, and -3) are present in the aqueous humor^71^ and, once released, trigger multiple downstream processes^72^. A double role was recently described in CEnC for TGF-β: it induces the correct morphology and formation of novel cell junctions during the maturation phase, while it promotes a fibroblastic phenotype during proliferation^23^. This double role reflects its already observed activity in other studies, where TGF-βs is involved in the correct maturation of CE^41^, in stimulating CEnC migration during wound healing^26^, in promoting EnMT^42,60^ and, most importantly, in suppressing CEnC entry into the S-phase^17,18,55–58^.

Following the proteomic results, downregulation of some TGF-β effectors observed in dividing rCEnC may be consequent to the absence of a TGF-β stimulus in vitro: cultured rCEnC, no longer blocked in a non-proliferative state by TGF-β, acquire in this manner the ability to proliferate.

The other pathway arising from the proteomic analysis involves Wnt/β-catenin signalling. When the cells are in contact with each other, β-catenin is usually bound to the cell membrane in complex with cadherin (N-cadherin in CE^73^) while it is released when cells lose their cell–cell junctions^74^. Once in the cytoplasm, free β-catenin is targeted for degradation by GSK-3β phosphorylation. However, when Wnt activates the canonical pathway, it promotes the stabilisation of the cytoplasmic β-catenin and eventually, its translocation into the nucleus^5^, where it drives transcription of target genes, in particular of those that are critical in promoting cell proliferation, such as c-myc and cyclin D1^75^. Proliferation is known to be induced by β-catenin activation in different cell types, including cancer^76^ and stem cells^74,77^. In CEnC, β-catenin acts as a key regulator of two crucial mechanisms: proliferation and cellular morphology^26,27^ but also in expressing CE markers, as observed following treatment with a GSK-3β inhibitor (6-bromoindirubin-3′-oxime, BIO) which increases β-catenin levels^41^.

Herein we attempt to explain the role of β-catenin during rCEnC proliferation and uncover the reasons of its rapid degradation in the cytoplasm via cellular machinery. In fact, we observed binding of β-catenin to the cell membrane in confluent cells in vitro, while it disappeared from the membrane when the cell–cell junctions were lost (Fig. 1). This result was confirmed by the proteomic characterization that indicated activation of proteasome components, previously linked to β-catenin degradation^40^ (Tables 1, 2, 3). Moreover, the majority of cells in culture were shown to downregulate various proteins involved in β-catenin activation. Of those proteins, Endoglin^43^, ITGB5^78^, and SPARC^79^ were previously found to be expressed in CEnC (Supplementary Table S2). β-catenin is indeed generally fast degraded once in the cytoplasm^75^, and the condition captured by the proteomic analysis showed how β-catenin may have been already digested by the proteasome or moving back to the cell membrane (Fig. 2b,c). At the same experimental condition, half of rCEnC population (60% confluence) was in the G0/G1 phase, while only a minority of them were actively proliferating in the G2/M phase of the cell cycle (Fig. 1b). However, within this population, we could observe a nuclear β-catenin localisation in isolated or mitotic CEnC (Fig. 2a), suggesting the importance of this translocation during cell division.

Collectively, these results raised questions regarding the possible role of β-catenin in rCEnC expansion that we tackled using a GSK-3 inhibitor, CHIR99021, to induce β-catenin stability, and quercetin as a counteracting inhibitor of β-catenin nuclear translocation^67^. Treating rCEnC with quercetin, we observed a substantial decrease in the cells in the G2/M phase of the cell cycle (Fig. 5d), while we never found a corresponding increased amount of cells in the G2/M phase at all tested CHIR99021 concentrations (Fig. 5a). Conversely, CHIR99021 (at 10 and 0.5 µM) was able to raise drastically the number of cells where β-catenin had translocated to the nucleus (Fig. 5b,c). Taken together, these data confirm how β-catenin expression and localization are fundamental during cell proliferation process, in accordance with previous reports^26,27^, but probably not sufficient to promote it.

Based on the described role of β-catenin in dividing rCEnC and on the effect of growth factors in eliciting in vitro CEnC proliferation^19^, we sought to understand whether specific growth factors would act via β-catenin activation. β-catenin was shown to be involved in orchestrating the downstream activity of FGF in various cell types^54,80^, and, as previously introduced, bFGF stimulates CEnC proliferation through PI3K/Akt activation^4,19–21^, while TGF-β^19,22^ inhibits cell proliferation elicited by bFGF^54^. Here we investigated how the pro- and anti-proliferative activities of these two specific growth factors might crosstalk through β-catenin signalling. Consistently with previous reports, we confirmed the role of bFGF in promoting CEnC proliferation^19,26^ showing how bFGF mediates an increase in the G2/M phase of the cell cycle and in β-catenin nuclear translocation (Fig. 3). Moreover, bFGF pro-proliferative effect was completely abolished by the quercetin-mediated inhibition of the β-catenin pathway (Fig. 5d). Similarly, we assessed the anti-proliferative effect of TGF-β^17,18,55,56^, which exerted an analogous decrease in the number of cells in the G2/M phase either alone or in the presence of bFGF, thus abolishing any pro-proliferative effect of the latter (Fig. 3a). We proved for the first time here that this effect, described also by Lu and collaborators^19^, is mediated by the inhibition of β-catenin translocation to the nucleus (Fig. 3b). Collectively, the results reported in our study confirmed the importance of β-catenin during CEnC proliferation process both after cell–cell disruption and bFGF stimulation. Consistent with this finding and with previous reports, TGF-β inhibits proliferation by blocking β-catenin nuclear translocation.

As introduced earlier, the proliferation process is often balanced in a delicate equilibrium with EnMT. Although more specific experiments are necessary to clarify the role of β-catenin in EnMT, we measured a significant increase in the number of α-SMA and S100A4 (markers of EMT) positive CEnC when treating cells with 10 µM CHIR99021 (Fig. 5b,c). Such evidence suggests that a sustained stimulation with CHIR99021 may induce β-catenin nuclear translocation and that EnMT ensues when β-catenin is activated over a certain threshold. Similarly, Zhu et al. confirmed how CEnC loosing cell–cell junctions after treatment with EDTA exhibit a defined nuclear localization of β-catenin, promoting proliferation but also EnMT^26^, whereas Kinoshita et al. observed that CEnC attempt to block EnMT by deregulating β-catenin^28^. Altogether these results confirmed that β-catenin expression requires fine regulation since a sustained stimulus towards β-catenin activation might induce CEnC transformation into a mesenchymal phenotype, similar to cancer progression^75^. This observation may also explain why CEnC degrade β-catenin in the cytoplasm and deregulate β-catenin-activating pathways immediately after cell division, enabling thereby a putative CEnC mechanism of protection from β-catenin induced EnMT.

The data presented here suggest a scenario where β-catenin is necessary for CEnC proliferation, but its over-activation drives cells to EnMT. However, β-catenin alone is not sufficient to unlock the mitotic block requiring other cellular effectors, as those activated by the disruption of cell–cell membranes and stimulated by growth factors. For instance, RAC1 has been proposed to act in concert with β-catenin in inducing CEnC proliferation through Cyclin D1 transcriptional activation^27^.

In conclusion, herein we present a study that, starting from a proteomic analysis, provides insights into the major intracellular pathways that CEnC activate in culture. Further studies of these mechanisms are fundamental to understand how to unlock the CEnC mitotic block and improve their regenerative capacity in order to develop a localised therapy that would overcome the need for corneal transplantation.

## Materials and methods

### Corneal endothelial cell harvesting and culture

Human corneas, preserved in Eusol at 4 °C, were selected for experiments with the following criteria: age ranging from 49 to 78 years old, no history of corneal diseases, CEnC density greater than 1,800 cells/mm^2^, death to preservation interval lower than 15 h and used for experiments within 10 days from death. CEnC isolation was performed following washing in Dulbecco’s phosphate-buffered saline (DPBS; Thermo Fisher Scientific, USA), Descemet’s stripping and subsequent digestion with 1 mg/ml Collagenase A (Roche, USA) in DMEM (Thermo Fisher Scientific, USA) for 3 h at 37 °C. Isolated cell tangles were then further dissociated with TrypLE (Thermo Fisher Scientific, USA) for 5 min at 37 °C. After that, the cells were pelleted at 1,200 rpm for 3 min and harvested for direct cytofluorimetric analysis or plated after coating the wells with FNC Coating mix (AthenaES, USA). Growth medium was composed of OptiMEM-I (Thermo Fisher Scientific, USA), 8% HyClone fetal bovine serum (FBS; FisherScientific, USA), 5 ng/mL epidermal growth factor (EGF; Thermo Fisher Scientific, USA), 20 μg/mL ascorbic acid (Sigma-Aldrich, USA), 200 mg/L calcium chloride (Sigma-Aldrich, USA), 0.08% chondroitin sulphate (C4384, Sigma-Aldrich, USA), and penicillin/streptomycin (Euroclone, Italy). Human CEnC were cultured at 37 °C in 5% CO_2,_ and the medium was changed every 2 days.

Corneas from white New Zealand rabbits (3 months old, equivalent to a human age of 10 years), obtained from a local slaughterhouse (Maini SRL Modena), were used for proteomic purposes and subsequent analyses.

Corneas were harvested the same day of euthanization and processed within 24 h. Intact Descemet’s membrane was stripped off the corneas and transferred to Accutase (ECB3056D, Euroclone, Italy) for 20 min at 37 °C. Isolated corneal endothelial cells were then pelleted at 1,200 rpm for 3 min. The fifteen pellets obtained from the right eyes of each rabbit were washed in DPBS, the Descemet’s nude tissue was removed, and the cells were immediately frozen at − 80 °C after a second centrifugation. The fifteen corresponding left eyes were used for cultures, expanding the cells in 6 well plates coated with FNC Coating Mix with using the same medium as for human CEnC, changing it every 2 days, at 37 °C in 5% CO_2_. Upon confluence, the cells were rinsed in DPBS and passaged at ratio of 1:2 or 1:3 with TrypLE for 10–15 min at 37 °C in 5% CO_2_. Cells at 60% confluence at passage 1 were harvested, washed in DPBS, and pellets were frozen at − 80 °C for the proteomic analysis. In all other experiments cells were used between the first and the third passages, when the morphology was maintained perfectly polygonal, and always compared with their internal control.

### Cell cycle analysis by flow cytometry

The cell cycle distribution was studied using Propidium Iodide (PI) staining (Sigma-Aldrich). rCEnC from tissue or cell culture were washed with DPBS and incubated in 300 μL of a PBS solution containing PI 50 µg/mL, Triton X-100 (Bio-Rad, USA) 0.1% for 1 h at 4 °C in the dark. After staining, cells were analysed using BD FACSCanto II (BD BIOSCIENCES; San Jose, CA USA). For each sample, 20,000 events were counted and considered for the analysis to ensure statistical relevance. Results were analysed with a ModFit 3.0 software.

### Proteomic analysis

Cell pellets were defrosted from − 80 °C and proteins were isolated using RIPA buffer, supplemented with phosphatase I, protease I, and EDTA (all from Thermo Fisher Scientific, USA), following the manufacturer’s instructions.

Total protein (20 µg) was reduced with DTE, alkylated with iodoacetamide (Sigma-Aldrich), and digested with trypsin. Aliquots of the sample containing tryptic peptides were desalted using StageTip C18 (Merck Millipore, Italy), injected into and separated by UPLC, and analysed using nLC-MS/MS UPLC in line with a mass spectrometer Q-Exactive (Thermo Fisher Scientific, Germany). Peptide separations occurred on a reverse-phase silica capillary column (75 μm i.d., 15 cm long), packed with 1.9-μm ReproSil-Pur 120 C18-AQ (Dr. Maisch GmbH, Germany). A gradient of eluents A (LC–MS grade water containing 0.1% v/v formic acid) and B (acetonitrile containing 0.1% v/v formic acid) was used to achieve separation of peptides (300 nL/min flow rate), from 2 to 40% B in 88 min. Full scan spectra were acquired with the lock-mass option, resolution set to 70,000, and mass range from m/z 300 to 20,000 Da. The ten most intense doubly and triply charged ions were selected and fragmented in the ion trap, using a resolution of 17,500. All samples were analysed in technical replicates. MaxQuant software (v 1.6.1.0) was used in order to perform a label-free quantification, based on the intensity of the precursors, to identify the proteins in the complete rabbit proteome 20,190,508. Statistically significant differences between the two sets of samples were identified using the software for statistical analysis MeV v. 4.9.0. T-test was selected for the comparison and proteins differently represented in the two conditions, with p-values lower than 0.001 and 0.005, were screened for further analysis.

### Gene ontology analysis

Gene Ontology (GO) analysis was performed in order to identify enriched biological themes on the toweb-based DAVID Bioinformatics Resources v6.8 (NIAID, NIH, USA). Adjusted Benjamini P values, calculated by the software using the Modified Fisher Exact test, were measured for each theme. Smaller P values represent higher significance of enrichment.

### Immunofluorescence on cultured rCEnC

rCEnC were plated on glass coverslip treated with FNC Coating Mix, in parallel to each cytofluorimetric analysis. After one rinse in DPBS, they were fixed in methanol for 10 min at − 20 °C, washed twice in PBS, and then preserved at 4 °C until staining. The non-specific binding sites were saturated with blocking solution composed of bovine serum albumin (BSA; Sigma-Aldrich, USA) 2%, FBS 2%, Triton X-100 0.01% in PBS for 30 min at 37 °C. Primary and secondary antibodies were diluted in blocking solution. Primary antibodies were incubated for 1 h at 37 °C, while incubation with secondary antibodies was done for 45 min at 37 °C. Nuclei were subsequently counterstained with DAPI (Roche, USA) 1:40,000 in PBS at RT for 5 min. Three rinses in PBS were performed between all steps except before incubation with the primary antibody. The glass coverslips were then dried and flattened on a glass slide using a DAKO mounting medium (Agilent, USA).

The primary antibodies used here were β-catenin 1:100 (ab32572, Abcam, UK), α-SMA 1:200 (A5228 clone1A1, Sigma-Aldrich, USA) and S100A4 1:100 (PA5-95736, Thermo Fisher Scientific, USA), while the secondary antibodies were Alexa Fluor 488 anti-rabbit, 1:2000, and Alexa Fluor 568 anti-mouse, 1:1,000 (Thermo Fisher Scientific, USA). Images were obtained with a fluorescent microscope (AXIO Imager.A1—Carl Zeiss).

### Growth factors and β-catenin Inhibitor/activator cell treatment

Rabbit CEnC (2.5 × 10^5^ cells) were seeded on an FNC-coated 6 well, 24 h prior to harvesting for the cytofluorimetric analysis. bFGF (Thermo Fisher Scientific, USA) and TGFβ (Miltenyi, Germany) were resuspended in MilliQ H_2_0 and then used at a final concentration of 20 ng/mL and 10 ng/mL, respectively. Cells were treated at different concentrations of Quercetin (Q4951, Sigma-Aldrich, USA) and CHIR 99,021 (SML1046, Sigma-Aldrich, USA). The treatments were performed at 24 h as both drugs were previously shown to elicit their effect at this time point^81,82^. CHIR99021 was tested at 50, 500 nM, 1, 3, 6 and 10 µM, Quercetin at 10 and 25 µM. Both compounds were dissolved in DMSO (Sigma-Aldrich, USA) and used 0.1% v/v in culture medium. Cytofluorimetric analysis of treated and untreated cells (DMSO as a vehicle control) was performed as described in the flow cytometry section. All the treatments described were performed 3 h after plating and the cells harvested 24 h after the treatment.

### Cell vitality assay

Viability of rCEnC upon CHIR99021 treatment was evaluated with calcein-AM (Thermo Fisher Scientific, USA), and propidium iodide (P3566_Invitrogen, Thermo Fisher Scientific, USA) staining. After 24 h from CHIR99021 treatment, rCEnC were incubated with 4 μM Calcein AM and 5 μg/ml propidium iodide for 30 min at 37 °C in 5% of CO_2_, then stained with DAPI 1:40,000, mounted on a glass slide and imaged with fluorescent microscope, as previously described. Cells were treated with 10 mM H_2_O_2_ for 2 h before vitality assay as a positive control for cell death.

### Ethical statement

Human corneas, non-suitable for transplantation, were obtained from Monza Eye Bank with written informed consent from donor’s next of kin. Experimental protocol was approved by ISS-CNT (Italian National Transplant Centre): a national health authority managing the national procedures and rules regarding all Italian transplants and delegating the Tissue Banks to collect the written informed consents. The research protocol on human corneal tissues was approved by the local ethical committee (Comitato Etico dell’Area Vasta Emilia Nord, p. 0002956/20). The tissues were handled in accordance with the declaration of Helsinki.

## Supplementary information

Supplementary Information.
