# Supplementary material for: A fine-tuned β-catenin regulation during proliferation of corneal endothelial cells revealed using proteomics analysis
Source: Sci Rep. 2020 Aug 14;10:13841. doi: 10.1038/s41598-020-70800-w (PMC7427785; doi:10.1038/s41598-020-70800-w)
Supplement: Supplementary file 1 — Supplementary Information. [file 41598_2020_70800_MOESM1_ESM.pdf]

A fine-tuned β-catenin regulation during proliferation of corneal endothelial cells revealed using proteomics analysis

Eleonora Maurizi[1,4], Davide Schiroli[2], Roberta Zini[1], Anna Limongelli[3], Raffaela Mistò[3], Claudio Macaluso[4], and Graziella Pellegrini[1]

Affiliations

1. Centre for Regenerative Medicine ''S. Ferrari'' Department of Life Sciences, University of Modena and Reggio Emilia, Modena, Italy
2. Department of Life Sciences, University of Modena and Reggio Emilia, Modena, Italy
3. Eye Bank, San Gerardo Hospital, Monza, Italy
4. Department of Medicine and Surgery, University of Parma, Italy

Supplementary Figure S1 Human cultured CEnC cell cycle phases was assessed at 60% confluence and different passages by cytofluorimeter following PI staining. Cultured CEnC morphology was observed by Axiovert 40C inverted microscope (Zeiss), objective 5x, at the passage prior to the cytofluorimetric assay. (A) Human CEnC at passage 2, showing a polygonal morphology, presents 20.8%±2.3% of its population in G2/M of the cell cycle. (B) Human CEnC at passage 4 are undergoing EnMT process and the percentage of cells in G2/M increases to 35±3.5%. Results are presented as mean ± Standard Error (SE), n=3.

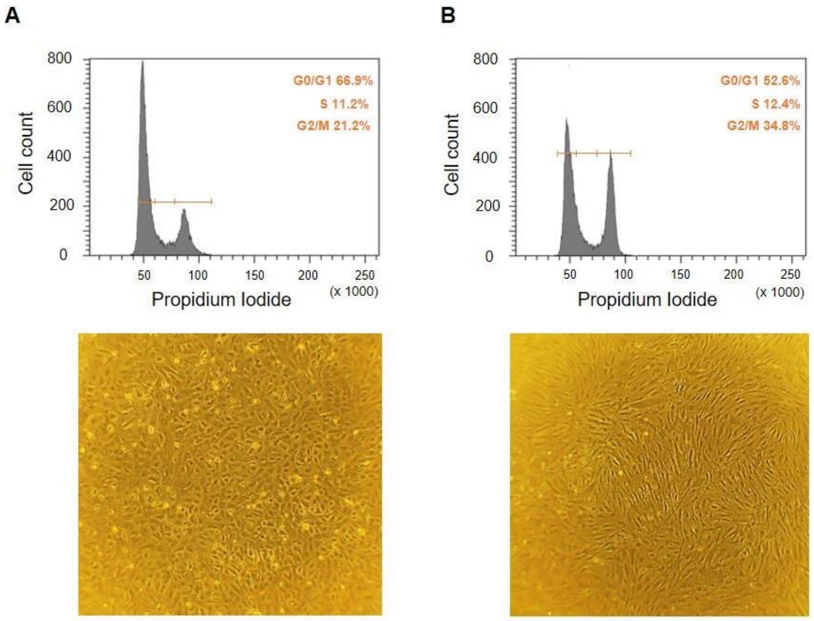

Supplementary Figure S2 Heat map showing the differentially expressed proteins between the cultured rCEnC (C1, C2, C3, each including a pool of five growing cultures obtained from left rabbit eyes at passage 1, 60% confluent) and the non-proliferating rCEnC isolated from the tissue (T1, T2, T3, each including a pool of cells isolated from the right five corresponding eyes). In (A), 19 proteins differentially expressed are represented with a p < 0.001; in (B), 77 proteins with a p < 0.005.

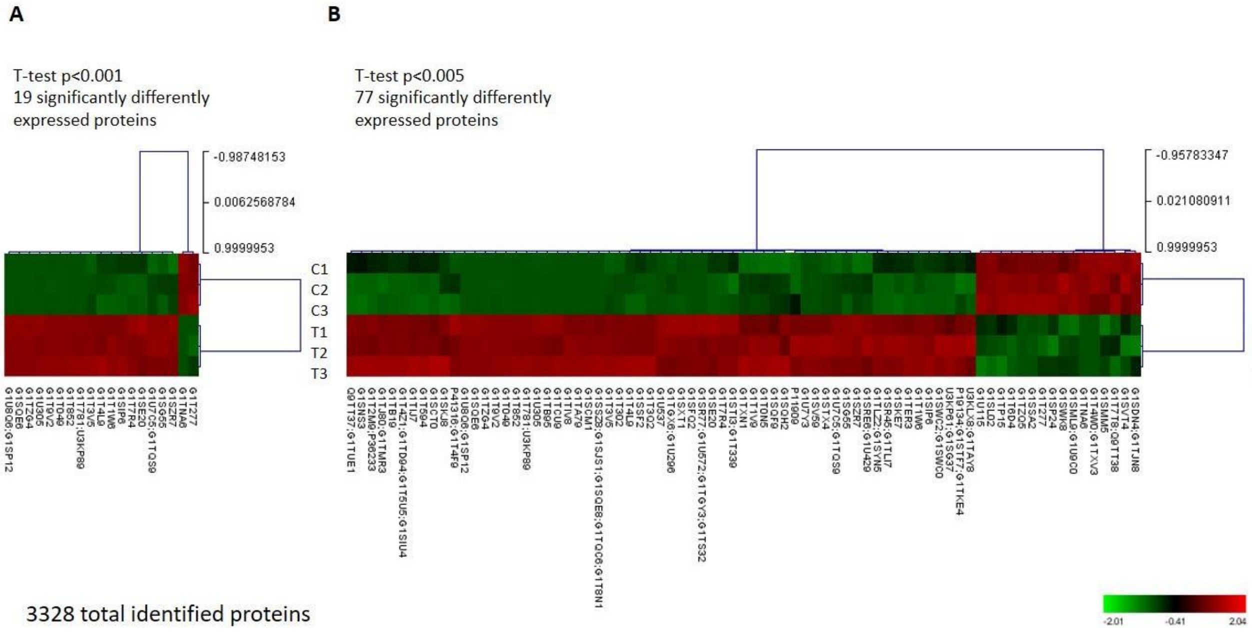

Supplementary Figure S3. Vitality assay upon CHIR99021 treatment on rCEnC. The panel shows representative immunofluorescence images of calcein AM (green, left column), propidium iodide (red, central column) and merge with DAPI in blue (right column). rCEnC were treated with H_2O_2 (top row) as a positive control and then with Mock DMSO control, CHIR99021 0.5μM and 10μM at the same conditions used for the experiment shown in Figure 5. Scale bar 50 μM, n=3 biological replicates for each condition.

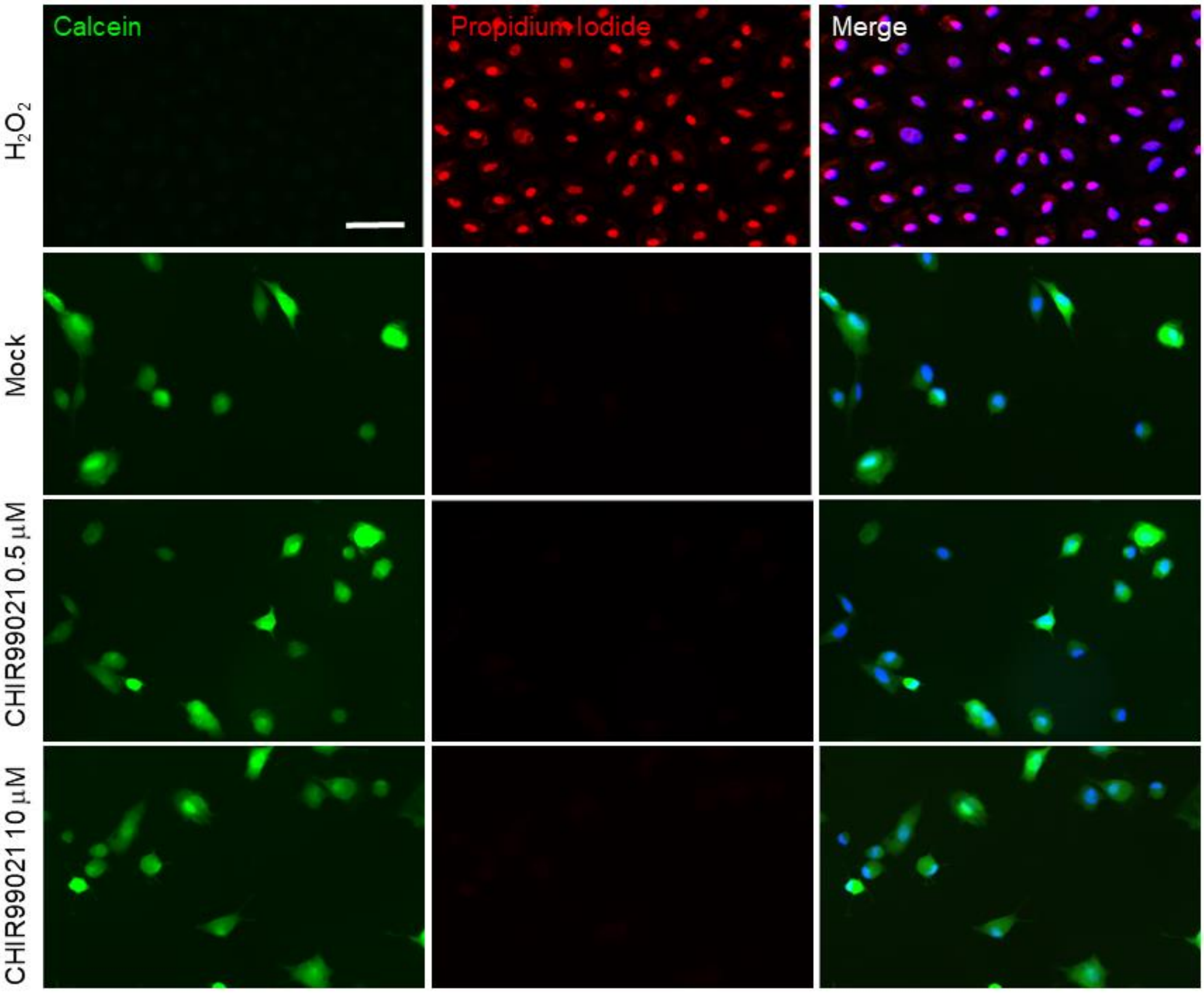

Supplementary Table S1 Full protein dataset including the 3328 proteins identified

Protein IDs	EM_C1	EM_C2	EM_C3	EM_T1	EM_T2	EM_T3
U3KP46	0	3,06E+08	0	0	0	0
G1SGH0;G1U414;G1U3L5	6,64E+09	9,8E+09	1,2E+10	6,11E+09	7,79E+09	1,2E+10
G1T1V0;G1SWB8;G1T4Q6	3,95E+10	3,96E+09	1,41E+10	1,31E+10	1,16E+10	1,07E+10
G1SDN2	0	0	0	0	2,23E+08	0
G1T8T1	0	0	0	0	0	59019000
G1SY72;G1TYI7;G1SHZ4	0	0	0	0	47592000	0
G1U9R4	0	29398000	0	0	0	0
P29338;A0A0A0MQQ6;G1STZ1	7,37E+08	3E+09	2,73E+09	2,76E+08	5,22E+08	5,07E+08
A0A0B4J1Q3;P01847;G1TKP3	0	0	0	2,36E+08	0	0
A0A0B4J1Q4;G1U2K3;G1TB60	4,43E+08	1,45E+09	1,76E+09	3,14E+08	3E+08	2,34E+08
P02103;P02099;G1TPE2	3,16E+08	1,1E+09	4,15E+08	2,72E+09	1,6E+10	1,53E+09
A0A140TAW0;Q6XLQ7	5,27E+10	1,63E+10	1,48E+10	1,89E+10	1,83E+10	2,74E+10
A2IBH5	0	0	0	5,72E+08	0	0
A7X8X3;G1T5F2	4,81E+08	4,19E+09	4,73E+09	3,11E+08	4,52E+08	8,89E+08
B6V9S9	1,77E+10	6,29E+10	6,73E+10	9,22E+09	9,19E+09	9,08E+09
B7NZD2	3,56E+09	9,02E+09	8,89E+09	3,32E+09	4,11E+09	4,11E+09
B7NZE9;G1T0U9;O77814	6,09E+08	0	0	7,88E+08	3,22E+09	6,18E+08
B7NZG7	7,68E+08	4,18E+09	4,11E+09	1,6E+09	2,27E+09	1,74E+09
B7NZI5	3,03E+09	5,51E+09	5,94E+09	4,23E+08	3,86E+08	5,47E+08
B7NZJ1	0	5,03E+08	3,71E+08	1,62E+08	2,15E+08	1,28E+08
B7NZJ4	4,62E+08	0	1,93E+08	2,67E+08	3,48E+08	2,46E+08
B7NZK2	1,74E+09	1,01E+09	7,53E+08	1,99E+09	2,26E+09	2,77E+09
B7NZM4	0	2,57E+08	3,6E+08	0	0	0
B7NZM8	3,08E+09	1,57E+10	1,57E+10	1,19E+09	1,23E+09	1,08E+09
G1TBW2;B7NZN8	7,6E+09	1,64E+09	1,68E+09	1,93E+09	1,98E+09	1,88E+09
B7NZN9	2,06E+10	5,84E+09	6,12E+09	1,76E+10	1,76E+10	1,68E+10
B7NZQ0	9,48E+08	7,25E+08	9,23E+08	2,83E+09	2,76E+09	3,87E+09
B7NZQ2;G1U2A1;G1T117	6,42E+09	3,4E+10	2,84E+10	8,54E+09	7,97E+09	7,32E+09
B7NZQ6	1,59E+09	6,55E+09	7,86E+09	1,09E+09	2,02E+09	1,2E+09
B7NZQ9	0	99639000	0	0	0	2,15E+08
C1IYH7	90632000	0	0	63524000	1,65E+08	1,11E+08
C6ZII8	0	0	0	0	53895000	0
G1SCE1	0	0	2,12E+08	0	0	0
G1SCE6	1,53E+08	2,63E+08	7,08E+08	1,56E+08	0	0
G1SCE7	2,57E+10	4,36E+09	3,98E+09	1,9E+10	1,31E+10	1,24E+10
G1SCF0;P13834	0	1,54E+08	2,41E+08	1,4E+08	0	1,4E+08
G1SCF4	3,77E+08	1,96E+09	1,52E+09	3,15E+08	2,96E+08	1,34E+08
G1SCF6	1,1E+09	2,36E+08	2,38E+08	2,66E+08	2,48E+08	2,74E+08
G1SCG8	2,77E+08	2,32E+08	0	4,03E+08	0	0
G1SCI0	1,71E+09	5,28E+09	8,34E+09	1,37E+09	1,11E+09	6,39E+08
G1SCI5	3,29E+09	1,14E+09	7,47E+08	6,11E+08	6E+08	5,33E+08
G1SCI9;G1TI92;G1U1U4	5,22E+08	4,95E+09	4,52E+09	5,41E+08	5,23E+08	1,02E+09
G1SCJ5	4,41E+09	1,6E+09	1,45E+09	4,71E+09	3,59E+09	4,1E+09
G1TRV3;G1SCJ6;G1TVT6	4,45E+09	1,85E+10	1,79E+10	5,37E+09	6,27E+09	5,43E+09
G1SCJ9	3,18E+08	0	0	8,22E+08	7,97E+08	8,67E+08
G1SCK0	2,88E+09	1,31E+09	9,42E+08	2,82E+09	2,07E+09	9,56E+08
G1SCK7	0	0	0	0	1,08E+08	0

G1SCL2	0	0	0	0	0	2,27E+08
G1SCM1	0	0	0	1,74E+09	1,8E+09	1,97E+09
G1SCM2;G1TMH6	1,46E+09	5,65E+08	4,53E+08	2,76E+09	1,76E+09	2,5E+09
G1SCM7;G1ST10	4,24E+08	1,02E+09	4,87E+08	3,09E+08	5,11E+08	4,34E+08
G1SCN1;G1TU10	0	0	0	3,23E+08	3,2E+08	0
G1SCN8	1,22E+10	2,86E+10	3,21E+10	5,76E+09	5,99E+09	5,41E+09
G1SCP0	0	2,77E+08	3,77E+08	1,61E+08	0	0
G1SCP7;G1TKE7;G1T2T2	4,97E+09	8,22E+09	8,31E+09	2,6E+09	2,34E+09	2,1E+09
G1SCP8	8,55E+09	2,8E+10	2,86E+10	2,35E+09	2,53E+09	1,67E+09
G1SCQ0	0	0	0	1,6E+08	0	0
G1SCQ1	4,67E+09	4,65E+09	5,5E+09	4,19E+09	3,54E+09	4,5E+09
G1SCQ6	5,72E+08	0	0	4,81E+08	0	0
G1SCR0	1,13E+09	5,83E+08	4,05E+08	2,9E+09	2,98E+09	3,17E+09
G1SCR1	0	1,11E+08	82587000	3,41E+08	1,27E+08	94621000
G1SCR7	2,83E+09	1,03E+09	1,29E+09	2,23E+09	1,31E+09	7,89E+08
G1SCS0	0	0	1,48E+08	2,98E+08	0	0
G1SCS8	1,75E+08	0	0	69620000	0	0
G1SCT0	1,31E+11	3,59E+10	3,35E+10	4,80E+11	5,22E+11	6,61E+11
G1SCT1	1,16E+09	3,63E+09	4,34E+09	8,38E+08	1,19E+09	1,07E+09
G1SCT9;G1TQ26	1,05E+09	3,53E+08	3,33E+08	2,54E+09	2,39E+09	1,26E+09
G1SCU8	0	3,61E+08	6,96E+08	2,37E+08	0	1,67E+08
G1SCW0	3,52E+09	9,09E+08	7,92E+08	1,42E+09	2,74E+09	3,42E+09
G1SCW7	4,33E+08	1,57E+09	1,73E+09	4,16E+08	5,14E+08	4,07E+08
G1SCX1;G1TA25;U3KMG2	0	0	0	0	0	8,94E+08
G1SCY3	0	4,31E+08	0	0	0	0
G1SCY4	5,64E+09	7,18E+09	8,55E+09	1,69E+09	2,64E+09	2,08E+09
G1SCY7	4,71E+09	8,73E+08	1,12E+09	4,04E+09	3,2E+09	1,78E+09
G1SCY8	1,19E+09	4,36E+08	5,12E+08	0	0	0
G1SCZ4;U3KN50	5,54E+08	4,8E+08	4,71E+08	3,03E+08	2,92E+08	3,11E+08
G1SCZ9	8,69E+08	3,54E+08	3,55E+08	7,08E+08	7,45E+08	5,37E+08
G1SD02	3,93E+08	2,1E+09	1,43E+09	8,99E+08	7,33E+08	6,69E+08
G1TC05;O46480;G1SD04	0	1,55E+08	0	0	0	0
G1SD05;G1TI13	9,94E+08	8,24E+09	5,1E+09	7,36E+08	5,44E+08	4,56E+08
G1SD09	3,5E+09	1,29E+09	1,33E+09	1,93E+09	1,75E+09	3,72E+09
G1SD25	1,02E+08	1,17E+08	0	66859000	0	0
G1SD27	0	2,51E+08	2,87E+08	2,49E+08	2,6E+08	3,55E+08
G1SD30	0	0	0	0	1,68E+08	0
G1SD44	1,56E+09	3,61E+08	3,47E+08	1,1E+09	9,8E+08	1,06E+09
G1SD48	2,69E+09	6,97E+08	5,25E+08	1,97E+09	2,12E+09	3,3E+09
G1SD49	0	5,18E+08	7,08E+08	0	1,16E+08	96482000
G1SD50	2,19E+09	6,2E+08	7,31E+08	2,44E+09	1,45E+09	1,09E+09
G1SD54	6,06E+08	2,05E+08	1,53E+08	1,69E+08	0	0
G1SD77	1,99E+08	4,08E+08	2,62E+08	3,5E+08	4,09E+08	5,2E+08
G1SD83	1,08E+10	2,92E+09	3,54E+09	1,81E+08	39539000	1E+08
G1SD87;G1TLX0;G1TN12	62090000	0	0	0	0	0
G1SD91	0	5,15E+08	6,22E+08	0	0	0
G1SD92	0	0	0	1,89E+09	1,62E+09	9,72E+08
G1U5X9;G1TXJ9;G1SD95	98311000	5,99E+08	4,49E+08	9,63E+08	1,4E+09	1,61E+09
G1U727;G1SD98	2,27E+08	0	0	1E+08	0	0
G1SD99	0	0	0	1,2E+09	9,27E+08	9,07E+08

G1SDA4	1,85E+09	5,89E+09	5,33E+09	1,43E+09	9,83E+08	5,29E+08
G1SDA8	4,01E+09	6,77E+09	6,88E+09	2,17E+09	2,16E+09	1,67E+09
G1SDA9	2,4E+08	0	1,07E+08	4,03E+08	1,17E+08	1,54E+08
G1SDB3	6,15E+08	2,78E+08	2,42E+08	2,94E+08	3,48E+08	3,47E+08
G1SDC7	3,67E+08	1,62E+09	2,2E+09	7,19E+08	6,11E+08	1,06E+09
G1SDD0	2,57E+10	8,73E+09	7,94E+09	2,12E+10	2,41E+10	3,71E+10
G1SDD7	1,03E+08	0	0	0	0	0
G1SDE1	3,08E+08	0	0	2,76E+08	0	0
G1SDF2	9,73E+08	2,97E+09	3,31E+09	2,19E+09	2,58E+09	2,47E+09
G1SDG2	3,56E+08	1,29E+09	1,07E+09	0	2,52E+08	0
G1SDG8	5,53E+08	7,21E+08	8,12E+08	2,99E+08	1,66E+08	2,07E+08
G1SDH2	7,21E+08	1,75E+08	1,91E+08	0	0	1,44E+08
G1SDH3	1,56E+09	1,9E+08	1,78E+08	1,48E+08	1,54E+08	1,85E+08
G1SDH5	0	1,11E+09	9,69E+08	2,04E+08	1,65E+08	3,44E+08
G1SDJ0;G1U695;G1TN15	2E+10	1,32E+10	1,53E+10	2,31E+10	2,74E+10	2,49E+10
G1SDJ3	4,2E+09	1,07E+09	1,06E+09	5,9E+09	5,29E+09	5,26E+09
G1SDJ7	9,07E+08	1,85E+09	2,18E+09	8,87E+08	7,91E+08	8,08E+08
G1SDK0	0	0	1,13E+08	0	0	0
G1SDK5	0	0	0	0	1,41E+08	0
G1SDK8	0	1,19E+09	1,73E+09	5,33E+08	1,94E+08	2,33E+08
G1SDK9	1,3E+08	0	0	0	0	0
G1TWY4;G1SDL3	8,11E+08	5,64E+08	3,51E+08	1,17E+09	7,38E+08	6,88E+08
G1SDL7;Q4VK78	5,68E+08	0	0	0	2,12E+08	3,73E+08
G1SDL9	6,08E+08	3,36E+08	1,94E+08	1,81E+09	9,22E+08	1,49E+09
G1SDM0;G1T9R6	1,24E+08	2,68E+08	1,77E+08	0	98697000	0
G1SDM2	3,31E+09	1,07E+09	8,73E+08	1,82E+09	1,34E+09	7,83E+08
G1SDM6	54750000	63716000	67989000	90361000	0	0
G1SDN1;G1SW87	9E+08	1,38E+09	1,53E+09	1,84E+09	1,62E+09	1,46E+09
G1SDN3	5,79E+08	5,94E+09	6,1E+09	5,45E+08	5,34E+08	6,52E+08
G1SDN4;G1TJN8	1,45E+10	1,39E+10	1,39E+10	1,01E+10	9,4E+09	1,07E+10
G1SDN8	1,06E+09	4,44E+08	2,37E+08	5,64E+09	4,32E+09	6,02E+09
G1SDN9	3,88E+08	0	1,89E+08	0	3,63E+08	5,76E+08
G1SDP2	2,57E+10	1,15E+10	1,06E+10	2,7E+10	2,74E+10	3,96E+10
G1SDR2	3,45E+08	2,01E+09	1,58E+09	8,82E+08	1E+09	1,4E+09
G1SDS3	0	1,32E+08	0	0	0	0
G1SDT0	2,82E+08	0	0	0	0	0
G1SDU1	1,29E+08	4,86E+08	2,98E+08	3,19E+08	5,17E+08	7,55E+08
G1SDU2	0	0	2,3E+08	0	0	0
G1SDU5	8,53E+08	2,72E+09	3,07E+09	2,17E+09	1,44E+09	1,36E+09
G1SDU6	3,08E+08	8,29E+08	6,8E+08	2,42E+08	2,6E+08	2,51E+08
G1SDU8	1,31E+09	0	1,03E+08	3,2E+08	3,16E+08	3,49E+08
G1TGU3;G1SDV0	2,22E+08	0	0	0	0	0
G1SDV3	3,47E+08	7,78E+08	5,18E+08	4,94E+08	4,17E+08	4,73E+08
G1SDV5	1,28E+08	0	0	0	0	0
G1SDW3	5,26E+08	0	0	1,1E+09	9,08E+08	7,24E+08
G1SDW8	2,89E+09	8,39E+08	1,12E+09	0	3,11E+08	1,87E+08
G1SDX2	1,39E+09	1,72E+08	2,19E+08	2,31E+08	2,19E+08	2,01E+08
G1SDX3	2,35E+09	1,86E+08	1,05E+08	1,97E+08	1,71E+08	94463000
G1SDX5	7,4E+08	2,32E+08	1,71E+08	1,36E+09	8,85E+08	5,04E+08
G1SDX9	1,72E+08	0	0	2,59E+08	3,92E+08	1,8E+08

G1SDY5	3,1E+10	1,10E+11	1,19E+11	1,68E+10	2,64E+10	2,35E+10
G1SDZ0	5,62E+09	2,37E+09	1,96E+09	2,47E+09	3,18E+09	3,17E+09
G1SDZ7	0	1,92E+08	1,9E+08	0	0	0
G1SDZ9	3,06E+08	0	0	0	0	0
G1SE10	9,21E+08	2,98E+08	3,17E+08	3,55E+08	3,09E+08	2,58E+08
G1SE12	6,05E+10	1,73E+10	1,74E+10	3,01E+10	3,45E+10	4,62E+10
G1SE20	3,68E+08	2,24E+08	2,32E+08	1,67E+09	1,48E+09	1,39E+09
G1SE27	3,46E+09	9,78E+08	7,99E+08	4,84E+09	6,47E+09	7,43E+09
G1SE28;G1TLD5;G1TH55	2,79E+09	1,49E+10	1,31E+10	4,26E+09	4,45E+09	3,41E+09
G1SE36;P56201	1,52E+09	6,37E+08	4,53E+08	7,78E+08	4,9E+08	5,47E+08
G1SE37;U3KN93	1,92E+09	3,51E+08	3,31E+08	3,39E+08	4,74E+08	4,79E+08
G1SE39	0	2,78E+08	3,32E+08	2,56E+08	3,8E+08	4,21E+08
G1SE41	0	0	0	4,3E+08	4,22E+08	6,99E+08
G1SE46	8,33E+08	8,25E+08	5,74E+08	4,06E+09	2,97E+09	3,65E+09
G1SE50	3,22E+08	1,24E+09	6,71E+08	0	5,41E+08	3,95E+08
G1SE51	5,79E+08	5,12E+08	6,8E+08	5,4E+08	5,78E+08	6,81E+08
G1SE56	8,45E+08	4,24E+08	5,06E+08	4,97E+08	8,35E+08	4,85E+08
G1SE57	8,07E+09	1,05E+10	1,01E+10	8,72E+09	6,91E+09	5,66E+09
G1SE61;Q9MZD2	4,85E+09	1,19E+10	1,35E+10	1,17E+10	9,55E+09	7,31E+09
G1SE63	0	0	0	2,28E+08	0	0
G1SE67	0	3,24E+08	3,7E+08	0	1,96E+08	2,77E+08
G1TRP8;G1SE76;G1TF04	5,03E+09	1,93E+10	1,82E+10	7,06E+09	6,29E+09	6,93E+09
G1SE78	44176000	0	0	0	0	0
G1SE87	4,83E+08	3,68E+08	3,97E+08	1,12E+10	6,36E+09	5,22E+09
G1SE88	3,81E+09	9,59E+08	9,13E+08	1,26E+09	1,51E+09	1,61E+09
G1SE95	3,21E+10	7,94E+10	9,32E+10	1,82E+10	1,28E+10	1,13E+10
G1SE97	0	0	0	0	3,17E+08	0
G1SEA8	0	1,33E+08	1,68E+08	0	0	0
G1SEC8	2,56E+09	3,14E+08	2,78E+08	2,75E+08	3,13E+08	1,15E+08
G1SEC9	8,72E+09	3,15E+09	2,46E+09	4,96E+09	6,59E+09	6,59E+09
G1SED7	0	0	0	0	1,37E+08	0
G1SED9	2,52E+08	4,07E+09	3,62E+09	44318000	47512000	0
G1SEE0	1,94E+09	4,51E+09	4,07E+09	3,99E+09	4,98E+09	7,36E+09
G1SEF1	7,14E+09	1,16E+09	1,04E+09	5,09E+09	4,71E+09	4,95E+09
G1SEF8	1,05E+10	4,81E+09	4,37E+09	7,26E+09	8,54E+09	1,05E+10
G1SEH7	4,82E+09	7,12E+08	5,85E+08	3,79E+09	3,95E+09	5,78E+09
G1SEI0	1,7E+09	9,53E+08	9,2E+08	2,06E+09	2,06E+09	2,17E+09
G1SEI8	4,79E+08	1,36E+08	1,07E+08	2,26E+08	2,52E+08	4,02E+08
G1SEJ4;G1TQ38	1,05E+10	7,57E+09	7,5E+09	6,74E+09	8,64E+09	7,25E+09
G1SEJ5	0	0	0	0	4,84E+08	3,14E+08
G1SEJ8	46650000	0	0	0	0	0
P62143;G1SEK1	5,73E+08	3,06E+09	3,02E+09	1,78E+09	1,82E+09	1,5E+09
G1SEK2	0	2,56E+08	1,58E+08	90462000	0	0
G1SEL8	5,84E+09	3,04E+09	2,87E+09	1,13E+10	1,43E+10	1,22E+10
G1SEM0	8,38E+08	2E+09	1,67E+09	3,77E+08	6,38E+08	0
G1SEN8	3,67E+09	7,74E+08	8,77E+08	1,04E+10	6,56E+09	4,76E+09
G1SEQ2	2,24E+08	1,36E+09	1,55E+09	3,58E+08	4,99E+08	4,71E+08
G1SEQ7	0	2,58E+08	1,35E+08	0	1,08E+08	0
G1SER3	6,68E+09	1,73E+09	1,83E+09	7,96E+09	6,13E+09	4,39E+09
G1SER8	9,14E+09	4,98E+10	4,66E+10	3,35E+09	5,28E+09	3,88E+09

G1SER9	0	2E+08	2,18E+08	0	0	0
G1SES2;G1SYK9	4,56E+08	4,84E+09	3,95E+09	3,94E+08	3,17E+08	3,65E+08
G1SES4;G1T5L8	4,84E+08	1,52E+09	1,45E+09	2,2E+09	2,23E+09	2,27E+09
G1SES5	93449000	1,3E+08	95580000	0	0	0
G1SES8	7,96E+09	2,3E+09	2,14E+09	1,89E+09	2,23E+09	2,14E+09
G1SES9;U3KMZ3	4,81E+09	1,81E+10	1,87E+10	4,23E+09	5,08E+09	3,69E+09
G1SET0	3,91E+09	7,56E+09	9,6E+09	4,16E+09	3,25E+09	2,47E+09
G1SEU9	9,66E+08	4,65E+08	0	7,8E+08	9,76E+08	9,46E+08
G1SEV2;G1TTY0	2,67E+11	1,52E+11	1,32E+11	4,09E+11	4,39E+11	6,37E+11
G1SEV7	1,23E+08	1,78E+09	3,37E+09	35823000	41305000	90615000
G1SEW2	3,08E+08	1,5E+08	1,72E+08	2,14E+08	2,09E+08	2,98E+08
G1SEW3;G1TZC2	2,47E+09	5,62E+08	7,07E+08	3,62E+08	3,02E+08	1,68E+08
G1SEW5	5,5E+08	2,74E+08	2,63E+08	2,79E+08	1,85E+08	2,46E+08
G1SEX0	1,69E+08	0	0	1,13E+09	4,5E+08	1,9E+08
G1SEX5	0	4,98E+08	1,82E+08	94170000	1,18E+08	1,45E+08
G1SEZ0;G1T6Y3;G1SF90	4,84E+08	2,82E+08	2,21E+08	3,17E+08	3,05E+08	3,35E+08
G1SF08;G1U7Y8;G1U062	2,14E+09	1,06E+10	7,71E+09	1,77E+09	1,79E+09	1,57E+09
G1SF32	1,24E+10	3,74E+09	4,18E+09	1,85E+10	1,72E+10	2,43E+10
G1SF35	79424000	0	0	0	0	0
G1SF36	2,07E+10	6,03E+09	5,72E+09	1,96E+10	1,45E+10	9,52E+09
G1SF38	0	2,49E+08	0	0	0	2,33E+08
G1SF45	2,34E+08	0	0	0	0	0
G1SF47;G1SKQ9	1,03E+09	5,8E+09	6,46E+09	1,25E+09	1,55E+09	1,41E+09
G1SF51	0	0	0	0	0	6,32E+08
G1SF58	1,02E+08	0	0	0	0	0
G1SF64	2,08E+08	0	0	0	0	0
G1SF70	0	0	0	0	0	2,03E+08
G1SF78	4,53E+08	0	0	0	0	0
G1SF82	4,85E+09	1,35E+09	1,31E+09	4,77E+09	6,99E+09	1,09E+10
G1SF95	1,33E+10	1,12E+10	9,76E+09	2,82E+10	3,18E+10	3,88E+10
G1SF97	2,44E+09	8,6E+08	1,08E+09	9,2E+08	1,11E+09	9,73E+08
G1SFA0;G1SR02	0	1,03E+08	1E+08	0	0	0
G1SFB2	1,54E+09	3,54E+08	3,59E+08	6,62E+08	5,95E+08	5,19E+08
U3KPD0;G1SFC1	1,15E+09	8,61E+08	6,08E+08	1,39E+09	1,67E+09	1,76E+09
G1THY6;G1SFC4	5,55E+08	1,24E+08	1,73E+08	1,04E+09	4,65E+08	7,09E+08
G1SFC5;G1SFC8	9,56E+08	2,93E+09	3,11E+09	1,01E+09	1,39E+09	1,47E+09
G1SFC6	9,22E+08	2,33E+08	1,98E+08	2,74E+08	2,94E+08	4,68E+08
G1SFD5	0	0	0	0	0	2,6E+08
G1SFD8;U3KNQ7	1,67E+09	6,18E+08	7,4E+08	2,52E+09	2,05E+09	2,02E+09
G1SFE0	3,62E+09	5,92E+09	6,95E+09	1,45E+09	1,37E+09	2,09E+09
G1SFE6	43034000	76321000	0	57967000	0	64291000
G1SFE9	8,16E+08	1,37E+09	1,89E+09	2,6E+08	2,78E+08	5,53E+08
G1SFF2	3,48E+09	8,28E+08	7,83E+08	1,14E+09	9,24E+08	7,56E+08
G1SFF5	2,53E+08	0	0	0	0	0
G1SFF7	3,25E+09	1,25E+09	1,12E+09	2,39E+10	1,85E+10	1,45E+10
Q9N1E2;G1SFG6	3,39E+09	1,1E+10	1,22E+10	3,92E+09	5,04E+09	2,66E+09
G1SFG7	2E+10	1,89E+09	4,64E+09	4,47E+08	0	1,75E+08
G1SFH4	3,44E+09	9,03E+08	1,08E+09	1,54E+09	2,31E+09	2,47E+09
G1SFH5	3,26E+10	1,53E+10	1,35E+10	5,22E+10	4,19E+10	5,49E+10
G1SFH6	7,78E+08	3,37E+09	3,32E+09	1,04E+09	1,15E+09	1,23E+09

G1SFH8	1,52E+08	3,36E+08	2,45E+08	0	0	0
G1SFH9	8,46E+09	3,23E+09	3,68E+09	1,99E+10	1,52E+10	1,31E+10
G1SFI4	1,21E+08	78442000	0	2,83E+08	1,35E+08	1,05E+08
G1SFI7	1,3E+09	7,5E+09	5,52E+09	1,74E+09	1,55E+09	2,19E+09
G1SFJ8	0	1,27E+09	9,89E+08	8,21E+08	1,24E+09	1,26E+09
G1SFJ9	1,62E+08	6,36E+08	5,46E+08	0	0	0
G1SFK3	6,87E+08	1,83E+09	2,25E+09	4,72E+08	5,01E+08	4,19E+08
G1SFL5	2,46E+08	5,82E+08	1,28E+09	0	3,25E+08	2,51E+08
G1SFL7	6,17E+08	2,7E+09	2,09E+09	3,09E+09	3,56E+09	2,57E+09
G1SFN1	6,03E+08	0	1,75E+08	2,11E+08	1,67E+08	2,13E+08
Q28653;G1SFN5	0	2,19E+08	4,51E+08	0	0	0
G1SFP0	2,86E+09	3,3E+09	2,81E+09	2,03E+09	2,44E+09	1,56E+09
G1SFQ1;U3KNE1	9,49E+08	1,18E+09	1,27E+09	9,4E+08	8,12E+08	9,33E+08
G1SFQ2	0	0	0	69282000	62258000	60453000
G1SFQ3	1,3E+09	4,21E+08	4,9E+08	7,68E+08	8,43E+08	7,09E+08
G1SFQ9	0	0	0	0	6526800	0
G1SFR8	1,26E+10	5,19E+10	6,49E+10	7,23E+09	1,14E+10	1,21E+10
G1SFS8	4,08E+09	1,39E+10	1,63E+10	6,79E+09	5,78E+09	5,48E+09
G1SFT2	1,82E+08	8,02E+08	5,46E+08	6,33E+08	1,2E+09	1,22E+09
G1SFU0;O97572;U3KLX2	2,35E+10	5,99E+09	7,24E+09	1,16E+10	9,97E+09	7,13E+09
G1SFU4	2,4E+09	1,05E+09	8,67E+08	3,94E+09	3,42E+09	3,97E+09
G1SFV1	6,7E+10	3,37E+10	2,92E+10	5,74E+10	5,92E+10	7,8E+10
G1SFV7	8,84E+09	1,89E+10	2,1E+10	3,98E+10	3,32E+10	3,37E+10
G1SFV8;G1U6M2	0	0	3,32E+08	2,08E+09	1,57E+09	5,84E+08
G1SFW9	2,25E+09	7,08E+08	7,56E+08	8,46E+08	1,3E+09	1,21E+09
G1SFX0	1,02E+09	1,14E+08	1,22E+08	60468000	2,29E+08	2,33E+08
G1SFX7	6,92E+08	1,46E+09	1,47E+09	0	0	0
G1SFZ1;G1SWZ9	1,05E+09	3,54E+09	5,7E+09	7,34E+08	8,26E+08	7,12E+08
G1SFZ2	1,86E+09	2,66E+08	3,04E+08	8,08E+08	6,05E+08	3,97E+08
G1SFZ8;B8K197	1,58E+09	3,52E+09	4,08E+09	1,14E+09	1,58E+09	1,43E+09
G1SG06	7,39E+09	2,36E+09	1,88E+09	8,29E+09	8,28E+09	9,94E+09
G1SG11	3,7E+10	4,34E+09	4,5E+09	3,41E+10	3,1E+10	4,43E+10
G1SG14	0	0	0	0	1,53E+08	0
G1SG16	0	0	1,9E+08	0	0	0
G1SG29	1,47E+09	6,65E+09	8,88E+09	2,63E+09	3,13E+09	3,17E+09
G1TQC3;G1SG30	1,42E+09	1,97E+08	1,85E+08	2,29E+08	2,28E+08	2,04E+08
G1SG31	2,52E+09	9,44E+08	7,96E+08	2,81E+09	2,81E+09	2,34E+09
G1SG35	0	0	4,54E+08	0	0	0
U3KP61;G1SG37	4,3E+08	2,27E+08	2,17E+08	1,11E+09	1,48E+09	1,41E+09
G1SG41	1,76E+09	8,07E+08	7,28E+08	3,33E+09	2,15E+09	1,18E+09
G1SG42;G1THG4	2,64E+08	5,99E+08	7,58E+08	1,43E+09	1,57E+09	2,08E+09
G1SG44	2,48E+09	5,07E+08	6,34E+08	2,53E+09	1,28E+09	1,75E+09
G1SG47	77754000	0	0	0	0	0
G1SG55	9,82E+09	1,07E+10	9,7E+09	1,95E+10	1,99E+10	1,89E+10
G1SUR2;G1SG59	0	1,95E+08	1,54E+08	0	0	0
G1SG61	0	0	0	0	0	0
G1SG63	2,65E+09	0	1,26E+09	7,38E+08	1,39E+09	6,44E+08
G1SG68	3,86E+08	8,12E+08	9,62E+08	1,57E+08	3,04E+08	3,5E+08
G1SG72	5,62E+08	2,21E+09	3,44E+09	7,16E+08	3,71E+08	3,66E+08
G1SG80	2,29E+10	1,16E+10	1,08E+10	1,64E+10	2,06E+10	2,59E+10

G1SGA5	1,78E+09	5,1E+09	4,27E+09	5,79E+08	7,93E+08	6,14E+08
G1SGB5	3,08E+10	1,61E+10	1,69E+10	5,23E+10	6,21E+10	6,36E+10
G1SGB7;U3KND8	8,82E+08	3,17E+08	4,91E+08	3,98E+08	0	0
G1SGC2;G1SUJ3;O62695	1,08E+09	8,11E+08	7,83E+08	9,85E+08	1,18E+09	1,08E+09
G1SGC3	1,55E+08	1,04E+08	1,31E+08	0	0	0
G1SGD7	0	0	0	2,36E+08	1,49E+08	0
G1SGE5	1,34E+09	5,66E+08	4,69E+08	1,13E+09	9,21E+08	9,28E+08
G1SGF7	0	0	0	1,16E+08	69138000	1,2E+08
G1SGG2	1,5E+08	2,17E+08	1,85E+08	0	0	1,2E+08
G1SGG6	7,76E+09	2,35E+09	2,09E+09	5,76E+09	4,89E+09	3,21E+09
G1SGH2	3,3E+09	7,3E+08	9,1E+08	2,17E+09	1,79E+09	1,49E+09
G1SGH3	1,56E+08	1,58E+08	1,38E+08	58544000	0	0
G1SGI3;U3KPQ1	4,14E+08	0	0	0	0	0
G1SGI8	8,02E+09	3,97E+09	3,87E+09	6,29E+09	6,51E+09	1,02E+10
G1SGJ7	0	0	0	0	0	87060000
G1SGK1	0	3,01E+08	2,73E+08	0	0	3,48E+08
G1SGK6	3,31E+08	0	0	1,46E+08	2,23E+08	1,29E+08
G1SGL0	3,64E+08	0	2,66E+08	7,43E+08	7,59E+08	5,8E+08
G1U830;G1U6I8;G1SGL3	1,9E+08	1,78E+09	1,82E+09	3,3E+09	1,28E+09	4,46E+09
G1SGL4	9,09E+08	1,84E+08	0	2,43E+08	1,69E+08	0
G1SGM2	3,28E+08	3,38E+08	6,87E+08	5,1E+08	3,74E+08	5E+08
G1SGM3	9,69E+09	2,34E+09	2,67E+09	3,14E+10	2,09E+10	3,07E+10
G1SGN5	5,99E+08	4,33E+08	4,38E+08	1,01E+09	8,02E+08	8,48E+08
G1SGN6	5,51E+08	5,2E+08	4,38E+08	8,65E+08	1,4E+09	1,42E+09
G1SGP1	9,8E+10	2,01E+10	1,81E+10	4,6E+10	5,14E+10	7,14E+10
G1SGP4	0	0	0	1,19E+08	0	0
G1SGQ0	8,68E+09	2,01E+09	1,95E+09	5,3E+09	5,32E+09	6,94E+09
G1SGQ1	76257000	0	0	0	0	0
G1SGQ6	1,54E+08	3,68E+08	1,86E+08	0	0	0
G1SGR8;G1U1E9	1,4E+08	3,6E+08	4,77E+08	2,36E+08	2,6E+08	2,93E+08
G1SGR9;G1TWZ7	4,62E+08	6,56E+08	1,09E+09	2,13E+09	2,38E+09	2,08E+09
G1SGS2	2,81E+08	1,88E+08	4,26E+08	1,15E+10	4,69E+09	3,52E+09
G1SGS7	1,6E+09	2,51E+09	1,87E+09	9,13E+09	8,25E+09	7,16E+09
G1SGV5	6,77E+08	3,95E+09	5,46E+09	1,05E+09	5,89E+08	4,8E+08
G1SGX2	1,73E+09	6,71E+08	4E+08	4,6E+08	6,37E+08	4,18E+08
G1SGX4	2,83E+09	8,21E+09	9,03E+09	3,23E+09	2,59E+09	1,98E+09
G1SGX5	4,39E+08	0	0	1,92E+08	2,32E+08	2,44E+08
G1SGY0	5,26E+08	6,96E+08	5,98E+08	4,51E+08	3,82E+08	3,63E+08
G1SGY8	1,89E+10	3,82E+09	5E+09	6,81E+09	8,19E+09	6,36E+09
G1SGZ2	32366000	1,64E+08	0	18360000	19733000	18609000
P00489;G1SGZ6;U3KMU3	0	1,63E+08	1,02E+08	97005000	1,09E+08	0
G1SGZ7	8,06E+08	3,52E+08	1,96E+08	1,4E+08	0	1,69E+08
G1SH00	5,61E+09	1,82E+09	1,73E+09	1,46E+09	1,46E+09	1,65E+09
G1SH05	6,71E+10	3,66E+11	3,46E+11	7,39E+10	8,4E+10	1,02E+11
G1SH08	0	0	0	2,95E+08	2,1E+08	1,89E+08
G1SH10	3,79E+09	1,48E+09	1,46E+09	2,45E+09	1,37E+09	1,22E+09
G1SH18	0	2,12E+08	0	0	0	0
G1SH25	1,12E+09	5,81E+08	7,74E+08	5,01E+08	6,4E+08	3,38E+08
G1SH26	1,53E+09	4,06E+09	3,48E+09	1,12E+09	1,88E+09	1,5E+09
G1SH27	1,16E+08	0	0	0	0	0

G1SH44	0	0	0	8,24E+08	8,89E+08	1,09E+09
G1SH63	2,71E+08	1,77E+09	1,67E+09	4,16E+08	5,98E+08	3,78E+08
G1SH66	3,52E+09	2,05E+10	2,64E+10	3,96E+09	5,66E+09	7,35E+09
G1SH73	0	0	0	0	0	1,61E+08
G1SH78	3,94E+09	7,19E+08	5,83E+08	4,19E+09	4,87E+09	3,54E+09
G1SH80	2,21E+09	4,71E+08	3,02E+08	9,15E+08	5,86E+08	8,38E+08
G1SH81	2,86E+09	4,88E+09	5,93E+09	2,18E+08	3,36E+08	3,41E+08
G1SH86	1,43E+08	9,22E+08	4,27E+08	0	0	1,27E+08
G1SH88	2,8E+08	4,47E+09	4,12E+09	1,57E+08	2,13E+08	2,15E+08
G1SH95	6,81E+08	1,08E+09	1,07E+09	0	0	0
G1SHB9;G1SS57	7,48E+08	5,49E+09	5,62E+09	9,12E+08	1E+09	1,34E+09
G1SHC5;G1TPV2	1,15E+09	3,41E+08	3,46E+08	1,29E+09	8,41E+08	1,98E+09
O02818-2;O02818;G1SHC9	0	0	0	1,56E+08	0	0
G1SHD6;G1TC46	7,5E+08	3,58E+09	3,69E+09	7,27E+08	7,5E+08	6,22E+08
G1SHE2	1,13E+09	9,45E+08	0	3,75E+09	2,43E+09	1,83E+09
G1SHE6	1,19E+09	4,02E+08	4,28E+08	2,62E+09	3,83E+09	3,24E+09
G1SHF0	0	54479000	1,21E+08	0	0	0
G1SHF1	6,24E+08	4,67E+09	5,36E+09	1,19E+09	1,39E+09	1,06E+09
G1SHF3	8,64E+08	1,4E+09	1,2E+09	8,33E+08	8,95E+08	7,75E+08
G1SHF6	0	0	0	1,78E+08	0	0
G1SHF7	1,4E+08	2,8E+08	1,91E+08	1,28E+08	1,08E+08	0
G1SHF9	1,62E+08	1,14E+09	7,41E+08	3,34E+08	2,32E+08	2,11E+08
G1SHG0;G1TS06;G1TM17	3,66E+09	1,5E+10	1,56E+10	5,35E+09	6,03E+09	6,54E+09
G1SHG5	50033000	0	0	0	0	0
G1SHI0	2,72E+09	7,31E+08	5,76E+08	2,15E+09	2,19E+09	1,97E+09
G1SHI3	1,17E+08	2,69E+08	3,02E+08	1,47E+08	85882000	1,61E+08
G1SHI9	4,27E+10	1,15E+10	1,19E+10	1,81E+10	1,48E+10	1,5E+10
G1SHJ3	2,33E+08	0	0	1,31E+08	75254000	1,11E+08
G1SHK6	9,2E+09	5,08E+09	4,47E+09	3,06E+10	2,31E+10	2,35E+10
G1SHK7	2,14E+09	1,13E+09	1,18E+09	3,55E+09	4,75E+09	5,24E+09
G1SHK8;G1TBD6	7,09E+10	2,01E+10	1,59E+10	5,95E+10	5,71E+10	6,87E+10
G1SHL0	1,09E+09	7,17E+08	1,03E+09	1E+09	1,24E+09	1,32E+09
G1SHL2	3,33E+08	2,73E+08	3,75E+08	1,13E+09	1,44E+09	2,05E+09
U3KN03;G1SHL8	0	3,92E+08	3,13E+08	52503000	70725000	1,07E+08
G1SHL9	5,1E+09	1,62E+09	1,4E+09	3,7E+09	3,8E+09	4,38E+09
G1SHM1;G1TNF5;G1TTY1	1,84E+09	4,09E+09	4,46E+09	2,59E+09	3,81E+09	3,92E+09
G1SHM2	5,74E+08	2E+08	1,72E+08	4,08E+08	2,4E+08	1,7E+08
G1SHQ1	0	0	0	1,36E+08	0	0
G1SHQ2;G1U567;G1TJF3	4,76E+09	2,7E+10	2,54E+10	7,69E+09	8,37E+09	6,85E+09
U3KLZ8;G1SHR5	0	0	0	2,23E+08	0	0
O97554;G1SHR6	2,43E+08	92479000	0	0	0	0
G1SHS0	60067000	0	0	0	0	0
G1SHS7	1,26E+10	5,7E+10	6,1E+10	8,28E+09	9,67E+09	7,83E+09
G1SHS8	0	5,61E+08	5,96E+08	0	1,76E+08	1,25E+08
G1SHS9	2E+08	0	0	0	1,41E+08	2,1E+08
G1SHT2;G1TS13	1,86E+10	5,18E+09	4,02E+09	1,24E+10	1,55E+10	1,47E+10
G1SHT5	2,42E+08	1,71E+08	0	0	0	0
G1SHU3	0	1,91E+08	0	2,39E+08	5,65E+08	2,29E+08
G1SHU5	1,77E+08	7,3E+08	8,39E+08	0	0	0
G1U437;G1SHU8;G1TUN8	5,79E+09	2,24E+10	2,06E+10	7,12E+09	6,62E+09	5,71E+09

G1SHV1	2,71E+08	3,67E+08	3,6E+08	2,21E+08	2,06E+08	2,31E+08
G1SHV9	1,72E+09	1,82E+09	1,68E+09	8,36E+08	6,41E+08	5,06E+08
G1SHW6	54667000	0	0	0	0	0
G1SHX1	1,43E+09	5,61E+09	6,17E+09	5,14E+08	4,9E+08	5,02E+08
G1U660;G1SHY4	1,91E+09	1,05E+09	7,18E+08	5,54E+08	7,5E+08	8,97E+08
G1SHZ8	1,38E+10	4,52E+10	4,96E+10	8,53E+09	7,25E+09	7,07E+09
G1SI19	5,2E+08	9,3E+08	8,69E+08	5,04E+08	4,68E+08	8,07E+08
G1SI20	4,16E+09	1,38E+10	1,39E+10	4,63E+09	5,99E+09	5,57E+09
G1SI22	2,81E+09	1,74E+10	1,53E+10	5,47E+08	4,9E+08	7,59E+08
G1SI26	2,4E+09	7,99E+08	7,77E+08	1,2E+09	1,31E+09	1,9E+09
G1SI29	7,89E+10	2,33E+10	2,22E+10	2,47E+10	3E+10	3,08E+10
G1SI31	1,76E+09	6,15E+08	7,47E+08	5,84E+09	7,59E+09	8,58E+09
G1SI36	0	0	45131000	0	0	0
G1SI37	4,48E+10	9,06E+09	1,11E+10	2,2E+10	1,52E+10	1,82E+10
G1SI41;G1U2G6	0	0	3,59E+08	0	0	0
G1SI54	5,97E+08	6,1E+08	5,79E+08	9,65E+08	5,85E+08	4,25E+08
G1SI58	1,48E+08	2,17E+08	0	1,62E+08	2,03E+08	0
G1SI62	1,84E+08	1,24E+08	1,84E+08	0	0	0
G1SI68	2,17E+08	0	0	2,97E+08	3E+08	4,5E+08
G1SI71;U3KMZ1	3,78E+08	1,02E+09	5,01E+08	0	1,69E+08	0
G1SI74	1,32E+08	0	0	0	0	0
G1SI76	1,29E+09	3,01E+08	1,85E+08	7,72E+08	1,11E+09	7,84E+08
G1SI83	2,1E+09	5,21E+09	4,13E+09	3,44E+09	5,63E+09	4,22E+09
G1SI85	8,59E+09	1,57E+09	1,54E+09	4,33E+09	3,78E+09	3,29E+09
G1TZR3;G1SI89	7,11E+08	4,03E+08	3,36E+08	1,59E+09	1,57E+09	1,55E+09
G1SI95	5,49E+08	2,62E+08	2,11E+08	4,11E+08	4,83E+08	4,59E+08
G1SIB0	5,75E+09	2E+09	1,99E+09	1,2E+10	1,03E+10	1,19E+10
G1SIB1	0	1,32E+08	1,74E+08	0	0	1,32E+08
G1SIB2	2,03E+10	5,82E+09	6,5E+09	3,56E+09	3,53E+09	3,76E+09
G1SIB6	1,89E+09	1,15E+10	1,09E+10	8,42E+08	9,76E+08	1,18E+09
G1SIB9	6,35E+08	2,37E+09	2,23E+09	1,64E+09	1,61E+09	7,32E+08
G1SIC4;U3KND5	1,69E+09	1,79E+08	4,1E+08	7,72E+09	4,06E+09	4,36E+09
G1SIC7	0	0	69974000	0	0	0
G1SIE2	1,74E+08	0	0	0	0	0
G1SIE6	4,79E+09	1,68E+09	1,43E+09	3,91E+09	4,56E+09	3,66E+09
G1SIE8	7,26E+08	1,26E+09	7,26E+08	0	0	1,4E+08
G1SIF2;G1TEQ1	2,78E+10	7,75E+09	8,29E+09	7,5E+09	6,28E+09	7,44E+09
G1SIG2	0	1,58E+09	1,73E+09	4,41E+08	7,13E+08	7,71E+08
G1SIH0;G1STP1;B6A7Q3	4,53E+08	4,56E+09	3,4E+09	2,72E+08	2,56E+08	2,71E+08
G1SIH1;G1TIQ5	6,17E+08	2,42E+09	2,22E+09	7,62E+08	7,08E+08	7,03E+08
G1SIH3;G1TJP1	0	0	2,43E+08	0	0	0
G1SIJ0;G1SIJ6	5,45E+08	2,94E+08	1,16E+08	1,42E+08	1,41E+08	1,37E+08
G1SIJ1;G1TN01	79933000	0	0	0	0	0
G1SIJ2	6,5E+10	2,16E+10	1,79E+10	3,72E+10	4,5E+10	5,05E+10
G1SIJ7	1,55E+10	4,2E+09	3,26E+09	1,66E+09	8,63E+08	8,21E+08
G1SIJ8	0	2,51E+08	2,86E+08	0	0	0
G1SIK2	0	0	16714000	0	0	0
G1SIK4	5,72E+08	1,12E+08	1,66E+08	4,28E+08	1,77E+08	1,36E+08
G1SIL1	1,42E+08	2,72E+08	4,07E+08	0	0	0
G1SIL2	9,41E+08	2,94E+08	3,37E+08	2,65E+08	0	0

G1SIL8	0	7,21E+08	4,81E+08	0	1,67E+08	0
G1SIM3;U3KP10	1,07E+10	2,12E+09	2,04E+09	6,69E+09	5,45E+09	4,75E+09
G1SIN2	0	2,12E+08	1,87E+08	0	0	0
G1SIN4;G1SUC6	0	2,32E+08	2,94E+08	3,75E+08	0	0
G1SIN7	0	2,72E+08	2,82E+08	0	0	0
G1SIP1	4,04E+09	4,34E+08	2,06E+08	2,72E+09	2,17E+09	3,17E+09
G1SIP2	1,45E+10	4,64E+09	4,77E+09	7,22E+09	9,01E+09	8,9E+09
G1SIP6	5,74E+09	2,19E+09	1,69E+09	3,43E+10	3,52E+10	3,83E+10
G1SIP9	2,89E+09	8,55E+08	9,4E+08	8,84E+08	1,18E+09	1,08E+09
G1SIQ9	1,12E+09	4,73E+08	5,19E+08	0	1,9E+08	0
G1SIS2	1,38E+09	2,44E+08	3,58E+08	1,2E+09	6,3E+08	5,64E+08
G1SIT0	4,43E+08	3,22E+08	1,88E+08	4,33E+09	3,83E+09	4,82E+09
G1SIT5;G1U5E6;G1U7X5	3,47E+09	1,4E+10	1,52E+10	7,31E+09	6,22E+09	6,19E+09
G1SIT6	2,07E+08	0	0	0	0	0
G1SIT9	4,81E+09	2,18E+10	2,03E+10	3,66E+09	5,8E+09	6,09E+09
G1SIV3	1,69E+09	4,28E+08	4,76E+08	1,54E+09	1,48E+09	1,34E+09
G1SIV7	5,1E+08	8,41E+08	9,79E+08	0	0	0
G1SIV9	2,82E+08	1,12E+08	1,29E+08	3,15E+08	2,71E+08	1,18E+08
G1SIW1;G1SGY7;G1SUA9	8,28E+08	1,26E+09	1,34E+09	1,1E+09	7,88E+08	7,44E+08
G1SIW8	1,45E+09	1,17E+09	1,44E+09	5,74E+08	7,36E+08	5,39E+08
G1SIX1	1,9E+08	5,92E+08	4,96E+08	1,79E+08	2,25E+08	1,95E+08
G1SIX5	1,3E+08	0	0	0	0	0
G1SIY9	5,45E+08	7,63E+08	0	4,05E+08	3,5E+08	7,4E+08
G1SIZ2	3,57E+09	1,82E+10	1,73E+10	4,09E+09	4,66E+09	3,57E+09
G1SIZ4	0	3,99E+08	0	0	0	0
G1SJ10	6,73E+08	1,66E+08	9,79E+08	5,64E+09	2,22E+09	7,66E+09
G1SJ20	1,04E+09	5,7E+08	1,15E+09	1,87E+09	1,7E+09	2,38E+09
G1SJ23	3,67E+08	7,59E+08	1,42E+09	1E+09	8,4E+08	4,13E+08
G1SJ30	5,28E+08	97523000	73767000	0	0	0
U3KN72;G1SJ40	3,5E+08	0	0	1,04E+08	1,15E+08	1,02E+08
G1SJ41	1,05E+09	1,6E+09	1,48E+09	6,28E+08	6,3E+08	5,07E+08
G1SJ48;G1TG79	2,76E+08	0	0	0	4,34E+08	0
G1SJ56	8,71E+09	2,71E+10	3,2E+10	5,57E+09	7,86E+09	8,89E+09
G1SJ57	0	1,54E+09	6,9E+08	3,27E+08	3,26E+08	0
G1SJ66;U3KNB8	1,97E+09	6,56E+08	6,61E+08	1,36E+09	1,63E+09	1E+09
G1SJ77	5,57E+08	1,4E+09	4,38E+09	2,88E+08	6,86E+08	0
G1SJ87	0	0	2,07E+08	0	0	0
G1SJB4;G1TVH1;G1TI49	8,77E+09	4,82E+10	5,18E+10	1,41E+09	3,76E+09	1,71E+09
G1SJB9	2,68E+09	1,06E+09	8,24E+08	4,16E+09	4,12E+09	5,98E+09
G1SJC7	2,61E+08	1,7E+09	1,29E+09	2,11E+08	2,8E+08	2,81E+08
G1SJF1	2,9E+08	2,44E+08	3,35E+08	1,19E+08	1,68E+08	1,41E+08
G1SJF4	0	0	0	0	1,69E+08	0
G1SJF5	0	2,5E+09	1,16E+09	2,99E+08	0	0
G1SJG0	1,41E+09	4,86E+08	4,01E+08	2,32E+09	1,96E+09	2,01E+09
G1SJH8	2,64E+08	2,22E+08	2,18E+08	5,53E+08	5,53E+08	7,31E+08
G1SJI4	3,39E+08	5,97E+08	4,77E+08	9,86E+09	1,62E+10	7,67E+09
G1SJI6	2,17E+09	7,52E+08	7,16E+08	7,59E+08	9,68E+08	1,25E+09
G1SJI7	6,58E+08	0	0	2,14E+08	2,31E+08	0
G1SJI8	3,02E+09	9,5E+08	1,17E+09	3,21E+09	3,91E+09	3,26E+09
G1SJK0	1,04E+09	2,6E+08	3,16E+08	8,89E+08	9,88E+08	8,9E+08

G1SJK9	1,87E+09	4,96E+08	5,81E+08	5,05E+08	4,95E+08	5,5E+08
G1U8V4;G1SJL0	3,55E+08	0	0	5,01E+08	3,42E+08	3,16E+08
G1SJL6	6,91E+09	1,31E+09	8,5E+08	4,63E+09	3,12E+09	4,23E+09
G1SJL9	3,61E+08	5,35E+08	7,18E+08	8,8E+08	9,26E+08	1,15E+09
G1SJM6	2,41E+08	2,46E+08	2,21E+08	1,33E+09	1,07E+09	1,61E+09
G1SJN4	1,49E+09	5,17E+09	5,56E+09	4E+09	3,87E+09	4,84E+09
G1SJN5	8,88E+08	3,75E+08	3,78E+08	3,98E+08	3,21E+08	3,02E+08
G1SJQ2	2,1E+10	7,01E+09	6,38E+09	4,77E+10	4,86E+10	5,32E+10
G1U4I6;G1SJQ6	2,13E+08	0	0	0	0	0
G1T7P3;G1TZV1;G1SN51	3,22E+08	0	0	0	0	0
G1SJR2;G1SUG7	0	7,8E+08	6,59E+08	1,16E+09	1,73E+09	1,39E+09
G1SJR4	3,47E+09	5,1E+08	5,11E+08	2,95E+09	2,85E+09	2,86E+09
G1SSZ8;G1SJS1;G1SQE8	0	0	0	4,14E+08	4,52E+08	5,03E+08
G1SJU8	3,62E+08	0	0	0	0	0
G1SJV2;G1SVL9;G1U1H4	68622000	1,62E+08	1,06E+08	1,59E+08	94312000	98987000
G1T6M4;G1SJW7;G1U7T7	2,54E+09	2,08E+09	1,88E+09	4,03E+09	3,71E+09	3,92E+09
G1SJW8;U3KNR1	4,57E+08	1,12E+09	9,41E+08	1,43E+09	8,4E+08	9,59E+08
G1SJX2	2,01E+08	4,51E+08	6,78E+08	1,71E+08	2,57E+08	2,47E+08
G1SJX3;U3KMC6	4,66E+09	1,88E+09	1,33E+09	3,97E+09	4,99E+09	4,67E+09
G1SJY0	1,59E+09	4,09E+08	4,05E+08	4,49E+08	4,98E+08	2,24E+08
G1SJY1	0	86859000	2,01E+08	2,39E+08	1,35E+08	1,44E+08
G1SJY8	5,25E+08	9,4E+09	9,19E+09	1,88E+08	1,69E+08	1,91E+08
G1SJZ2;U3KN52	1,61E+08	87273000	0	0	0	0
G1SJZ4	5,37E+08	1,1E+09	1,29E+09	1,94E+08	2,57E+08	2,89E+08
G1SJZ8	2,01E+09	4,1E+08	3,38E+08	1,71E+09	2,06E+09	2,24E+09
G1SJZ9;P08628	2,76E+10	5,55E+10	6,23E+10	3,27E+09	3,83E+09	5,12E+09
G1SK00;U3KN42;G1SQ76	2,94E+09	1,16E+10	1,24E+10	3,64E+09	4,68E+09	4,67E+09
G1SK04	5,57E+08	3,94E+09	3,34E+09	3,47E+08	1,4E+09	3,89E+08
G1U743;G1SK09	0	0	0	7,88E+08	0	0
G1SK10	4,26E+08	4,14E+08	4,19E+08	6,03E+08	4,56E+08	5,3E+08
G1SK16	2,11E+08	0	0	0	0	0
G1SK17;G1U7M0	50231000	1,59E+08	1,24E+08	4,46E+08	2,26E+08	7,03E+08
G1SK22;P62975;G1SF46	6,91E+09	1,39E+10	1,7E+10	1,23E+10	1,41E+10	1,16E+10
G1SK25	4,55E+08	2,68E+08	3,13E+08	2,47E+08	1,39E+08	2,89E+08
G1SK33	3,66E+10	1,01E+10	1,09E+10	1,64E+10	1,54E+10	1,88E+10
G1SK48;P13019	1,71E+09	5,61E+09	5,74E+09	1,26E+09	1,46E+09	9,97E+08
G1SK49	1,04E+09	1,75E+08	4,35E+08	1,13E+09	9,78E+08	9,43E+08
G1SK52	1,94E+08	1,49E+09	1,41E+09	1,91E+08	2,12E+08	2,06E+08
G1SK61;G1U234;G1T9Q8	2,8E+08	0	0	0	0	0
G1SK67	1,9E+08	0	0	0	0	0
G1SK72	2,38E+08	0	0	6,03E+08	0	0
G1SK92	6,33E+08	1,59E+08	1,68E+08	2,02E+08	2,31E+08	0
G1SKA3	2,25E+08	2,14E+08	1,5E+08	0	0	0
G1SKD2	5,44E+08	0	0	0	0	0
G1SKD5;G1SD38	1,55E+08	8,76E+08	7,03E+08	1,65E+08	1,82E+08	78553000
G1SKD9	2,92E+10	8,13E+09	8,24E+09	1,89E+10	2,09E+10	2,12E+10
G1SKE0	2,64E+08	1,21E+09	1,28E+09	3E+08	0	3,11E+08
G1SKE2	1,78E+08	2,16E+08	2,4E+08	2,16E+08	2,04E+08	2,03E+08
G1SKE5	4,05E+08	2,55E+08	1,86E+08	4,65E+08	4,63E+08	6,54E+08
G1SKE7	3,73E+08	2,12E+08	1,37E+08	1,21E+09	1,41E+09	1,27E+09

G1SKF0;G1TWH4;Q95KK4	8,17E+09	2,37E+09	2,5E+09	5,27E+09	5,75E+09	7,11E+09
G1SKF1;G1SYF3	2,37E+09	8,91E+08	7,35E+08	1,7E+09	1,23E+09	1,18E+09
G1SKF5	4,02E+08	1,8E+08	1,32E+08	2,08E+08	2,79E+08	2,6E+08
G1SKF7;G1TG04;G1U1N5	7,32E+09	2,3E+10	2,24E+10	9,56E+09	6,72E+09	4,92E+09
G1SKH2	0	4,68E+08	5,58E+08	65283000	81098000	1,1E+08
G1SKH4	1,74E+08	0	74492000	0	1,29E+08	1,04E+08
G1SKI1	6,72E+08	7,09E+09	5,22E+09	3,96E+08	2,93E+08	5,59E+08
G1SKI8	5,4E+08	1,21E+08	83002000	0	0	0
G1SKJ4	3,55E+09	7,56E+08	7,43E+08	1,46E+09	1,3E+09	1,29E+09
G1SKJ5	5,01E+08	1,17E+09	8,96E+08	7,24E+08	6,69E+08	7,08E+08
G1SKJ8	6,96E+08	7,16E+08	5,19E+08	1,69E+09	1,55E+09	1,94E+09
G1SKK0	6,18E+08	5,75E+09	7,7E+09	5,83E+08	1,77E+08	3,49E+08
G1SKK1	2,35E+09	8,28E+09	1,3E+10	3,61E+08	3,48E+08	3,27E+08
G1SKK9	4,29E+08	3,81E+08	3,26E+08	2,04E+08	1,97E+08	2,15E+08
G1SKL7	7,4E+09	3,99E+09	3,8E+09	1,46E+09	1,32E+09	7,46E+08
G1SKM2;U3KME5;G1SUS5	1,39E+09	3,42E+08	3,94E+08	1,54E+09	7,53E+08	1,2E+09
G1SKM5	73825000	6,64E+08	5,96E+08	2,2E+08	1,92E+08	1,77E+08
G1SKN0	4,33E+10	9,79E+09	8,98E+09	3,01E+10	2,61E+10	2,55E+10
G1SKN2	3,19E+10	5,25E+09	3,65E+09	4,12E+10	3,54E+10	3,63E+10
G1SKN7	1,2E+09	5,18E+08	6,13E+08	5,25E+08	5,4E+08	5,7E+08
G1SKP1	6,85E+08	3,62E+08	3,7E+08	1,54E+09	6,24E+08	5,13E+08
G1SKP2;G1SV83	3,42E+09	8,16E+09	8,55E+09	2,03E+09	1,53E+09	6,05E+08
G1SKR3	3,59E+08	0	0	4,72E+08	6,12E+08	6,89E+08
G1SKR5	3,2E+08	0	0	0	0	0
G1SKS0	4,77E+08	5,72E+08	5,42E+08	4,47E+08	5,04E+08	3,2E+08
G1SKS8	6,83E+08	7,88E+09	7,95E+09	1,17E+09	1,43E+09	1,57E+09
G1SKS9	3,45E+09	9,71E+09	1,05E+10	3,65E+08	4,74E+08	2,58E+08
G1SKT1	7,18E+09	1,77E+09	1,83E+09	1,41E+10	1,3E+10	1,8E+10
G1SKT3	1,33E+09	0	3,38E+08	5,37E+08	3,93E+08	3,63E+08
G1SKT4	3,82E+11	7,49E+10	7,84E+10	2,97E+11	2,70E+11	2,32E+11
G1SKT7	3,71E+08	1,14E+08	1,44E+08	7,56E+08	7,73E+08	4,85E+08
G1SKU1	65387000	0	0	0	0	0
G1SKW5	1,98E+09	2,75E+09	2,81E+09	8,9E+08	9,13E+08	1E+09
G1SKY7	3,67E+09	3,15E+09	3,22E+09	3,16E+09	4,41E+09	4,81E+09
G1SKZ2;G1TIG1	8,07E+08	4,19E+08	3,24E+08	9,06E+08	1,19E+09	8,93E+08
G1SKZ3	1,14E+09	2,3E+10	2,35E+10	2,48E+08	5,41E+08	8,4E+08
G1SKZ8;G1TY53;G1TSL0	6,17E+09	1,14E+10	1,22E+10	6,22E+09	5,09E+09	3,58E+09
G1SL02	8,89E+09	8,76E+09	8,88E+09	4,05E+09	3,59E+09	3,04E+09
G1SL03	6,46E+08	3,2E+09	4,34E+09	3,57E+08	2,69E+08	4,45E+08
G1SL07	3,58E+09	2,41E+09	2,2E+09	9,78E+09	1,45E+10	1,52E+10
G1SL14	7,32E+08	3,5E+08	5,47E+08	2,19E+10	2,05E+10	9,85E+09
G1SL16	3,78E+09	3,69E+09	3,46E+09	3,63E+09	4,22E+09	5,2E+09
G1SL22	0	2,02E+08	0	4,17E+08	2,72E+08	2,5E+08
G1SL31	2,32E+08	0	0	0	0	0
G1SL32	5,06E+08	1,4E+08	2,06E+08	2,06E+08	2,15E+08	2,22E+08
G1SL38	3,91E+08	1,76E+09	1,37E+09	1,55E+08	3,35E+08	3,4E+08
G1SL41	1,87E+09	7,84E+08	8,24E+08	5,41E+09	4,27E+09	4,01E+09
G1SL42;G1TN54	7,84E+09	2,23E+09	2,07E+09	2,35E+09	2,66E+09	2,53E+09
G1SL46	2,2E+08	1,71E+09	1,54E+09	4,78E+08	8,16E+08	6,18E+08
G1SL51	6,76E+08	1,26E+09	1,45E+09	1,08E+09	1,39E+09	1,62E+09

G1SL57;U3KN01	1,67E+09	4,52E+09	5,04E+09	7,78E+08	1,07E+09	8,07E+08
G1SL60	6,04E+09	3,45E+09	4,54E+09	8,63E+09	6,99E+09	6,84E+09
G1SL62	4,26E+10	1,91E+11	1,74E+11	1,26E+10	2,69E+10	2,35E+10
G1SL68;P04461;G1TVQ7	2,06E+10	4,83E+10	5,82E+10	7,41E+09	6,08E+09	7,15E+09
G1SL80	2,16E+08	1,92E+09	1,81E+09	54355000	1,52E+08	2,5E+08
G1SL81	0	0	0	1,96E+08	0	0
G1SL85	7,09E+09	2,4E+09	2,2E+09	3,53E+09	2,81E+09	3,37E+09
G1SL95	78347000	1,44E+08	2,83E+08	77317000	1,08E+08	1,08E+08
G1SL97;G1TLQ3	1,68E+08	0	0	1,74E+08	1,49E+08	1,28E+08
G1SL98	1,96E+09	4,12E+08	5,4E+08	2,07E+09	1,09E+09	8,25E+08
G1SLA2	0	0	0	46542000	0	0
G1SLA3;P52786	1,31E+09	4,68E+08	3,02E+08	2,06E+09	2,1E+09	1,37E+09
G1SLA4	0	55346000	0	0	0	0
G1SLA5	1,65E+09	3,2E+08	3,02E+08	1,22E+09	8,81E+08	5,05E+08
G1SLB2	4,3E+08	1,69E+08	0	0	0	0
G1SLC0	2,41E+10	9,72E+09	8,54E+09	4,15E+10	4,56E+10	6,31E+10
G1SLC2;U3KNL5	2,64E+09	9,06E+09	9,54E+09	4,01E+08	5,25E+08	6,18E+08
G1SLC5	0	0	0	3,63E+08	3,12E+08	6,12E+08
G1SLD1	4,12E+09	1,3E+09	1,19E+09	6,57E+09	8,53E+09	7,3E+09
G1SLD5	5,29E+10	1,87E+10	1,6E+10	3,18E+10	2,92E+10	3,72E+10
G1SLD6	1,94E+09	3,44E+09	3,67E+09	1,27E+09	7,73E+08	9,4E+08
G1SLD7	3,37E+08	0	0	2,18E+08	0	0
G1SLE1	1,02E+09	0	1,45E+08	2,96E+08	3,14E+08	3,95E+08
G1SLE6	1E+09	1,97E+09	1,81E+09	1,84E+08	2,29E+08	2,67E+08
G1SLF1;O19053	1,28E+09	6,27E+09	6,11E+09	3,1E+09	3,43E+09	3,45E+09
G1SLF5	2,36E+08	1,59E+08	1,23E+08	1,52E+08	2,06E+08	4,53E+08
G1SLF6	4,94E+08	7,6E+09	5,24E+09	1,71E+08	2,79E+08	2,21E+08
G1SLF8;G1TVW9	0	0	0	33468000	0	0
G1U4L4;G1SWZ6;G1SLG0	7,36E+08	3,08E+09	3,85E+09	1,07E+09	1,44E+09	1,59E+09
G1SLG4	0	0	0	0	2,24E+08	0
G1SLH7	0	0	3,61E+08	0	0	0
G1SLI0	9,37E+09	1,63E+09	1,71E+09	1,04E+10	1,07E+10	1,36E+10
P80405;G1SLI3	2,28E+08	2,07E+08	0	1,43E+08	0	1,87E+08
G1SLI6	0	6,92E+08	1,12E+09	0	0	0
G1SLI8	25930000	0	0	0	0	0
G1SLI9	0	0	2,29E+08	0	0	0
G1SLJ6	5,89E+08	0	0	6,79E+08	3,82E+08	3,74E+08
G1SLJ8	5,35E+08	2,89E+08	2,55E+08	3,11E+08	2,75E+08	2,64E+08
G1SLJ9	5,49E+08	1,79E+08	2,87E+08	9,17E+08	5,77E+08	3,61E+08
G1SLK2;U3KN18;G1SDE4	8,16E+09	1,06E+10	1,03E+10	3,84E+09	3,85E+09	4,34E+09
G1SLK4	3,97E+08	3,03E+08	2,59E+08	3,19E+08	0	0
G1SLK6	90811000	1,09E+08	1E+08	1,29E+08	0	87956000
G1SLL1;G1U8Y2	1,26E+09	3,88E+09	4,23E+09	2,51E+09	3,09E+09	2,83E+09
G1SLL6	0	0	0	0	0	3,76E+08
G1SLL8	1,42E+09	6,21E+08	5,14E+08	4,12E+08	6,97E+08	5,98E+08
G1SLM0;G1TUR3;G1SIU2	1,01E+10	2,04E+10	2,52E+10	7,59E+09	8,04E+09	8,05E+09
G1SLM1	1,72E+09	7,04E+09	7,91E+09	3,16E+08	4,78E+08	3,36E+08
G1SLM2	3,54E+08	4,26E+08	3,55E+08	2,36E+08	3,54E+08	3,88E+08
G1SLN3;U3KNQ1	2,23E+09	1,91E+08	4,36E+08	7,59E+08	5,13E+08	8,92E+08
G1SLP3;G1TFE3;G1U223	1,34E+09	4,84E+08	5,79E+08	4E+08	3,9E+08	4,18E+08

G1SLP7	5,85E+08	2,54E+08	0	3,82E+08	3,7E+08	4,72E+08
U3KNP2;G1SLQ3	1,97E+10	5,05E+09	4,7E+09	5,72E+09	3,81E+09	3,45E+09
G1SLQ4;G1SDC1	8,45E+08	1,18E+09	1,6E+09	2,24E+08	2,49E+08	4,79E+08
G1SLR4	1,26E+08	89659000	1,35E+08	2,46E+08	5,1E+08	9,96E+08
G1SLS1;G1TQ02	2,35E+09	3,03E+08	3,66E+08	7,52E+08	6,31E+08	8,83E+08
G1SLS3	4,46E+08	3,29E+08	4,31E+08	1,77E+08	2,22E+08	0
G1TMH2;G1SLS7;G1TNU8	2,07E+08	0	0	98782000	0	0
G1SLS8	1,78E+09	6,06E+08	5,74E+08	1,86E+09	1,8E+09	1,41E+09
G1U8D5;G1TSI6;G1SLT2	7,73E+08	2,48E+09	2,72E+09	2,99E+09	4,95E+09	5,14E+09
G1SLT8	3,41E+09	1,9E+09	1,82E+09	2,11E+09	2,65E+09	3,63E+09
G1SLV3	7,37E+08	2,33E+08	2,18E+08	2,2E+08	2,01E+08	1,53E+08
G1SLW8	9,69E+08	4,41E+09	4,4E+09	2,35E+08	4,61E+08	3,75E+08
G1SLX0;G1U1T8	5,51E+08	4,33E+09	4,94E+09	6,05E+08	7,34E+08	1,06E+09
G1SLX9;U3KNQ3;U3KNM5	3,25E+08	5,65E+08	3,23E+08	6,01E+08	9,11E+08	8,49E+08
G1SLY1;G1TJ20;G1TLY6	89596000	1,57E+08	2,01E+08	1,31E+08	1,13E+08	0
G1SLY2	5,73E+08	6,13E+08	2,8E+08	1,02E+09	1,46E+09	1,64E+09
G1SLY8	1,5E+08	2,42E+08	2,02E+08	1,94E+08	0	2,72E+08
G1SLZ8	3,18E+08	4,55E+08	4,43E+08	0	0	0
G1SM02	1,67E+08	0	0	0	0	0
G1SM05	5,11E+09	2,77E+09	2,72E+09	5,23E+09	4,93E+09	5,24E+09
G1SM08	1,2E+09	1,48E+08	71513000	1,48E+08	0	86273000
G1STS2;G1SM13;G1T0A0	0	0	0	0	86160000	0
G1SM15;G1TZT2	2,47E+09	2,69E+09	3,22E+09	1,8E+09	1,52E+09	9,2E+08
G1SM23	0	0	3,71E+08	0	4,15E+08	0
G1SM27	7,59E+09	2,66E+09	2,54E+09	1,88E+10	1,86E+10	2,48E+10
G1SM28	0	0	0	1,35E+08	0	0
G1SM43	1,75E+09	4,06E+08	3,59E+08	7,21E+08	4,15E+08	4,42E+08
G1SM46	0	2,57E+08	2,41E+08	2,84E+08	4,56E+08	8,02E+08
G1SM48	2,48E+08	3,36E+08	3,49E+08	2,6E+08	3,39E+08	3,97E+08
G1SM50	1,25E+09	5,28E+08	5,35E+08	8,84E+08	9,89E+08	1,31E+09
G1SM51;U3KME2	5,59E+09	7,38E+09	7,84E+09	2,54E+09	2,49E+09	2,73E+09
G1SM52	2,29E+10	6,74E+09	6,77E+09	1,11E+10	1,06E+10	1,08E+10
G1SM60;G1TW86	3,24E+08	2,01E+09	1,81E+09	3,67E+08	3,48E+08	0
G1SM62;G1T2K1	3,58E+08	1,02E+09	1,13E+09	1,24E+09	1,51E+09	7,28E+08
G1SM76	3,69E+08	1,05E+09	1,16E+09	1,36E+09	1,82E+09	1,99E+09
G1SM77	5,67E+10	1,34E+10	1,29E+10	4,45E+10	3,92E+10	4,01E+10
G1SM91	0	5,19E+08	4,88E+08	0	0	0
Q28735;U3KMN2;G1SMA1	2,03E+10	5,3E+09	4,92E+09	4,37E+10	3,72E+10	3,71E+10
G1SMA6;G1SUF9;G1T6Q6	0	1,82E+08	1,66E+08	0	0	3,07E+08
U3KP00;G1SMA7	53175000	99418000	1,32E+08	0	0	0
G1SMB1	2,73E+08	4,64E+08	6E+08	4,91E+08	5,77E+08	4,35E+08
G1SMB3	1,8E+08	0	0	0	0	0
G1SMB8	0	1,58E+08	2,26E+08	0	0	0
G1SMC4	9,79E+08	6,92E+08	7,87E+08	1,94E+09	1,55E+09	2,07E+09
G1SME4;G1U738	8,3E+09	4,48E+09	5,76E+09	5,28E+09	5,39E+09	6,63E+09
G1SMG0	1,97E+09	3,64E+08	3,41E+08	2,49E+08	3,12E+08	0
G1SMG1;G1TMF1	0	4,31E+08	4,21E+08	0	3,09E+08	0
G1SMG5	2,23E+09	1,19E+10	1,27E+10	9,77E+08	1,81E+09	1,24E+09
G1SMH9	0	0	0	4,71E+08	7,75E+08	1,13E+09
G1SMI2	1,14E+10	2,18E+09	2,21E+09	2,31E+09	1,76E+09	1,47E+09

G1SMI6	1,86E+10	1,08E+10	9,93E+09	2,44E+10	2,33E+10	3,49E+10
G1SMI7	3,96E+09	4,66E+08	5,19E+08	1,54E+09	1,35E+09	9,25E+08
G1SMJ0	3,82E+08	1,37E+08	2,13E+08	1,35E+08	0	83946000
G1SMJ5	1,82E+08	1,03E+09	1,06E+09	1,84E+08	3,31E+08	2,41E+08
G1SMK8	1,86E+08	1,34E+08	1,23E+08	0	0	0
G1SMK9	3,1E+08	0	0	6,19E+08	1,09E+09	8,42E+08
G1SML4	3,07E+09	3,25E+08	3,91E+08	9,39E+08	5,17E+08	2,96E+08
G1SML5	1,43E+10	3,26E+09	3,17E+09	1,6E+10	1,28E+10	8,5E+09
G1SML9;G1U9C0	3,98E+09	4,3E+09	4,46E+09	1,31E+09	1,76E+09	1,82E+09
G1SMM1	0	9,27E+08	9,86E+08	0	0	0
G1SMM5	4,86E+09	4,72E+09	4,34E+09	1,99E+09	2,61E+09	2,42E+09
G1SMM7	7,34E+09	5,12E+09	4,09E+09	9,57E+09	7,76E+09	8,71E+09
G1SMN2	0	0	0	0	0	17913000
G1SMN3	1,43E+08	0	2,11E+08	2,92E+08	4,77E+08	5,49E+08
G1SMN9	8,22E+08	3,51E+08	0	9,98E+08	1,05E+09	8,29E+08
G1SMP3	3,01E+08	0	0	2,73E+08	2,74E+08	0
G1SMP5	2,01E+09	7,32E+08	4,72E+08	1,05E+09	7,92E+08	9,46E+08
G1SMP6;G1TSK5	36308000	1,4E+08	1,65E+08	0	0	0
G1SMQ4	6,37E+08	3,46E+09	3,58E+09	3,05E+08	3,09E+08	2,58E+08
G1SMQ9	0	3,69E+08	4,24E+08	2,33E+08	2,76E+08	3,47E+08
G1SMR7;G1TBC3	6,64E+09	1,03E+10	1,09E+10	6,02E+09	6,21E+09	7,43E+09
G1SMS2;G1SY79	0	4,79E+08	4,63E+08	4,02E+08	0	0
G1SMS3	3,69E+09	3,19E+09	4,58E+09	1,24E+09	1,05E+09	1,02E+09
G1SMT2	4,55E+08	3,46E+09	6,45E+09	4,23E+08	3,67E+08	1,04E+08
G1SMT7	0	6,1E+08	6,59E+08	1,68E+08	1,89E+08	1,14E+08
G1SMU4	4,52E+08	0	0	0	0	0
G1SMU8	3,72E+08	1,04E+09	1,09E+09	0	3,33E+08	4,01E+08
G1SMW5	0	0	0	0	19484000	0
G1SMW8	5,19E+08	6,2E+08	5,69E+08	4,35E+08	3,82E+08	4,02E+08
G1SMX4	25432000	0	0	0	0	0
G1SMY1	9,95E+09	8,93E+09	1,04E+10	2,65E+10	2,91E+10	3,17E+10
G1SMY4	8,3E+09	3,5E+09	3,51E+09	2,38E+09	1,26E+09	1,33E+09
G1SMY6	3,5E+08	1,52E+09	1,59E+09	0	2,5E+08	0
G1SMY7;G1U1W4	6,51E+08	3,78E+09	3,66E+09	6,45E+08	5,9E+08	6,59E+08
G1SMZ5	3,72E+09	6,29E+09	7,09E+09	8,76E+08	7,9E+08	5,54E+08
G1SMZ8	5,47E+09	1,25E+09	1,14E+09	3,31E+09	2,94E+09	3,58E+09
G1SN05	0	8,6E+08	1,1E+09	1,19E+09	1,1E+09	1,04E+09
G1SN06	1,86E+10	2,76E+09	4,13E+09	3,94E+09	2,42E+09	2,15E+09
G1SN08	1,93E+08	6,3E+08	3,17E+08	2,15E+08	0	0
G1SN09	0	4,21E+08	4,76E+08	0	0	0
G1SN11;G1T7I5;G1SD01	7,91E+09	9,99E+09	1,15E+10	1,47E+10	1,25E+10	6,77E+09
G1SN14;G1SWW2	8,53E+08	3,03E+09	2,62E+09	2,03E+09	1,59E+09	8,46E+08
G1SN16	5,72E+08	1,44E+09	1,44E+09	4,55E+08	7,2E+08	7,65E+08
G1SYB6;G1SN20	4,41E+08	1,51E+08	1,59E+08	2,42E+08	2E+08	1,64E+08
G1SN21	0	1,02E+09	1,16E+09	0	0	0
G1SN22	0	2,23E+08	2,16E+08	0	0	1,43E+08
G1SN26	0	0	95339000	0	0	0
G1SN37	1,23E+09	9,51E+08	9,33E+08	1,58E+09	1,61E+09	1,6E+09
G1SN43	0	0	0	0	0	22756000
U3KN70;G1SN52	0	0	60081000	0	0	0

G1TYG0;G1SN53	0	0	1,31E+08	0	0	0
G1SN55	2,34E+09	3,4E+09	3,57E+09	2,36E+10	1,78E+10	1,3E+10
G1SN56	0	2,2E+08	1,66E+08	2,8E+08	1,92E+08	2,02E+08
G1SN62	0	1,8E+08	0	0	0	0
G1SN66	3,47E+09	3,31E+08	3,96E+08	4,62E+09	4,94E+09	4,46E+09
G1SN67	3,4E+08	1,12E+09	9,96E+08	3,75E+08	4,13E+08	3,92E+08
G1SN68	4,29E+08	2,04E+09	1,91E+09	1,19E+09	1,31E+09	5,65E+08
G1SN73	59761000	0	47389000	0	0	0
G1SN74	6,48E+08	2,76E+08	0	0	0	0
G1SN83	1,38E+09	8,99E+08	5,84E+08	2,24E+09	1,94E+09	2,13E+09
G1SN91	1,67E+08	2,79E+09	3,32E+09	2,85E+08	0	0
G1SN95	1,4E+10	7,6E+09	6E+09	3,63E+10	4,2E+10	6,19E+10
G1SNB5;U3KPG8	9,68E+08	3,32E+08	2,83E+08	3,53E+08	3,53E+08	2,76E+08
G1SNB8	2,94E+08	7,18E+08	3,03E+08	0	0	0
G1SNC0	9,01E+08	6,85E+09	6,47E+09	7,04E+09	9,46E+09	8,52E+09
G1SNC3	52613000	0	0	0	0	0
G1U107;G1SNC4;P00569	4,94E+08	3,64E+09	3,93E+09	2,78E+09	3,84E+09	3,63E+09
P17635;G1SND1;P17636	3,75E+09	1,15E+09	1,23E+09	5,1E+09	3,12E+09	2,64E+09
G1SNE1	3,23E+08	2,43E+09	1,26E+09	0	2,23E+08	0
G1SNE5	3,21E+09	7,97E+08	4,31E+08	2,31E+08	0	2,87E+08
G1SNF2;G1SP76	3,47E+09	5,2E+08	5,08E+08	1,64E+08	2,37E+08	2,29E+08
G1SNH4	4,36E+08	0	0	4,68E+08	4,32E+08	6E+08
G1SNH7	6,69E+08	1,51E+09	1,49E+09	8,24E+08	1,11E+09	8,46E+08
G1SNI2	2,79E+08	0	1,4E+08	2,35E+08	2,04E+08	2,21E+08
G1SNI4	3,35E+09	1,58E+10	1,32E+10	3,03E+09	4,3E+09	5,53E+09
G1SNJ3	3,01E+09	1,34E+09	1,55E+09	2,87E+09	3,22E+09	3,83E+09
G1SNJ9	0	0	0	0	0	0
G1SNK5	39620000	4,23E+08	4,59E+08	0	1,32E+08	1,17E+08
G1SNK6	0	0	0	0	1,19E+08	0
G1SNK8	7,27E+08	2,13E+08	2E+08	1,47E+09	1,12E+09	1,5E+09
G1SNM1	8,75E+09	1,47E+10	1,46E+10	2,54E+08	2,43E+08	2,03E+08
G1SNM5	1,27E+08	0	0	0	0	0
G1SNM8	0	0	0	0	2,53E+08	0
G1SNP4;G1TXZ3	4,96E+09	8,1E+09	7,34E+09	1,56E+10	2,01E+10	2,23E+10
G1SNP7	0	0	0	0	0	37483000
G1SNP8;U3KLW8;G1TRA8	4,34E+08	4,3E+09	3,5E+09	2,91E+08	3,53E+08	3,16E+08
G1SNP9	4,86E+09	2,31E+09	1,74E+09	6,61E+09	5,59E+09	5,99E+09
G1TKS6;G1SNQ8	1,31E+09	7,74E+08	6,42E+08	6,79E+08	9,92E+08	1,17E+09
G1SNQ9	6,51E+09	1,45E+09	1,65E+09	6,6E+09	5,76E+09	3,59E+09
G1SNR2	7,53E+09	1,4E+09	1,66E+09	1,22E+09	1,26E+09	0
G1SNR6	47375000	0	0	0	0	0
G1SNS3	8,27E+08	2,5E+08	1,55E+08	2,36E+09	2,26E+09	2,7E+09
G1SNS8	2,71E+08	0	0	0	0	0
G1SNT1	5,17E+08	2,59E+09	2,94E+09	3,62E+08	4,17E+08	3,99E+08
G1SNT8	7,91E+09	2,55E+10	2,5E+10	6,73E+09	7,51E+09	7,02E+09
G1SNU3	4,61E+08	5,34E+08	6,5E+08	4,09E+08	5,04E+08	5,28E+08
G1SNV4	3,79E+09	2,41E+09	2,55E+09	4,43E+09	4,31E+09	6,59E+09
G1SNW2	1,28E+09	4,39E+08	6,31E+08	4,07E+08	0	4,2E+08
G1SNX5	2,49E+10	6E+09	5,61E+09	1,49E+10	1,39E+10	1,36E+10
G1SNY0;U3KML6;G1TWD4	4,34E+09	2,1E+10	1,49E+10	5,85E+09	4,83E+09	4,74E+09

G1SNY5	1,11E+08	1,1E+09	7,05E+08	1,6E+08	1,95E+08	1,5E+08
G1SNZ2	1,28E+08	0	0	0	0	0
G1SNZ3	5,84E+08	4,25E+09	4,31E+09	2,49E+08	1,38E+09	1,45E+09
G1SNZ8	9,69E+08	3,01E+08	1,76E+08	1,12E+09	1,63E+09	1,95E+09
G1SP02	3,12E+09	8,14E+08	8,4E+08	1,46E+09	1,11E+09	1,1E+09
G1SP11	1,29E+08	0	0	0	0	0
G1SP21	2,9E+08	1,15E+08	0	2,48E+08	2,73E+08	2,11E+08
G1SP22	3,77E+08	0	0	5,18E+08	5,73E+08	3,52E+08
G1SP24	9,68E+08	1E+09	1,04E+09	5,83E+08	4,67E+08	5,76E+08
G1SP25	6,58E+08	4,36E+08	4,04E+08	6,87E+08	6,16E+08	4,6E+08
G1SP27	2,52E+08	1,6E+09	1,66E+09	2,37E+08	3,25E+08	2,79E+08
G1SP30	4,79E+08	3,49E+09	2,66E+09	5,85E+08	5,47E+08	3E+08
G1SP32	3,55E+09	6,96E+08	6,37E+08	2,81E+09	2,21E+09	3,29E+09
G1SP33	1,27E+08	0	0	0	0	0
G1SP34	1,51E+09	1,2E+09	1,25E+09	4,59E+08	5,53E+08	5,7E+08
G1SP37	6,54E+08	62506000	1,4E+08	2,13E+08	6,68E+08	1,45E+08
G1SP45	2,18E+08	1,16E+09	1,38E+09	5,44E+08	5,65E+08	6,85E+08
G1SP48	3,55E+09	2,64E+10	2,8E+10	1,95E+08	1,81E+08	1,15E+08
G1SP51	4,7E+09	1,39E+10	1,57E+10	6,86E+09	6,09E+09	4,82E+09
G1SP54	2,55E+08	1,45E+09	1,68E+09	8,61E+08	9,97E+08	4,54E+08
G1SP61	9E+08	3,78E+08	3,18E+08	5,59E+08	8,13E+08	4,03E+08
G1SP68;G1SP71;G1TZV8	2,51E+09	5,12E+08	5,5E+08	9,96E+08	8,09E+08	6,32E+08
G1SP83	4,84E+09	1,47E+09	1,19E+09	8,55E+08	7,77E+08	8,56E+08
G1SP85	2,85E+08	0	0	0	0	0
G1SP89;G1TFZ5	5,33E+08	3,78E+08	2,31E+08	4,64E+08	3,54E+08	2,77E+08
G1SPA6	2,29E+10	1,05E+10	9,58E+09	3,07E+10	2,87E+10	3,77E+10
G1SPB2	0	0	2,83E+08	5,21E+08	6,57E+08	5,82E+08
G1SPB6	2,58E+09	4,76E+08	4,45E+08	3,79E+09	2,23E+09	1,91E+09
G1SPB8	1,2E+09	4,66E+09	4,21E+09	4,51E+08	8,13E+08	9,01E+08
G1SPD1	1,5E+09	9,81E+09	9,67E+09	1,94E+09	1,61E+09	3,01E+09
G1SPD2	7,58E+08	1,89E+09	2,08E+09	9,14E+08	1,08E+09	9,08E+08
G1SPE9	1,7E+08	82604000	69644000	0	0	0
G1SPF1	1,04E+09	2,95E+08	2,89E+08	6,76E+08	3,62E+08	6,79E+08
G1TWB2;G1SPF4	6,09E+08	0	73154000	2,13E+08	0	0
G1SPF5;G1U7S4	9,94E+09	4,28E+10	3,9E+10	1,36E+10	2,09E+10	1,78E+10
G1SPF6	6,29E+08	2,93E+08	2,58E+08	2,55E+08	2,61E+08	2,6E+08
G1SPG2	2,81E+09	2,25E+09	1,63E+09	3,7E+09	3,81E+09	4,49E+09
G1SPG6	6,62E+08	2,13E+08	2,27E+08	8,2E+08	7,21E+08	3,7E+08
G1SPH4	1,9E+08	7,25E+08	6,1E+08	2,2E+08	2,67E+08	2,82E+08
G1SPH7	1,99E+08	1,31E+08	0	0	96693000	99020000
G1SPH8	1,02E+09	3,46E+08	3,21E+08	7,01E+08	6,02E+08	4,52E+08
G1SPI6	1,87E+09	6,39E+08	5,23E+08	5,4E+08	7,25E+08	9,34E+08
G1SPI7	0	0	0	3,57E+08	0	0
G1SPJ2	4,92E+09	1,99E+09	1,83E+09	1,81E+09	1,94E+09	1,55E+09
G1SPJ5	5,38E+08	2,06E+09	1,82E+09	6,03E+08	3,68E+08	0
G1SPJ6	1,34E+08	5,82E+08	2,81E+08	2,4E+08	0	0
G1SPK4	7,57E+09	7E+09	7,3E+09	5,59E+09	4,58E+09	4,97E+09
G1SPL0	2,76E+08	2,91E+09	2,98E+09	1,47E+08	0	3,15E+08
G1SPL1	3,2E+08	7,76E+08	6,62E+08	2,8E+08	0	2,62E+08
G1SPL7	1,71E+08	4,38E+08	3,7E+08	5,6E+08	3,77E+08	7,52E+08

G1SPM5	1,18E+09	3,42E+09	3,54E+09	3,69E+08	3,41E+08	2,99E+08
G1SPN1	3,71E+08	2,4E+09	2,6E+09	4,09E+08	4,63E+08	4,82E+08
G1SPN3	6,39E+08	0	0	0	0	0
G1SPN7	0	4,95E+08	1,7E+08	81899000	0	0
G1SPN9	1,45E+08	4,27E+08	4,03E+08	0	0	0
G1SPP4	8,53E+08	3,62E+08	2,43E+08	3,94E+08	4,24E+08	6,95E+08
G1SPQ7;G1SVD2	1,37E+08	2,6E+09	1,21E+09	0	1,6E+08	1,23E+08
G1TB88;G1SPQ9	2,23E+09	4,89E+09	4,79E+09	8,68E+09	1,03E+10	9,42E+09
G1SPR5	1,91E+09	9,34E+08	9,25E+08	2,61E+09	1,92E+09	1,88E+09
G1SPR9	3,27E+10	9,6E+09	8,81E+09	3,9E+10	2,69E+10	2,02E+10
G1SPS5	0	0	0	81823000	0	0
G1SPS6	0	1,2E+08	0	0	0	0
G1SPS8	2,72E+08	2,5E+09	3,19E+09	0	0	2,95E+08
G1SPT2	0	3,65E+08	9,3E+08	2,04E+08	1,33E+08	0
G1SPU3	1,06E+08	0	0	0	0	0
G1SPU6	1,12E+11	1,26E+11	1,54E+11	7,15E+10	6,49E+10	8,42E+10
G1SPV0	4,39E+08	2,64E+09	3,01E+09	1,23E+08	0	2,87E+08
G1SPV4	0	9,21E+08	0	0	0	0
G1SPW1	0	0	0	0	0	19916000
G1SPY1	3,88E+08	2,25E+09	1,4E+09	3,17E+08	4,14E+08	0
U3KPB5;G1SPY8;P13355	1,59E+10	9,7E+09	7,84E+09	4,55E+10	4,85E+10	2,95E+10
G1SPY9	7,42E+09	1,55E+09	2,37E+09	2,62E+09	1,31E+09	1,71E+09
U3KN40;G1SPZ2	1,72E+08	8,55E+08	4,06E+08	2,25E+08	3,71E+08	0
G1SPZ7;G1T569	2,99E+08	1,08E+09	1,08E+09	6,76E+09	6,66E+09	8,25E+09
G1SPZ9;G1U2B1	2,47E+09	1,47E+09	1,24E+09	5,17E+08	8,59E+08	1,08E+09
G1SQ01;G1TDJ6	3,66E+09	1,66E+10	1,83E+10	3,74E+09	4,92E+09	4,51E+09
G1SQ02	4,05E+10	1,64E+11	1,71E+11	2,48E+10	3,96E+10	3,59E+10
G1SQ03	4,38E+08	0	0	1,13E+08	0	0
G1SQ07	1,1E+09	1,71E+09	9,03E+08	0	2,34E+08	0
G1SQ11	1,52E+10	3,04E+09	2,87E+09	7,9E+09	5,91E+09	6,49E+09
G1SQ22	2E+09	6,17E+09	5,51E+09	2E+09	1,87E+09	1,26E+09
G1SQ23	0	2,85E+08	0	0	0	0
Q9XSC5;G1SQ27	2,92E+10	1,82E+10	1,27E+10	1,6E+08	3,92E+08	1,29E+08
G1SQ29;G1TLG4;G1TSB8	0	2,93E+08	2,44E+08	0	0	0
G1SQ30	0	0	0	1,73E+08	94370000	0
G1SQ32	1,15E+09	3,09E+08	3,09E+08	9,93E+08	1,03E+09	7,06E+08
G1SQ44;G1U6I6;G1SQ49	8,73E+08	2,65E+08	3,97E+08	1,31E+09	6,95E+08	9,89E+08
G1SQ45	2,74E+08	47074000	38273000	0	0	0
G1SQ52	0	3,49E+08	4,23E+08	0	0	0
P43242;G1SQ54	4,21E+09	9,55E+08	9,95E+08	1,84E+09	1,48E+09	1,97E+09
G1SQ57	2,72E+08	3,92E+08	4,57E+08	5,42E+08	7,25E+08	7,11E+08
G1SQ70;G1TR31	2,78E+08	2,28E+08	1,72E+08	1,37E+10	8,9E+09	7,41E+09
G1SQ87	3,45E+08	3,13E+09	3,68E+09	7,25E+08	1,26E+09	1,53E+09
G1SQA4	8,21E+08	2,12E+08	2,61E+08	5,62E+08	3,03E+08	1,64E+08
G1SQA8	5,29E+11	1,99E+11	1,91E+11	4,72E+11	5,16E+11	6,36E+11
G1SQB1	8,34E+09	1,8E+09	1,96E+09	7,25E+09	6,14E+09	5,21E+09
G1SQD1;P41110;G1TR97	2,18E+09	4,93E+09	5,73E+09	1,35E+09	1,58E+09	8,65E+08
G1SQE6	1,56E+08	1,7E+08	2,53E+08	1,31E+10	1,24E+10	1,24E+10
G1SQF2	0	1,34E+09	1,52E+09	0	2,76E+08	3,18E+08
G1SQF7	1,73E+09	3,75E+08	5,03E+08	2,35E+09	2,01E+09	2,2E+09

G1SQF9	4,35E+09	5,36E+09	5,41E+09	9,19E+09	1,06E+10	1,13E+10
G1SQG1	4,98E+08	0	1,85E+08	6,51E+08	4,86E+08	5,29E+08
G1SQG5	7,91E+09	2,3E+10	2,16E+10	1,56E+10	1,72E+10	1,44E+10
G1SQG7;G1SJ22	0	0	0	0	0	0
G1SQH0;G1TET8	4,84E+09	2,11E+10	1,9E+10	6,04E+09	6,1E+09	5,33E+09
G1SQH1	0	0	0	0	0	1,48E+08
G1SQH2	7,76E+08	9,96E+08	1,06E+09	2,13E+09	1,91E+09	2,32E+09
G1SQI0	5,73E+08	1,46E+09	1,79E+09	6,68E+08	8,94E+08	1,26E+09
G1SQI2	0	62226000	0	0	0	0
G1SQI4	6,02E+09	2,47E+09	2,68E+09	1,74E+10	2,31E+10	2,97E+10
G1SQK0	0	1,99E+08	0	0	0	0
G1SQK1	0	4,75E+08	4,65E+08	0	88736000	61917000
G1SQK3	2,66E+08	5,41E+08	7,12E+08	0	4,38E+08	0
G1SQL0	2,4E+09	5,41E+09	5,08E+09	2,58E+09	2E+09	2,37E+09
G1SQL3	0	3,25E+08	3,47E+08	4,53E+08	3,75E+08	4,26E+08
G1SQM2	0	0	1,39E+08	0	0	0
G1SQM7	7,13E+08	7,7E+09	7,62E+09	2,46E+09	4,29E+09	4,91E+09
G1SQP0	1,82E+08	1,59E+08	1,47E+08	1,88E+08	1,64E+08	72781000
G1SQP9	3,07E+09	3,74E+09	3,73E+09	3,59E+09	2,68E+09	1,94E+09
G1SQQ1	1,3E+08	82314000	1,41E+08	6,34E+09	4,53E+09	7,67E+09
G1SQQ9	0	7,69E+09	6,2E+09	1,52E+10	8,82E+09	8,93E+09
G1SQR6	3,72E+09	3,77E+10	3,53E+10	1,3E+10	1,68E+10	2,32E+10
G1SQR7	4,09E+08	3,16E+09	3,04E+09	5,59E+08	1,02E+09	9,6E+08
G1SQS0	4,28E+08	0	0	0	0	0
G1SQS1	3,6E+08	1,54E+09	1,34E+09	4,71E+08	4,84E+08	3,29E+08
G1SQS5	2,63E+09	6,72E+08	9,12E+08	2,31E+09	1,94E+09	2,7E+09
G1SQS9;G1TPK9	6,02E+09	1,25E+09	1,35E+09	4,11E+09	4,26E+09	3,78E+09
G1SQT0	0	4,18E+08	3,91E+08	0	0	2,65E+08
G1SQT1	2,59E+09	4,95E+08	5,85E+08	1,84E+09	2,13E+09	2,22E+09
G1SQT2	8,22E+08	4,85E+08	7,65E+08	9,89E+08	7,14E+08	9,33E+08
G1SQU0	1,36E+10	7,7E+09	7,03E+09	3,89E+09	5,63E+09	6,46E+09
Q28677;G1SQU5	1,1E+09	7,34E+08	6,38E+08	1,6E+09	9,6E+08	7,7E+08
G1SQU6	1,79E+10	4,28E+09	3,66E+09	1,36E+10	1,34E+10	1,92E+10
G1SQV5	1,65E+09	5,82E+09	6,73E+09	6,61E+08	8,86E+08	1,18E+09
G1SQW0;G1U5I0	3,88E+09	2,1E+09	1,4E+09	9,78E+09	7,65E+09	4,43E+09
G1SQX9	0	0	0	0	0	9,67E+08
G1SQY8;G1TEZ4	1,22E+09	7,27E+09	6,66E+09	5,2E+08	4,76E+08	4,26E+08
G1SQZ2;G1TWS2	3,16E+08	4,72E+08	4,8E+08	0	0	0
G1SQZ4	3,87E+09	8,21E+09	8,49E+09	2,89E+09	2,52E+09	2,64E+09
G1SQZ5	0	0	0	1,74E+08	0	1,25E+08
G1SR03	3,6E+10	7,31E+10	7,78E+10	3,47E+10	3,9E+10	4,53E+10
G1SR13	1,87E+10	4,78E+09	4,49E+09	1,3E+10	1,41E+10	1,7E+10
G1SR16;U3KNN0	5,03E+08	1,83E+09	1,81E+09	1,14E+09	1,01E+09	1,33E+09
G1SR19;G1TXB5;G1SXP0	4,93E+08	0	1,02E+09	9,78E+08	7,56E+08	8,3E+08
G1SR20	0	1,69E+08	1,37E+08	0	0	0
G1SR25	3,45E+08	7,62E+08	4,44E+08	4,49E+08	2,23E+08	3,98E+08
G1SR27	2,18E+08	1,17E+09	1,45E+09	1,99E+08	2,33E+08	2,27E+08
G1SR28	2,38E+08	1,96E+09	2,08E+09	3,95E+08	4,38E+08	3,08E+08
G1SR29	1,11E+10	8,07E+09	7,82E+09	6,84E+09	7,62E+09	7,27E+09
G1SR36	4,26E+09	1,18E+09	1,07E+09	1,45E+09	1,76E+09	1,73E+09

G1SR45;G1TLI7	2,18E+10	8,15E+09	7,61E+09	7,45E+10	8,38E+10	7,45E+10
G1SR49	1,63E+08	1,31E+09	1,01E+09	0	1,25E+08	1,28E+08
Q9XSZ4;G1SR51;Q9XSZ4-2	4,42E+08	93445000	70742000	1,02E+10	7,32E+09	5,3E+09
G1SR61	6,3E+08	1,24E+08	1,36E+08	1,01E+08	63837000	1,66E+08
G1SR63	3,2E+08	0	1,48E+08	6,66E+08	2,67E+08	1,79E+08
G1SR73	0	0	0	6,9E+08	7,84E+08	1,47E+09
G1SR77;G1U572;G1TGY3	0	0	0	3,14E+08	2,62E+08	2,69E+08
G1SR79	1,56E+09	2,11E+09	2,14E+09	9,39E+08	1,04E+09	1,14E+09
G1SR83	0	0	0	6,68E+08	0	6,23E+08
G1SRA8;P33887	3,15E+09	3,58E+09	4,28E+09	1,01E+09	1,8E+09	1,06E+09
G1SRA9	1,62E+08	9,69E+08	1,53E+09	3,19E+08	1,21E+08	97320000
G1SRB6	1,45E+09	1,06E+10	1,1E+10	2,91E+09	4,78E+09	5,26E+09
G1SRB7	3,65E+09	2,01E+09	1,73E+09	4,16E+09	4,34E+09	4,42E+09
G1SRD0;Q8MJC3	0	1,63E+08	1,98E+08	0	0	0
G1SRD1	2,45E+08	0	0	5,91E+08	3,34E+08	2,77E+08
G1SRD2	2,98E+10	9,53E+09	9,7E+09	8,93E+09	1,15E+10	1,06E+10
G1SRD5	0	0	1,99E+08	3,62E+08	0	90331000
G1SRD9	2,41E+08	3,27E+08	3,9E+08	0	0	0
G1SRE0	1,55E+09	5,71E+08	4,12E+08	9,68E+08	8,19E+08	1,13E+09
G1SRE6;G1U429	1,54E+09	2,14E+09	1,9E+09	5,07E+09	5,47E+09	4,6E+09
G1SRE8	3,1E+08	3,33E+08	4,6E+08	4,98E+08	3,77E+08	4,3E+08
G1SRF1;U3KNU1	4,84E+08	0	1,79E+08	1,65E+08	0	0
G1SRF7	3,05E+11	7,51E+10	7,66E+10	1,05E+11	1,45E+11	1,63E+11
G1SRG3	4,94E+08	6,79E+08	7,17E+08	3,28E+08	3,46E+08	4,74E+08
G1SRG7	0	1,03E+08	0	1,56E+08	1,22E+08	0
G1SRH7	5,11E+09	7,99E+09	8,27E+09	8,59E+09	1,2E+10	1,41E+10
G1SRH8	0	1,23E+08	1,55E+08	1,31E+08	0	1,53E+08
G1SRH9	3,71E+09	1,24E+09	1,2E+09	5,24E+09	4,9E+09	5,37E+09
U3KNF3;G1SRI2	1,67E+08	5,97E+08	4,43E+08	1,49E+08	2,36E+08	0
G1SRI8;G1SMV5;G1SD81	5,87E+09	3,05E+09	2,44E+09	5,61E+09	4,06E+09	2,35E+09
G1SRI9;P45701	9,13E+08	1,42E+08	1,61E+08	3,77E+08	2,06E+08	2,1E+08
G1SRL3	1,85E+08	2,8E+08	7,2E+08	1,04E+08	0	0
G1SRL4	9,84E+09	4,54E+09	3,7E+09	7,2E+08	1,35E+09	1,78E+09
G1SRM4;O77821;G1U734	2,45E+09	2,31E+09	1,92E+09	1,2E+09	1,02E+09	1,29E+09
G1SRN1;U3KPK6	2,81E+08	0	0	0	0	0
G1SRN2;G1TU04	0	1,51E+09	1,45E+09	6,36E+08	4,84E+08	8,08E+08
G1SRP2	1,21E+09	3,89E+08	3,43E+08	9,85E+08	1,02E+09	6,23E+08
G1SRP7	1,89E+08	7,95E+08	8,54E+08	3,92E+08	3,67E+08	2,95E+08
G1SRP8	0	3,6E+08	2,49E+08	2,17E+08	2,69E+08	3,45E+08
G1SRQ2;U3KMW7	1,61E+09	8,75E+09	1,14E+10	1,86E+09	1,79E+09	2,62E+09
G1SRQ8	0	1,64E+08	0	0	0	0
G1SRQ9	3,29E+08	0	0	0	0	0
G1SRR2	0	0	2,81E+08	0	0	0
G1SRR4	78343000	0	0	1,25E+08	0	0
G1SRS0	1,53E+09	6,72E+08	6,89E+08	3,09E+09	2,53E+09	1,67E+09
G1SRS3	70467000	0	0	95115000	1,12E+08	0
G1SRT1	2,62E+08	1,91E+09	3,14E+09	3,9E+08	4,73E+08	3,58E+08
G1SRV1;G1U3G7	1E+09	3,73E+08	6,34E+08	4,03E+08	2,93E+08	2,95E+08
G1SRV2	0	3,17E+08	2,8E+08	0	0	0
G1SRW3	1,36E+08	4,32E+08	3,25E+08	0	0	0

G1SRW4	0	0	0	2,31E+08	0	0
G1SRX2	9,85E+09	4,57E+09	4,85E+09	8,06E+09	5,96E+09	6,39E+09
G1SRY1	1,18E+10	2,22E+09	2,3E+09	2,78E+09	2,61E+09	2,14E+09
G1SRY7	2,04E+08	0	0	2,08E+08	99776000	65940000
G1SRZ6	0	3,2E+08	3,29E+08	0	0	0
G1SS22	0	0	0	1,14E+08	1,32E+08	86804000
G1SS32	0	0	0	1,79E+08	2,4E+08	1,9E+08
G1SS33;U3KMI5;G1TIW5	2,56E+09	1,19E+09	1,06E+09	1,34E+09	2,26E+09	2,31E+09
G1SS37	5,93E+09	7,81E+08	8,99E+08	9,41E+08	1,12E+09	6,85E+08
G1SS49	0	1,31E+08	64100000	0	55860000	59381000
G1SS51	9,78E+08	2,31E+08	3,22E+08	2,52E+08	0	0
G1SS70;G1TN72;G1U444	1,05E+10	4,62E+10	4,38E+10	9,91E+09	1,18E+10	9,04E+09
G1SS73	2,04E+09	8,52E+09	9,38E+09	6,82E+09	8,5E+09	7,09E+09
G1SS77	0	83097000	61161000	0	0	0
G1SS79;G1U4U4	7,71E+08	4,59E+08	6,74E+08	1,24E+08	1,18E+08	0
G1SS85	0	2,01E+08	0	0	0	0
G1SS93	0	0	36279000	0	0	0
G1SSA2	8,46E+09	9,14E+09	9,55E+09	3,45E+09	3,22E+09	3,35E+09
G1SSB3	1,3E+08	74254000	0	0	0	0
G1SSB4	3,8E+08	6,67E+08	7,17E+08	3,56E+08	4,93E+08	3,09E+08
G1SSB5	5,04E+08	3,97E+08	7,47E+08	6,16E+08	4,44E+08	6,14E+08
G1SSC9;G1TX43;G1SCH2	2,05E+08	1,92E+09	3,11E+09	81359000	1,47E+08	89707000
G1SSF0	2,24E+08	2,52E+08	2,12E+08	1,63E+08	2,31E+08	1,96E+08
G1SSF2	6,31E+08	3,77E+08	2,2E+08	2,8E+09	2,78E+09	3,28E+09
G1SSF6	0	1,5E+09	7,75E+08	2,53E+08	2,08E+08	1,76E+08
G1SSG3;G1SIQ6;U3KMG0	0	0	0	30298000	0	0
G1SSG9	0	1,03E+08	86032000	0	91774000	1,09E+08
G1SSH0	1,3E+08	1,61E+08	1,74E+08	6,1E+08	4,67E+08	6,6E+08
G1SSJ7	7,2E+10	1,81E+10	1,69E+10	3,63E+10	3,27E+10	3,41E+10
G1SSK8	9,04E+10	1,79E+10	1,89E+10	4,38E+10	3,46E+10	4,44E+10
G1SSK9	2,3E+10	1,05E+11	1,11E+11	3,05E+10	3,93E+10	4,35E+10
G1SSL0	3,71E+10	2,05E+10	2,16E+10	4,49E+10	3,83E+10	6,62E+10
G1SSL2	1,86E+10	1,11E+10	1,15E+10	1,67E+10	1,76E+10	2,74E+10
G1SSM6	1,89E+09	7,05E+08	6,53E+08	6,81E+08	6,74E+08	4,1E+08
G1SSN2	1,87E+09	6,18E+08	1,07E+09	1,44E+09	1,39E+09	1,53E+09
G1SSN5	0	1,12E+08	1,23E+08	0	0	0
G1SSN9	1,59E+08	1,78E+08	1,89E+08	1,82E+08	0	0
G1SSP0	3,59E+08	4,91E+08	4,3E+08	2,98E+08	3,46E+08	3,3E+08
G1SSQ3	2,99E+08	0	0	2,49E+08	1,78E+08	1,93E+08
G1SSR3	1,58E+08	0	0	0	0	0
G1SSR8	5,16E+09	9,17E+08	1,38E+09	1,28E+09	1,37E+09	2,36E+09
G1SST6	2,1E+08	0	82624000	4,65E+08	4,86E+08	4,06E+08
G1SST7	9,12E+08	7,88E+08	5,23E+08	1,04E+09	1,32E+09	1,49E+09
G1SST8	0	0	0	1,65E+08	0	0
G1SST9	3,26E+09	1,11E+10	9,7E+09	3,45E+09	3,25E+09	4,91E+09
U3KNG3;G1SSU5	1,36E+08	0	0	2,19E+08	5,11E+08	3,84E+08
G1SSV1	5,52E+08	1,73E+09	1,41E+09	1,09E+09	1,07E+09	9,57E+08
G1SSW2	2,61E+08	6,06E+08	8,69E+08	1,94E+08	2,28E+08	2,25E+08
G1SSW3;G1TH11;G1TE10	6,96E+08	6E+08	4,85E+08	1,18E+09	1,55E+09	1,11E+09
G1SSX0	38951000	0	0	0	0	0

G1SSX2	2,26E+08	5,77E+08	7,15E+08	2,1E+08	0	2,12E+08
G1SSX5	2,51E+08	5,21E+08	6,33E+08	3,14E+08	3,15E+08	2,15E+08
G1SSY8	1,07E+09	2,86E+09	2,7E+09	0	0	0
G1SSZ1	1,69E+08	3,2E+08	2,13E+08	0	0	1,91E+08
G1ST05	5,45E+08	0	0	3,17E+08	0	2,74E+08
G1ST16	2,4E+08	1,09E+09	1,87E+08	4,38E+08	0	0
G1ST24	0	0	66753000	0	0	0
G1ST34	1,04E+09	0	0	1,89E+08	0	0
G1ST38	3,63E+08	1,76E+08	2,19E+08	1,15E+09	1,06E+09	4,3E+08
G1ST44	3,04E+08	0	0	0	0	0
G1ST49	8,49E+08	1,04E+09	1,12E+09	4,81E+08	4,49E+08	5,76E+08
G1ST52	1,72E+08	0	0	0	0	0
G1ST56	3,38E+08	7,37E+08	7,68E+08	0	0	0
G1ST58	2,47E+08	2,33E+09	2,39E+09	3,5E+08	0	2,89E+08
G1ST60	0	0	0	2,36E+08	0	1,66E+08
G1ST63;G1TLG5	54041000	1,07E+08	2,21E+08	62204000	79984000	54301000
G1ST66	4,83E+08	0	0	0	0	0
G1ST68;G1TKV1;G1TB85	2,06E+09	7,74E+09	9,22E+09	5,46E+09	7,71E+09	6,2E+09
G1ST69	3,41E+10	1,67E+10	1,54E+10	3,02E+10	2,5E+10	3,36E+10
G1ST78;G1TAQ7	6,19E+08	4,64E+08	0	1E+09	1,02E+09	0
G1ST81	3,29E+09	5,48E+08	6,44E+08	3,48E+09	2,81E+09	1,81E+09
G1ST83	5,3E+08	2,46E+08	0	1,36E+09	5,89E+08	5,53E+08
G1ST95	1,15E+09	7,68E+09	7,38E+09	7,34E+08	6,2E+08	7,2E+08
G1STA1	2,03E+08	0	0	4,27E+08	0	2,59E+08
G1STA9	0	0	0	0	0	0
G1STB1;G1U3P7	3,27E+08	4,1E+09	4,35E+09	3,7E+08	3,94E+08	4,66E+08
G1STB6;P68135;G1SL06	2,75E+10	5,71E+10	4,57E+10	6,96E+09	8,06E+09	1,65E+10
G1STC5	2,79E+08	0	0	3,12E+08	3E+08	5,54E+08
G1STC9	3,01E+08	1,92E+08	2,03E+08	1,03E+09	1,19E+09	1,35E+09
G1STD4	0	0	0	4,7E+08	0	0
G1STE1	1,73E+10	5,29E+09	5,14E+09	1,2E+10	1,59E+10	2,02E+10
G1STE3;G1T8H2	4,94E+08	2,03E+09	2,26E+09	1,93E+09	1,59E+09	1,99E+09
P19134;G1STF7;G1TKE4	8,2E+08	6,06E+08	7,45E+08	1,51E+09	1,68E+09	1,57E+09
G1STF8	2,6E+09	2,91E+08	2,22E+08	3,64E+08	2,69E+08	2,73E+08
G1STF9;Q5IH81	4,62E+09	1,45E+10	1,63E+10	1,19E+09	2,11E+09	2,06E+09
G1STG2	6,1E+09	1,65E+09	1,64E+09	4,66E+09	3,73E+09	2,73E+09
G1STG5	0	3,89E+08	4,56E+08	0	0	0
G1STG8	0	0	5,85E+08	0	0	0
G1STH0	7,09E+09	3,8E+09	3,86E+09	6,97E+09	7,19E+09	8,55E+09
G1STH4	1,98E+09	9,35E+08	1,06E+09	2,05E+09	1,46E+09	1,15E+09
G1STI3;G1T339	3,23E+09	2,46E+09	2,84E+09	5,96E+09	5,53E+09	5,72E+09
G1STJ3	0	0	1,26E+08	0	1,04E+08	1,02E+08
G1STJ8;G1TZ77	3,5E+08	0	0	0	0	0
G1STL1	0	0	0	0	0	1,85E+08
G1STM7;U3KN63	0	0	0	3,29E+08	0	0
G1STM8;G1U3C3	0	1,47E+08	0	0	0	0
G1STN6;G1TIP6	4,03E+09	1,22E+09	1,43E+09	7,07E+09	6,78E+09	8,29E+09
G1STP3	5,69E+09	1,56E+09	1,39E+09	1,05E+10	8,2E+09	1,41E+10
G1STP6;Q59I72;U3KNC8	1,92E+10	5,42E+09	6,22E+09	3,38E+10	3,03E+10	4,7E+10
G1STQ0	4,19E+08	0	0	4,7E+08	3,76E+08	4E+08

P10658;G1STQ6	3,6E+09	1,08E+10	9,9E+09	2,71E+09	2,99E+09	2,19E+09
G1STR6	0	1,14E+08	0	0	0	0
G1STS0;U3KMR0	6,26E+09	1,7E+09	1,99E+09	1,24E+09	1,63E+09	1,7E+09
G1STS3	0	0	5,83E+08	9,46E+08	9,85E+08	0
G1STS5	1,69E+08	0	77890000	2,47E+08	1,08E+08	1,36E+08
G1STS8	89546000	0	0	0	0	0
G1STS9	2,11E+09	5,02E+08	5,9E+08	1,32E+09	1,38E+09	1,54E+09
G1STU0	3,11E+08	1,72E+09	1,84E+09	4,69E+08	6,52E+08	5,6E+08
G1STU4	3,53E+08	0	0	0	0	0
G1STU7;G1TGR8;G1TJZ9	4,06E+10	6,51E+09	7,39E+09	3,66E+10	3,02E+10	3,05E+10
G1STV0;P08855	1,01E+09	9,72E+09	1,03E+10	1,91E+09	4,67E+09	3,47E+09
G1STV3;G1TNL4;G1TAM4	4,7E+08	0	0	4,06E+08	3,35E+08	3,67E+08
G1STW0	1,21E+10	2,67E+10	2,88E+10	1,27E+10	1,03E+10	8,66E+09
G1STW7	4,06E+08	6,05E+08	8,26E+08	2,33E+08	2,23E+08	0
G1STX3	2,79E+09	1,9E+09	2,03E+09	6,13E+09	4,41E+09	4,26E+09
G1STX4	4,83E+08	6,07E+08	6,13E+08	2,34E+09	2,69E+09	2,44E+09
G1STX7	8,92E+08	1,16E+09	1,31E+09	8,96E+08	1,05E+09	1,33E+09
G1STX9	2,29E+08	0	0	1,97E+08	1,59E+08	1,67E+08
G1TPR2;G1STY8	2,19E+08	9,18E+08	3,63E+08	0	1,66E+08	0
G1STZ4	6,85E+09	1,63E+10	1,57E+10	9,67E+08	9,68E+08	5,69E+08
G1STZ8	0	4,65E+08	2,04E+08	0	3,17E+08	1,6E+08
G1SU01	6,84E+09	1,11E+09	8,62E+08	6,31E+09	5,26E+09	6,55E+09
G1SU11;G1U053	7,12E+08	4,64E+08	4,15E+08	1,42E+09	1,07E+09	1,69E+09
G1SU15	2,8E+09	8,5E+08	6,6E+08	2,75E+09	2,01E+09	1,57E+09
G1SU17	2,31E+09	1,12E+09	6,67E+08	8,11E+08	6,72E+08	4,07E+08
G1SU30	8,23E+08	1,58E+09	1,69E+09	9,8E+08	9,42E+08	1,13E+09
G1SU33	1,04E+08	1,36E+08	2,93E+08	1,03E+08	1,13E+08	0
G1SU50	2,96E+08	0	0	3,08E+08	2,1E+08	1,73E+08
G1SU61	5,07E+09	1,27E+09	1,54E+09	1,12E+09	1,11E+09	1,3E+09
G1SU66	3,31E+08	5,45E+09	6,43E+09	0	1,01E+08	1,21E+08
G1SU71	3,62E+09	4,16E+09	3,49E+09	1,74E+09	1,9E+09	1,97E+09
G1SU75	9,97E+08	2,44E+08	3,25E+08	1,39E+09	1,13E+09	4,32E+08
G1SU97	5,77E+09	1,94E+09	1,92E+09	3,21E+09	4,52E+09	4,96E+09
G1SU98;G1SR41;G1STI0	6,05E+08	1,21E+10	1,65E+10	1,67E+08	1,81E+08	1,12E+08
G1SUA2;G1TQ14;G1TTF0	54305000	1,01E+09	1,13E+09	95344000	1,26E+08	76911000
G1SUA4;G1U611;G1TUI3	6,98E+08	1,34E+09	1,39E+09	4,25E+09	7,33E+09	7,55E+09
G1SUB5	0	0	0	2,45E+08	0	0
G1SUC4	0	0	0	0	86771000	0
G1SUC8	3,62E+08	2,8E+09	3,34E+09	1,62E+08	97355000	1,22E+08
G1SUD1	2,37E+08	3,41E+08	0	7,64E+08	1,17E+09	2,31E+09
G1SUD2	3,4E+09	7,07E+08	7,11E+08	4,18E+09	2,91E+09	2,11E+09
G1SUD9;G1U4Z7	2,37E+09	4,79E+08	3,29E+08	1,86E+09	1,94E+09	1,7E+09
O97862;G1SUE4	3,48E+08	1,58E+08	0	4,27E+08	5,01E+08	3,23E+08
G1SUE6;G1TQW9;G1TT51	6,57E+08	2,84E+08	2,22E+08	1,7E+09	5,32E+08	7,22E+08
G1SUE8	1,35E+08	9,6E+08	3,78E+08	1,59E+08	2,95E+08	2,31E+08
G1SUF4	3,29E+09	6,61E+08	7,04E+08	2,27E+09	1,77E+09	1,45E+09
G1SUF7	5,73E+08	2,93E+08	3,67E+08	6,83E+08	7,22E+08	6,7E+08
G1SUG3	4,65E+08	0	2,33E+08	1,18E+09	8,54E+08	6,65E+08
G1SUH5	1,63E+10	5,85E+09	3,75E+09	1,22E+10	1,45E+10	1,9E+10
G1SUI9	1,24E+09	1,93E+10	2,09E+10	5,06E+09	8,24E+09	7,7E+09

G1SUJ8	0	0	0	0	0	2,79E+08
G1SUK4	1,07E+09	8,43E+08	1,3E+09	5,34E+08	0	0
G1SUL3	2,15E+08	0	1,31E+08	1,47E+08	1,95E+08	1,69E+08
G1SUL7	9,15E+08	3,43E+08	3,07E+08	5,49E+08	4,28E+08	6,04E+08
G1SUM3	2,78E+08	1,37E+09	1,61E+09	2,76E+08	2,66E+08	2,56E+08
G1SUN1	7,45E+09	2,16E+09	2,6E+09	6,89E+09	7,42E+09	7,54E+09
G1SUN6	0	0	1,69E+08	0	0	0
G1SUP1;G1SYU6	1,92E+08	6,64E+08	5,82E+08	2E+08	1,8E+08	2,4E+08
G1SUP4	2,1E+08	2,81E+08	2,72E+08	4,6E+08	6,07E+08	6,63E+08
G1SUP8	1,19E+09	4,1E+08	3,85E+08	1,55E+09	1,51E+09	9,6E+08
G1SUP9	3,01E+09	3,44E+08	2,7E+08	1,68E+08	2,61E+08	2,31E+08
G1SUQ0	3,75E+08	1,67E+08	1,02E+08	0	0	0
G1SUQ9	2,57E+09	1,41E+09	2,42E+09	1,27E+09	1,21E+09	1,31E+09
G1SUR4	7,28E+09	1,2E+09	1,46E+09	5,97E+08	5,72E+08	2,32E+08
G1SUS6	1,25E+09	3,02E+08	3,25E+08	4,37E+08	4,23E+08	7,45E+08
G1SUT0	0	59298000	1,37E+08	0	31658000	0
G1SUT8	0	1,32E+08	0	0	0	0
G1SUU2	3,23E+09	2,09E+09	2,56E+09	2,75E+09	3E+09	3,72E+09
G1SUU7	1,93E+09	7,33E+09	7,09E+09	4,35E+09	4,8E+09	5,15E+09
G1SUV0	2,41E+08	96399000	66984000	0	89167000	0
G1SUV2	3,48E+08	1,25E+08	85977000	5,82E+08	4,51E+08	3,72E+08
G1SUX1;O97590	4E+08	1,23E+08	74335000	5,3E+08	1,68E+08	1,22E+08
G1TRC0;G1SUX8;G1U3E0	7,76E+08	1,59E+09	1,79E+09	2,98E+09	3,56E+09	3,65E+09
G1SUY2;G1SU45	2,93E+10	1,04E+10	1,01E+10	1,51E+10	1,07E+10	1,5E+10
G1SUY5	7,39E+08	2,82E+08	0	3,69E+09	1,34E+09	8,52E+08
G1SUY8	3,75E+08	4,06E+08	0	4,04E+08	7,99E+08	7,87E+08
G1SUZ7	2,67E+08	5,35E+08	0	9,49E+08	6,27E+08	7,05E+08
G1SV03	0	0	0	59075000	0	0
G1SV04	2,61E+09	3,14E+08	3,82E+08	3,16E+09	2,84E+09	3,71E+09
G1SV05	8,63E+09	2,49E+10	2,67E+10	9,29E+09	9,51E+09	8,96E+09
G1SV06	0	0	1,79E+08	0	0	0
G1SV12	1,39E+10	4,98E+09	4,35E+09	7,36E+09	7,2E+09	6,25E+09
G1SV13;Q29504	5,16E+09	2,43E+10	2,46E+10	7,74E+09	7,98E+09	7,91E+09
G1SV22	0	0	0	0	0	2,29E+09
G1SV24;G1TEK5;G1TCT0	6,59E+10	3,52E+10	4E+10	3,64E+10	3,15E+10	4,37E+10
G1SV32;G1TGX8	8,02E+09	1,83E+10	2,07E+10	9,03E+09	7,98E+09	4,65E+09
G1SV53	0	2,44E+08	2,93E+08	0	0	0
G1U097;G1SV58;G1SCT3	0	4,08E+08	4,22E+08	4,46E+08	2,66E+08	3,15E+08
G1SV59	0	0	0	4,13E+08	4,87E+08	4,59E+08
G1SV60	1,04E+09	2,26E+08	1,54E+08	3,95E+08	3,35E+08	1,88E+08
G1SV63;U3KPG1	0	0	1,96E+08	0	1,95E+08	3,09E+08
G1SV65	0	80884000	75368000	0	0	0
U3KPM5;G1SV69	0	0	2,21E+08	0	0	2,57E+08
G1SV75	2,25E+09	5,36E+08	4,95E+08	1,47E+09	1,69E+09	1,62E+09
G1SV79	0	1,66E+08	0	0	0	0
G1SV81	0	4,89E+08	0	0	0	0
G1SV82	32914000	0	0	0	0	0
G1SV87	4,36E+08	0	0	1,54E+08	0	2,03E+08
Q09YN8;G1SV99	0	3,27E+08	5,57E+08	3,38E+08	2,58E+08	2,79E+08
G1SVA3	6,28E+09	8,15E+09	8,05E+09	3,47E+09	3,19E+09	2,82E+09

G1SVB0;G1U0Z0;G1U598	1,08E+10	3,31E+10	2,79E+10	9,8E+09	1,1E+10	1,01E+10
G1SVB3	1,3E+08	0	0	0	0	0
G1SVB6;G1TV20	7,73E+10	2,99E+10	2,42E+10	2,58E+10	2,53E+10	2,34E+10
G1SVC4	4,29E+08	0	0	0	2,7E+08	2,55E+08
G1SVD5	9,5E+09	3,17E+09	3,4E+09	8,1E+09	5,25E+09	4,38E+09
G1SVE2	1,3E+10	6,53E+09	5,89E+09	6,96E+10	4,79E+10	5,72E+10
G1SVE5	0	0	0	0	1,01E+08	0
G1SVE6;G1U1J2;G1TGM4	1,75E+08	1,44E+09	1,79E+09	1,54E+08	2,31E+08	3,41E+08
G1SVF2	7,25E+09	1,09E+10	1,24E+10	4,13E+09	3,57E+09	4,09E+09
G1SVF9	9,31E+08	4,22E+08	4,26E+08	0	3,64E+08	2,94E+08
G1SVH0	2,71E+09	1,57E+10	1,45E+10	8,37E+09	9,61E+09	8,11E+09
G1SVH1	1,33E+09	1,31E+08	1,13E+08	1,4E+08	1,1E+08	87666000
U3KM97;G1SVI1	3,74E+08	4,44E+08	4,35E+08	6,01E+08	6,02E+08	5,74E+08
G1SVI3	1,84E+08	0	0	3,82E+08	0	0
G1SVI9	1,05E+10	2,64E+09	3,2E+09	1,1E+10	1,32E+10	1,3E+10
G1SVJ2	0	0	0	0	2,28E+08	0
P13642;G1SVJ5	1,58E+09	2,13E+09	2,54E+09	4,42E+08	4,9E+08	4,63E+08
G1SVK5	1,74E+08	3,89E+09	4,25E+09	1,59E+08	2,24E+08	82745000
G1TZX9;G1SVL8	0	0	3,15E+08	0	0	0
G1SVM1	2,55E+09	5,52E+09	8,1E+09	4,07E+09	4,01E+09	5,47E+09
G1SVM2	7,35E+08	0	0	5,93E+08	6E+08	7,15E+08
G1U2Q4;G1TMN0;G1SVP6	8,67E+08	7,72E+08	5,94E+08	1,29E+09	1,64E+09	2,42E+09
G1SVP7	1,42E+09	3,86E+09	3,81E+09	6,26E+08	8,42E+08	5,14E+08
G1TB22;G1SVP9	1,57E+09	9,15E+08	1,1E+09	1,79E+09	1,98E+09	2,66E+09
G1SVQ8;G1TL37	6,07E+09	1,46E+09	1,39E+09	7,45E+09	9,01E+09	7,81E+09
G1SVR2	0	0	0	79270000	0	0
G1SVR6;G1U988	0	0	0	0	1,64E+08	0
G1SVS0	4,97E+08	3,5E+08	0	1,36E+09	7,52E+08	4,53E+08
G1SVS1	3,07E+08	0	0	0	0	0
G1SVT3	1,69E+09	1,78E+09	1,74E+09	2,59E+09	3,33E+09	3,95E+09
G1SVT4	4,12E+09	4,42E+09	3,77E+09	2,27E+09	1,63E+09	1,96E+09
G1SVU0	2,59E+09	1,11E+09	7,94E+08	1,98E+09	1,75E+09	2,77E+09
G1SVU1	2,02E+08	3,4E+08	3,13E+08	0	0	0
G1SVV2	2,08E+10	6,06E+09	7,19E+09	3,91E+09	3,07E+09	2,28E+09
P29678;G1SVV6	4,83E+08	6,6E+09	5,15E+09	4,16E+08	4,54E+08	3,67E+08
G1SVW5	1,07E+10	2,4E+10	2,48E+10	9,32E+09	7,04E+09	4,9E+09
G1SVW7	6,1E+08	0	0	1,82E+09	7,98E+08	4,58E+08
G1SVW9	1,21E+08	0	0	0	0	0
G1SVX5	0	1,53E+08	1,37E+08	0	0	0
G1SVY6	2,49E+08	1,54E+08	1,92E+08	2,18E+08	1,78E+08	1,7E+08
G1SVY8;P00567	8,12E+09	3,81E+10	5,03E+10	1,4E+09	2,62E+09	4,76E+08
G1SW00	3,6E+08	6,76E+08	6,42E+08	6,67E+08	1,51E+09	6,41E+08
G1SW09	0	0	4,12E+08	5,02E+08	6,84E+08	5,54E+08
G1SW10	1,28E+09	1,2E+09	1,35E+09	4,38E+09	2,52E+09	1,61E+09
G1SW24	3,44E+09	6,26E+09	1,01E+10	5,76E+08	6,67E+08	3,51E+08
G1SW25	41364000	0	0	97536000	67919000	0
G1SW32;G1TN91	2,77E+08	3,34E+09	9,5E+08	1,43E+08	4,11E+08	1,23E+08
G1SW36	1,58E+09	5,44E+08	4,32E+08	1,22E+09	6,76E+08	7,67E+08
G1SW44	0	6,15E+08	4,94E+08	0	0	0
G1SW48	0	3,17E+08	0	0	0	0

G1SW61	1,45E+09	3,55E+08	3,98E+08	9,98E+08	5,99E+08	5,58E+08
G1SW66	1,13E+08	53049000	52372000	59683000	36489000	25717000
G1SW75	0	0	2,95E+08	0	0	2,25E+08
G1SW77;U3KN98	1,41E+09	2,62E+10	2,61E+10	3,06E+09	4,72E+09	5,12E+09
G1SW78	1,32E+08	4,51E+08	5,47E+08	1,22E+08	2,38E+08	2,36E+08
U3KN12;G1SW89	2,94E+08	5,39E+08	0	7,86E+08	6,39E+08	6,52E+08
G1SW91	4,22E+08	0	0	0	0	0
G1SW97	1,37E+10	5,52E+09	4,4E+09	8,83E+09	9,33E+09	8,75E+09
G1SWA1;G1SLR8	0	1,3E+08	0	1,9E+08	0	0
G1SWA5	1,22E+08	89529000	97086000	1,9E+08	1,37E+08	2,44E+08
G1U008;G1SWA6	1,14E+09	6,56E+08	7,71E+08	9,25E+08	9,39E+08	1,1E+09
G1SWC2;G1SWC0	4,07E+08	3,11E+08	3,12E+08	9,58E+08	1,15E+09	1,05E+09
G1SWC9	3,21E+08	5,48E+08	4,63E+08	6,26E+08	9,78E+08	1,07E+09
G1SWD1	5,24E+09	1,43E+09	1,24E+09	3,05E+09	2,11E+09	1,47E+09
G1SWD3;G1T0J1;G1SRW6	2,15E+08	4,39E+08	5,38E+08	1,17E+08	1,66E+08	0
G1SWD8	0	0	0	3,77E+08	0	0
G1SWD9	0	0	2,03E+08	0	0	0
G1SWE4	2,4E+08	2,38E+08	0	0	2,1E+08	3,36E+08
G1SWE5	3,21E+08	1,3E+09	1,23E+09	2,54E+08	4,21E+08	4,04E+08
G1SWF2	2,96E+08	1,82E+09	1,47E+09	3,79E+08	3,83E+08	4,57E+08
G1SWF3	1,41E+09	1,48E+08	1,22E+08	6,9E+08	3,27E+08	3,58E+08
G1SWI3;P68003	2,87E+10	1,12E+10	9,15E+09	5,19E+10	5,21E+10	3,92E+10
G1SWI7	2,16E+09	3,64E+09	4,73E+09	1,81E+09	1,56E+09	1,64E+09
G1SWJ0	1,82E+09	6,49E+08	9,04E+08	8,46E+08	5,45E+08	9,16E+08
G1SWK3;U3KNS5	3,45E+08	3,72E+08	2,6E+08	0	0	0
G1SWK4	0	0	0	0	0	2,27E+08
G1SWK5	8,63E+08	4,99E+08	2,34E+08	1,19E+09	5,29E+08	4,66E+08
G1SWK8	1,57E+09	1,98E+09	1,81E+09	5,82E+08	7,12E+08	7,96E+08
G1SWL6	0	3,86E+08	5,07E+08	3,32E+08	0	3,24E+08
G1SWM1	0	0	0	0	0	3,87E+08
G1SWM7;U3KPM4	2,06E+09	5,13E+08	4,08E+08	1,68E+09	1,99E+09	2,36E+09
G1SWN4	2,53E+08	1,08E+09	4,14E+08	1,05E+08	1,54E+08	1,6E+08
G1SWN7	9,08E+08	3,58E+08	3,02E+08	8,27E+08	5,54E+08	6,09E+08
G1SWP1	7,49E+08	0	0	0	0	0
G1SWR0	2,52E+09	1,15E+09	1,19E+09	1,25E+09	1,54E+09	2,06E+09
G1SWR1	6,06E+08	2,86E+08	1,78E+08	2,55E+08	2,05E+08	99123000
G1SWR7	2,08E+08	0	0	2,65E+08	2,25E+08	0
G1SWS6;O46378	0	4,34E+08	0	1,38E+09	4,37E+08	9,35E+08
G1SWS9;G1SRX8;G1SMX2	2,09E+12	7,80E+11	6,53E+11	9,79E+11	9,16E+11	6,22E+11
G1SWT6	4,62E+08	2,56E+08	2,58E+08	4,18E+09	7,32E+09	6,11E+09
G1SWT7	6,44E+08	2,58E+08	1,9E+08	1,75E+08	1,97E+08	1,85E+08
G1SWU1	2,35E+10	7,34E+09	6,82E+09	1,09E+10	1,22E+10	1,28E+10
G1SWU8	0	1,89E+08	1,29E+08	0	0	0
G1SWV9	6,99E+08	3,94E+08	3,94E+08	7,69E+08	6E+08	1,01E+09
G1SWW4	0	5,32E+08	6,4E+08	8,12E+08	7,75E+08	1,08E+09
G1SWW6	0	1,59E+08	2,84E+08	1,16E+08	0	1,52E+08
G1SWW7	6,62E+08	6,49E+08	6,6E+08	4,39E+08	7,85E+08	8,44E+08
G1SWW8	3,92E+08	8,02E+08	9,45E+08	4,29E+08	4,04E+08	0
G1SWY0	1,27E+10	3,4E+09	3,58E+09	1,88E+10	1,36E+10	8,22E+09
G1SWY2	1,95E+08	0	0	1,81E+08	2,35E+08	2,19E+08

G1SWY4;G1TS94	1,05E+09	2,53E+08	2,92E+08	2,76E+08	7,69E+08	2,47E+08
G1SWY6	9,37E+08	1,42E+09	1,5E+09	5,91E+08	4,54E+08	3,89E+08
G1SWY7	88939000	0	0	1,4E+08	2,15E+08	0
G1SWZ1	3,11E+08	2,13E+09	2,4E+09	7,37E+08	6,38E+08	1,26E+09
G1SWZ5	39134000	0	0	0	0	0
G1SX00	2,3E+09	2,28E+09	2,42E+09	5,99E+09	7,28E+09	9,26E+09
G1SX03	2,71E+08	69054000	1,51E+08	4,68E+08	4,57E+08	1,83E+08
G1SX32	3,01E+08	1,57E+09	1,71E+09	2,5E+08	2,53E+08	2,87E+08
G1SX37	3E+08	3,1E+09	2,92E+09	2,97E+08	4,26E+08	3,46E+08
G1SX44	2,65E+09	4,87E+08	6,22E+08	7,98E+08	6,79E+08	9,72E+08
G1SX47	7,43E+08	0	0	6,55E+08	7,15E+08	6,14E+08
G1SX50	1,22E+08	0	0	0	0	0
G1SX70	2,45E+08	2,35E+08	0	2,39E+08	2,93E+08	0
G1SX71	0	0	0	3,18E+08	2,44E+08	0
G1SX73	7,84E+08	4,02E+09	4,17E+09	7,33E+08	7,96E+08	8,43E+08
G1SX89	6,34E+08	0	0	3,37E+08	2,48E+08	2,87E+08
G1SX92	0	0	1,55E+08	0	0	0
G1SXA5	2,48E+10	1,05E+10	9,78E+09	3,88E+10	2,62E+10	4,3E+10
G1SXA8	0	1,83E+08	0	0	0	0
G1SXB6	6,75E+08	1,8E+08	2,02E+08	1,13E+09	6,78E+08	5,63E+08
G1SXB8	6,75E+08	5,64E+08	7,23E+08	3,09E+08	0	0
G1SXC8	89682000	0	0	0	0	0
G1SXD6	2,91E+09	9,91E+08	8,75E+08	4,62E+09	7,15E+09	5,22E+09
G1SXE6	1,76E+08	7,71E+08	4,61E+08	1,08E+08	0	1,33E+08
G1SXF1	3,97E+09	6,64E+08	7,07E+08	1,13E+09	2,38E+09	2,17E+09
G1SXG8	2,05E+09	5,52E+08	4,98E+08	9,16E+09	5,61E+09	7,12E+09
G1SXH2	0	0	3,91E+08	0	0	0
G1SXI9	2,33E+10	7,85E+09	5,98E+09	4,16E+10	5,38E+10	6,37E+10
G1SXJ0	1,47E+09	2,62E+09	2,21E+09	3,64E+09	5,62E+09	7,15E+09
G1SXJ3	0	0	0	0	0	38435000
G1SXJ6	0	0	0	1,46E+08	0	0
G1SXK6	0	1,33E+09	1,37E+09	1,07E+08	1,36E+08	91257000
G1SXK7	1,87E+08	0	0	0	0	0
G1SXL3;G1TQF7;G1SCD7	6,09E+09	1,89E+10	1,77E+10	3,23E+09	4,61E+09	5,38E+09
G1SXL9	1,13E+09	2,23E+08	1,51E+08	6,01E+08	4,34E+08	3,12E+08
G1SXN0	3,21E+08	1,28E+08	1,48E+08	1,73E+08	0	1,22E+08
G1SXP2	1,13E+08	5,57E+08	6,12E+08	0	0	0
G1SXP8	29119000	0	0	0	0	0
G1SXQ0	2,13E+09	6,13E+09	6,21E+09	2,01E+09	2,42E+09	1,92E+09
G1SXQ1	4,11E+09	1,01E+09	9,85E+08	4,19E+09	3,65E+09	3,75E+09
G1SXR6	0	0	0	0	0	2,31E+08
G1SXR7	1,34E+10	3,59E+09	2,99E+09	3,24E+09	4,43E+09	4,5E+09
G1SXS3	0	0	0	0	0	2,14E+08
G1SXT1	2,61E+09	2,55E+09	2,41E+09	7,28E+09	6,64E+09	6,53E+09
G1SXT7	5,6E+10	1,85E+10	1,58E+10	8,89E+10	8,25E+10	9,44E+10
G1SXU2;G1U0H1	3,96E+09	2,27E+10	2,14E+10	1,22E+09	1,36E+09	1,13E+09
G1SXU3;G1TMR9	0	0	1,82E+08	0	0	0
G1SXW0	4,91E+09	1,72E+09	1,49E+09	4,78E+09	4,93E+09	4,37E+09
G1SXX2	4,31E+09	5,71E+09	5,49E+09	6,46E+09	8,25E+09	8,87E+09
G1SXX3	0	3,41E+08	3,49E+08	0	0	0

G1SXX4;U3KMB9;G1TZ87	0	2,92E+08	1,81E+08	0	0	1,18E+08
G1SXX5	5,1E+10	8,44E+09	7,89E+09	2,92E+10	2,43E+10	2E+10
G1SXX8	7,73E+08	7,7E+08	0	2,01E+09	1,35E+09	6,1E+08
G1SXZ9	2,88E+09	1,12E+09	1,05E+09	1,92E+09	1,94E+09	2,08E+09
G1SY02	0	1,1E+09	1,23E+09	0	5,38E+08	5,38E+08
G1SY05	0	6,59E+08	4,44E+08	3,66E+08	3,88E+08	4,89E+08
G1SY12	4,1E+08	2,65E+08	2,01E+08	0	0	0
G1SY15;G1TQJ8	0	0	0	2,25E+08	0	0
G1SY18	1,56E+09	4,63E+08	3,32E+08	1,03E+09	1,23E+09	1,68E+09
G1SY19;U3KNR7	1,64E+09	9,98E+08	6,92E+08	9,71E+08	1,11E+09	1,98E+09
G1SY27	1,87E+09	9,39E+08	1,21E+09	1,4E+09	2,71E+09	3,51E+09
G1SY28;Q9TTT7	1,36E+10	4,51E+09	2,69E+09	1,64E+10	1,64E+10	3,26E+10
G1SY30	5,07E+09	2,67E+09	2,29E+09	5,82E+09	4,16E+09	2,55E+09
G1SY36	5,22E+09	2,21E+10	2,32E+10	3,69E+09	4,09E+09	3,95E+09
G1SY38	0	6,08E+08	5,74E+08	0	0	0
G1SY47;G1U1Z0	2,56E+08	2,81E+08	2,19E+08	6,89E+08	4,01E+08	7,9E+08
G1SY50	1,31E+10	3,48E+09	3,1E+09	1,17E+10	1,44E+10	1,71E+10
G1SY53;G1U4H0	2,86E+09	1,25E+10	1,16E+10	2,78E+09	3,54E+09	2,64E+09
G1SY62;G1TQ21	2,98E+08	2,41E+08	0	0	0	0
G1SY65;G1U5T1	3,55E+08	2,1E+09	2,3E+09	0	0	0
G1SY68;G1TPR8	3,5E+09	2,79E+09	2,64E+09	8,31E+09	8,46E+09	1,14E+10
G1SY70	1,07E+09	3,47E+09	3,74E+09	5,55E+08	5,94E+08	2,29E+08
G1SY84;G1T2U7	4,37E+08	2,73E+09	2,81E+09	2,17E+08	3,01E+08	2,59E+08
G1SY87	2,16E+08	0	0	7,31E+08	7,71E+08	7,12E+08
G1SY88	0	1,34E+08	1,94E+08	0	0	0
P35814;G1SY94	0	0	3,46E+08	0	0	3,29E+08
G1SY96	4,66E+09	1,3E+09	1,06E+09	3,16E+09	2,52E+09	1,71E+09
G1SYA5;G1U4K0	1,25E+09	8,34E+09	9,23E+09	3,04E+09	4,05E+09	3,36E+09
G1SYB4;G1U978	8,4E+09	4,86E+09	5,13E+09	1,1E+10	8,95E+09	8,14E+09
G1SYB9	8,71E+09	3,24E+09	3,38E+09	4,13E+09	3,38E+09	4,45E+09
G1SYC1	6,02E+09	5,88E+08	6,02E+08	4,33E+09	3,45E+09	2,62E+09
G1SYC5	1,97E+08	0	0	0	0	0
G1SYC6	2,22E+08	0	0	1,35E+09	1,83E+09	1,98E+09
G1SYC9	2,51E+08	0	0	2,26E+08	0	0
G1SYD2	0	0	0	0	3,81E+08	0
G1SYD3	6,99E+08	7,25E+09	6,36E+09	1,87E+08	2,27E+08	5,33E+08
G1SYD6;U3KNJ6	1,51E+11	5,98E+10	6,72E+10	1,07E+11	9,42E+10	1,21E+11
G1SYE0	3,08E+08	1,37E+09	4,37E+08	2,47E+08	3,09E+08	2,9E+08
G1SYE3	2,28E+08	0	0	4,36E+08	4,61E+08	3,43E+08
G1SYE7	2,19E+09	1,13E+09	1,51E+09	1,05E+09	1,15E+09	2,03E+09
G1SYG0	1,58E+08	0	0	0	0	0
G1SYH5	2,53E+09	4,38E+08	4,07E+08	2,58E+09	3,92E+09	4,28E+09
G1SYI1	1,24E+08	0	0	0	0	0
G1SYI2;G1T4D5	3,45E+10	1,32E+10	1,16E+10	1,46E+10	2,33E+10	1,61E+10
G1SYI3	6,67E+08	3,83E+08	3,6E+08	1,12E+09	5,61E+08	4,87E+08
G1SYI6	2,57E+08	6,37E+08	5,49E+08	4,06E+08	2,51E+08	0
G1SYJ4;U3KN85	7,79E+10	3,10E+11	3,12E+11	9,01E+10	1,51E+11	1,05E+11
G1SYJ6;G1SZF4;G1SZ30	1,2E+10	3,2E+10	3,23E+10	5,27E+09	6,36E+09	4,07E+09
G1SYK0;G1TRJ6;G1TVT8	1,56E+08	1,46E+08	1,52E+08	1,61E+08	1,58E+08	1,5E+08
G1SYK3	0	2,05E+08	2,59E+08	0	0	0

G1SYL7	0	98573000	0	0	0	0
G1SYL8	1,01E+09	2,93E+09	3,39E+09	1,18E+09	1,63E+09	1,43E+09
G1SYL9	9,01E+08	1,5E+09	2,11E+09	1,6E+09	2,65E+09	2,76E+09
G1SYM3	5,27E+09	2,14E+09	2,02E+09	4,31E+09	9,01E+09	5,92E+09
G1SYN3	2,32E+08	1,2E+09	1,16E+09	2,9E+08	3,58E+08	3,49E+08
G1SYP7	0	0	8,7E+08	0	0	0
G1SYQ5;G1U879	0	0	1,39E+08	0	0	0
G1SYR2	1,86E+09	3,49E+08	3,46E+08	4,36E+08	3,85E+08	3,74E+08
Q28690;G1SYR5	0	1,72E+08	1,59E+08	0	0	0
G1SYR9	3,95E+10	1,25E+10	1,14E+10	2,97E+10	2,88E+10	3E+10
G1SYS4	0	5,17E+08	5,36E+08	0	0	0
G1SYS5	1,06E+10	8,13E+09	7,35E+09	1,67E+10	1,61E+10	1,82E+10
G1SYS9	3,45E+08	0	0	3,21E+08	2,68E+08	2,12E+08
G1SYT7;U3KMB7	1,17E+10	3,61E+09	3,21E+09	4,42E+09	4,56E+09	5,34E+09
G1SYU7	5,36E+08	4,61E+09	3,92E+09	6,99E+08	6,95E+08	7,75E+08
G1SYV0	6,15E+09	9,35E+09	1,01E+10	4,09E+09	3,8E+09	4,27E+09
G1SYV9	1,4E+10	3,74E+10	4,38E+10	6,24E+09	7,34E+09	7,06E+09
G1SYW2	1,25E+08	1,21E+08	0	3,91E+08	1,57E+08	98331000
G1SYX4;Q95220	1,28E+08	0	0	0	0	0
G1SYX7	0	8,77E+08	6,52E+08	1,24E+09	2,72E+09	1,5E+09
G1SYY0	88457000	0	0	0	0	0
G1SYY1	4,45E+08	0	0	4,99E+08	0	0
G1SYY4	4E+08	6,24E+08	6,16E+08	0	0	0
G1SYZ6	9,68E+08	3,47E+08	3,08E+08	5,06E+08	5,46E+08	5,09E+08
G1SZ00	1,04E+09	1,33E+10	1,5E+10	1,2E+09	1,36E+09	1,06E+09
G1SZ03	2,44E+09	1E+10	1,24E+10	6,83E+08	4,91E+08	4,2E+08
G1SZ12;U3KNW6	3,95E+09	1,65E+10	1,49E+10	5,24E+09	4,31E+09	3,03E+09
G1SZ14	4,05E+09	7,53E+09	8,04E+09	2,44E+09	2,7E+09	2,76E+09
G1SZ15	6,94E+09	3,65E+09	3,79E+09	2,91E+09	2,69E+09	3,9E+09
G1SZ16	1,97E+08	0	1,52E+08	1,44E+08	2,91E+08	2,62E+08
G1SZ18;G1SZH9;G1TD43	9,39E+08	8,17E+08	5,34E+08	1,33E+09	1,75E+09	1,46E+09
G1SZ19	5,5E+08	0	0	0	0	0
G1SZ23	1,29E+10	2,88E+09	2,68E+09	4,22E+09	3,85E+09	4,16E+09
G1TQR4;G1SZ25	0	7,22E+08	1,3E+09	2,85E+08	3,02E+08	3,84E+08
G1SZ37	5,6E+09	1,66E+09	1,68E+09	5,92E+09	6,96E+09	9,11E+09
G1SZ44	1,08E+11	4,17E+10	4,86E+10	6,55E+10	7,26E+10	9,29E+10
G1U046;G1SZ47;G1TZC6	1,3E+09	1,23E+10	1,2E+10	2,96E+09	3,08E+09	2,43E+09
P29562;G1SZ59;U3KMP9	1,95E+10	9,87E+10	9,86E+10	5,68E+09	6,41E+09	5,97E+09
G1SZ63	3,56E+09	3,55E+09	3,04E+09	4,84E+09	4,24E+09	3,13E+09
G1SZ64	1,21E+09	0	5,7E+08	0	0	0
G1SZ66	1,09E+09	2,07E+08	1,96E+08	1,04E+09	9,41E+08	5,86E+08
G1SZ72	0	5,64E+08	4,77E+08	4,92E+08	4,39E+08	4,66E+08
U3KNV8;G1SZ76;Q04504	2,27E+09	7,77E+09	8,74E+09	3,35E+09	3,53E+09	4,14E+09
G1SZ85	2,93E+08	2,3E+09	2,26E+09	4,73E+08	5,01E+08	3,73E+08
G1SZ91	4,91E+09	1,85E+10	2,25E+10	1,32E+09	1,18E+09	1,06E+09
G1SZ93	7,66E+09	1,83E+09	1,77E+09	3,87E+09	2,83E+09	2,16E+09
G1SZ94;G1SVK2	0	7,21E+08	6,57E+08	0	0	0
G1SZA1;G1TTZ3	5,5E+08	3,99E+08	5,3E+08	1,4E+09	7,41E+08	2,13E+09
G1SZB1	4,45E+08	0	0	3,08E+08	3,6E+08	2,83E+08
G1SZC5;G1TUU1;G1SM80	9,36E+08	2,49E+09	2,41E+09	2,79E+09	2,61E+09	4,06E+09

G1SZD2	1,97E+09	3,83E+08	4,03E+08	4,6E+08	4,95E+08	7,51E+08
G1SZD6;Q6Q6X0	6,98E+09	2,95E+10	3,06E+10	3,7E+09	5,56E+09	5,99E+09
G1SZE0	4,93E+08	3,11E+09	3,28E+09	3,8E+08	4,67E+08	2,14E+08
G1SZE1	0	0	0	0	0	1,37E+08
G1SZE4;G1SXY0;G1SZL0	6,99E+08	7,27E+08	7,67E+08	2,84E+09	1,73E+09	2,41E+09
G1SZE8	1,14E+08	0	0	0	0	0
G1SZF5	0	0	0	3,76E+08	3,12E+08	5,05E+08
G1SZF7	2,8E+10	6,08E+09	5,99E+09	1,93E+10	1,85E+10	3,01E+10
G1SZF9	4,93E+09	1,18E+09	1,18E+09	4,43E+09	4,99E+09	5,86E+09
G1SZG3	7,1E+08	2,33E+08	2,2E+08	1,17E+09	7,01E+08	4,88E+08
G1TMS0;G1SZG4	52574000	0	0	0	0	0
G1SZG7	1,58E+08	3,48E+08	3,33E+08	1,04E+08	0	1,13E+08
G1SZH6	1,2E+08	0	0	2,02E+08	2,12E+08	0
G1SZI0	1,52E+08	0	0	0	0	0
G1SZI7	1,65E+08	0	0	0	0	0
G1SZJ5	6,19E+08	3,99E+09	4,58E+09	5,14E+08	6,46E+08	8,08E+08
G1SZK4	3,74E+08	1,62E+09	1,57E+09	2,58E+08	3,33E+08	1,97E+08
G1SZK8	0	1,76E+08	1,85E+08	0	0	0
G1SZL3	2,19E+08	2,41E+08	2,08E+08	1,46E+09	1,17E+09	2,41E+09
G1SZL5	0	0	51553000	0	0	0
G1SZM2;G1SYG6	1,54E+09	4,66E+08	4,96E+08	3,02E+09	2,65E+09	2,38E+09
G1SZN0	5,78E+08	3,44E+08	3,95E+08	6,16E+08	4,26E+08	3,7E+08
G1SZN2	4,07E+09	5,06E+08	6,59E+08	1,68E+09	1,53E+09	1,64E+09
G1SZP0;U3KM53	2,5E+08	3,97E+08	3E+08	1,54E+08	2,21E+08	2,76E+08
G1SZP6	7,49E+10	9,28E+09	8,98E+09	3,4E+08	3,52E+09	5,65E+09
G1SZQ1	4,66E+08	2,16E+09	6,68E+09	4,08E+08	6,17E+08	4,65E+08
G1SZQ7	1,2E+09	2,41E+09	2,03E+09	3,65E+08	3,76E+08	2,7E+08
G1SZR4	0	3,49E+08	0	0	0	3,57E+08
G1SZR7	5,57E+09	1,58E+10	1,5E+10	7,25E+10	6,85E+10	6,54E+10
G1SZR8;G1T2N0	9,08E+08	6,4E+09	7,16E+09	4,38E+09	8,19E+09	8,19E+09
G1SZS2	0	0	0	3,46E+08	2,15E+08	3,01E+08
G1SZT0	0	0	0	50303000	0	0
G1SZT8	3,32E+09	5,42E+09	6,32E+09	1,63E+09	2,25E+09	3,32E+09
G1SZW0;G1T1Z6	4,43E+08	6,35E+08	5,17E+08	0	0	0
G1SZW8	0	1,81E+08	1,53E+08	0	0	0
G1SZX4	0	0	0	0	0	5,32E+08
G1SZZ2	2,16E+08	1,01E+09	8,08E+08	2,54E+08	2,53E+08	2,01E+08
G1T004	1,48E+09	7,34E+09	3,71E+09	0	2,02E+09	1,48E+09
G1T006	3,65E+09	2,29E+09	2,8E+09	1,07E+09	1,11E+09	1,91E+09
G1T011	4,7E+08	5,25E+08	5,61E+08	3,9E+08	0	0
G1T024	3,43E+08	2,33E+09	3,24E+09	5,62E+08	5E+08	3,01E+08
G1T026;G1TLN3	0	8,42E+08	7,59E+08	3,65E+08	4,03E+08	4,93E+08
G1T029	1,59E+08	0	0	0	0	0
G1T044	2,24E+08	0	0	0	0	0
G1T049	0	0	0	1,43E+09	1,42E+09	1,48E+09
G1T057	0	55349000	0	0	0	0
G1T060	1,37E+10	3,47E+09	3,26E+09	9,11E+09	7,7E+09	6,32E+09
G1T069	6,16E+08	0	2,34E+08	1,34E+08	1,12E+08	0
G1T072;G1U2N8	2,57E+08	3,54E+08	2,98E+08	2,18E+09	2,1E+09	4,36E+09
G1T078	0	2E+08	1,61E+08	0	0	0

U3KM69;G1T089	0	3,72E+08	3,75E+08	0	0	0
G1TUD0;G1T091	0	0	0	0	41859000	0
G1T096	2,41E+09	2,09E+09	2,41E+09	2,14E+09	3,6E+09	3,79E+09
G1T0B0	0	1,54E+08	0	0	0	0
G1T0B1	4,92E+08	0	0	0	72245000	0
G1T0B4	8,65E+09	2,83E+09	3,03E+09	2,05E+10	1,85E+10	1,8E+10
G1T0C1;G1SF30;G1U432	5,33E+09	1,39E+10	1,47E+10	5,71E+09	4,01E+09	3,31E+09
G1T0C2	2,78E+08	0	0	0	0	0
G1T0C5	1,7E+08	4E+08	3,12E+08	0	0	0
U3KNP3;G1T0C8;G1T0F7	7,73E+08	2,83E+08	3,37E+08	0	8,76E+08	2,42E+08
G1T0E3;U3KLU2	2,38E+08	3,17E+08	3,16E+08	1,13E+09	9,01E+08	5,25E+08
G1T0E5	7,79E+08	4,1E+08	2,33E+08	1,06E+09	1,02E+09	1,01E+09
G1T0F3	1E+09	2,12E+08	2,29E+08	1,49E+09	1,45E+09	3,89E+08
G1T0F6	2,57E+08	6,15E+08	3,34E+08	0	0	0
G1T0G0	66737000	0	0	0	0	0
G1T0H0	3,35E+08	1,12E+09	1,01E+09	3,28E+08	4,28E+08	4,22E+08
G1T0H1	2,23E+08	0	0	0	0	0
G1T0H3	3,35E+08	5,8E+08	1,14E+09	2,52E+08	0	0
G1T0H6	0	3,17E+08	0	0	0	0
G1T0H7	2,91E+08	6,17E+08	8,78E+08	2,62E+08	2,93E+08	2,62E+08
G1T0H8;P47859;U3KNG6	6,29E+09	2,53E+10	2,45E+10	6,47E+09	6,07E+09	4,32E+09
G1T0I5	1,8E+10	4,36E+09	4,91E+09	7,9E+09	5,97E+09	5,94E+09
U3KMP8;G1T0J3	4,81E+08	0	1,35E+08	1,45E+08	1,99E+08	0
G1T0J4	1,36E+09	9,81E+08	3,77E+08	1,79E+09	1,13E+09	1,78E+09
G1T0K1	5,81E+09	1,71E+09	1,56E+09	7,02E+09	5,84E+09	3,46E+09
G1T0K2	0	2,47E+08	4,39E+08	0	2,3E+08	2,63E+08
G1T0K5	5,38E+08	0	0	5,67E+08	2,71E+08	3,61E+08
G1T0L0;G1TJ21	0	1,3E+08	0	1,42E+08	0	1,85E+08
G1T0L9	6,91E+10	1,35E+10	1,42E+10	7,03E+10	5,34E+10	3,73E+10
G1T0M0	2,31E+08	64613000	1,17E+08	1,08E+08	1,17E+08	1,04E+08
G1T0M2	5,55E+09	1,58E+09	1,76E+09	2,37E+09	2,31E+09	3,03E+09
G1T0M8;G1TR21;U3KMT8	0	2,27E+09	1,89E+09	3,29E+08	5,14E+08	5,74E+08
G1U1F7;G1TXI1;G1T0N4	4,75E+10	7,8E+10	8,13E+10	9,28E+09	1,32E+10	1,54E+10
G1T0N5	1,94E+09	3,76E+09	3,24E+09	1,03E+10	1,16E+10	1,24E+10
G1T0P3	0	0	2,1E+09	0	0	0
G1T0Q6	0	0	0	1,33E+08	0	0
G1T0R8	0	3,2E+08	2,43E+08	4,11E+08	1,04E+09	8,24E+08
G1T0R9	2,31E+09	8,39E+09	6,71E+09	1,18E+10	1,69E+10	1,81E+10
G1T0S0;G1TNJ0	1,88E+08	2,34E+08	1,4E+08	9,41E+08	6,89E+08	3,58E+08
G1T0T1	2,57E+09	4,84E+08	4,53E+08	2,25E+09	2,72E+09	2,28E+09
G1T0T3	0	0	1,97E+08	0	0	0
G1T0T5	3,58E+08	6,63E+08	6,78E+08	1,03E+09	7,83E+08	8,16E+08
G1T0T6	1,15E+09	3,97E+08	3,53E+08	8,44E+08	7,77E+08	7,51E+08
G1T0U0	0	0	0	0	0	1,09E+08
G1T0U4	6,54E+08	0	0	1,01E+08	0	0
G1T0U5	3,92E+08	4,7E+08	7,69E+08	1,43E+08	1,84E+08	2,26E+08
G1T0U8	3,14E+10	6,15E+09	5,54E+09	4,99E+10	4,11E+10	3,73E+10
G1T0V4;G1TXZ2	2,45E+09	2,73E+09	2,56E+09	4,86E+09	5,37E+09	6,18E+09
G1T0W0	0	4,3E+08	7,08E+08	0	0	0
G1T0W1	1,62E+08	97572000	97072000	2,32E+08	1,42E+08	1,12E+08

G1T0W7	2,77E+09	6,51E+08	5,35E+08	1,24E+09	1,14E+09	1,37E+09
G1T0X6	0	0	0	1,89E+08	0	0
G1T0Y3	4,68E+08	68882000	0	49776000	77818000	63121000
G1T0Y9	2,52E+09	8,08E+09	8,61E+09	1,51E+09	1,96E+09	1,95E+09
G1T0Z8	3,92E+08	1,46E+09	1,82E+09	3,03E+08	2,47E+08	2,45E+08
G1T103	0	1,1E+09	8,7E+08	2,95E+08	5,2E+08	5,59E+08
G1T108	1,2E+09	2,81E+08	4,14E+08	3,52E+08	2,82E+08	3,74E+08
G1T109;U3KPJ1;G1ST36	0	1,86E+08	1,07E+08	94256000	1,25E+08	0
G1T112;O46651	6,97E+09	2,06E+09	1,93E+09	8,81E+09	1,14E+10	1,31E+10
G1T116	1,5E+09	3,29E+08	2,58E+08	2,52E+08	2,41E+08	2,25E+08
G1T120	2,88E+09	4,33E+08	4,22E+08	1,42E+09	1,23E+09	9,32E+08
G1T125	1,56E+08	1,8E+08	0	1,85E+08	2,21E+08	2,89E+08
G1T134	7,57E+08	0	0	2,28E+08	2,56E+08	3,1E+08
G1T135	0	0	0	0	0	4,01E+08
G1T139	7,1E+09	1,94E+09	2,28E+09	2,57E+09	2,33E+09	2,28E+09
G1T150	3,16E+08	3,43E+08	2,75E+08	3,47E+08	2,93E+08	0
U3KNV1;G1T151	4,28E+08	5,21E+08	4,62E+08	4,18E+08	4,67E+08	3,87E+08
G1T156	1,16E+09	5,44E+08	4,74E+08	6,41E+08	5,32E+08	6,16E+08
G1T159	5,61E+08	1,41E+08	1,73E+08	1,37E+08	1,34E+08	0
G1T161	81873000	0	0	1,38E+08	91288000	0
Q95209;G1T165	2,21E+08	0	0	0	2,78E+08	3,81E+08
G1T169	1,77E+08	3,09E+08	2,9E+08	2,42E+08	3,78E+08	3,78E+08
G1T176	2,02E+08	4,11E+08	2E+08	7,85E+08	1,08E+09	1,73E+09
G1T182	1,03E+09	1,25E+09	1,26E+09	3,56E+09	4,42E+09	4,44E+09
G1T188	6,65E+09	1,73E+09	1,59E+09	6,55E+09	7,97E+09	6,67E+09
G1T194	3,34E+08	1,9E+08	1,91E+08	2,42E+08	2,58E+08	4,84E+08
G1T196;G1TT26;G1U3A7	4,87E+08	1,37E+09	7,76E+08	5,75E+08	5,05E+08	8,7E+08
G1T197	1,48E+08	0	0	0	0	0
G1T1B7	1,63E+08	2,54E+08	3,61E+08	1,96E+08	3,29E+08	3,14E+08
G1TQ59;G1T1C4	8,12E+08	0	2,02E+08	2,69E+08	2,35E+08	1,67E+08
G1T1C5	0	1,37E+08	80750000	0	55619000	0
G1T1C6	1,65E+09	0	5,48E+08	0	0	0
G1T1D9	0	5,57E+08	8,23E+08	4,91E+08	4,65E+08	3,94E+08
G1T1E6	1,62E+08	5,25E+08	2,91E+08	0	0	0
G1U472;G1T1F0;G1TLY4	4,2E+09	1,49E+10	1,6E+10	4,36E+09	4,89E+09	4,6E+09
G1T1G0	1,49E+09	3,15E+08	2,65E+08	4,49E+08	3,49E+08	7,86E+08
G1T1G2;U3KPJ6	6E+09	9,59E+08	7,6E+08	5,53E+08	6,55E+08	2,99E+08
U3KLU6;G1T1G4	1,39E+08	3,74E+08	3E+08	74282000	0	0
G1U586;G1T1G5;G1SJD2	2,91E+08	5,08E+08	2,28E+08	0	1,26E+08	1,41E+08
G1T1K0	4,26E+08	0	3,06E+08	7,49E+08	8,98E+08	3,34E+08
G1T1L1	1,06E+09	3,22E+08	2,24E+08	1,72E+09	1,29E+09	5,23E+08
G1T1L4	9,31E+08	1,11E+09	1,54E+09	4,27E+08	3,71E+08	4,18E+08
G1T1L7	89052000	0	0	0	0	0
G1T1P3	5,16E+09	7,07E+08	7,72E+08	1,63E+09	1,84E+09	7,98E+08
G1T1Q2;P62160;G1TV62	9,31E+09	1,26E+10	1,62E+10	3,8E+09	7,23E+09	8E+09
G1T1R0	1,8E+08	5,74E+08	5,04E+08	0	0	0
G1T1S7	1,9E+08	3,1E+09	3,53E+09	3,29E+08	6,24E+08	8,39E+08
G1T1T2	0	8,24E+08	8,27E+08	6,76E+08	8,22E+08	1,3E+09
G1T1T4;U3KMG1	4,09E+09	6,15E+08	8,18E+08	6,01E+09	5,9E+09	4,23E+09
G1T1T8	4,24E+09	8,97E+08	9,19E+08	9,37E+08	1,1E+09	5,66E+08

G1T1U7;G1U5R9	6,15E+08	5,48E+09	3,93E+09	3,13E+08	2,77E+08	1,29E+08
G1T1V9	2,75E+09	5,1E+09	4,37E+09	1,66E+10	1,95E+10	1,99E+10
G1T1W6	7,87E+08	7,48E+08	6,88E+08	1,55E+09	1,63E+09	1,64E+09
G1T1X2	4,52E+08	7,45E+09	7,05E+09	9,79E+08	1,12E+09	1,67E+09
G1T1X7	0	0	2,76E+08	4,51E+08	2,46E+08	4,52E+08
G1T1Y3;G1U4H2	79083000	0	0	75393000	0	0
G1T217	2,89E+08	8,56E+08	1,63E+09	1,57E+08	1,81E+08	0
G1T221	0	0	0	0	0	2,02E+08
G1T235	5,18E+09	9,49E+09	1,23E+10	3,79E+09	3,34E+09	3,89E+09
G1T237	5,58E+10	1,61E+10	1,7E+10	6,28E+10	5,14E+10	3,19E+10
G1T239	6,04E+09	1,74E+09	1,88E+09	3,3E+09	2,49E+09	1,87E+09
G1T242	1,11E+10	2,63E+09	2,3E+09	1,07E+10	1,4E+10	1,6E+10
G1T243	7,86E+08	4,22E+09	3,45E+09	8,05E+09	1,23E+10	1,25E+10
G1U8R0;G1T252;G1TZJ9	0	3,5E+08	2,33E+08	1,27E+08	0	1,46E+08
G1T268	87424000	0	0	0	0	0
G1T271	2,65E+08	1,08E+08	1,57E+08	0	0	0
G1T275	1,98E+08	6,64E+08	6,34E+08	1,63E+08	2,71E+08	2,63E+08
G1T276	5,84E+09	1,45E+09	1,07E+09	5,94E+09	3,79E+09	2,47E+09
G1T277	8,71E+08	8,99E+08	1,01E+09	2,52E+08	1,99E+08	3,04E+08
G1T284;G1TI10	4,94E+09	1,47E+09	1,59E+09	5,13E+09	3,81E+09	2,54E+09
G1T288	0	0	0	0	0	1,9E+09
G1T295;P04068	5,25E+09	1,16E+09	1,08E+09	1,82E+10	9,85E+09	9,13E+09
G1T297	2,61E+09	3,62E+08	5,16E+08	4,46E+09	4,04E+09	2,8E+09
G1T298	1,56E+08	0	0	0	0	0
G1T2B8	1,35E+09	6,76E+08	6,64E+08	5,22E+09	5,84E+09	7,01E+09
G1TBN0;G1T2C3	1,61E+09	1,07E+10	1,21E+10	9,55E+08	1,47E+09	9,15E+08
G1T2C4	2,87E+09	1,73E+10	1,28E+10	1,01E+09	1,01E+09	1,78E+09
G1T2D5;G1U1I6;G1TU02	8,7E+08	1,26E+09	1,28E+09	0	0	0
G1T2E3	0	0	0	0	0	38236000
G1T2E6	6,59E+09	3,01E+09	2,68E+09	1,11E+10	9,48E+09	1,15E+10
G1T2F2;G1TNN8	3,13E+10	1,05E+10	8,96E+09	3,62E+10	3,04E+10	3,67E+10
G1T2F4	3,77E+08	1,6E+08	0	1,3E+09	1,64E+09	1,22E+09
O97755;G1T2F8	0	0	0	0	2E+08	0
G1U5V2;G1T2G3	9,44E+08	5,22E+08	6,47E+08	8,46E+08	1,04E+09	1,61E+09
G1T2G4;P83268	3,34E+09	7,33E+09	7,9E+09	7,22E+08	9,65E+08	8,95E+08
G1T2G5	4,46E+08	0	0	0	0	0
G1T2H9	0	0	0	9,99E+08	2,43E+08	1,16E+08
G1T2I4	2,45E+09	5,66E+09	5,89E+09	2,67E+09	1,89E+09	1,14E+09
G1T2I5	2,16E+10	4,19E+09	4,28E+09	7,24E+09	7,79E+09	7,26E+09
G1T2I6	9,09E+08	1,82E+08	0	1,94E+09	1,94E+09	2,79E+09
G1T2J0	1,89E+08	1,24E+08	1,3E+08	5,23E+08	2,37E+08	3,1E+08
G1T2J6	9,68E+09	3,04E+09	2,39E+09	1,13E+10	8,35E+09	6,26E+09
G1T2K2	4,46E+08	2,98E+09	3,21E+09	1,99E+08	1,89E+08	0
G1T2K5	3,68E+10	8,79E+09	9,48E+09	5,57E+09	2,89E+09	2,87E+09
G1T2K6	2,09E+09	7,51E+08	6,17E+08	5,51E+09	4,54E+09	3,65E+09
G1T2L1	3,92E+09	6,46E+09	5,62E+09	1,7E+09	1,56E+09	1,26E+09
G1T2L3;G1TLG9	8,85E+08	0	3,7E+08	4,88E+08	0	0
G1T2M0	1,11E+09	6,4E+08	5,61E+08	1,18E+09	1,35E+09	1,11E+09
G1T2M9;P36233	1,56E+10	5,35E+09	4,93E+09	5,34E+10	4,86E+10	5,87E+10
G1T2N1	4,02E+08	1,55E+09	1,76E+09	1,12E+09	1,17E+09	9,68E+08

G1T2N4	2,1E+08	1,18E+08	1,22E+08	1,09E+08	96541000	1,09E+08
G1T2Q8;G1TII5	0	1,26E+09	1,25E+09	1,25E+09	1,13E+09	1,46E+09
G1T2R2	9,05E+08	3,75E+08	4,51E+08	4,59E+09	2,97E+09	1,12E+09
G1T2R3	0	0	1,65E+08	0	0	0
G1T2S0	0	3,06E+09	1,83E+09	0	0	0
G1T2S7	3,24E+09	4,78E+08	5,51E+08	1,17E+09	7,53E+08	1,22E+09
G1T2T9	0	2,19E+08	1,62E+08	5,56E+08	3,32E+08	2,27E+08
G1T2U6	5,84E+08	1,24E+09	1,24E+09	2,39E+09	1,81E+09	1,83E+09
G1T2V0	2,86E+09	8,69E+08	9,61E+08	7,55E+08	1,22E+09	1,32E+09
G1T2V2;G1TIJ8	2,76E+09	8,64E+09	9,6E+09	1,25E+09	1,62E+09	1,4E+09
G1T2V6	1,87E+09	1,47E+09	1,81E+09	1,44E+09	1,95E+09	1,53E+09
G1T2V8;G1TKQ5	7,38E+08	7,96E+08	6,87E+08	5,4E+08	8,85E+08	7,16E+08
G1T2W1	2,19E+08	5,5E+08	8,24E+08	2,01E+08	2,06E+08	1,29E+08
G1T2W4	1,59E+08	0	0	1,53E+08	0	1,89E+08
G1T2Y2;U3KPC4	1,53E+08	2,84E+08	3,55E+08	1,97E+09	1,05E+09	1,4E+09
G1T2Y3;G1SG03;U3KPJ5	1,04E+09	1,27E+09	9,43E+08	1,71E+09	1,53E+09	1,81E+09
G1T2Y5	2,86E+10	1,29E+10	1,34E+10	2,78E+10	3,03E+10	3,74E+10
G1T2Z5;Q28668	4,34E+08	5,95E+08	2,96E+08	1,28E+10	1,05E+10	1,34E+10
G1T2Z8	1,26E+09	6,81E+09	8,66E+09	9,53E+08	1,41E+09	9,71E+08
G1T301;G1TS96	8,03E+09	2,91E+09	2,56E+09	5,86E+09	6,65E+09	6,15E+09
G1T302	4,53E+08	6,22E+08	5,77E+08	3,2E+09	3,23E+09	3,68E+09
G1T310	6,12E+08	0	0	2,76E+09	2,29E+09	1,63E+09
G1T312	1,23E+09	8,45E+08	5,8E+08	2,31E+09	2,34E+09	1,99E+09
G1T326	3,42E+08	0	0	0	1,12E+08	0
G1T329	1,21E+09	1,87E+09	1,38E+09	7,29E+09	6,97E+09	9,1E+09
G1T330	1,54E+08	0	0	0	0	0
G1T332;P12343	1,04E+09	4,7E+09	4,09E+09	2E+09	2,55E+09	2,35E+09
G1T336;G1SVI0	5,5E+09	1,15E+10	1,21E+10	3,63E+09	4,64E+09	3,89E+09
G1T338	2,17E+09	5,75E+08	5,1E+08	5,95E+08	1,2E+09	1,18E+09
G1T346	2,12E+10	2,66E+10	2,61E+10	3,25E+10	3,57E+10	3,41E+10
G1T359	5,71E+10	1,59E+10	1,39E+10	4,7E+10	5,55E+10	5,85E+10
G1T361	2,6E+10	5,92E+09	5,66E+09	1,28E+10	1,3E+10	1,37E+10
G1T362;U3KMK5	1,06E+09	2,54E+08	2,72E+08	8,68E+08	5,13E+08	3,6E+08
G1T364	0	0	0	0	90047000	0
G1T369	0	0	8,82E+08	0	0	0
G1T370	0	0	0	0	0	1,85E+08
G1T373	3,74E+08	4,57E+09	3,24E+09	2,12E+08	3,65E+08	2,07E+08
G1T377	6,54E+08	0	1,54E+08	0	0	2,14E+08
G1T379	1,84E+09	4,17E+08	2,79E+08	2,32E+09	1,44E+09	1,06E+09
G1T387	6,43E+09	1,48E+10	1,47E+10	5,29E+09	5,79E+09	8E+09
G1T398	8,43E+08	4,61E+09	4,82E+09	2,46E+09	3,63E+09	3,95E+09
G1T3A2	1,39E+09	3,41E+08	3,65E+08	1,09E+09	1,12E+09	8,72E+08
G1T3A6	8,96E+09	2,07E+09	1,88E+09	2,85E+09	2,33E+09	1,8E+09
G1T3C3	0	3,98E+08	3,34E+08	0	0	0
G1T3D1	1,14E+10	5,38E+09	5,12E+09	2,3E+10	2,18E+10	2,31E+10
G1TLY5;G1T3D7	2,99E+08	1,67E+09	1,78E+09	8,65E+08	8,34E+08	5,5E+08
G1T3D8;G1TS40;G1TPS1	2,43E+09	9,79E+09	9,64E+09	2,18E+09	1,97E+09	1,57E+09
G1T3E1	0	3,3E+08	3,23E+08	0	0	0
G1T3E2	5E+09	8,63E+08	8,68E+08	4,56E+09	3,42E+09	2,27E+09
G1T3E6	5,49E+08	5,87E+08	5,7E+08	9,55E+08	7,8E+08	5,75E+08

G1T3G8	4,35E+09	1,5E+09	1,52E+09	1,08E+09	1,35E+09	1,41E+09
G1T3H3	1,13E+09	3,83E+08	2,42E+08	1,07E+09	8,03E+08	3,98E+08
G1T3H5	2,9E+08	1,49E+09	1,75E+09	0	0	0
U3KMH8;G1T3I0	0	6,46E+08	4,4E+08	0	0	2,5E+08
G1T3I9	1,09E+09	4,08E+09	4,36E+09	7,86E+08	9,31E+08	9,42E+08
G1T3K3	6,32E+08	2,99E+08	0	7,09E+08	4,55E+08	1,06E+09
G1T3K7	0	0	0	2,61E+08	0	0
G1T3K9	1,84E+08	0	0	4,42E+08	3,06E+08	5,38E+08
G1T3L2	3,55E+08	3,3E+09	3,91E+09	3,32E+08	3,52E+08	3,45E+08
G1T3L5;U3KM54	5,75E+08	5,14E+08	5,72E+08	1,88E+09	1,88E+09	2,23E+09
G1T3L6	4,28E+08	0	0	5,65E+08	6,5E+08	1,36E+09
G1T3M0	0	2,07E+08	0	0	0	0
G1T3M3	1,91E+09	6,12E+08	6,79E+08	1,73E+09	1,33E+09	8,62E+08
G1T3M5	2,06E+08	5,37E+08	3,95E+08	3,95E+08	2,73E+08	4,04E+08
G1T3N1;G1U642;G1U5I3	1,61E+09	3,87E+08	3,09E+08	4,22E+08	0	0
G1T3N8	3,09E+08	1,21E+09	1,35E+09	5,66E+08	4,11E+08	3,48E+08
G1T3P1;U3KNW1	1,88E+09	8,27E+09	9,63E+09	5,23E+08	6,55E+08	4,32E+08
G1T3Q2	5,44E+08	3,82E+08	4,05E+08	3,19E+09	3,17E+09	3,75E+09
G1T3R1	4,68E+09	1,86E+09	2,28E+09	3,13E+09	3,52E+09	5,12E+09
G1T3R5	3,94E+09	5,63E+08	6,86E+08	2,18E+09	1,61E+09	2,61E+09
G1T3S1	6,49E+09	1,23E+10	1,41E+10	5,1E+09	5,28E+09	5,31E+09
G1T3S4;U3KN23	1,01E+09	1,39E+08	1,74E+08	4,89E+08	3,3E+08	8,51E+08
G1T3U1	2,23E+09	1,51E+09	1,22E+09	2,7E+09	3,66E+09	4,7E+09
G1T3U3	2,49E+08	0	0	0	0	0
G1T3U5	0	0	0	4,7E+08	6,13E+08	1,19E+09
G1T3V0	5,01E+09	1,55E+10	1,75E+10	5,18E+09	5,38E+09	5,79E+09
G1T3V1;G1TF21;G1TAT7	0	0	0	0	3,21E+08	0
G1T3V2;G1TRF1;G1TGC4	5,09E+09	1,33E+10	1,23E+10	1,35E+10	1,53E+10	2,04E+10
G1T3V5	5,39E+09	6,04E+09	6,42E+09	1,85E+10	1,88E+10	2E+10
G1T3W1	0	2,46E+08	0	0	0	0
G1T3W6	1,21E+09	1,59E+09	1,71E+09	2,61E+09	2,67E+09	3,04E+09
G1T3W8	0	2,34E+08	3,27E+08	3,19E+08	0	3,42E+08
G1T3W9	6,66E+08	1,89E+08	3,25E+08	1,82E+08	0	0
G1T3Y0	5,87E+09	8,54E+09	1,09E+10	1,21E+09	1,6E+09	1,63E+09
G1T3Y8	3,75E+11	1,41E+11	1,50E+11	1,00E+11	1,24E+11	1,52E+11
G1T3Z2	7,49E+08	6,63E+09	8,54E+09	8,74E+08	1,78E+09	2,02E+09
G1T3Z5	0	1,49E+09	1,39E+09	0	0	0
G1T3Z6	53131000	4,77E+08	2,73E+08	1,75E+08	1,68E+08	1,33E+08
G1T419	2,64E+09	2,36E+08	87505000	4,28E+08	3,86E+08	0
G1T432;G1TX42	8,48E+09	2,67E+10	3,03E+10	3,29E+09	4,36E+09	4,39E+09
G1T433	0	0	0	0	0	42709000
G1T434	2,48E+09	1,65E+09	1,57E+09	5,75E+09	6,83E+09	8,18E+09
G1T435	3,67E+08	0	0	4,34E+08	5E+08	4,62E+08
G1T437	3,47E+08	2,5E+08	0	4,33E+08	4,63E+08	5,64E+08
G1T442	0	3,57E+08	3,94E+08	0	0	0
G1T443	0	0	0	1,99E+08	0	0
G1T444	5,63E+08	1,12E+08	1,28E+08	5,34E+08	6,01E+08	8,01E+08
G1T450	0	2,5E+08	1,31E+08	0	0	0
G1T458	1,85E+08	0	0	0	0	0
G1T461	0	2,4E+09	2,54E+09	6,03E+08	9,35E+08	9,33E+08

G1T464	0	2,75E+09	3,29E+09	0	2,76E+08	0
G1T466	0	0	1,86E+08	1,24E+08	0	0
G1T473	1,09E+09	2,38E+09	2,78E+09	9,21E+08	7,98E+08	9,21E+08
G1T489	4,19E+08	2,22E+09	2,14E+09	6,19E+08	5,72E+08	6,61E+08
G1T495	0	0	1,8E+08	0	0	0
Q08863;G1T4A7;Q08862	0	0	0	2,11E+08	0	0
G1T4C9	1,87E+09	5,23E+08	1,96E+08	1,59E+10	1,46E+10	2,25E+10
G1T4D2	3,18E+10	5,55E+09	5,36E+09	7,62E+09	5,89E+09	5,56E+09
G1T4D3;G1TQB2	6,18E+08	0	89346000	3,27E+08	6,26E+08	5,88E+08
G1T4D8;G1U2R3;G1TPZ2	1,11E+10	3,24E+09	3,28E+09	1,09E+10	1,42E+10	1,65E+10
G1T4E1	3,57E+08	2E+08	0	7,79E+08	4,89E+08	3,64E+08
G1T4E7	1,48E+09	4,41E+08	5,26E+08	2,59E+09	2,12E+09	1,74E+09
G1T4E8	1,06E+09	4,35E+08	3,74E+08	3,59E+08	5,72E+08	9,94E+08
G1T4H3	6,48E+10	2,26E+10	2,24E+10	9,97E+10	9,78E+10	1,46E+11
G1T4I7	0	3,33E+08	3,39E+08	0	0	0
G1T4J4	4,56E+09	2,06E+10	1,88E+10	2,67E+09	3,77E+09	4,15E+09
G1T4K5	2,1E+08	3,55E+09	2,92E+09	5,48E+08	1,1E+09	9,97E+08
G1T4K9	1,3E+09	6,24E+08	7,57E+08	6,04E+08	5,19E+08	5,78E+08
G1T4L4;G1T3G0	2,31E+08	1,02E+08	0	3,2E+08	3,28E+08	1,82E+08
G1T4L8	7,5E+08	3,62E+08	0	0	0	0
G1T4L9	1,46E+09	1,36E+09	1,11E+09	4,09E+09	3,94E+09	4,55E+09
G1T4M1	9,34E+08	4,26E+08	5,2E+08	3,56E+08	3,63E+08	2,68E+08
G1T4M2;G1SEM2	7,97E+08	1,74E+08	1,44E+08	3,1E+08	1,17E+08	1,09E+08
U3KN47;G1T4N4;G1T575	3,15E+08	6,77E+08	8,26E+08	1,85E+08	1,15E+08	0
G1T4P7	0	0	0	0	9547500	0
G1T4P8	8,01E+08	5,03E+09	4,77E+09	1E+09	4,36E+08	4,1E+08
G1T4Q8	2,43E+08	8,35E+08	9,19E+08	1,5E+08	0	0
G1T4Q9	3,01E+09	3,82E+09	3,91E+09	2,16E+09	1,59E+09	1,42E+09
G1T4S1	89052000	0	0	0	0	0
G1T4S5	0	0	0	1,47E+09	7,93E+08	8,17E+08
G1T4T0	0	0	1,61E+08	0	0	0
G1T4T3	6,49E+09	2,26E+09	2,67E+09	6,91E+09	5,43E+09	8,06E+09
G1T4T5	0	2,4E+08	0	7,08E+08	1,06E+09	1,16E+09
G1T4V2	6,15E+09	1,62E+09	1,43E+09	1,18E+10	1,02E+10	6,84E+09
G1T4V9	0	0	7,82E+08	0	0	0
G1T4W0;G1TXV3	3,64E+08	3,81E+08	3,02E+08	0	0	0
G1T4W2	0	1,82E+08	0	0	0	0
G1T4W4	4,47E+08	0	0	5,84E+08	1,53E+09	4,95E+08
G1T4W5	3,6E+08	3,53E+08	2,85E+08	9,83E+08	7,36E+08	6,93E+08
G1T4X8	4,16E+09	3,91E+09	4,1E+09	1,71E+09	1,28E+09	1,79E+09
G1T4Y7	5,8E+08	5,23E+09	5,47E+09	4,26E+08	9,66E+08	8,92E+08
G1T4Z1;G1TD94;G1T5U5;G1SIU4	4,05E+10	1,19E+10	1,41E+10	1,69E+11	1,70E+11	1,95E+11
G1T4Z2	1,2E+09	3,63E+09	3,78E+09	1,49E+09	1,23E+09	7,22E+08
G1T4Z7	0	0	0	0	0	1,36E+08
G1T501	9,21E+08	1,03E+09	9,73E+08	3,81E+09	3,04E+09	2,28E+09
G1T508	2,19E+08	0	0	0	0	0
G1T510	1,09E+09	4,88E+08	4,91E+08	4E+08	5,8E+08	4,67E+08
G1T511	2,03E+09	1,56E+09	1,57E+09	2,19E+09	1,81E+09	2,4E+09
G1T512	1,03E+08	0	0	2,18E+08	0	1,38E+08
G1T519	4,86E+09	8,55E+09	8,17E+09	2,09E+09	2,46E+09	2,27E+09

G1T520	3,04E+08	8,49E+08	9,36E+08	4,3E+08	6,45E+08	4,66E+08
G1T521	3,2E+08	0	0	83963000	80240000	0
G1T524;U3KPH3	1,77E+11	7,42E+10	6,24E+10	1,94E+11	1,59E+11	9,52E+10
G1T530	1,49E+08	1,51E+09	1,46E+09	1,97E+08	2,22E+08	2,48E+08
G1T534	2,87E+08	7,03E+08	6,04E+08	0	0	1,18E+08
G1T536;G1TM49;G1SSJ8	2,73E+09	1,15E+09	1,02E+09	2,17E+09	2E+09	1,97E+09
G1T545	1,08E+09	5,25E+09	5,87E+09	1,43E+09	2,63E+09	2,58E+09
G1T548	1,99E+09	4,91E+08	3,39E+08	1,2E+09	9,82E+08	1,03E+09
G1T550	3,78E+09	1,77E+09	1,91E+09	3,89E+09	3,91E+09	2,51E+09
G1T555	3,67E+09	1,08E+09	8,93E+08	2,26E+09	2,18E+09	2,31E+09
G1T567;G1TTV0;G1TQZ8	1,01E+10	6,83E+09	6,61E+09	1,58E+10	1,58E+10	1,59E+10
G1T568	1,12E+09	2,08E+09	2,41E+09	1,96E+09	1,63E+09	1,33E+09
G1T573	0	0	44419000	0	0	0
G1T578	1,42E+09	4,16E+08	4,55E+08	1,47E+09	1,4E+09	1,5E+09
G1T579	2,37E+08	1,85E+08	0	4,85E+08	0	1,88E+08
G1T580	3,67E+08	2,59E+08	2,29E+08	2,88E+08	4,98E+08	6,23E+08
G1T586;G1TWH7	8,82E+10	5,39E+10	5,38E+10	4,74E+10	4,79E+10	6,31E+10
G1T593	1,02E+09	3,14E+09	3,24E+09	4,68E+08	5,07E+08	4,7E+08
G1T594	2,77E+09	1,05E+09	5,52E+08	1E+10	1,03E+10	1,22E+10
G1T5A0	7,61E+08	7,55E+09	9,71E+09	4,81E+08	3,49E+08	7,28E+08
G1T5A2	3,2E+09	7,79E+08	9,3E+08	5,12E+08	4,54E+08	5,5E+08
G1T5A5	3,09E+08	43956000	43522000	53921000	67294000	43348000
G1T5A9	2,59E+08	0	0	0	0	0
G1T5B0	7,64E+08	2,78E+08	2,81E+08	3,64E+08	3,13E+08	3,13E+08
G1T5C5	9,46E+09	3,93E+09	3,42E+09	2,21E+10	2,41E+10	2,01E+10
G1T5D2	2,14E+08	1,24E+09	1,35E+09	5,15E+08	6,91E+08	6,65E+08
G1T5E2	6,91E+08	1,08E+09	1,19E+09	1,56E+09	2,26E+09	3,24E+09
G1T5E6	1,51E+09	3,63E+09	3,47E+09	1,01E+09	1,67E+09	1,49E+09
G1T5G8	2,46E+08	0	0	98759000	0	0
G1T5H0	0	0	0	5,07E+08	2,83E+08	0
G1T5H2	0	0	0	0	0	3,48E+08
G1T5H5	1,31E+09	3,07E+08	4,2E+08	4,48E+08	1,23E+09	2,96E+08
G1T5H7	3,89E+08	8,07E+08	1,24E+09	0	0	0
G1T5H8	1,64E+09	6,72E+09	8,59E+09	6,52E+08	1,27E+09	1,35E+09
G1T5I3	1,22E+09	3,57E+08	3,59E+08	1,53E+09	1,01E+09	3,99E+08
G1T5I9	4,53E+08	1,86E+09	2,08E+09	3,16E+09	2,8E+09	3,8E+09
G1T5J8	2,29E+08	3,17E+08	4,06E+08	0	0	0
G1T5J9	1,43E+10	2,74E+09	2,87E+09	1,2E+10	9,51E+09	1,09E+10
G1T5L3	1,21E+09	4,1E+08	2,93E+08	1,43E+09	7,19E+08	9,33E+08
G1T5M0	9,76E+10	3,14E+10	3,05E+10	4,76E+10	4,91E+10	5,36E+10
G1T5N5	2,5E+10	6,49E+09	6,79E+09	6,67E+09	6,61E+09	7,96E+09
G1T5N7;U3KM88	5,1E+09	1,55E+09	1,6E+09	1,82E+09	2,58E+09	1,84E+09
G1U207;G1T5Q8	3,2E+09	7,93E+08	6,6E+08	1,96E+09	2,46E+09	2,4E+09
G1T5R3	37961000	59051000	64392000	1,53E+08	40305000	45673000
G1T5R7	1,95E+08	0	0	0	0	0
G1T5S9	2,8E+09	3,44E+08	2,65E+08	4,96E+09	3,41E+09	3,82E+09
G1T5T8	2E+08	8,71E+09	8,58E+09	6,65E+08	5,62E+08	5,52E+08
G1T5U2;P47814	2,36E+08	5,95E+08	1,05E+09	0	2,65E+08	2,34E+08
G1T5V3	7,73E+09	2,83E+09	3,35E+09	1,29E+10	1,12E+10	7,41E+09
G1T5V8	0	3,52E+08	4,09E+08	0	0	0

G1T5W4;U3KM39	7,29E+09	2,41E+09	2,53E+09	4,27E+09	4,18E+09	3,99E+09
G1T5W7	8,49E+08	1,69E+09	1,65E+09	5,39E+08	0	0
G1T5X6;U3KPQ8	2,48E+09	1,21E+09	9,86E+08	1,79E+09	1,95E+09	2,35E+09
G1T5Y1	2,18E+08	7,74E+08	1,04E+09	3,22E+08	3,19E+08	2,92E+08
G1T752;G1T5Z3	0	3,3E+08	3E+08	0	0	0
G1T5Z7;G1T4J7;G1T2T1	2,13E+08	1,1E+09	1,5E+09	3,95E+08	3,37E+08	2,84E+08
G1T601	2,13E+09	1,22E+09	9,42E+08	2,75E+09	2,76E+09	4,11E+09
G1T616	4,87E+08	1,87E+09	1,9E+09	1,15E+09	1,14E+09	1,01E+09
G1U662;G1T617	2,12E+09	9,03E+08	1,08E+09	1,7E+09	1,47E+09	1,55E+09
G1T638;G1TUL0	3,31E+08	0	0	0	0	0
G1T639	0	0	0	4,87E+08	0	0
G1T641	1,2E+09	3,68E+08	0	5,51E+08	3,05E+08	2,8E+08
G1T643	1,27E+08	3,28E+08	2,22E+08	0	0	0
G1T645	1,15E+09	2,14E+08	2,99E+08	1,32E+09	7,91E+08	8,18E+08
G1T647	3,36E+08	9,03E+08	4,24E+08	0	0	0
G1T650	6,53E+08	1,87E+08	0	2,33E+08	2,7E+08	2,31E+08
G1T652;G1U8Q8;P79226	0	9,88E+08	9,68E+08	9,99E+08	5,86E+09	1,9E+09
G1T657;G1U371;G1U208	8,76E+09	4,97E+09	5,54E+09	6,67E+09	7,58E+09	9,1E+09
G1T658	0	0	0	0	0	3,03E+08
G1T659	71728000	1,84E+08	4,05E+08	58572000	0	0
G1T661	4,43E+09	1,08E+10	1,08E+10	3,41E+09	4,22E+09	4,7E+09
G1T667	1,24E+08	3,28E+08	0	0	0	1,57E+08
G1T670	4,55E+08	1,05E+09	1,56E+09	0	5,04E+08	6,26E+08
G1T671	2,42E+09	1,25E+10	9,26E+09	8,24E+08	1,05E+09	8,41E+08
G1T673	1,35E+09	2,63E+08	2,21E+08	2,15E+08	3,33E+08	2,47E+08
G1T678	0	1,39E+09	9,72E+08	0	0	0
G1T688	9,81E+08	1,68E+08	2,18E+08	4,19E+08	4,17E+08	3,54E+08
G1T695	8,04E+09	2,36E+10	2,3E+10	2,55E+09	3,53E+09	3,49E+09
G1T6A3	2,71E+09	1,25E+09	1,04E+09	2,5E+09	3,59E+09	5,14E+09
G1T6B3	2,37E+09	1,15E+10	1,14E+10	9E+08	1,08E+09	8,48E+08
G1T6C0	1,79E+09	8,04E+08	6,72E+08	1,96E+09	1,62E+09	8,46E+08
G1T6D1;G1TPF6	3,41E+09	1,83E+10	1,66E+10	4,51E+09	4,4E+09	4,58E+09
G1T6D4	2,25E+09	2,43E+09	2,59E+09	1,16E+09	8,77E+08	9,14E+08
G1T6E6	72863000	0	0	0	0	0
G1T6E9	8,24E+08	2,06E+08	2,75E+08	1,78E+09	1,88E+09	1,72E+09
G1T6G1	8,78E+08	0	5,22E+08	6,9E+08	5,82E+08	7,4E+08
G1T6I0;P80456	0	92703000	63631000	0	0	0
G1T6I6	4,14E+10	9,84E+09	9,11E+09	2,14E+10	1,62E+10	1,66E+10
G1T6J2;G1U756	1,38E+09	2,9E+08	3,23E+08	1,41E+09	8,67E+08	3,28E+09
G1T6L0	4,11E+08	0	0	0	0	0
G1T6L5	6,88E+08	3,27E+09	3,41E+09	1,5E+09	1,8E+09	1,91E+09
G1T6L7	7,26E+09	3,17E+09	3E+09	8E+09	1,11E+10	1,16E+10
G1T6M0	1,48E+09	5,59E+08	2,94E+08	2,48E+08	1,84E+08	1,42E+08
G1T6M1	5,78E+08	0	0	3,37E+08	3,38E+08	3,79E+08
G1T6M2;G1TWM4	1,24E+09	3,93E+08	4,23E+08	1,65E+09	1,28E+09	8,12E+08
G1T6N0	3,48E+08	0	0	2,86E+08	0	0
G1T6N3	3,17E+09	1,56E+09	1,1E+09	4,26E+08	7,09E+08	7,05E+08
G1T6N7	2,76E+09	7,19E+08	8,22E+08	1,71E+09	2,48E+09	2,64E+09
G1T6P5	1,52E+09	3,72E+08	3,85E+08	5,26E+08	4,18E+08	0
P25915;G1T6Q8	1,46E+10	1,99E+09	2,2E+09	2,5E+08	1,31E+08	1,96E+08

G1T6Q9	2,05E+09	5,92E+08	5,81E+08	2,89E+09	3,94E+09	3,74E+09
G1T6S0;G1SV73	1,41E+08	7,08E+08	8,85E+08	2,58E+08	2,02E+08	2,75E+08
G1T6S2	0	3,64E+08	2,26E+08	1,24E+08	0	1,01E+08
G1T6S6;G1T1E8	1,26E+09	4,6E+08	5,06E+08	3,68E+08	5,81E+08	1,22E+09
G1T6S9;U3KNM6	0	5,59E+08	5,05E+08	2,96E+08	4,72E+08	4,1E+08
G1T6T0	21993000	0	0	0	0	0
G1T6T5	0	4,43E+08	1,21E+09	2,19E+08	0	0
G1T6T7	2,87E+08	0	0	0	0	0
G1T6T8	1,69E+09	9,6E+08	6,3E+08	1,85E+09	1,44E+09	1,12E+09
G1T6U1	4,89E+08	3,01E+08	5,2E+08	2,01E+09	1,23E+09	6,81E+08
G1T6U3	61765000	0	0	99387000	84494000	0
G1T6U4	4,47E+08	67451000	78974000	1,04E+08	71855000	53432000
G1T6U5	0	7,54E+08	8,04E+08	0	0	0
G1T6V7	0	0	0	0	0	56272000
G1T6W3	0	0	0	0	0	3,69E+09
G1T6W4;O97529;U3KNG8	2,62E+08	3,75E+08	6,46E+08	2,19E+08	0	0
G1T6W7	6,84E+09	2,78E+09	2,33E+09	4,68E+09	4,94E+09	4,09E+09
G1T6W9	0	3,04E+08	0	0	0	0
G1T701	5,18E+09	1,04E+09	9,59E+08	3,21E+09	2,9E+09	2,32E+09
G1T703	1,04E+09	0	9,8E+08	9,53E+08	6,52E+08	0
G1T705	6,4E+09	1,21E+09	1,03E+09	4,37E+09	2,4E+09	1,99E+09
G1T714	8,91E+08	2,3E+08	1,5E+08	4,78E+08	4,7E+08	3,58E+08
G1T720	1,59E+09	5,79E+08	5,4E+08	3,63E+09	2,52E+09	1,69E+09
G1T726	2,25E+10	6,36E+09	5,07E+09	1E+10	9,3E+09	8,16E+09
G1T736	0	0	0	1,57E+08	1,73E+08	2,35E+08
G1T748	3,76E+08	1,3E+09	1,34E+09	0	1,35E+08	0
G1T753	0	1,63E+08	0	0	1,02E+08	0
G1T754	0	4,31E+08	0	0	0	0
G1T756	0	0	0	0	5,2E+08	3,83E+08
U3KMH9;G1T765	1,79E+11	4,72E+10	5,38E+10	5,39E+10	5,6E+10	5,43E+10
G1T781;U3KP89	0	0	0	3E+08	2,91E+08	3,19E+08
G1T782	1,33E+09	2,08E+08	2,78E+08	2,27E+08	2,56E+08	1,75E+08
G1T786	0	0	2,08E+08	1,95E+08	2,01E+08	2,05E+08
G1T792	4,66E+08	1,87E+08	1,68E+08	5,91E+08	3,04E+08	2,93E+08
G1T798	3,93E+08	3,36E+08	2,14E+08	9,61E+08	1,21E+09	1,63E+09
G1T7B1	1,51E+08	2,95E+08	3,72E+08	97661000	1,16E+08	1,81E+08
G1T7B6	2,31E+09	7,14E+08	7,18E+08	3,78E+08	0	0
G1T7C0	4,47E+09	1,1E+09	1,13E+09	6,42E+09	8,76E+09	9,35E+09
G1T7C1	1,27E+09	4,86E+08	6,13E+08	7,85E+08	1,03E+09	9,98E+08
G1T7D0	3,22E+08	4,71E+09	4,6E+09	2,4E+09	2,8E+09	2,08E+09
G1T7D1;G1TQ05	3,28E+10	5,14E+10	4,72E+10	6,44E+10	4,97E+10	5,44E+10
G1T7D7	1,01E+09	2,36E+09	7,43E+08	2,2E+09	2,35E+09	2,43E+09
G1T7D9;G1U6L5	0	1,39E+08	0	0	0	0
G1T7E3	4,75E+09	4,69E+09	6,49E+09	0	0	0
G1T7E6	0	0	0	3,82E+08	3,67E+08	5,16E+08
G1U155;G1TP83;G1TJC1	6,83E+09	6,17E+09	5,03E+09	1,28E+10	1,3E+10	1,23E+10
G1TI16;G1T7G2	1,84E+08	0	0	0	1,41E+08	3,18E+08
G1T7G3	3,41E+08	0	1,31E+08	1,39E+08	1,35E+08	1,13E+08
G1T7G6	1,13E+08	1,43E+08	1,57E+08	0	0	0
G1T7H0	7,33E+10	4,4E+10	4,45E+10	6,7E+10	4,93E+10	6,14E+10

G1T7H3	1,46E+09	6,12E+08	5,55E+08	2,64E+09	3,83E+09	4,65E+09
G1T7H4	1,91E+08	0	74292000	0	0	0
G1T7H6	1,25E+08	0	0	1,5E+09	1,69E+09	1,93E+09
G1T7I0	16415000	0	0	0	0	0
G1T7I3	2,08E+09	5,25E+09	7,29E+09	0	1,33E+09	2,41E+09
G1T7I4	2,81E+09	6,64E+09	6,83E+09	4,06E+09	3,42E+09	1,57E+09
G1T7I8	0	1,98E+08	2,12E+08	0	0	0
G1T7J5	3,36E+09	9,27E+08	7E+08	2,39E+09	2,61E+09	3,18E+09
G1T7J9	2,72E+08	93443000	0	4,63E+08	3,59E+08	1,27E+08
G1T7L0	2,59E+09	1,67E+09	2,14E+09	1,67E+09	2,11E+09	1,98E+09
G1T7L5	4,21E+08	2,81E+09	2,03E+09	5,88E+08	6,83E+08	6,23E+08
G1T7L6	91292000	0	0	0	0	0
G1T7L7	2,43E+08	0	87841000	3,38E+08	1,58E+08	1,93E+08
G1T7L9;G1U4R9	3,34E+08	1,11E+09	1,42E+09	0	0	0
G1TWJ2;G1T7M0	0	0	0	0	0	52541000
G1T7M3	0	5,5E+08	5,54E+08	0	0	0
G1T7P9	9,08E+08	4,49E+08	3,55E+08	1,07E+09	5,75E+08	8,38E+08
G1T7Q2	1,52E+09	5,89E+08	5,63E+08	1,12E+09	8,33E+08	1,1E+09
G1T7Q3	7,54E+08	3,45E+08	0	4,11E+08	3,5E+08	3,02E+08
G1T7R2;G1TF91	1,54E+10	6,44E+10	6,8E+10	1,56E+10	2,23E+10	2,2E+10
G1T7R4	1,95E+09	7,48E+08	1,07E+09	1,13E+10	1,01E+10	1,06E+10
G1T7S0	3,42E+09	1,05E+10	1,33E+10	5,79E+09	7,21E+09	6,89E+09
G1T7S1	7,35E+08	1,06E+09	1,36E+09	5,98E+08	6,51E+08	4,89E+08
G1T7S4	3,82E+08	0	6,03E+08	7,02E+08	4,82E+08	0
G1T7S5	2E+08	0	0	4,46E+08	4,89E+08	1,34E+08
G1T7S6	0	0	0	90432000	0	0
G1T7T0	3,95E+08	2,86E+08	2,57E+08	3,3E+08	2,38E+08	4,71E+08
G1T7T2	1,75E+09	2,73E+08	1,32E+08	3,97E+08	1,12E+09	1,13E+09
G1T7T6	2,45E+08	9,96E+08	7,41E+08	3,45E+08	3,13E+08	0
G1T7T8;Q9TT38	3,55E+10	3,16E+10	3,57E+10	1,54E+10	1,86E+10	1,43E+10
U3KM36;G1T7U4;G1SV38	1,44E+08	0	3,97E+08	0	0	0
G1T7U6	7,28E+09	1,5E+09	1,83E+09	6,11E+09	4,9E+09	5,21E+09
G1T7U7	3,15E+08	0	0	4,66E+08	3,66E+08	3,14E+08
G1T7V5	3,86E+10	1,21E+10	1,26E+10	2,29E+10	2,68E+10	3,17E+10
G1T7W2	0	0	9,62E+08	4,27E+08	6,68E+08	1,09E+09
G1T7W7	7E+09	2,17E+09	2,51E+09	8,52E+09	6,01E+09	4E+09
G1T7X9	1,07E+09	0	2,63E+08	7,77E+08	3,32E+08	3,05E+08
G1T7Y5	1,06E+09	5E+08	5,07E+08	8,38E+08	4,87E+08	4,13E+08
G1T7Y7	6,15E+09	7,34E+09	7,54E+09	2,28E+09	2,83E+09	2,71E+09
G1T7Z0	2,27E+09	6,88E+09	6,82E+09	2,54E+09	2,36E+09	1,93E+09
G1T7Z2;G1SK19	1,34E+09	3,13E+08	1,85E+08	1,22E+08	0	0
G1T7Z6	1,79E+10	7,87E+10	8,21E+10	4,39E+10	5,83E+10	4,15E+10
G1T810;G1SHS4	0	1,42E+08	87595000	0	0	0
G1T813;G1SHS1	3,09E+10	9,71E+09	8,26E+09	2,74E+10	3,22E+10	3,13E+10
G1T822;U3KP78;G1T0N9	0	5,63E+08	4,34E+08	0	0	65996000
G1T823	2,3E+08	2,7E+09	2,52E+09	2,04E+08	2,1E+08	1,03E+08
G1T824	3,64E+09	1,05E+10	1E+10	8,39E+09	9,86E+09	8,69E+09
G1T840	1,7E+09	3,74E+09	3,69E+09	7,07E+08	7,09E+08	6,63E+08
G1T845	4,74E+09	1,29E+09	1,46E+09	3,96E+09	2,86E+09	1,64E+09
G1T846	2,29E+09	5,19E+09	5,31E+09	1,5E+09	1,88E+09	1,38E+09

G1T847;G1SFA6;G1SSH5	1,04E+09	0	0	0	0	0
G1T850	0	1,33E+08	60945000	0	0	0
G1T852	0	0	0	3,52E+08	3,43E+08	3,75E+08
G1T853	0	0	0	0	0	0
G1T855;G1TLR2	2,96E+09	5,87E+08	6,89E+08	3,61E+09	3,87E+09	4,64E+09
G1T863	0	0	0	0	4,34E+09	0
G1T866	3,7E+09	1,02E+09	7,26E+08	4,2E+09	3,12E+09	3,34E+09
G1T868	1,23E+09	1,9E+09	2,1E+09	1,46E+09	2,24E+09	1,94E+09
G1T881	2,36E+08	5,52E+08	3,95E+08	3,59E+08	0	0
G1T887	1,01E+09	3,66E+08	3,06E+08	5,6E+08	4,93E+08	6,61E+08
G1T888	0	5,95E+08	5,05E+08	0	0	0
G1T890	5,42E+09	1,06E+09	1,11E+09	1,86E+09	1,4E+09	7,81E+08
G1T894	1,97E+08	7,33E+08	8,18E+08	1,84E+08	2,48E+08	2,19E+08
G1T897	2,54E+08	0	0	0	2,9E+08	0
G1T8A2;G1U1Y5	1,62E+08	4,05E+09	4,55E+09	1,19E+09	1,8E+09	8,46E+08
G1T8A6	1,03E+09	2,32E+08	1,91E+08	2,82E+08	2,3E+08	0
G1T8A7	3,41E+09	1,07E+09	8,79E+08	8,99E+08	1,15E+09	8,6E+08
G1T8B3	4,44E+08	1,22E+08	0	1,78E+08	1,33E+08	1,47E+08
G1T8B5;G1TJP8	0	1,17E+09	1,28E+09	2,87E+09	4,03E+09	4,43E+09
G1T8C2	0	0	0	0	0	3,1E+08
G1T8C8	3,92E+08	1,69E+09	1,71E+09	4,62E+08	7,08E+08	6,11E+08
G1T8D4;U3KNE3	1,36E+09	4,37E+09	4,56E+09	1,51E+09	1,6E+09	1,34E+09
G1T8D7;G1T0A9	1,63E+08	0	0	5,2E+08	2,04E+08	2,56E+08
G1T8E0	2,99E+09	9,73E+08	8,94E+08	1,07E+09	8,88E+08	9E+08
G1T8E2;G1U0J7	0	0	62887000	0	0	0
G1T8E7	2,7E+08	8,48E+08	8,14E+08	8,29E+08	5,45E+08	3,56E+08
G1T8E8	0	0	2,71E+08	0	0	0
G1T8F7	3,41E+09	6,54E+08	5,66E+08	6,98E+08	6,19E+08	6,09E+08
G1T8F8;G1TFL6	2,64E+09	4,03E+09	3,67E+09	6,36E+08	7,14E+08	6,85E+08
G1T8G4	0	0	0	0	56170000	0
G1T8H1	8,38E+09	2,73E+09	3,02E+09	1,06E+10	7,2E+09	4,22E+09
G1T8H3	2,27E+08	2,48E+09	2,79E+09	2,07E+08	3,5E+08	3,34E+08
G1T8H5	5,18E+08	1,18E+09	1,19E+09	8,78E+08	9,74E+08	1,28E+09
P84246;G1TNB7;G1TJR7	8,2E+08	2,55E+09	1,91E+09	3,26E+09	4,18E+09	3,73E+09
G1T8H8	1,31E+09	3,56E+08	3,16E+08	0	1,52E+08	0
G1T8I9	0	0	0	1,2E+09	6,65E+08	7,78E+08
G1T8J0	1,24E+09	3,11E+08	0	1,99E+08	0	0
G1T8K1	2,91E+09	5,69E+08	6,99E+08	1,39E+09	9,36E+08	1,03E+09
G1T8K2	1,13E+08	0	0	0	0	0
G1T8L1	0	0	0	1,47E+08	0	0
G1T8L2	2,87E+09	2E+09	1,58E+09	8,83E+09	1,09E+10	1,37E+10
G1T8M6	73324000	1,75E+08	2,17E+08	0	0	0
G1T8M9	8,07E+10	1,42E+10	1,48E+10	2,67E+10	1,92E+10	1,68E+10
G1T8N7	2,06E+08	6,08E+08	9,51E+08	2,73E+08	2,99E+08	3,54E+08
G1T8N8	3,39E+08	0	0	0	0	0
G1T8P1	6,55E+08	0	1,32E+08	1,55E+08	0	1,15E+08
G1T8P3	4,59E+09	3,44E+09	3,08E+09	5,94E+09	7,04E+09	7,14E+09
G1T8P4	1,72E+10	3,43E+10	4,05E+10	1,32E+10	1,63E+10	2,38E+10
G1T8P7	0	8,47E+08	8,5E+08	0	0	0
G1T8R1;G1U3R6	1,09E+09	4,45E+09	3,98E+09	6,05E+08	1,11E+09	7,31E+08

G1T8R2;G1T050	8,78E+08	3,14E+08	0	9,13E+08	7,23E+08	4,23E+08
G1T8S4	0	0	1,84E+08	0	0	0
G1T8S7	1,43E+09	4,16E+08	3,72E+08	1,44E+09	1,32E+09	1,6E+09
G1T8S8;G1TTH3	0	0	0	0	0	66863000
G1T8T9	0	0	0	99899000	0	0
G1T8V6	0	1,14E+08	1,6E+08	0	0	0
G1T8V9	0	1,69E+08	0	0	0	0
G1T8Y0	3,23E+08	0	0	1,08E+08	0	0
G1T8Z0	6,09E+09	3,37E+10	3,13E+10	1,68E+10	2,33E+10	1,94E+10
G1T916;G1TXR9	8,55E+08	2,27E+09	2,28E+09	1,77E+08	2,63E+08	6,5E+08
G1T918	3,07E+09	4,8E+09	4,66E+09	1,48E+09	1,69E+09	1,88E+09
G1U4M1;G1T920	1,5E+08	2,01E+09	1,77E+09	6,05E+08	6,53E+08	7,04E+08
G1T923	1,23E+09	3,52E+08	2,69E+08	3,31E+08	2,94E+08	3,34E+08
G1T925	2,42E+09	5,8E+08	6,98E+08	4,12E+09	3,13E+09	3,8E+09
G1T926	7,75E+08	2,93E+09	3,34E+09	9,29E+08	1,96E+08	1,96E+08
G1T932;P79371	3,04E+08	1,75E+08	0	1,12E+09	5,13E+08	4,05E+08
G1T950	0	0	0	0	3,15E+08	0
G1T964	1,51E+09	3,11E+08	4,13E+08	2,88E+09	2,89E+09	4,31E+09
G1T970	7,23E+09	1,97E+09	1,95E+09	2,74E+09	2,27E+09	2,78E+09
G1T974	2,8E+09	5,16E+08	5,86E+08	2,52E+09	1,46E+09	1,08E+09
G1T980	3,77E+09	9,75E+08	1,17E+09	1,75E+09	1,93E+09	1,91E+09
G1T983	1,98E+09	3,18E+09	2,93E+09	1,93E+09	2,67E+09	3,42E+09
G1T994;Q28902	1,04E+11	4,89E+09	5,14E+09	1,07E+11	6,76E+10	6,74E+10
G1T9B1	6,09E+08	0	0	2,84E+08	2,67E+08	2,72E+08
G1T9B9	0	0	0	0	0	6,97E+08
G1T9C1	2,08E+08	0	0	1,51E+08	1,14E+08	0
G1T9C8	3,99E+08	2,52E+08	2,9E+08	1,07E+09	1,41E+09	1,41E+09
G1T9D6	0	0	3,39E+08	0	0	0
G1T9D7	8,32E+08	2,95E+08	2,42E+08	2,15E+08	2,57E+08	2,44E+08
G1T9E6	0	0	0	1,81E+08	1,71E+08	0
G1T9F3	6,59E+09	2,07E+10	2,07E+10	4,19E+09	3,78E+09	3,24E+09
G1T9F6	3,2E+09	5,22E+08	4,2E+08	4,62E+09	4,24E+09	3,78E+09
G1T9F8	7,26E+09	5,46E+09	5,7E+09	6,63E+08	3,98E+08	3,7E+08
G1T9G4	6,22E+08	1,41E+09	1,29E+09	6,66E+08	9,33E+08	1,13E+09
G1T9G8	0	0	1,33E+08	0	0	0
G1T9H0	0	3,89E+08	5,02E+08	0	0	0
G1T9H4	3,04E+08	97183000	1E+08	1,5E+08	1,21E+08	94676000
G1T9I3	0	4,99E+08	7,32E+08	0	0	0
G1T9I4	3,42E+08	9,75E+08	1,19E+09	4,9E+08	7E+08	7,62E+08
G1T9I9	0	3,72E+08	4,27E+08	0	0	0
G1T9J3	1,83E+09	1,54E+09	6,4E+08	3,15E+09	1,47E+09	1,03E+09
G1T9J4;G1SJD1	0	0	0	1,77E+08	0	0
G1T9L6	1,94E+09	6,51E+08	7,31E+08	3,96E+09	3,36E+09	4,71E+09
G1T9M7	0	1,82E+09	1,17E+09	0	0	0
G1T9M9	1,18E+11	2,53E+11	2,96E+11	1,15E+11	1,32E+11	1,43E+11
G1T9N2	6,52E+10	1,79E+10	1,83E+10	5,5E+10	6,51E+10	8,1E+10
G1T9N3	2,93E+08	1,18E+09	1,62E+09	1,96E+08	2,31E+08	1,3E+08
G1TMS2;G1T9P1	0	1,67E+08	0	0	0	0
G1T9P2	1,79E+09	1,49E+09	1,63E+09	2,22E+09	2,08E+09	2,38E+09
G1T9P8;G1TP78	0	2,79E+09	2,09E+09	1,92E+08	1,81E+08	1,41E+08

G1T9Q1	0	99704000	1,13E+08	2,84E+08	3,96E+08	3,76E+08
G1T9R5	0	2,31E+08	0	1,08E+09	1,19E+09	3,99E+09
G1T9R8	0	3,71E+08	3,55E+08	0	0	0
G1T9S4	1,87E+10	5,04E+09	4,4E+09	1,35E+10	1,35E+10	1,45E+10
G1T9T5	2,59E+10	7,94E+09	7,53E+09	5,8E+09	7,06E+09	7,48E+09
G1T9T8	0	0	2,01E+08	0	0	0
G1T9U1	4,12E+08	0	1,08E+08	0	0	0
G1T9U5;U3KM43	4,55E+08	1,37E+08	2,69E+08	8,53E+09	5,18E+09	2,28E+09
G1T9V1	3,27E+10	9,76E+09	9,85E+09	3,21E+10	3,36E+10	4,35E+10
G1T9V2	3,69E+08	3,18E+08	3,82E+08	2,65E+09	2,63E+09	2,72E+09
G1T9V4	6,71E+09	1,4E+10	1,47E+10	4,05E+09	4,94E+09	4,49E+09
G1T9W3	2,22E+08	3,66E+08	4,5E+08	7,52E+08	5,97E+08	3,91E+08
G1T9W8	0	2,61E+08	1,9E+08	0	0	0
G1T9W9;U3KM57	0	3,14E+08	0	0	0	0
G1T9X4	0	0	0	6,65E+08	4,17E+08	4,43E+08
G1T9X8	1,37E+08	1,18E+08	0	3,85E+08	1,88E+08	2,99E+08
G1T9Y5	1,83E+08	6,28E+08	5,98E+08	1,63E+08	2,01E+08	2,45E+08
G1T9Z9	1,18E+09	5,43E+08	1,97E+08	6,88E+08	5,02E+08	6,04E+08
G1TTP5;G1TA01	9,27E+09	2,31E+09	2,32E+09	1,23E+10	1,06E+10	8,09E+09
G1TA04	1,03E+10	6,43E+09	5,87E+09	9,3E+09	8,07E+09	8,93E+09
O77751;G1TA10	0	0	0	0	3,21E+08	3,24E+08
G1TA11	8,03E+08	2,84E+09	2,91E+09	1,37E+09	1,14E+09	4,82E+08
G1TA15	6,31E+08	1,9E+09	2,41E+09	7,8E+08	4,83E+08	4,72E+08
G1TA21	1,49E+09	3,81E+08	3,82E+08	1,05E+09	9,8E+08	1,03E+09
G1TA37	7,09E+08	4,91E+09	4E+09	1,31E+09	2,02E+09	2,17E+09
G1TA38	6,13E+08	2,96E+08	2,37E+08	3,8E+08	4,25E+08	4,75E+08
G1TA40	2,87E+08	0	0	2,71E+08	3,15E+08	2,23E+08
G1TA42	4,6E+08	1,62E+08	1,56E+08	3,54E+08	2,6E+08	1,69E+08
G1TA48	0	4,3E+08	1,17E+09	2,03E+08	1,92E+08	2,62E+08
G1TA50	1,34E+08	4,51E+08	6,07E+08	0	1,42E+08	85081000
G1TA53	1,02E+08	0	0	0	0	0
G1TA59	3,7E+10	1,16E+10	1,06E+10	1,12E+10	1,57E+10	1,67E+10
G1TA69	2,53E+09	7,84E+08	3,81E+08	2,79E+09	3,41E+09	4,01E+09
G1TA79	0	0	0	1,25E+08	1,35E+08	1,42E+08
G1TA80	0	2,47E+08	0	0	0	0
G1TA83	1,73E+09	1,08E+10	1,05E+10	1,7E+09	3,06E+09	2,08E+09
G1TA86	5,56E+08	1,82E+08	1,5E+08	2,11E+08	1,8E+08	1,74E+08
G1TAA4	1,14E+09	3,97E+08	4,47E+08	1,35E+09	1,13E+09	7,93E+08
G1TAB2	2,27E+09	8,31E+08	5,31E+08	1,41E+09	2,09E+09	2,65E+09
G1TAB7	1,01E+10	1,96E+09	2,08E+09	8,66E+09	8,67E+09	9,37E+09
G1TAB8;G1TN05;G1U6S9	1,19E+08	0	0	0	0	0
G1TAC3	3,52E+09	9,36E+08	5,22E+08	3,35E+09	2,67E+09	1,58E+09
P12864;G1TAC4	4,97E+08	2,67E+09	3,67E+09	8,04E+08	1,24E+09	1,14E+09
G1TAD3	32174000	39202000	26873000	67546000	3,25E+08	3,16E+08
G1TAE0	67830000	0	0	0	0	0
G1TAE2;G1SPW3;G1TWN7	8,96E+09	3,08E+09	2,78E+09	6,24E+09	6,54E+09	6,45E+09
G1TAE4	0	0	2,96E+08	0	0	0
G1TAF5	4,38E+08	5,28E+08	4,17E+08	1,1E+08	3,1E+08	2,72E+08
G1TAF8	8,12E+09	3,41E+10	3,73E+10	4,43E+09	6,9E+09	7,24E+09
G1TAG3;G1SLM9	2,85E+08	1,96E+08	1,37E+08	2,94E+08	2,67E+08	5,62E+08

G1TAH7	3,72E+10	2,85E+11	2,50E+11	5,35E+11	6,74E+11	5,12E+11
G1TAI0	1,61E+09	3,5E+09	2,62E+09	2,37E+08	3,3E+08	3,99E+08
G1TAI8	2,34E+08	6,93E+09	5,84E+09	5,02E+08	2,67E+08	3,13E+08
G1TAJ3;G1TX67	0	0	1,44E+08	0	0	1,27E+08
G1TAK0	0	1,52E+09	1,67E+09	1,4E+09	1,42E+09	3,6E+09
G1TAK1	3,89E+09	4,93E+08	5,26E+08	2,3E+09	2,51E+09	2,34E+09
G1TAK6	1,74E+09	3,37E+09	6,31E+09	1,86E+09	2,25E+09	1,49E+09
G1TVU5;G1TAL1	0	4,01E+08	4,66E+08	2,67E+08	2,26E+08	4,38E+08
G1TAL6;G1SPP7	4,48E+09	8,7E+08	9,77E+08	4,76E+09	3,6E+09	2,24E+09
G1TAM3	1,77E+08	2,7E+09	2,9E+09	2,58E+08	3,38E+08	2,73E+08
G1TAM8;G1TGJ6	1,25E+09	5,02E+09	5,14E+09	1,86E+09	2,13E+09	2,57E+09
G1TAN3	0	4,1E+08	3,39E+08	0	0	0
G1TAN4	1,29E+08	72824000	68574000	1,23E+08	1,17E+08	1,01E+08
G1TAN9	6,43E+08	3,2E+09	3,8E+09	3,22E+08	5,37E+08	2,37E+08
G1TAP1	1,07E+09	5,84E+09	5,07E+09	0	6,68E+08	0
G1TAQ9	3,69E+08	0	0	0	0	0
G1TAR0	3,03E+08	81197000	65323000	73787000	0	0
G1TAS1	2,61E+08	0	1,92E+08	0	0	0
G1TAS9;G1TAV8	1,52E+09	3,14E+08	3,08E+08	6,94E+08	1,06E+09	1,25E+09
G1TAT4	0	1,12E+08	0	0	0	0
G1TAU6	0	0	0	3,19E+08	2,52E+08	4,71E+08
G1TAU7	3,99E+08	0	1,41E+08	1,78E+08	2E+08	2,18E+08
G1TAV2	72730000	0	0	1,02E+08	0	0
G1TAV7	0	0	0	2,91E+08	2,34E+08	1,71E+08
G1TAV9	1,42E+08	1,86E+08	0	6,28E+08	2,4E+08	0
G1TAW7	2,28E+08	2,12E+09	1,63E+09	1,67E+08	2,69E+08	0
U3KP94;G1TAY2;G1TUH1	0	4,3E+08	3,97E+08	0	0	0
U3KLX8;G1TAY8	5,98E+08	4,51E+08	4,86E+08	9,78E+08	1,09E+09	9,34E+08
G1U2T0;G1TAZ4	97909000	0	0	0	0	0
G1TB05	6,45E+09	1,71E+09	2E+09	2,48E+09	1,87E+09	1,34E+09
G1TB07;G1TME1	0	0	0	0	80274000	0
G1TB10	2,39E+08	0	0	1,46E+08	1,51E+08	1,41E+08
G1TB17	0	8,69E+08	1,01E+09	5,03E+08	0	0
G1TB19	3,42E+09	1,3E+09	1,52E+09	1,38E+10	1,37E+10	1,59E+10
G1TB33	2,17E+09	4,5E+08	4,15E+08	8,84E+08	1,28E+09	1,33E+09
G1TB34	5,46E+08	2,67E+08	2,07E+08	5,49E+08	3,32E+08	3,09E+08
G1TB39	2,38E+08	2,78E+08	4,7E+08	1,36E+08	0	0
G1TB40	1,51E+08	1,61E+09	1,27E+09	0	2,82E+08	3,17E+08
G1TB45	4,95E+08	5,59E+08	7,79E+08	5,95E+08	5,46E+08	6,27E+08
G1TB47	0	1,15E+09	1,61E+09	0	0	0
G1TB49	1,49E+09	1,55E+09	1,57E+09	1,23E+09	1,53E+09	1,48E+09
G1TB50	3,35E+09	5,55E+08	6,43E+08	1,13E+08	1,18E+08	0
G1TB57	1,86E+09	3,86E+08	6,31E+08	6,04E+08	5,81E+08	5,67E+08
G1TB64	4,25E+08	0	0	0	0	0
G1TB66	36679000	0	2,1E+08	0	0	0
G1TB68	0	0	1,96E+08	0	0	0
G1TB71	0	1,94E+08	0	0	0	0
G1TB74	5,19E+09	3,93E+09	3,16E+09	8,09E+10	8,26E+10	1,12E+11
Q2PS21;G1TB78	0	2,63E+08	3,61E+08	1,4E+08	3,32E+08	1,69E+08
G1TB95	6,68E+08	7,84E+08	7,08E+08	8,5E+09	7,31E+09	8,23E+09

G1TB98	7,83E+09	4,45E+09	4,9E+09	3,73E+09	5,06E+09	6,69E+09
G1TBA4	3,05E+08	0	92562000	0	0	0
G1TBC0	1,54E+08	2,25E+08	2,23E+08	8,97E+08	7,39E+08	4,17E+08
G1TBC1;O18750	2,78E+11	1,55E+11	1,37E+11	3,17E+11	3,07E+11	4,44E+11
G1TBC4	1,14E+09	4,67E+08	3,18E+08	3,69E+08	0	3,3E+08
G1TBC6	1,51E+09	5,51E+08	5,28E+08	1,66E+09	2,21E+09	2,46E+09
G1TBC8	0	5,4E+08	1,89E+08	0	0	0
G1TBD8	2,24E+08	67045000	1,69E+08	4,08E+09	3,03E+09	3,6E+09
G1TBE5	92091000	0	0	0	0	0
G1TBE9	0	0	0	4,33E+08	2,81E+08	4,63E+08
G1TBH5;G1U5N8	3,7E+09	3,32E+09	3,15E+09	5,38E+09	5,94E+09	7,1E+09
G1TBH6	0	0	0	1,36E+08	0	91866000
G1TBI3	3,06E+08	0	0	0	0	0
G1TBK0	0	3,48E+08	4,82E+08	0	0	0
G1TBL0	4,46E+08	7,45E+08	9,34E+08	0	0	0
G1TBL1	2,68E+09	6,64E+08	4,89E+08	3,15E+09	2,14E+09	1,71E+09
G1TBL6;G1SF26	3,41E+10	3,82E+10	3,9E+10	2,02E+10	1,65E+10	8,71E+09
G1TBN5	6,45E+08	2,47E+08	2,26E+08	6,37E+08	4,25E+08	1,12E+09
G1TBQ5	0	1,79E+09	1,81E+09	4,68E+08	4,65E+08	3,78E+08
G1TBQ6	3,57E+09	1,21E+09	7,74E+08	6,81E+09	5,99E+09	6,47E+09
G1TBR1	0	0	1,66E+08	0	0	0
G1TBR5	5,49E+09	2,15E+09	1,79E+09	6,48E+09	5,74E+09	4,28E+09
U3KN28;G1TBR6	37483000	0	0	0	0	0
G1TBS1	1,24E+10	2,77E+10	2,37E+10	2,16E+10	2,67E+10	2,82E+10
G1TBS2	4,36E+09	9,04E+09	9,06E+09	2,78E+09	2,94E+09	2,56E+09
G1TBS4;G1SMV4;Q00804-2	1,53E+09	3,55E+08	4,68E+08	2,85E+09	2,81E+09	2,13E+09
G1TBT4;G1SNL8	94235000	0	0	0	0	0
G1TBU4	1,79E+08	0	0	0	0	0
G1TBU8	3,09E+08	3,85E+08	3,06E+08	0	0	0
G1TBU9	5,31E+10	1,39E+10	1,13E+10	1,76E+10	1,5E+10	1,5E+10
G1TBV4	2E+08	5,42E+09	5,73E+09	92470000	0	58127000
G1TBW1	1,41E+09	6,98E+09	8,02E+09	1,95E+09	2,34E+09	2,62E+09
G1TBW7;G1SFI5;G1SMS5	2,45E+09	4,39E+08	1,92E+08	3,08E+09	2,77E+09	1,99E+09
G1TBW9	1,5E+08	5,41E+08	3,84E+08	2,87E+08	1,31E+08	5,24E+08
G1TBX7;G1TZW4	0	0	0	22116000	0	0
G1TBX9	0	0	89233000	0	0	0
G1TBY1	3,08E+10	1,39E+10	1,55E+10	1,33E+10	1,35E+10	2,03E+10
G1TBY2	0	4,46E+08	4,62E+08	0	0	0
G1TBY3	0	0	0	0	16078000	0
G1TBY9	0	0	2,06E+08	0	0	0
G1TBZ1	0	2,03E+08	1,16E+08	0	0	0
G1TBZ5;U3KPE7	3,32E+09	1,81E+10	1,87E+10	7,11E+09	8,17E+09	7,28E+09
G1TC03	3,86E+09	1,18E+10	1,26E+10	2,88E+10	2,88E+10	3,85E+10
G1TC07	0	1,04E+08	65174000	0	77392000	66890000
G1TC10	3,99E+08	1,88E+09	2,47E+09	0	1,65E+08	0
G1TC19	2,21E+09	4,16E+08	2,19E+08	1,15E+09	9,65E+08	8,77E+08
G1TC33;Q99105	0	0	0	0	60558000	0
G1TC38	5,19E+08	2,66E+08	2,79E+08	7,11E+08	9,99E+08	1,03E+09
G1TC48	3,25E+08	1,08E+09	1,12E+09	1,23E+09	5,78E+08	9,08E+08
G1TC61;G1SCN3	1,2E+09	4,78E+09	4,71E+09	4,84E+09	4,88E+09	7,09E+09

G1TC64	1,27E+09	1,72E+08	1,93E+08	2,13E+08	2,16E+08	2,6E+08
G1TC70;G1SJB0	1,95E+09	2,58E+09	2,23E+09	2,63E+09	2,81E+09	3,46E+09
G1TC90	0	1,93E+08	0	0	0	0
G1TCA0	3,77E+08	0	0	0	0	0
G1TCA8	0	0	0	0	0	1,78E+08
G1TCB7	9,83E+08	3,11E+09	2,93E+09	5,8E+08	7,74E+08	8,49E+08
G1TCC1	1,5E+09	3,35E+08	3,21E+08	1,01E+09	1,45E+09	3,67E+08
G1TCC2	1,5E+08	76279000	0	2,49E+08	1,94E+08	6,49E+08
G1TCC8	1,88E+09	1,56E+09	1,91E+09	2,75E+09	3,02E+09	3,92E+09
G1TCD1;G1TF66	7,6E+08	0	0	2,53E+08	2,33E+08	2,31E+08
G1TCD4	2,24E+08	8,5E+08	9,32E+08	8,15E+08	9,07E+08	9,96E+08
G1TCE2	2E+09	6,18E+08	6,32E+08	2,9E+09	2,26E+09	1,31E+09
G1TCE9	5,8E+09	9,35E+08	9,89E+08	4,32E+09	4E+09	3,45E+09
G1TCF3	1,01E+09	1,75E+09	1,08E+09	3,59E+08	5,12E+08	5,45E+08
G1TCG3	7,02E+08	9,02E+08	5,8E+08	1,53E+09	8,29E+08	1,78E+09
G1TCH9	1,03E+09	2,59E+08	3,04E+08	1,02E+09	6,6E+08	2,95E+08
G1TCI9	7,37E+08	0	0	0	0	0
G1TCJ4	1,31E+08	0	0	85238000	1,41E+08	0
G1TCJ8	7,39E+08	5,83E+08	4,23E+08	7,86E+08	7,95E+08	7,41E+08
G1TCK9	5,09E+08	1,64E+09	1,71E+09	4,41E+08	4,13E+08	1,63E+08
G1TCL7	4,54E+08	3,55E+08	4,65E+08	2,7E+09	2,04E+09	3,19E+09
G1TCM0	64205000	1,64E+08	0	0	47143000	0
G1TCM9	6,43E+08	3,2E+09	3,48E+09	4,36E+08	5,45E+08	6,19E+08
G1TCP3	1,93E+09	1,26E+10	1,36E+10	3,02E+09	3,22E+09	4,73E+09
G1TCP8	1,01E+09	3,75E+08	2,93E+08	1,22E+09	1,62E+09	2,21E+09
G1TCQ4	2,08E+08	7,8E+08	4,25E+08	0	0	0
G1TCS3;G1TG27	71852000	1,19E+09	1,17E+09	1,25E+08	2,62E+08	1,6E+08
G1TCS8	2,31E+10	7,42E+09	9,17E+09	1,73E+10	1,45E+10	9,88E+09
G1TCT3	1,93E+08	5,28E+08	8,82E+08	0	2,11E+08	2E+08
G1TCU4	1,35E+09	8,83E+08	1,1E+09	1,5E+09	1,54E+09	2,06E+09
G1TCU6;G1SNW9	1,32E+08	0	73938000	3,06E+08	4,59E+08	4,26E+08
G1TCU9	0	0	0	7,92E+08	6,82E+08	8,32E+08
G1TCW1;G1SDP0;G1TTY5	1,97E+10	5,41E+09	6,47E+09	6,9E+09	6,43E+09	4,49E+09
G1TCW2	2,8E+08	1,49E+09	1,66E+09	2,28E+08	2,63E+08	2,87E+08
G1TCW5	2,91E+08	1,53E+09	1,26E+09	1,46E+08	3,15E+08	1,94E+08
G1TCX0	4,08E+09	1,04E+09	7,74E+08	1,78E+09	1,81E+09	1,94E+09
G1TCX6	3,94E+09	7,73E+08	8,08E+08	2,31E+09	1,59E+09	1,34E+09
G1TCY3	1,34E+09	0	0	0	0	0
G1TCY4	5,72E+08	2,21E+09	2,42E+09	9,31E+08	8,84E+08	1,52E+09
G1TCY7	0	0	1,39E+08	0	0	0
G1TCY8	2,83E+08	6,29E+08	5,09E+08	3,65E+08	6,63E+08	5,2E+08
G1TCZ0	2,47E+10	7,03E+09	5,83E+09	3,23E+10	3,09E+10	4,37E+10
G1TCZ8	3E+09	5,78E+09	4,8E+09	1,36E+09	1,3E+09	1,51E+09
G1TD13	89456000	0	0	0	0	0
G1TD22	0	1,59E+08	0	0	0	0
G1TD26	1,82E+09	3,68E+08	4,43E+08	3,72E+08	3,3E+08	0
G1TD30	1,81E+08	4,68E+08	3,52E+08	2,19E+09	3,53E+09	7,76E+09
G1TD34	0	0	0	1,36E+08	1,36E+08	0
G1TD36	4,19E+08	0	0	2,58E+08	0	0
G1TD41;G1U248	1,42E+10	1,25E+10	1,36E+10	1,17E+10	1,54E+10	1,79E+10

G1TN68;G1TD51	2,43E+09	2,47E+09	1,55E+09	4,83E+09	5,75E+09	4,88E+09
G1TD61	4,38E+08	3,59E+08	3,42E+08	6,27E+08	7,03E+08	7,6E+08
G1TD69	2,43E+08	0	0	0	0	0
G1TD91;G1TQQ3;G1TSJ3	5,31E+08	1,22E+09	1,02E+09	1,54E+09	2,24E+09	1,4E+09
G1TD98;U3KP75	9,77E+08	2,09E+09	2,03E+09	8,81E+08	9,54E+08	9,83E+08
G1TD99	8,53E+09	3,47E+09	2,82E+09	5,53E+09	5,38E+09	5,91E+09
G1TDA7	2,7E+09	5,8E+08	7,96E+08	1,59E+09	1,45E+09	1,7E+09
G1TDA9	4,46E+08	9,22E+08	7,24E+08	7,25E+08	7,29E+08	8,24E+08
G1TDB3	2,96E+09	1,2E+10	1,11E+10	7,15E+09	6,75E+09	7,95E+09
G1TDB8	5,9E+08	3,49E+08	0	1,12E+09	6,75E+08	4,73E+08
G1TDC3	6,28E+10	1,97E+10	1,99E+10	3,73E+10	2,8E+10	2,7E+10
G1TDD2	3,05E+08	2,21E+09	2,61E+09	3,04E+08	2,86E+08	3,38E+08
G1TDF6	4,04E+08	3,41E+08	0	7,05E+08	4,77E+08	0
G1TDH4;U3KP56	4,47E+10	1,14E+10	1,2E+10	1,92E+10	2,34E+10	2,61E+10
G1TDH6	6,07E+08	0	0	2,35E+08	3,53E+08	3,19E+08
G1TDI0	4,61E+09	1,27E+10	1,37E+10	2,29E+09	2,87E+09	2,01E+09
G1TDI1	3,16E+08	0	0	0	0	0
G1TDI3	2,02E+08	5,39E+08	0	1,14E+09	8,26E+08	1,46E+09
G1TDJ2	1,3E+09	8,07E+08	9,95E+08	1,3E+09	2,05E+09	1,55E+09
G1TDJ3	1,02E+10	2,88E+09	2,65E+09	9,13E+09	7,44E+09	5,6E+09
G1TDJ9;G1SQ94;G1SMM8	2,37E+08	5,56E+08	6,75E+08	3,29E+08	3,73E+08	3,13E+08
G1TDK0;G1TDJ5	0	0	1,58E+08	0	0	0
G1TDK8;G1SGG5	3,23E+09	1,77E+09	1,55E+09	2,11E+09	2,4E+09	2,47E+09
G1U1G9;G1TDL2;G1TTU0	7,48E+09	2,26E+10	2,47E+10	7,05E+09	8,01E+09	6,56E+09
G1TDM4	0	0	1,34E+08	0	0	0
G1TDM9	2,26E+09	9,71E+08	8,91E+08	1,46E+09	2,16E+09	2,72E+09
G1TDN1	2,67E+09	1,11E+09	1,47E+09	4,88E+09	7,66E+09	8,81E+09
G1TDN3	8,01E+08	3,83E+08	2,75E+08	4,21E+08	5,35E+08	5,71E+08
G1TDN4	3,27E+09	8,33E+09	9,93E+09	1,8E+09	2,03E+09	2,94E+09
U3KNF5;G1TDQ1	60818000	3,59E+08	4,14E+08	0	69350000	83252000
G1TDQ2;G1TH28	8,02E+08	3,66E+09	3,87E+09	5,67E+08	6,01E+08	6,22E+08
G1TDQ3	9,54E+08	4,38E+09	3,84E+09	3,27E+09	3,13E+09	3,15E+09
P00511;G1TDQ5	1,8E+08	1,04E+09	1E+09	3,19E+08	2,67E+08	2,13E+08
G1TDS3	6,98E+08	1,37E+08	1,2E+08	1,63E+08	1,61E+08	1,61E+08
G1TDT3	2,53E+08	1,38E+09	8,26E+08	3,84E+08	1,99E+08	0
G1TDU0;G1TMW4	9,68E+08	2,34E+09	2,72E+09	6,54E+08	5,36E+08	4,88E+08
G1TDU5;G1TF09	7,65E+08	2,27E+08	3,69E+08	1,18E+10	1,74E+10	2,12E+10
G1TDV4	0	0	2,89E+08	0	0	1,49E+08
G1TDX2	1,29E+09	5,81E+08	1,6E+08	7,9E+08	9,91E+08	5,22E+08
G1TDY4	88881000	0	0	0	0	0
G1TE03	5,98E+08	0	2,05E+08	2,7E+08	2,96E+08	3,66E+08
G1TE08	1,7E+09	9,96E+08	1,11E+09	1,54E+09	1,65E+09	1,65E+09
G1TE13	1,51E+09	3,49E+08	3,39E+08	4,52E+08	3,84E+08	3,91E+08
G1TE17	0	0	0	52418000	0	0
U3KPE9;G1TE20	9,39E+08	64750000	1,31E+08	2,3E+08	1,51E+08	1,37E+08
G1TE27	5,56E+09	2,02E+09	2,37E+09	4,62E+09	5,27E+09	5,16E+09
G1TE34	4,17E+09	1,22E+09	1,32E+09	1,17E+10	1,36E+10	1,62E+10
G1TE37	7,86E+08	0	0	0	3,31E+08	2,93E+08
G1TE39	2,67E+09	1,91E+09	1,25E+09	1,04E+10	6,52E+09	5,82E+09
G1TE45;G1TML5;G1TLG3	0	0	2,78E+08	0	0	0

Q9N0V7;G1TE53	0	0	1,03E+08	0	0	0
G1TE60	2E+08	1,3E+08	85170000	88209000	0	0
G1TE61;G1STV1	9,11E+08	1,81E+09	1,86E+09	5,23E+08	4,59E+08	5,81E+08
G1TE64	1,67E+09	9,3E+08	9,98E+08	1,67E+09	1,5E+09	1,37E+09
G1TE68	5,96E+09	3,72E+09	2,76E+09	9,31E+09	1,13E+10	1,39E+10
G1TE69;G1T068	6,86E+09	6,98E+09	6,77E+09	1,23E+10	1,11E+10	1,54E+10
G1TE76	6,83E+08	6,73E+09	8,68E+09	1,3E+09	1,15E+09	1,93E+09
G1TE78	4,14E+08	1,56E+09	1,9E+09	3,77E+08	1,01E+09	1,12E+09
G1TE83	2,52E+08	4,33E+08	4,64E+08	2,84E+08	4,24E+08	3,21E+08
G1TE88	0	64251000	0	0	0	0
G1TE89	63258000	0	0	0	0	0
G1TE90	6,1E+08	1,76E+08	1,28E+08	3,22E+08	1,01E+08	1,33E+08
G1TE96	0	3,76E+08	5,77E+08	2,24E+08	2,19E+08	1,82E+08
G1TEA0	4,69E+08	2,58E+08	2,38E+08	4,85E+08	4,81E+08	8,64E+08
G1TEA5	2,72E+08	1,49E+09	1,48E+09	2,47E+08	5,99E+08	4,25E+08
G1TEA8;G1SIV0;U3KN16	2,9E+09	1,51E+10	1,9E+10	4,59E+08	3,56E+08	4,76E+08
G1TED0	1,96E+09	1,04E+09	8,52E+08	9,31E+08	9,76E+08	9,82E+08
G1TED6	4,86E+09	2,25E+10	2,29E+10	2,2E+10	2,33E+10	2,6E+10
G1TEE3;G1SRX1	0	1,46E+08	2,05E+08	1,37E+08	98862000	1,35E+08
G1TEG1;U3KNA6	1,71E+09	7,04E+08	4,41E+08	1,34E+09	7,11E+08	6,22E+08
G1TEG8	3,19E+09	8,8E+09	8,75E+09	2,39E+09	2,12E+09	1,74E+09
G1TEH2	3,67E+08	2,93E+08	3,41E+08	3,6E+08	2,83E+08	2,49E+08
G1TEI0	0	1,14E+09	1,51E+09	2,13E+09	1,17E+09	1,29E+09
G1TEI1;U3KNK5	3,7E+09	1,49E+09	1,56E+09	4,53E+09	4,49E+09	4,99E+09
G1TEI2	2,78E+09	4,84E+08	5,66E+08	7,73E+08	1,83E+09	1,93E+09
G1TEK3	4,58E+08	0	0	0	0	2,38E+08
G1TEM5	2,32E+09	2,44E+09	2,51E+09	2,66E+09	3,14E+09	3,66E+09
G1TEM7	5,82E+09	2,88E+10	2,76E+10	6,9E+09	7,6E+09	6,54E+09
G1TEN1	1,39E+09	1,5E+09	1,2E+09	8,84E+08	8,81E+08	8,96E+08
G1TEN4;U3KNV4	1,23E+10	4,79E+09	5,93E+09	7,26E+09	7,92E+09	8,69E+09
G1TEN5	5,27E+09	6,69E+08	7,3E+08	6,01E+09	5,03E+09	6,06E+09
G1TEN9	1,6E+08	0	0	1,03E+08	0	0
G1TEP2	6,07E+08	0	0	4,83E+08	0	0
G1TEQ9	0	0	3,48E+08	0	0	0
G1TER3	1,18E+10	4,07E+09	4,01E+09	4,84E+10	5,62E+10	5,64E+10
G1TES0;G1TTQ1	0	0	0	1,29E+08	0	0
G1TES1	0	0	0	0	0	39420000
G1TES2;G1SEZ3;G1U0F8	8,78E+09	4,51E+09	4,07E+09	7,25E+09	9,76E+09	1,08E+10
G1TES5	1,65E+08	0	0	1,1E+08	1,11E+08	0
G1TES6	3,38E+10	1,11E+10	9,82E+09	1,42E+10	1,17E+10	1,08E+10
G1TET2	1,36E+09	2,62E+09	2,64E+09	1,29E+09	1,26E+09	1,98E+09
G1TET3	5,09E+08	1,03E+08	3,37E+08	2,52E+08	3,29E+08	2,84E+08
U3KPF0;G1TEU5	3,48E+08	1,9E+09	2,79E+09	3,04E+08	3,84E+08	3,47E+08
G1TEU8	3,61E+08	6,04E+08	6,29E+08	2,37E+08	3,75E+08	0
G1TEV2	1,69E+09	4,02E+08	4,7E+08	1,34E+09	1,54E+09	1,44E+09
G1TEW3;G1U118	2,36E+09	8,96E+09	8,65E+09	1,21E+09	1,37E+09	1,16E+09
G1TEW4	1,02E+08	1,74E+08	1,59E+08	0	0	0
G1TEZ1	8,86E+08	1,43E+08	1,43E+08	95500000	0	0
G1TF14	5,33E+09	3,59E+10	5,04E+10	2,26E+09	3,24E+09	2,47E+09
G1TF32	7,56E+09	1,81E+09	1,75E+09	7,76E+09	6E+09	4,62E+09

G1TF72	9,6E+08	4,5E+08	4,77E+08	6,98E+08	7,85E+08	1,12E+09
G1TF80;G1SWM0	1,59E+08	0	0	1,03E+08	1,2E+08	0
G1TF82	3,56E+08	4,93E+08	1,06E+09	0	3,63E+08	0
G1TFB5	3,57E+09	1,45E+09	1,96E+09	3,46E+09	2,39E+09	1,93E+09
G1TFD8	7,32E+08	1,49E+08	1,47E+08	2,39E+08	1,95E+08	2,04E+08
G1TFE0	3,33E+09	6,17E+09	5,68E+09	3,89E+09	2,46E+09	1,6E+09
G1TFE8;G1TTE5	1,15E+09	8,04E+09	6,26E+09	1,86E+09	1,59E+09	1,87E+09
U3KM48;G1TFI4	3,23E+08	1,31E+09	6,67E+08	3,26E+08	3,46E+08	0
G1TFK0	0	0	0	0	0	1,26E+08
G1TFL3	3,3E+09	1,39E+10	1,45E+10	2,8E+09	2,73E+09	2,54E+09
G1TFM5;U3KMN4	8,29E+09	1,33E+10	1,31E+10	1,14E+10	1,51E+10	1,39E+10
G1TFR5	0	0	0	32049000	0	0
G1TFS6;U3KPF2	0	4,58E+08	5,92E+08	0	0	0
G1TFT4;G1TTY8	0	4,13E+08	5,47E+08	7,03E+08	8,28E+08	6,33E+08
G1TFU9	9,42E+08	4,09E+08	7,17E+08	5,76E+08	6,26E+08	6,68E+08
G1TFV2	0	1,34E+08	0	0	0	0
G1TFV8	2,07E+08	1,34E+08	0	0	0	0
G1TFW8	0	0	0	0	0	9064800
G1TFX7;G1T421	1,49E+08	2,94E+08	1,37E+09	1,68E+08	1,57E+08	1,56E+08
G1TFY8	2,28E+09	8,43E+08	7,63E+08	8,85E+08	1,02E+09	1,44E+09
G1TFZ6	2,96E+08	9,79E+08	8,77E+08	0	2,91E+08	0
G1TFZ7	1,55E+08	3,32E+09	4,17E+09	4,95E+08	8,93E+08	6,64E+08
G1TG03	1,82E+08	0	0	0	0	0
G1TG28	7,3E+09	2,78E+09	3,54E+09	1,1E+09	2,14E+09	1,62E+09
G1TG30;G1TUS9;P00563	0	0	2,8E+08	0	0	0
G1TG89;U3KPI0;G1TPZ0	2,58E+09	5,38E+09	6,02E+09	2,83E+09	2,65E+09	2,12E+09
G1TGA8	7,91E+09	3,84E+09	3,2E+09	2,4E+10	1,58E+10	1,3E+10
G1TGD9	5,82E+08	0	0	1,07E+09	9,53E+08	1,15E+09
G1TWX4;G1TGF1;G1T1F5	4,27E+09	1,14E+10	1,43E+10	1,45E+09	1,94E+09	2,35E+09
G1TGH1	3,56E+08	0	0	0	76514000	78505000
G1TGH4	0	0	0	5,4E+08	2,69E+08	1,43E+08
G1TGK9	2,11E+09	1,02E+09	1,26E+09	1,64E+09	1,28E+09	8,48E+08
G1TGM9	1,85E+08	1,78E+08	0	0	0	0
G1TGP7	79779000	0	0	62921000	68646000	0
G1TGT5	6,21E+09	2,06E+09	1,98E+09	5,08E+09	3,48E+09	2,12E+09
G1TGT8	2,25E+08	77770000	71272000	1,94E+08	3,69E+08	4,1E+08
G1TGW1;G1TLV8;G1SPI8	9,34E+08	5,78E+09	5,55E+09	4,17E+08	8,38E+08	6,87E+08
G1TGX2	3,26E+08	2,09E+08	2,57E+08	1,8E+08	2,52E+08	2,09E+08
G1TGX6;G1U296	2,58E+08	3,81E+08	2,68E+08	2,55E+09	2,3E+09	2,1E+09
G1TGZ1	0	0	1,69E+08	0	0	0
G1TGZ6	0	0	0	4,18E+08	0	0
G1TGZ8	87249000	4,79E+08	4,74E+08	1,3E+08	0	0
G1TH06	3,14E+09	2,24E+10	2,99E+10	1,71E+09	1,94E+09	1,97E+09
G1TH09;G1TUS2	1,9E+09	5,43E+09	5E+09	1,75E+09	1,67E+09	1,19E+09
G1TH12	1,71E+09	4,06E+08	1,08E+09	1,3E+09	5,13E+08	3,85E+08
G1TH13	0	0	0	3,15E+08	0	1,66E+08
G1TH33	2,29E+10	6,48E+09	6,02E+09	1,48E+10	1,6E+10	1,45E+10
G1TH37	1,47E+08	4,66E+08	0	0	0	0
G1TH48	0	0	1,48E+08	0	0	0
G1TH59;G1SGL7	1,52E+09	1,24E+09	1,16E+09	2,06E+09	1,78E+09	2,19E+09

G1TH83;G1TFZ4	1,99E+09	7,94E+08	6,22E+08	4,15E+08	3,57E+08	6,49E+08
G1TH86;P46629;G1SPC8	2,47E+08	0	0	0	0	0
G1THA5	2,35E+08	1,09E+08	1,39E+08	0	0	1,34E+08
G1THB5	1,21E+08	0	0	0	0	0
G1THF1	3,08E+09	3,42E+08	4,18E+08	4,61E+08	4,37E+08	6,03E+08
G1THG5	0	0	0	0	0	1,27E+08
G1THH4	0	1,81E+08	1,21E+08	0	0	0
G1THH7	2,83E+09	1,07E+09	1,3E+09	1,24E+09	6,72E+08	5,4E+08
G1TTB2;G1THH9	0	2,68E+08	0	0	0	0
G1THL4;G1T107;G1TZG8	1,49E+09	6,39E+09	7,09E+09	1,12E+09	1,3E+09	1,11E+09
G1THP8;G1STE8	3,21E+08	7,33E+08	8,04E+08	5,12E+08	0	0
G1THQ5	9,28E+08	3,92E+08	2,18E+08	1,2E+09	1,53E+09	9,19E+08
G1THR4	1,31E+09	4,03E+08	3,94E+08	1,36E+09	1,36E+09	1,22E+09
G1THS6	1,52E+08	0	0	0	0	0
G1THT8;G1TQC7	2,19E+08	5,92E+08	6,81E+08	1,75E+09	1,1E+09	1,95E+09
O77541;G1THU6	7,58E+08	0	0	1,29E+09	1,86E+09	1,24E+09
G1U5R4;G1THV8	1,24E+09	5,18E+08	4,87E+08	3,22E+09	3,98E+09	5,47E+09
G1THW3	6,28E+08	2,29E+08	3,01E+08	2,5E+09	1,31E+09	8,69E+08
Q28678;G1THY2	0	0	0	0	0	1,87E+08
G1THY3	0	0	2,27E+08	0	0	0
G1THY5	2,34E+08	5,54E+08	4,75E+08	0	0	0
G1THZ6;P01870	0	0	0	9,66E+08	5,25E+08	5,47E+08
G1TI00	1,17E+09	2,97E+08	3,09E+08	1,48E+09	1,5E+09	8,66E+08
G1TI02	0	0	0	80639000	0	0
G1TI08	0	0	0	0	0	76134000
G1TI18	0	0	1,91E+08	0	46303000	0
G1TI27;Q9XSC2	7,5E+08	4,21E+08	4,13E+08	1,81E+09	2,4E+09	1,55E+09
G1TI39	3,74E+08	1,23E+09	1,64E+09	2,61E+08	0	1,59E+08
G1TI40	3,68E+09	5,22E+09	5,91E+09	9,01E+09	8,74E+09	1,07E+10
G1TI55	7,8E+09	2,73E+09	2,49E+09	8,79E+09	1,03E+10	1,5E+10
G1TI56	1,65E+09	6,41E+09	6,53E+09	4,43E+09	5,14E+09	6,33E+09
G1TI59;G1TVB5	3,51E+08	0	0	5,19E+08	1,03E+09	6,89E+08
G1TI64	1,64E+08	0	0	1,53E+08	0	0
G1TI71	5,82E+08	3,03E+09	2,74E+09	8,63E+08	1,4E+09	7,06E+08
G1TI97	0	1,15E+09	5,13E+08	0	0	0
G1TIB4	7,9E+08	9E+09	1,32E+10	6,76E+08	9,99E+08	1,47E+09
G1TIB7	48616000	0	0	0	0	0
G1TIC4	0	2,74E+08	6,63E+08	0	1,45E+08	95279000
G1TIC9	5,9E+08	3,12E+08	3,99E+08	4,32E+09	2,93E+09	3,72E+09
G1TID0	0	0	35106000	0	0	0
G1TID1	0	0	0	1,77E+09	1,79E+09	2,21E+09
G1TID8;G1TZK4	0	2,88E+08	2,94E+08	0	0	0
G1TIE9	52553000	6,1E+08	8,95E+08	69775000	60780000	40286000
G1TIF1	0	2,56E+08	1,67E+08	1,43E+08	1,32E+08	1,51E+08
P37153;G1TIF8	2,46E+09	2,24E+08	2,24E+08	0	0	0
G1TII2	6,34E+09	9,05E+08	8,65E+08	2,95E+09	2,86E+09	1,92E+09
G1TIJ7	3,7E+08	1,96E+08	1,75E+08	9,66E+08	9,68E+08	1,18E+09
G1TIM0	5,56E+08	2,48E+08	1,69E+08	3,62E+08	2,58E+08	1,56E+08
G1TIR7	1,13E+09	1,65E+09	1,51E+09	1,69E+09	2,51E+09	2,33E+09
G1TIS5	3,11E+09	8,96E+09	1,04E+10	1,23E+09	1,47E+09	1,46E+09

G1TIT1	3,91E+09	7,03E+08	9,25E+08	4,19E+09	3,47E+09	3,02E+09
G1TIV8	0	0	0	3,12E+08	3,4E+08	3,54E+08
G1TIW9	1,09E+08	0	1,16E+08	0	0	0
G1TIZ1;G1TYH5	0	8,18E+08	9,77E+08	2,09E+08	3,93E+08	2,5E+08
G1TIZ2	1,01E+10	2,71E+09	2,53E+09	1,85E+10	1,52E+10	1,61E+10
G1TIZ5	2,82E+10	1,33E+10	1,35E+10	3,25E+10	3E+10	5,06E+10
G1TIZ9	0	0	0	1,74E+08	0	0
G1TJ04	1,11E+08	0	0	0	0	0
G1TJ19	3,73E+09	1,09E+09	1,1E+09	9,1E+09	1,2E+10	1,55E+10
G1TJ33;G1SVD8	0	0	0	0	3,52E+08	0
G1TJ36	2,09E+08	0	2,19E+08	3,53E+08	3,4E+08	3,94E+08
P48738;G1TJ38	1,58E+08	3,15E+08	5,05E+08	0	0	0
G1TJ80;G1TMR3	1,35E+09	3,69E+08	6,14E+08	4,54E+09	4,64E+09	5,35E+09
G1TJC3	3,67E+09	1,94E+10	1,56E+10	5,49E+08	8,66E+08	7,45E+08
G1TJG3	3,93E+09	1,52E+10	1,63E+10	6,82E+09	9,58E+09	9,31E+09
G1TJG6;P46406;G1TYG1	7,33E+10	2,10E+11	2,18E+11	7,37E+10	1,03E+11	6,13E+10
G1TJH2;G1TN82;G1SGR6	1,03E+09	6,27E+09	5,16E+09	1,9E+09	1,52E+09	1,19E+09
G1TJK2	0	0	0	0	6,51E+08	0
G1TJL0	1,21E+08	0	1,01E+08	0	0	0
G1TJL3	1,53E+08	0	93343000	1,46E+08	0	0
G1TJN6;G1U438	4,25E+08	0	0	0	0	0
G1TJN7;U3KM51;G1T8S6	2,29E+09	5,64E+08	4,65E+08	2,83E+09	2,46E+09	1,38E+09
G1TJP0	2,72E+08	9,39E+08	1,19E+09	0	3,32E+08	3,48E+08
G1TJR3;G1TI85;G1U054	2,65E+09	1,26E+10	9,55E+09	3,96E+09	4,47E+09	3,87E+09
G1TJR5	1,21E+08	6,02E+08	5,75E+08	1,6E+08	1,52E+08	0
G1TJS2	4,06E+09	1,34E+09	1,46E+09	2,71E+09	2,79E+09	3,04E+09
G1TJV3	1,23E+09	0	0	5,1E+08	3,9E+08	3,56E+08
G1TJW1;G1T3D9	9,7E+09	3,84E+10	4,1E+10	9,5E+09	9,37E+09	8,23E+09
G1TJW3;G1TQ82	2,28E+10	1,69E+10	1,7E+10	3,44E+10	4,04E+10	3,84E+10
G1TJW7	2,33E+08	1,32E+08	1,23E+08	1,18E+08	1,3E+08	92079000
G1TJW8	5,69E+08	1,39E+09	2E+09	4,48E+08	3,31E+08	3,8E+08
G1TJX7;G1TYK6	2,76E+08	1,42E+09	1,62E+09	0	2,26E+08	1,89E+08
G1TJY2	2,03E+09	4,42E+08	3,07E+08	2,85E+09	2,12E+09	2,42E+09
G1TK17	1,55E+09	4,97E+09	5,36E+09	1,58E+09	1,72E+09	1,49E+09
G1TK32	0	9,79E+08	3,87E+08	2,43E+08	2,43E+08	1,5E+08
G1TK35	0	0	0	1,3E+08	1,21E+08	0
G1TK53	0	7,04E+08	5,84E+08	2,47E+08	4,96E+08	4,66E+08
G1TK63	9,6E+08	1,73E+09	9,75E+08	5,42E+09	5,29E+09	8,89E+09
G1TK72	5,34E+08	0	5,42E+08	8,73E+08	5,29E+08	4,92E+08
G1TK74;G1TK98	0	0	0	0	1,24E+08	0
G1TK81	2,27E+08	0	0	0	0	0
G1TKA2	1,38E+08	1,01E+08	1,41E+08	1,16E+09	8,6E+08	8,82E+08
G1TKC4	2,41E+09	5,02E+09	5,18E+09	1,42E+09	1,44E+09	1,36E+09
G1TKE0	5,36E+08	1,11E+08	0	5,84E+08	4,42E+08	4,08E+08
G1TKE3	7,07E+09	4,58E+10	5,45E+10	1,46E+09	2,02E+09	2,35E+09
G1TKE8	7,05E+08	0	0	1,4E+09	1,21E+09	1,02E+09
G1TKG2	2,24E+09	4,89E+08	3,73E+08	3,78E+09	1,78E+09	1,37E+09
G1TKH3;P09212	6,07E+09	2,21E+10	1,84E+10	8,72E+09	1,08E+10	1,29E+10
G1TKI8	0	5,74E+09	6,94E+09	0	0	0
U3KMX6;G1TKK2	9,42E+08	9,12E+09	7,11E+09	2,17E+08	2,86E+08	3,93E+08

G1TM83;G1TKL0	1,77E+09	1,92E+09	2,14E+09	2,49E+09	2,2E+09	3,38E+09
G1TKL2	1,17E+10	5,53E+10	5,98E+10	6,76E+09	8,97E+09	1,23E+10
G1TKL9	0	0	0	0	0	98650000
G1TKN4;G1SY93	4,45E+09	3,65E+09	3,8E+09	4,56E+09	4,72E+09	2,51E+09
G1TKN7;G1TJM8;G1TTS2	1,19E+10	1,39E+10	1,43E+10	2,31E+10	3,06E+10	3,23E+10
G1TKS5	1,82E+09	2,32E+08	1,8E+08	2,24E+09	3,07E+09	2,74E+09
G1TKV4	0	0	0	0	41081000	0
G1TKX5	2,06E+08	0	1,27E+08	1,94E+08	4,23E+08	5,73E+08
G1TKX7	0	0	0	1,95E+08	2,62E+08	0
G1TKY3;P06814	6,14E+09	1,76E+10	1,98E+10	1,82E+09	1,61E+09	1,33E+09
G1TKY7	2,33E+08	0	0	9,32E+08	1,02E+09	4,08E+08
G1TL06;G1U3I6;G1TL63	1,01E+10	3,71E+10	3,53E+10	1,17E+10	1,11E+10	7,42E+09
G1TL18	0	0	0	2,99E+09	0	0
G1TL29;G1TWZ0	0	5,69E+08	5,88E+08	0	0	0
G1TL55	1,02E+09	0	0	4,73E+08	5,14E+08	5,02E+08
G1TL80	1,84E+09	6,31E+08	5,85E+08	1,27E+09	1,23E+09	1,75E+09
G1TLA8	82181000	0	0	0	36288000	0
G1TLD3	2,44E+10	7,8E+09	6,71E+09	4,32E+10	4,53E+10	6,2E+10
G1TLE3	0	0	0	0	0	2,32E+09
G1TLE4	1,82E+10	4,91E+09	5,42E+09	9,35E+09	1,17E+10	9,98E+09
G1TLH1	1,08E+08	2,21E+08	1,21E+08	3,85E+08	1,89E+08	2,87E+08
G1TLK9;G1SS41	2,73E+10	5,23E+09	5,41E+09	1,3E+10	1,16E+10	1,04E+10
G1TLL7;G1SSV0	1,57E+09	4,27E+08	7,95E+08	5,11E+09	3,67E+09	5,45E+09
G1TLN2;G1TZX4	1,22E+09	3,6E+08	2,25E+08	8,1E+08	7,46E+08	6,56E+08
G1TLQ8	7,26E+09	1,03E+10	1,09E+10	3,63E+09	3,83E+09	4,53E+09
G1TLS9	0	0	85473000	0	1,97E+08	1,23E+08
G1TSW1;G1TLU5;G1TG12	1,26E+08	6,62E+08	7,38E+08	3,04E+08	2,07E+08	3,68E+08
G1TLW3	1,6E+10	1,18E+10	1,28E+10	1,07E+10	1,18E+10	1,22E+10
G1TLY0	3,94E+08	9,55E+08	1,51E+09	5,77E+08	8,2E+08	6,82E+08
G1TLZ2;G1SYN5	3,12E+09	1,76E+09	1,74E+09	9,1E+09	9,71E+09	9,46E+09
G1TM00	0	5,16E+08	4,05E+08	0	0	0
G1TM33	0	0	0	0	1,41E+08	0
G1TM35	3,61E+09	1,22E+09	1E+09	3,65E+09	4,99E+09	5,93E+09
G1TM42	0	4,21E+08	4,72E+08	2,3E+08	3,49E+08	4,87E+08
G1TM45	0	0	0	3,52E+08	2,55E+08	0
G1TM48	4,58E+09	1,27E+09	1,13E+09	7,04E+09	8,58E+09	8,83E+09
G1TM55;G1TTA9;G1U5C0	2,68E+09	2,46E+10	2,37E+10	6,61E+09	7,86E+09	6,62E+09
G1TM60	1,84E+10	2,48E+09	2,05E+09	2,27E+10	1,85E+10	1,45E+10
G1TM62	1,48E+10	6,33E+09	5,34E+09	4,65E+10	5,45E+10	7,33E+10
G1TM73	0	0	2,17E+08	0	0	0
G1TM82	9,55E+08	1,04E+10	1,17E+10	1,11E+09	1,81E+09	1,68E+09
G1TM95;G1TMH7	2,27E+09	2,3E+08	1,58E+08	6,09E+08	9,83E+08	1,2E+09
G1TMA2;U3KPI1	2,1E+08	0	0	0	0	0
G1TMD0	1,24E+09	2,67E+08	2,68E+08	2,05E+09	1,88E+09	2,08E+09
G1TMD8	2,08E+08	6,55E+08	5,19E+08	1,85E+08	2,39E+08	1,35E+08
G1TME5	1,42E+08	0	0	1,32E+08	82617000	0
G1TME7	3E+09	5,23E+09	4,82E+09	2E+09	2,65E+09	2,09E+09
G1TMG9;G1TRF4	4,12E+08	3,07E+08	2,17E+08	1,71E+09	5,87E+08	4,68E+08
G1TMI5	0	0	0	0	3,41E+08	4,57E+08
G1TMM5	6,25E+08	1,97E+08	3,06E+08	8,47E+08	7,01E+08	3,51E+08

G1TMM7	2,9E+08	1,97E+09	1,89E+09	1,21E+09	1,74E+09	1,7E+09
G1TMP1;G1U758	0	0	0	0	0	2,33E+08
G1TMP7	0	0	0	0	1,22E+08	0
G1TMQ5	6,71E+08	6,65E+08	7,28E+08	1,13E+09	8,5E+08	9,42E+08
G1TMQ8	0	0	0	4,12E+08	0	3,51E+08
G1TMS5	1,39E+10	4,91E+10	5,2E+10	5,83E+09	7,3E+09	6,65E+09
G1TMS7	1,42E+08	1,18E+08	1,4E+08	6,46E+08	2,63E+08	2,19E+08
G1TMU1	1,44E+09	1,99E+08	2,4E+08	2,02E+08	2,75E+08	3,32E+08
G1TMU2;G1U3W7	1,41E+10	3,32E+10	3,27E+10	1,37E+10	1,61E+10	1,91E+10
G1TMV1;G1T6M5	6,96E+09	3,4E+10	3,37E+10	3,98E+09	4,93E+09	6,54E+09
G1TMV6	0	3,96E+08	3,11E+08	2,92E+08	0	0
G1TMW2	7,58E+08	1,36E+09	1,5E+09	4,24E+08	6,82E+08	5,9E+08
G1TMZ2	2,76E+09	6,64E+08	7,53E+08	6,71E+08	7,25E+08	6,87E+08
G1TMZ6	1,16E+10	3,54E+09	2,92E+09	6,23E+09	7,11E+09	7,81E+09
G1TN14	1,63E+08	4,64E+08	4,33E+08	7,37E+08	7,35E+08	5,96E+08
G1TN20	8,02E+09	3,81E+09	3,24E+09	1,89E+10	2,32E+10	1,35E+10
G1TN29	0	0	1,42E+08	0	0	0
G1TN33	1,48E+08	1,04E+09	1,33E+09	0	0	0
G1TN42	8,09E+08	2,76E+08	1,63E+08	1,73E+08	2,25E+08	1,96E+08
G1TN62;G1TX41	5,8E+09	2,14E+10	2,17E+10	1,38E+10	1,49E+10	1,91E+10
G1TN80;G1TL01	7,78E+08	1,47E+08	0	3,1E+08	2,16E+08	2,1E+08
G1TN86	1,11E+09	1,54E+09	1,44E+09	6,19E+08	9,87E+08	9,24E+08
G1TN89	1,41E+09	3,75E+08	4,67E+08	3,25E+10	2,06E+10	2,49E+10
G1TNA6	3,23E+08	2,97E+08	3,03E+08	0	0	0
U3KPP2;G1TNB4	0	71258000	0	0	0	0
G1TNF3	0	0	0	0	0	90779000
G1TNH9	1,24E+09	2,22E+09	2,17E+09	2,06E+09	1,61E+09	1,33E+09
G1TNI4	0	2,66E+08	0	1,2E+09	8,97E+08	7,53E+08
G1TNJ2	90535000	8,2E+09	7,97E+09	1,3E+08	32806000	1,19E+08
G1TNK9;G1TNV4	0	0	0	1,66E+08	0	0
G1TNL6;G1U4H3	5,86E+08	5,24E+09	3,91E+09	5,48E+08	7,5E+08	7,87E+08
G1TNM3;G1TG00	7,98E+09	2,45E+10	2,56E+10	8,72E+09	8,53E+09	6,01E+09
G1TNM4	0	0	0	0	0	0
G1TNP7	0	1,98E+08	1,74E+08	0	0	0
G1TNT7	1,11E+09	0	0	4,29E+08	3,77E+08	3,28E+08
G1TNT9	1,95E+08	0	0	8,72E+08	3,95E+08	5,59E+08
G1TNU3	6,35E+08	1,69E+08	2,02E+08	6,68E+08	8,6E+08	1,03E+09
G1TNV7	2,95E+09	6,14E+08	5,39E+08	3,07E+09	2,5E+09	2,17E+09
G1TNW8	1,79E+09	6,8E+08	4,8E+08	2,1E+09	1,96E+09	1,98E+09
G1TNX6	0	0	6,16E+08	0	0	0
G1TNX7;Q28719;G1SMQ0	4,69E+08	1,6E+09	1,41E+09	6,67E+08	7,16E+08	4,02E+08
G1TNY1	1,04E+10	4,71E+09	4,94E+09	9,56E+09	1,37E+10	1,58E+10
G1TNZ3	3,09E+09	9,43E+08	8,37E+08	1,09E+09	1,03E+09	5,6E+08
G1TNZ4;P98118	0	0	0	98523000	0	0
G1TP15	3,21E+09	3,64E+09	3,67E+09	1,74E+09	1,3E+09	9,99E+08
G1TP23	1,18E+09	0	0	2,2E+09	2,54E+09	6,41E+09
G1TP25	1,46E+08	4,92E+09	6,67E+09	7,11E+08	7,5E+08	1,45E+09
G1TP30	6,55E+08	3,47E+09	3,48E+09	8,2E+08	9,04E+08	9,6E+08
G1TP59;U3KLU1	2,04E+10	3,01E+09	3,09E+09	1,57E+10	1,51E+10	1,55E+10
G1TP80	0	0	0	0	0	3,1E+08

G1TP81;G1SJX0	0	1,33E+08	1,38E+08	0	0	0
G1TPB1	3,75E+08	54136000	43880000	1,7E+08	1,05E+08	0
G1TPC5	6,81E+08	3,86E+09	3,7E+09	9,78E+08	1,41E+09	1,29E+09
G1TPD4	0	0	90940000	0	0	0
G1TPG3;G1SZI5	9,6E+09	2,74E+10	2,71E+10	1,19E+10	1,2E+10	9,85E+09
G1TPI0	2,81E+09	1,26E+09	9,99E+08	8,7E+09	8,5E+09	9,02E+09
G1TPI5	1,61E+08	0	0	0	0	0
G1TPI7	0	0	0	0	1,27E+08	43568000
G1TPK8;P54938	8,6E+08	1,78E+08	1,53E+08	1,22E+08	0	1,32E+08
G1TPL7;G1T090	7,89E+08	6,33E+09	6,25E+09	2,01E+09	1,72E+09	1,3E+09
G1TPN2	1,54E+09	5,65E+09	7,22E+09	1,87E+09	2,05E+09	3,09E+09
G1TPN3	3,24E+10	3,72E+10	4,47E+10	2,21E+10	2,91E+10	3,34E+10
G1TPP1;G1TYB5	0	0	1,24E+08	0	0	0
G1TPP4;G1U3G4	0	0	0	0	2,36E+08	0
G1TPS9	1,17E+09	5,91E+08	5,94E+08	4,44E+09	3,81E+09	5,38E+09
G1TPT3	0	1,85E+08	1,72E+08	0	0	0
G1TPV0;G1TKB3;G1ST96	4,5E+09	2,36E+10	2,24E+10	8,4E+09	7,77E+09	5,48E+09
G1TPV3;G1T168;G1U0Y5	5E+09	4,91E+09	4,36E+09	5,28E+09	5,53E+09	5,83E+09
G1TPV7	9,7E+09	1,61E+09	1,33E+09	1,81E+10	1,64E+10	1,52E+10
G1TPW2	1,42E+09	2,69E+09	2,53E+09	7,65E+08	7,08E+08	6E+08
G1TPY7	1,19E+09	4,98E+08	5,5E+08	1,11E+09	1,35E+09	1,84E+09
G1TPZ1	7,1E+10	8,67E+10	9,01E+10	1,48E+09	1,48E+09	2,36E+09
G1TPZ3	1,04E+09	3,67E+08	3,93E+08	6,03E+08	6,45E+08	9,56E+08
G1TPZ4;G1TH64	5,62E+08	2,28E+08	2,63E+08	5,29E+08	6,56E+08	2,75E+08
G1TQ11	4,84E+08	0	1,42E+08	2,74E+08	1,39E+08	0
G1TQ31;P33674	2,12E+09	2,54E+09	2,63E+09	2,66E+09	2,01E+09	2,23E+09
G1TQ32	0	0	0	2,33E+08	0	0
G1TQ57	0	0	99619000	1,22E+08	0	1,65E+08
G1TQ79	8,56E+08	1,04E+09	4,05E+08	3,47E+08	5,1E+08	4,59E+08
G1TWR1;G1TRK0;G1TQ90	2,6E+08	3,71E+08	2,65E+08	1,39E+08	1,79E+08	4,31E+08
G1TQ97	5,62E+08	7,05E+08	7,78E+08	9,08E+09	6,31E+09	9,55E+09
G1TQA4	0	0	1,64E+08	0	0	0
G1TQD7	0	3,11E+08	3,04E+08	0	0	0
G1TQG1	8,23E+09	2,67E+09	1,85E+09	9,19E+09	1,09E+10	1,37E+10
G1TQG4;G1U2K2	6,13E+09	1,54E+10	2,51E+10	1,19E+09	3,44E+09	2,15E+09
G1TQI2	0	0	3,81E+08	0	0	0
G1TQJ5	7,69E+08	2,56E+09	2,33E+09	2,57E+09	2,85E+09	3,53E+09
G1TQK5	0	1,26E+09	1,07E+09	0	0	0
G1TQL7	1,31E+08	0	0	0	0	91095000
G1TQM9	6,98E+09	4,68E+10	4,54E+10	3,96E+09	4,55E+09	5,28E+09
G1TQP4	1,38E+08	2,6E+08	2,92E+08	1,7E+08	1,65E+08	1,27E+08
G1TQP6	1,82E+08	2,75E+08	2,32E+08	0	0	0
G1TQR0;G1TEM1;G1U4H8	2,27E+09	2,21E+09	2,03E+09	2,34E+09	1,99E+09	1,46E+09
P34826;G1TQR2	4,35E+09	2,62E+10	2,73E+10	2,55E+09	2,92E+09	3,19E+09
G1TQT7	0	0	0	0	1,76E+08	1,84E+08
G1TQV4	0	0	2,78E+08	0	0	0
G1TQW7	0	0	0	0	63522000	0
G1TQX1	0	4,42E+08	1,96E+08	1,32E+08	1,31E+08	0
G1TR00	7E+08	2,38E+08	2,18E+08	9,06E+08	6,46E+08	4,26E+08
G1TR26	1,78E+09	4,36E+08	3,87E+08	6,64E+08	8,49E+08	4,63E+08

G1TWU9;G1TR42	0	3,06E+08	3,44E+08	0	0	0
G1TR82	4,22E+10	2,12E+11	1,89E+11	3,59E+10	4,56E+10	4,68E+10
G1TRA4	0	0	0	59695000	0	0
G1TRA9	0	0	4,92E+08	1,06E+09	6,85E+08	5,32E+08
G1TRE0	0	0	79388000	0	0	0
G1TRF3	1,02E+08	7,45E+08	5,98E+08	1,78E+08	1,63E+08	0
G1TRG8	1,96E+10	6,08E+09	6,18E+09	1,28E+10	1,08E+10	9,84E+09
G1TRG9	5,83E+08	3,89E+09	4,31E+09	2,72E+08	2,83E+08	4,69E+08
G1TRH3	6,82E+09	6E+09	4,88E+09	2,89E+09	4,99E+09	5,77E+09
G1TRH9;G1T4H0	1,12E+09	1,92E+09	2,5E+09	1,38E+09	1,78E+09	1,79E+09
G1TRI3	0	0	0	0	0	2,68E+08
G1TRI7;Q5XTS1	0	0	0	1,03E+08	0	0
G1TRK3;P82810	1,84E+09	6,1E+09	4,81E+09	4,64E+09	7,18E+09	5,14E+09
G1TRL5;U3KLT5	3E+08	2,06E+09	2,28E+09	1,26E+08	3,14E+08	1,57E+08
G1TRL8;G1T7B7	2,15E+09	1,82E+09	1,47E+09	3,12E+09	2,43E+09	2,06E+09
G1TRM4;G1T455	4,15E+09	2,98E+10	2,99E+10	6,11E+09	5,55E+09	4,61E+09
G1TRP3;G1TYB4;G1TMT0	1,48E+08	1,67E+08	1,95E+08	4,53E+08	5,38E+08	5,45E+08
G1TRP6	0	0	0	0	0	3,87E+09
G1TRR7	3,71E+08	0	0	2,64E+08	1,85E+08	2,63E+08
G1TRS0	1,12E+09	6,46E+08	5,06E+08	1,06E+09	6,83E+08	4,36E+08
G1TRS4	1,77E+10	7,76E+09	7,53E+09	2,79E+10	3,19E+10	4,6E+10
G1TRT5	3,06E+09	2,73E+09	2,92E+09	6,61E+09	5,98E+09	5,93E+09
G1TRV4	4,65E+08	1,48E+09	1,18E+09	3,95E+08	1,05E+09	9,75E+08
G1TRV7;G1TAT9	7,24E+08	0	0	2,14E+08	2,4E+08	1,84E+08
G1TRW0	1,01E+09	0	0	0	0	0
G1TRY5	5,37E+08	1,63E+09	1,59E+09	9,58E+08	6,97E+08	1,19E+09
G1TRZ2	7,5E+09	5E+09	4,28E+09	1,19E+10	1,16E+10	1,24E+10
G1TS18	0	0	0	0	1,34E+08	0
G1TS36	2,3E+09	1,9E+09	1,59E+09	2,83E+09	2,81E+09	4,39E+09
G1TS38	14131000	0	0	0	0	0
G1TS68	9,16E+08	7,96E+08	7,81E+08	4,32E+08	4,54E+08	5,47E+08
G1TS78;G1SJ74	1,03E+09	3,35E+08	2,92E+08	7,91E+08	5,63E+08	4,43E+08
G1TS90	4,17E+08	0	0	2,13E+09	1,18E+09	1,35E+09
G1TS93	1,81E+08	1,2E+09	1,1E+09	2,28E+08	2,66E+08	3,52E+08
G1TS97	3,29E+08	7,83E+08	1,28E+09	2,9E+08	4,2E+08	6,51E+08
G1TSG1	2,24E+09	6,79E+09	5,79E+09	1,94E+09	2,31E+09	2,1E+09
G1TSH8;G1U1V6;G1U324	79113000	4,74E+08	6,61E+08	0	1,11E+08	50757000
G1TSI3	47144000	89489000	99034000	0	0	0
G1TSK0	7,11E+08	2,1E+09	2E+09	1,39E+09	1,48E+09	1,87E+09
G1TSK4	5,34E+08	1,29E+08	1,05E+08	2,58E+08	1,81E+08	1,75E+08
G1TSL1	1,27E+10	2,2E+09	2,04E+09	2,89E+09	2,66E+09	2,93E+09
G1TSL5	0	6,71E+08	6,17E+08	0	0	0
G1TSM7	0	0	0	6,89E+08	5,71E+08	3,32E+08
G1TSM9	0	0	0	0	0	96554000
G1TSP3	6,02E+09	1,97E+09	1,78E+09	8,07E+09	5,81E+09	3,59E+09
G1TST2	2,42E+08	2,9E+08	0	3,68E+09	2,02E+09	1,07E+09
G1TST9;G1THA3	9,97E+08	1,65E+09	1,33E+09	2,53E+09	1,97E+09	1,41E+09
U3KPD1;G1TSU1	1,56E+08	0	0	0	0	0
G1TSX5;G1U675	0	0	0	0	0	2,8E+09
G1TSZ1	0	6,59E+08	7,35E+08	3,33E+08	2,43E+08	2,55E+08

G1TT06	1,29E+09	7,76E+09	7,35E+09	1,28E+10	1,47E+10	1,36E+10
G1TT27;G1TZB5	8,87E+09	2,45E+10	2,09E+10	9,59E+09	8,32E+09	7,03E+09
G1TT75	2,75E+10	9,26E+09	6,57E+09	5,45E+10	5,95E+10	6,33E+10
G1TTA5	1,3E+09	7,03E+08	3,68E+08	3,97E+09	2,97E+09	2,21E+09
G1TTB5;G1U8N8	5,37E+08	6,57E+08	3,6E+08	5,83E+08	8,17E+08	9,58E+08
G1TTD4	4,88E+08	2,71E+09	3,01E+09	4,27E+08	7,23E+08	4,77E+08
G1TTD6	7,3E+09	2,09E+10	2,21E+10	8,66E+09	6,22E+09	5,6E+09
G1TTE3	2,53E+08	8,38E+08	7,62E+08	0	0	0
G1TTI3	3,41E+08	8,26E+08	5,91E+08	6,3E+08	5,91E+08	1,12E+09
G1TTJ1	2,16E+08	2,22E+09	2E+09	2,37E+08	1,62E+08	2,04E+08
G1TTK6	5,27E+09	1,35E+09	1,09E+09	2,83E+09	2,31E+09	2,28E+09
G1TTL1;G1TF42	8,52E+08	3,91E+09	4,43E+09	4,58E+08	1,55E+09	2,35E+09
G1TTL7	1,48E+09	3,16E+08	2,46E+08	5,46E+08	5,63E+08	4,93E+08
G1TTM0	0	0	3,35E+09	0	0	0
G1TTM6	2,65E+09	6,97E+08	7,38E+08	1,25E+09	1,09E+09	6,95E+08
G1TTN9	1,28E+08	6,16E+08	3,39E+08	1,72E+08	0	0
G1TTP9	4,9E+08	2,56E+08	2,85E+08	6,54E+08	5,96E+08	8,6E+08
G1TTQ5;G1TBH2;G1TYX8	2,73E+09	9,9E+09	8,83E+09	3,06E+09	4,33E+09	4,46E+09
G1TTS1	6,62E+08	9,86E+08	7,51E+08	2,43E+09	2,05E+09	1,87E+09
G1TTU6	1,68E+09	3,18E+09	3,69E+09	1,27E+09	2,67E+09	3,08E+09
G1TTY7	3,96E+09	1,57E+10	1,5E+10	4,17E+09	3,72E+09	2,94E+09
G1TU00	96607000	3,62E+08	5,93E+08	1,43E+08	1,07E+08	1,37E+08
G1TU13;G1TS23	6,28E+09	2,42E+10	2,4E+10	1,03E+10	1,11E+10	1,23E+10
G1TU32	7,26E+08	5,35E+08	3,92E+08	3,86E+08	5,95E+08	5,45E+08
G1TU38	1,96E+08	0	0	0	1,11E+08	1,37E+08
G1U7Y7;G1TU65;G1SQP5	6,89E+08	5,34E+09	6,83E+09	5,19E+08	1,42E+09	7,55E+08
G1TU85	1,67E+08	83338000	86349000	0	98816000	0
G1TUA3	6,81E+10	5,8E+10	5,72E+10	1,32E+10	1,41E+10	1,42E+10
G1TUB1	0	3,2E+08	0	0	0	0
G1TUB8	7,06E+09	1,69E+10	1,53E+10	5,77E+09	6,41E+09	3,73E+09
G1TUC2	1,12E+09	1,48E+10	1,48E+10	1,58E+09	2,01E+09	2,47E+09
G1TUC5	3,64E+08	0	0	1,63E+08	1,64E+08	1,6E+08
G1TUC8	1,49E+10	1,04E+10	9,05E+09	6,59E+09	7,94E+09	7,58E+09
G1TUD2	1,97E+09	5,73E+08	4,73E+08	4,64E+09	4,81E+09	4,61E+09
G1TUD6	8,32E+09	1,44E+10	1,54E+10	5,41E+09	5,24E+09	6,55E+09
G1TUE0	2,39E+10	1,22E+10	1,18E+10	8,74E+09	8,99E+09	1,11E+10
G1TUF4	0	0	0	0	0	2,87E+08
G1TUG7;G1SGI2	0	1,06E+08	1,43E+08	0	0	0
G1TUH9;G1TVI6;G1TYJ3	7,19E+09	4,19E+10	4,42E+10	1,1E+10	1,34E+10	1,58E+10
G1TUK1	0	4,43E+08	3,45E+08	1,51E+08	2,02E+08	2,03E+08
G1TUK6	0	0	0	0	2,34E+08	0
G1TUM2	9,77E+08	1,46E+09	1,57E+09	1,2E+09	9,63E+08	5,5E+08
G1TUN6	1,49E+08	4,59E+08	6,7E+08	0	0	0
G1TUP1	4,27E+08	1,02E+09	1,33E+09	7,63E+08	1,01E+09	7,95E+08
G1TUP7;G1TTC8;G1TIW6	2,46E+09	2,35E+09	1,94E+09	1,81E+09	1,78E+09	1,61E+09
G1TUT9;G1U2S9	3,57E+09	1,75E+10	1,76E+10	4,08E+09	3,11E+09	2,51E+09
G1TUU8;Q7M370;G1U1J7	2,47E+09	5,48E+08	7,17E+08	4,26E+10	2,75E+10	2,61E+10
G1TUX2	5,2E+10	1,4E+10	1,48E+10	3,8E+10	2,92E+10	2,91E+10
G1TUY5	5,89E+09	1,35E+09	1,25E+09	1,36E+10	1,45E+10	1,71E+10
G1TV19	1,47E+09	3,3E+09	2,72E+09	2,28E+09	3,52E+09	3,56E+09

G1TV25	7,9E+08	3,18E+08	4,61E+08	4,55E+08	4,73E+08	5,36E+08
G1TV31	3,39E+08	0	0	0	0	0
G1TV43	3,96E+09	9,75E+09	8,68E+09	5,79E+09	5,26E+09	4,14E+09
G1TV79	3,62E+10	1,38E+10	1,31E+10	4,18E+10	3,6E+10	3,72E+10
G1TV91	99097000	1,63E+09	1,19E+09	7,8E+08	1,16E+09	1,77E+09
G1TV92	3,02E+08	2,88E+09	3,66E+09	2,88E+08	1,27E+08	3,26E+08
G1TVC8	1,84E+08	4,34E+08	2,65E+08	1,89E+08	0	0
G1TVD5	0	1,86E+10	4,19E+09	0	0	0
G1TVG7	1,36E+09	5E+08	6,15E+08	1,53E+09	1,24E+09	6,7E+08
G1TVG8	2,85E+09	5,42E+08	5,02E+08	5,49E+09	7,81E+09	6,31E+09
G1TVH0	3,03E+08	0	0	0	0	0
G1TVH4	0	0	0	2,34E+08	4,12E+08	0
G1TVH9	3,5E+09	1,54E+09	1,92E+09	4,33E+08	2,73E+08	3,21E+08
G1TVK4;G1TW45;G1U1D0	3,79E+09	1,42E+09	1,12E+09	1,98E+09	1,65E+09	1,95E+09
G1TVN1;G1SI90	1,63E+09	2,87E+08	2,5E+08	1,7E+08	1,33E+08	1,15E+08
G1TVQ3;G1SVF4	7,55E+08	3,79E+09	4,18E+09	7,9E+08	1,16E+09	1,11E+09
P20058;G1TVS4	0	0	0	2,72E+08	2,91E+08	3,92E+08
G1TVS8	3,47E+09	1,04E+10	9,73E+09	4,61E+09	3,76E+09	2,84E+09
G1TVT0	7,08E+10	7,58E+10	8,77E+10	5,85E+10	6,25E+10	8,01E+10
G1TVT1;G1T7F5	4,97E+08	1E+09	1,26E+09	2,11E+09	1,6E+09	1,9E+09
G1TVU3	0	0	0	8,69E+08	9,46E+08	1,35E+09
G1TVU4	1,69E+10	8,97E+09	9,02E+09	1,35E+10	1,01E+10	1,98E+10
G1TVW1	1,09E+10	3,07E+09	2,96E+09	6,59E+09	4,77E+09	5,3E+09
U3KNK4;G1TVW5	0	1,36E+08	0	0	0	0
G1TVX2	88449000	99023000	0	1,61E+08	87879000	0
G1TVY5;G1T3X9;G1T5G1	7,48E+09	5,13E+09	5,06E+09	6,28E+09	6,98E+09	9,9E+09
G1TVY8	0	5,9E+08	4,07E+08	0	0	0
G1TW04	5,98E+08	0	0	0	0	0
G1TW17	9,96E+08	1,67E+08	0	1,78E+08	1,76E+08	0
G1TWC3	3,25E+09	6,34E+08	6,56E+08	2,87E+09	2,72E+09	3,44E+09
G1TWC4	1,02E+08	0	0	0	0	0
G1TWC5;G1T0L1	4,92E+08	1,17E+09	1,36E+09	1,24E+09	1,33E+09	1,8E+09
G1TWH1;G1U730	1,98E+08	0	0	0	0	0
G1TWI8	0	0	0	2,49E+08	2,12E+08	5,28E+08
G1TWK1	8,19E+09	2,61E+09	2,8E+09	7,59E+09	5,97E+09	3,83E+09
G1TWK7	6,21E+09	2,07E+09	2,46E+09	3,25E+09	3,24E+09	5,07E+09
G1TWL0	2,56E+10	1,12E+10	9,55E+09	2,78E+10	2,92E+10	3,78E+10
G1TWL2	2,91E+08	1,66E+08	0	3,52E+08	4,76E+08	6,01E+08
G1TWP1	0	0	0	2,9E+08	4,55E+08	0
G1TWP4	1,1E+09	1,47E+09	1,93E+09	3,48E+08	4,28E+08	5,56E+08
G1TWQ3	3,75E+08	1,05E+09	5,79E+08	4,45E+08	3,08E+08	4,23E+08
G1TWS0	6,38E+08	84891000	97193000	0	1,25E+08	80957000
G1TWS6	7,44E+08	0	1,36E+08	2,96E+08	1,52E+08	96167000
G1TWU1	3,27E+09	3,32E+08	3,94E+08	1,2E+09	1,04E+09	1,25E+09
G1TWU8	1,23E+09	5,71E+08	5,95E+08	1,3E+09	7,28E+08	9,49E+08
G1TWX9	4,66E+08	7,7E+08	8,2E+08	0	0	0
G1TWY9	2,75E+09	5,46E+08	6,19E+08	5,67E+09	5,62E+09	6,41E+09
G1TX03	1,29E+09	1,21E+09	1,31E+09	6,74E+08	8,79E+08	1,06E+09
G1TX33;G1U4I5;G1TI26	5,26E+09	1,6E+10	1,58E+10	6,54E+09	6,3E+09	5,83E+09
G1TX53	7,89E+09	3,62E+09	2,7E+09	8,56E+09	1,03E+10	1,58E+10

G1TX70	8,56E+08	3,31E+09	2,86E+09	1,99E+09	1,15E+09	5,97E+08
G1TX74	2,58E+08	9,09E+08	7,94E+08	0	0	0
G1TX78	2,54E+09	5,62E+08	4,46E+08	8,02E+08	7,38E+08	6,89E+08
G1TX94	2,38E+10	1,2E+10	9,65E+09	2,74E+10	2,31E+10	2,16E+10
G1TXA2	0	2,88E+08	3,15E+08	0	0	0
G1TXA3;G1TS81;G1SGR2	7,62E+08	2,06E+08	2,25E+08	3,45E+08	2,81E+08	0
G1TXB6	1,26E+10	8,13E+10	7,96E+10	5,54E+09	6,18E+09	8,49E+09
G1TXF6;G1U5T4;G1TM43	1,3E+10	1,99E+10	2,21E+10	8,39E+09	7,71E+09	7,09E+09
G1TXF8	4,76E+08	2,4E+09	1,98E+09	5,78E+08	4,19E+08	4,95E+08
G1TXG9	4,89E+09	1,9E+09	1,81E+09	1,06E+10	1,26E+10	1,65E+10
G1TXK2	0	0	0	0	0	1,52E+09
G1TXK3	0	1E+09	9,7E+08	1,26E+08	1,93E+08	2,07E+08
G1TXN1	1,11E+09	1,33E+09	1,3E+09	2,57E+09	2,73E+09	3,03E+09
G1TXQ6	4,25E+09	6,92E+09	5,56E+09	3,3E+09	3,02E+09	2,52E+09
G1TXS5	1,19E+09	1,99E+08	2,91E+08	1,16E+09	6,43E+08	3,06E+08
G1TXV6	92911000	1,8E+08	1,63E+08	0	1,58E+08	1,4E+08
G1TXW1	0	1,98E+09	1,91E+09	0	0	0
G1TXW6	6,92E+09	1E+09	1,55E+09	0	0	0
G1U0Z8;G1TXY0	1,4E+09	0	0	2,79E+09	1,92E+09	2,86E+09
G1TY06;P46409	3,73E+08	1,78E+09	1,43E+09	1,45E+09	3,7E+09	2,66E+09
G1TYA7;P13490;U3KMM1	1,01E+10	2,85E+10	2,66E+10	2,39E+10	2,77E+10	1,92E+10
G1TYE2	3,01E+08	9,52E+08	5,06E+08	1,8E+08	0	0
G1TYH7	9,5E+09	2,83E+09	2,96E+09	1,04E+10	9,62E+09	9,33E+09
G1TYK8;G1SCY1	1,03E+09	5,34E+08	6,06E+08	5,44E+08	4,82E+08	4,05E+08
G1TYM6	1,68E+10	5,27E+09	5,93E+09	4,01E+09	5,03E+09	5,68E+09
G1TYN0	8,27E+09	3,25E+09	2,04E+09	2,49E+10	1,85E+10	2,48E+10
G1TYR3;G1SFQ6	5E+08	4,94E+08	3,94E+08	0	0	2,02E+08
G1TYU5	0	1,85E+08	0	0	0	0
G1TYV3	0	0	0	3,81E+08	2,89E+08	5,41E+08
G1TYV6	3,89E+09	9,26E+08	9,66E+08	5,34E+09	3,66E+09	2,17E+09
G1TYW1	1,37E+09	3,05E+09	3,44E+09	1,45E+09	2,05E+09	1,95E+09
O77506;G1TYY5;U3KNF1	1,93E+09	2,02E+10	2,14E+10	1,6E+09	3,02E+09	2,13E+09
G1TZ09	0	1,72E+08	1,03E+08	0	0	0
G1TZ19	2,29E+09	4,49E+08	5,12E+08	1,93E+09	1,92E+09	1,52E+09
G1TZ23	1,33E+08	0	0	1,46E+08	1,98E+08	1,34E+08
G1TZ26	5,1E+08	9,6E+08	1,36E+09	4,33E+08	5,58E+08	6,46E+08
G1TZ31	4E+09	2,58E+09	2,19E+09	5,13E+09	4,47E+09	8,08E+09
G1TZ40	6,22E+08	7,35E+08	6,08E+08	5,99E+09	6,26E+09	7,57E+09
G1TZ63	4,14E+09	1,05E+09	9,91E+08	1,68E+09	1,16E+09	7,8E+08
G1TZ76	8,81E+08	5,79E+09	5,94E+09	2,23E+09	1,82E+09	1,62E+09
G1TZB9	2,73E+08	0	0	1,31E+08	1,34E+08	1,49E+08
G1TZC9	9,26E+08	2,45E+08	3,27E+08	6,55E+08	5,08E+08	3,33E+08
G1TZE2	3,2E+09	9,94E+08	9,41E+08	3,06E+09	2,31E+09	1,91E+09
G1TZG4	0	0	0	4,32E+08	4,29E+08	4,22E+08
G1TZI2	1,65E+09	4,19E+08	2,89E+08	1,05E+09	1,03E+09	4,84E+08
G1TZJ6	2,42E+08	1,43E+08	1,36E+08	4,08E+08	9,39E+08	8,08E+08
G1TZK6	8,08E+08	2,96E+08	2,91E+08	2,71E+08	2,29E+08	3,58E+08
G1TZN2	2,75E+08	2,33E+08	3,58E+08	4,07E+08	8,16E+08	7,56E+08
G1TZN7	5,29E+10	1,85E+10	1,59E+10	6,88E+10	8,26E+10	1,01E+11
G1TZP0	1,4E+10	4,24E+10	4,69E+10	5,85E+09	1,02E+10	1,14E+10

G1TZQ5	1,84E+08	1,99E+08	2,18E+08	0	0	0
G1TZQ6	2,22E+10	5,77E+09	4,3E+09	1,74E+10	1,73E+10	1,55E+10
G1TZU5	2,7E+09	1,18E+09	1,26E+09	3,29E+09	3,91E+09	5,42E+09
G1TZV3;G1TSM0	2,91E+08	3,7E+08	1,6E+08	1,36E+08	1,67E+08	0
G1TZY1	0	0	0	0	5,96E+08	1,48E+08
G1TZZ3;G1TM28;G1SS07	7,09E+08	6,16E+09	6,67E+09	8,02E+08	7,7E+08	1,78E+09
G1U000	2,9E+08	48284000	0	38364000	1,63E+08	1,37E+08
G1U001;G1U4G2	5,19E+08	1,31E+10	1,45E+10	2,87E+09	3,87E+09	4,1E+09
G1U013;U3KMS5	2,68E+08	8,35E+08	9,19E+08	8E+08	7,91E+08	5,7E+08
G1U018	7,5E+09	2,4E+09	2,78E+09	7,63E+09	6,7E+09	7E+09
G1U058	2,06E+08	0	0	0	0	0
G1U070	6E+09	1,17E+09	1,27E+09	9,01E+09	6,19E+09	4,3E+09
G1U072;G1TTQ6;G1U6Q9	1,63E+09	2,25E+09	2,54E+09	4,51E+09	4,12E+09	5,5E+09
G1U074	0	0	0	38576000	0	0
G1U095	0	0	0	2,77E+08	0	0
G1U0A4;U3KML1;O46377	1,34E+09	5,06E+08	6,28E+08	1,41E+09	6,37E+08	7,5E+08
G1U0B1	0	0	0	3,07E+08	0	0
G1U0B3	6,79E+09	2,2E+09	1,9E+09	5,53E+09	3,95E+09	2,79E+09
G1U0B4;P00008;G1TKY2	2,89E+10	1,43E+10	1,23E+10	4,54E+10	4,41E+10	6,74E+10
G1U0B5;G1TZ50	2,09E+08	1,98E+09	7,69E+08	2,47E+08	96742000	1,2E+08
G1U0I7	6,57E+08	1,63E+09	1,78E+09	3,58E+08	3,19E+08	3,92E+08
G1U0J6	99157000	0	0	0	0	84090000
G1U0N2	4,66E+08	3,7E+08	2,83E+08	1,18E+09	9,27E+08	1,15E+09
G1U0Q0	0	1,41E+08	1,02E+08	4,32E+08	3,1E+08	6,54E+08
G1U0Q2	2,5E+09	9,7E+09	7,14E+09	2,37E+09	3,22E+09	4,3E+09
G1U0Q7	2,97E+09	1,12E+10	1,17E+10	1,98E+09	2,29E+09	2,36E+09
G1U0R8	0	2,78E+08	0	0	0	0
G1U0S4	7,71E+08	1,33E+08	0	1,32E+08	0	1,51E+08
G1U0T4	0	0	0	0	0	1,68E+08
G1U0U5	1,02E+08	1,5E+08	1,63E+08	0	0	0
G1U0V9	1,79E+08	2,22E+08	0	2,47E+08	3,06E+08	5,14E+08
G1U0W2	3,75E+08	6E+08	4,94E+08	4,03E+08	5,11E+08	4,64E+08
G1U0Y0	0	0	87250000	0	0	0
G1U0Y6	5,56E+09	1,22E+09	1E+09	4,31E+09	3,72E+09	3,71E+09
G1U0Z7	8,11E+08	4,03E+08	5,34E+08	1,6E+09	1,18E+09	1,29E+09
G1U104	7,84E+08	3,53E+08	0	6,09E+08	4,32E+08	3,66E+08
G1U105	3,15E+08	0	0	2,42E+08	0	0
G1U115	3,44E+09	3,47E+09	3,81E+09	1,34E+09	1,15E+09	6,35E+08
G1U120	73911000	0	0	0	0	0
G1U150	6,14E+09	9,35E+08	8,38E+08	3,1E+09	3,32E+09	3,31E+09
G1U153	2,72E+09	1,88E+09	2,56E+09	3,97E+09	3,82E+09	3,81E+09
G1U194	4,51E+08	6,87E+08	8,14E+08	1,5E+09	1,34E+09	1,63E+09
G1U198	0	1,4E+09	1,46E+09	1,57E+08	2,16E+08	2,57E+08
G1U1C0	2,38E+09	7,69E+09	9,03E+09	3,4E+09	4,94E+09	5,65E+09
G1U1E5	0	2,23E+08	1,84E+08	0	0	0
G1U7W7;Q9N121;G1U1E6	2,07E+09	6,09E+08	8,06E+08	1,67E+09	2,39E+09	3,38E+09
G1U1H2	1,96E+08	4,36E+08	5,77E+08	2,42E+08	3,3E+08	5,71E+08
G1U1H3	1,8E+08	0	0	2,58E+08	2,35E+08	2,14E+08
G1U1M3	1,48E+09	7,02E+09	6,63E+09	2,08E+09	2,63E+09	2,6E+09
G1U1P4;P10829	0	0	0	0	42945000	0

G1U1Q1	0	0	0	6,28E+08	3,29E+08	3,78E+08
G1U1Q8	2,07E+09	1,46E+10	1,27E+10	9,65E+09	1,52E+10	1,47E+10
G1U1W8	1,39E+09	2,15E+09	2,09E+09	3,08E+08	0	3,4E+08
G1U1X1	19935000	77695000	95291000	11342000	9974600	0
G1U1X6	8,59E+08	3,46E+08	4,6E+08	6,56E+08	9,43E+08	9,9E+08
G1U1X8;G1TFU8;G1SEE3	2,54E+09	8,84E+08	8,27E+08	1,05E+09	8,94E+08	9,7E+08
G1U211	0	0	0	4,61E+08	2,98E+08	2,58E+08
G1U266	3,39E+08	0	0	1,81E+08	0	0
G1U276;G1U1I2	1,83E+10	5,25E+09	5,34E+09	1,52E+10	1,52E+10	1,4E+10
G1U2B5	0	1,18E+09	1,13E+09	7,85E+08	1,06E+09	9,7E+08
G1U2E3;G1SRU4	6,81E+08	2,77E+09	3,19E+09	8,5E+08	1,08E+09	1,14E+09
G1U2E5	2,35E+08	5,41E+08	7,74E+08	3,14E+08	0	0
G1U2E6	3,22E+10	3,95E+09	4,14E+09	2,06E+10	1,56E+10	1,4E+10
G1U2E8;G1T0H4	7,41E+08	0	0	3,79E+08	3,54E+08	0
G1U2N6	2,15E+09	3,18E+08	3,36E+08	1,18E+09	1,3E+09	9,06E+08
G1U2N9;G1SGX6	87707000	0	0	1,68E+08	1,25E+08	1,03E+08
P14282;G1U2Q7	1,76E+09	1,68E+08	1,81E+08	2,82E+09	1,44E+09	2,27E+09
G1U2R0	0	0	0	0	1,01E+08	0
G1U2R1	5,15E+09	1,54E+09	1,48E+09	6,17E+09	4,99E+09	2,93E+09
G1U2R2	1,98E+09	4,97E+09	5,97E+09	2,71E+09	2,05E+09	1,51E+09
G1U2S7;G1TGJ7	1,82E+08	0	0	0	0	0
G1U7U5;G1U2T2	6,69E+08	2,3E+08	3,26E+08	6,07E+08	7,33E+08	6,68E+08
G1U2V6	3,09E+09	8,55E+09	1,08E+10	2,38E+09	1,92E+09	2,65E+09
G1U2W0	1,16E+09	3,81E+08	4,06E+08	1,64E+09	1,83E+09	1,75E+09
G1U2X9	2,58E+09	7,13E+09	8,16E+09	1,46E+09	1,87E+09	1,4E+09
G1U2Z9	0	0	0	0	0	1,41E+08
G1U304	1,51E+08	0	1,92E+08	2,61E+08	2,81E+08	2,32E+08
G1U305	3,15E+08	3,53E+08	3,33E+08	2,96E+09	2,73E+09	2,9E+09
G1U315	0	7,38E+08	5,51E+08	2,11E+08	3,7E+08	2,01E+08
G1U328	1,53E+08	3,77E+08	5,86E+08	1,87E+08	2,11E+08	0
G1U344;G1T040;G1TLI2	1,31E+09	9,92E+09	9,33E+09	1,86E+09	2,47E+09	2,11E+09
G1U354	1,25E+09	1,3E+09	1,45E+09	7,28E+08	5,11E+08	4,29E+08
Q28651-2;Q28651-4;Q28651-3	0	90100000	1,02E+08	0	0	0
G1U383	39746000	0	1,23E+08	54321000	0	0
G1U3B2	0	5,44E+08	9,86E+08	4,69E+08	2,68E+08	3,67E+08
G1U3B8	2,65E+09	7,37E+09	9,13E+09	1,83E+09	2,85E+09	3,06E+09
G1U3C5	3,89E+08	1,61E+08	2,82E+08	2,01E+09	1,37E+09	1,17E+09
G1U3G6	9,19E+09	2,52E+09	2,45E+09	3,19E+09	2,88E+09	2,3E+09
G1U3G8	6,47E+08	0	0	0	0	0
G1U3I5	1,66E+10	5,73E+09	5,89E+09	1E+10	1,11E+10	1,3E+10
G1U3M5	2,69E+09	9,27E+09	1,06E+10	5,65E+09	5,6E+09	5,49E+09
G1U3P6	1,16E+09	5,98E+08	4,68E+08	1,28E+09	1,16E+09	1,03E+09
G1U3Q0;G1T8W7	2,02E+09	6,15E+08	5,26E+08	2E+09	1,82E+09	9,97E+08
G1U3Q5	0	5,2E+08	7,74E+08	0	0	0
G1U3Q6	4,18E+08	0	0	7,38E+08	4,69E+08	3,15E+08
G1U3S3	1,65E+08	1,49E+08	0	0	0	0
G1U3S6	8,52E+08	2,79E+08	3,01E+08	1,73E+09	1,22E+09	7,39E+08
G1U3T2	0	5,19E+08	5,27E+08	0	0	0
G1U3V0;G1SR26	4,38E+09	1,68E+09	1,82E+09	9,29E+09	9,47E+09	1,26E+10
G1U3X5	3,65E+08	2,13E+09	5,86E+09	1,38E+08	0	1,33E+08

G1U410	1,07E+10	5,87E+10	6,24E+10	1,33E+10	1,43E+10	1,87E+10
G1U411;G1TIB3	1,2E+09	7,02E+09	7,74E+09	2,24E+09	3,04E+09	3,03E+09
G1U430	8,23E+08	5,57E+08	4,13E+08	9,51E+08	1,43E+09	1,92E+09
G1U442	2,31E+08	0	0	2,36E+08	2,37E+08	2,32E+08
G1U446	4,92E+08	1,67E+09	1,03E+09	2,1E+09	2,05E+09	2,73E+09
G1U448	2,05E+09	9,04E+09	1,01E+10	6,87E+09	1,1E+10	1,08E+10
G1U453;G1SVD7	4,42E+08	1,59E+08	1,93E+08	2,03E+09	2,77E+09	2,67E+09
G1U460	9,41E+09	3,8E+09	3,07E+09	1,02E+10	8,93E+09	9,8E+09
G1U463	8,96E+08	0	0	2,98E+08	3,62E+08	3,93E+08
G1U469;P35748	0	3,68E+08	5,5E+08	0	0	0
G1U482	0	0	0	0	0	5,6E+08
G1U487	3,9E+08	0	0	0	0	0
G1U4C2	3,53E+08	0	0	0	0	0
G1U4E6	6,26E+09	7,75E+09	8,95E+09	1,97E+09	2,16E+09	2,29E+09
G1U4G9;Q95MF9	2,98E+09	1,82E+10	2,12E+10	5,38E+08	7,61E+08	8,5E+08
G1U4H9	1,37E+08	2,38E+08	5,25E+08	0	0	0
G1U4I9;G1TID7	3,8E+08	2,64E+08	0	6,54E+08	7,39E+08	9,05E+08
G1U4K3	0	0	0	0	57857000	0
G1U4L1	0	0	4,44E+08	0	0	0
G1U4L6	2,22E+08	0	0	0	0	0
G1U4P7	9,29E+09	2,99E+09	3,04E+09	6,6E+09	1,04E+10	1,02E+10
G1U4R5	2,4E+10	6,54E+09	6,94E+09	3,01E+10	2,24E+10	1,6E+10
G1U4U7;G1SJR6	0	0	0	0	0	3,51E+08
G1U4V1	3,38E+08	1,98E+08	2,81E+08	3,94E+08	1,42E+08	2,04E+08
G1U8R4;G1U4Y5	1,5E+08	0	0	0	0	1,9E+08
G1U516	0	0	0	0	0	43924000
G1U522	1,05E+09	5,68E+09	5,56E+09	3,83E+09	4,29E+09	5,91E+09
G1U535	7,45E+08	0	0	1,37E+09	1,06E+09	1,09E+09
G1U537	0	0	0	2E+08	1,94E+08	1,72E+08
G1U540	1,02E+09	1,84E+08	2,37E+08	3,45E+08	6,63E+08	8,79E+08
U3KMU0;G1U541	0	0	6,73E+08	0	0	0
G1U571	0	2,11E+08	0	0	0	0
G1U577	0	24462000	0	0	0	0
G1U5B3	1,71E+10	1,39E+10	1,26E+10	9,73E+09	1,1E+10	1,32E+10
G1U5D4	3,74E+08	0	0	0	0	0
G1U5F4	0	0	0	0	0	9,54E+08
G1U5L3	6,06E+09	2,79E+09	2,09E+09	1,58E+10	1,44E+10	1,38E+10
G1U5M3	1,59E+08	0	0	0	0	0
G1U5M7	3,65E+09	1,73E+09	1,36E+09	1,65E+09	1,79E+09	1,79E+09
G1U5N7;G1TJ15;G1TK25	6,98E+09	9,69E+08	1,18E+09	2,63E+09	3,23E+09	3,13E+09
G1U5P9	6,74E+08	2,2E+08	1,85E+08	2,04E+08	2,5E+08	3,5E+08
G1U5Q7	1,09E+09	8,5E+08	1,02E+09	5,86E+08	5,58E+08	5,17E+08
G1U5U0	3,29E+09	5,66E+08	4,92E+08	6E+08	5,71E+08	6,2E+08
G1U5W5	0	4,15E+08	5,65E+08	0	0	0
G1U5X6	5,87E+09	2,11E+09	1,92E+09	5,83E+09	7,93E+09	9,69E+09
G1U5Z2	1,98E+08	0	84912000	4,04E+08	2,46E+08	3,95E+08
G1U5Z3	1,24E+08	0	0	0	0	0
G1U603;G1T966	1,6E+08	7,63E+08	8,62E+08	0	1,32E+08	1,07E+08
G1U636	3,06E+09	1,01E+10	1,14E+10	2,25E+09	1,53E+09	2,39E+09
G1U643	0	2,94E+08	2,76E+08	0	0	0

G1U644	71752000	0	0	0	0	0
G1U670	3,8E+08	1,01E+09	1,11E+09	5,01E+08	4,26E+08	6,02E+08
G1U684	7,9E+09	2,56E+09	2,71E+09	3,9E+10	4,93E+10	5,51E+10
G1U6A6	98729000	4,25E+08	4,88E+08	0	0	0
G1U6B2	1,09E+09	9,54E+09	9,89E+09	2,07E+09	2,7E+09	2,56E+09
G1U6B4	9,18E+09	2,64E+09	2,27E+09	9,08E+09	1,28E+10	9,15E+09
G1U6C7	0	0	0	0	0	56599000
G1U6F4;G1TSU5;G1U2F1	3,64E+08	7,83E+08	7,34E+08	9,53E+08	9,47E+08	1,06E+09
G1U6G2	1,35E+09	1,88E+09	1,96E+09	7,95E+08	7,35E+08	7,46E+08
G1U6H0;G1SRB3	5,8E+10	1,03E+10	9,8E+09	6,05E+10	7,25E+10	9,23E+10
G1U6H4	3,22E+08	4,85E+08	5,67E+08	1,17E+09	2,27E+09	1,71E+09
G1U6J6	0	0	0	1,67E+08	2,04E+08	2,39E+08
G1U6N2	8,25E+08	3,53E+09	4,6E+09	2,13E+09	2,07E+09	2,39E+09
G1U6N8	1,83E+10	4,37E+09	4,91E+09	1,85E+10	1,75E+10	1,87E+10
G1U6R0	9,77E+08	2,46E+08	2,56E+08	9,56E+08	8,54E+08	7,2E+08
G1U6T5	1,41E+09	1,13E+09	1,1E+09	4,1E+08	7,96E+08	4,55E+08
G1U6W9	0	0	0	2,94E+08	3,4E+08	0
G1U6X4	2,76E+08	3,56E+08	7,23E+08	2,02E+08	2,07E+08	2,59E+08
G1U6X6	3,29E+09	2,95E+09	3,17E+09	3,98E+09	5,28E+09	7,66E+09
G1U723;G1SPR2;P80508	4,56E+08	1,28E+09	1,06E+09	2,65E+09	4,51E+09	2,48E+09
G1U724	3,92E+08	2,26E+09	2,44E+09	4,41E+08	6,79E+08	6,16E+08
G1U797	7,5E+08	2,67E+09	2,27E+09	1,12E+09	1,3E+09	1,31E+09
G1U7A6	3,55E+08	0	0	0	0	0
G1U7C5;G1TQS9	9,65E+09	2,15E+10	2,05E+10	1,15E+11	1,20E+11	1,08E+11
G1U7C6	3,08E+09	4,43E+09	4,79E+09	3,42E+09	2,94E+09	3,38E+09
G1U7G6	3,86E+10	1,16E+10	1,2E+10	2,81E+10	2,79E+10	2,83E+10
G1U7I9;G1U968	5,55E+09	8,97E+09	9,31E+09	5,84E+09	8,09E+09	6,5E+09
G1U7J2	0	0	0	1,68E+08	2,51E+08	0
G1U7K4	1,04E+10	5,16E+10	5,42E+10	4,8E+09	8,84E+09	6,81E+09
G1U7K9;G1SL50	6,17E+09	3,28E+10	3,49E+10	6,41E+09	1,43E+10	1,41E+10
G1U7L1;G1TL08	5,42E+09	2,38E+10	2,37E+10	6,22E+09	6,21E+09	4,88E+09
G1U7L4	5,77E+11	2,24E+11	2,21E+11	4,15E+11	4,79E+11	6,15E+11
G1U7M4	9,78E+08	1,07E+10	9,05E+09	2,1E+09	2,69E+09	2,91E+09
G1U871;G1U7P6	0	3,35E+08	6E+08	1,74E+08	0	0
G1U7Q6	5,19E+08	1,16E+09	1,44E+09	6,45E+08	5,92E+08	6,49E+08
G1U7S8	1,51E+09	1,68E+09	1,89E+09	8,61E+08	8,88E+08	8,85E+08
G1U7U3	3,01E+08	1,38E+09	2,05E+09	7,24E+08	4,84E+08	7,86E+08
G1U7V8	6,73E+09	4,76E+10	4,99E+10	7,02E+09	9,09E+09	9,93E+09
G1U7X2	8,64E+08	2,9E+09	3,86E+09	3,63E+09	5,23E+09	5,84E+09
G1U7Y3	0	0	0	1,33E+08	1,59E+08	1,47E+08
G1U800	0	0	0	0	1,46E+08	3,63E+08
G1U826;G1U1Y9	2,26E+09	2,29E+09	1,96E+09	2,5E+09	4,06E+09	3,13E+09
G1U840	0	1,5E+08	1,13E+08	0	0	0
P00949;G1U866;P00949-2	2,34E+08	8,2E+08	8,53E+08	7,53E+08	4,95E+08	5,26E+08
G1U886	0	0	1,39E+08	0	0	0
G1U891	1,92E+09	2,84E+08	3,04E+08	5,44E+08	5,78E+08	1E+09
G1U895	0	0	0	0	0	2,2E+08
G1U8B3	1,74E+09	6,7E+09	7,65E+09	1,72E+09	2,3E+09	2,64E+09
G1U8C4	5,03E+08	2,39E+09	1,8E+09	5,16E+08	4,31E+08	4,78E+08
G1U8F0	2,76E+09	2,47E+09	2,77E+09	1,42E+09	1,03E+09	7,54E+08

U3KM44;G1U8G5	0	1,26E+08	0	0	0	0
G1U8G9	3,12E+08	4,21E+08	3,98E+08	7,08E+08	4,81E+08	9,03E+08
G1U8I5;P27123	3,83E+08	4,09E+09	4,41E+09	5,86E+08	1,12E+09	1,07E+09
G1U8J5	3,97E+10	4,11E+09	4,4E+09	1,56E+10	1,93E+10	2,6E+10
G1U8K3	1,86E+09	2,81E+08	3,46E+08	1,34E+08	0	0
G1U8M6	4,52E+08	5,92E+08	7,19E+08	2,15E+09	7,76E+08	4,89E+08
G1U8N0	1,17E+08	0	0	0	0	0
G1U8P2	7,91E+08	3,65E+09	4,33E+09	1,33E+09	1,96E+09	1,8E+09
G1U8Q6;G1SP12	0	0	0	1,12E+09	1,07E+09	1,08E+09
G1U8R2	1,89E+09	3,02E+09	3,14E+09	8,31E+08	1,83E+09	1,36E+09
P35324;G1U8R3;G1TRN5	1,61E+08	8,9E+08	6,67E+08	47477000	0	0
G1U8S6	0	0	0	2,44E+08	0	0
G1U8T9;G1T0R2;G1SMP4	3,67E+08	1,85E+09	1,65E+09	7,84E+08	2,99E+08	4,56E+08
G1U8V2	3,39E+09	8,27E+08	8,83E+08	7,88E+09	8,79E+09	1,09E+10
G1U8W6;G1SNS5;U3KN82	7,84E+09	2,65E+10	2,65E+10	4,16E+09	4,05E+09	4,48E+09
G1U8Y1	2,84E+09	1,18E+09	1,44E+09	1,29E+09	1,17E+09	7,83E+08
G1U913	3,61E+08	0	1,57E+08	1,04E+08	0	0
G1U945;U3KPP1;G1TKS8	2,38E+09	1,05E+10	8,15E+09	2,35E+09	2,55E+09	1,89E+09
G1U949	1,11E+09	9,23E+09	9,6E+09	2,09E+09	3,49E+09	2,63E+09
G1U971	1,78E+09	6,58E+09	7,73E+09	5,51E+08	6,69E+08	6,47E+08
G1U974	3,14E+09	1,54E+10	1,57E+10	8,44E+09	1,16E+10	1,08E+10
G1U998	1,99E+08	0	0	0	0	0
G1U9B4	3,94E+09	2,24E+09	2,04E+09	2,88E+09	3,51E+09	3,35E+09
G1U9C1;G1T953	84942000	3,11E+08	3,81E+08	0	74494000	0
G1U9D3	0	6,42E+08	6,99E+08	0	0	3,52E+08
G1U9G5	0	0	0	0	0	2,71E+09
G1U9J9	0	2,69E+08	1,51E+08	0	0	0
G1U9L6	1,69E+08	0	0	3,28E+08	4,62E+08	3,51E+08
G1U9Q1	9,54E+08	1,06E+09	9,13E+08	9,15E+08	1,24E+09	1,71E+09
G1U9R0	2,9E+10	2,07E+11	1,99E+11	3,3E+10	4,29E+10	4,11E+10
G1U9R6;Q28749	9,86E+09	3,48E+09	3,02E+09	3,68E+10	2,51E+10	2,04E+10
G1U9R7	5,2E+09	6,91E+08	4,32E+08	4,53E+09	3,21E+09	3,7E+09
G1U9R8	6,51E+09	1,83E+10	1,71E+10	9,1E+09	1,29E+10	1,09E+10
G1U9S2;P49065	2,57E+08	2,6E+08	3,05E+08	1,57E+09	3,26E+09	1,24E+09
G1U9S7	1,08E+10	3,79E+10	3,92E+10	6,48E+09	5,91E+09	5,64E+09
G1U9T1	7,51E+09	1,97E+10	2,18E+10	4,11E+09	3,68E+09	3,94E+09
G1U9T2;G1TRZ8;G1SR75	5,34E+08	9,5E+08	9,27E+08	3,27E+08	3,82E+08	4,18E+08
G1U9T4	1,17E+10	3,85E+10	3,62E+10	1,13E+10	1,4E+10	1,12E+10
G1U9T8;G1U9U0	6,21E+09	1,92E+10	2,18E+10	4,51E+09	4,79E+09	3,56E+09
P33477;G1U9U1	0	2,95E+08	2,98E+08	0	0	0
O02768;U3KM62	2,15E+10	2,93E+09	3,92E+09	2,4E+09	8,56E+09	8,93E+09
O18757	6,55E+09	8,68E+08	7,66E+08	4,46E+09	4,64E+09	5,46E+09
O19048	7,55E+09	3,91E+10	4,09E+10	8,08E+09	7,89E+09	9,64E+09
O19049	3,48E+10	5,1E+10	5,56E+10	4,94E+10	6,11E+10	7,57E+10
O19105;G1SWM3	7,73E+08	1,38E+08	0	1,14E+08	0	0
O46373	4,67E+10	1,61E+10	1,47E+10	4,48E+10	3,57E+10	2,45E+10
O46638;G1TLF0	1,13E+09	5,84E+09	5,91E+09	1,09E+09	2,69E+09	1,61E+09
O62742;O62742-2	2,69E+09	0	0	2,33E+09	2,4E+09	2E+09
O77622;G1T341	1,18E+10	3,03E+10	3,04E+10	4,72E+09	4,91E+09	4,56E+09
G1TVE5;G1TEL6;G1TP34	5,66E+08	3,22E+09	3,14E+09	4,31E+08	5,45E+08	2,48E+08

O77768;G1SW06;G1T4K6	1,81E+10	2,17E+09	1,76E+09	6,59E+09	1,16E+10	1,07E+10
O79431	8,13E+09	2,04E+09	2,24E+09	1,24E+10	1,45E+10	1,41E+10
O79436	4,76E+08	0	0	2,5E+08	2,25E+08	0
O79437	1,6E+08	65127000	0	0	0	77868000
P00169-2;P00169	2,99E+10	4,69E+09	5,13E+09	4,93E+10	5,85E+10	6,97E+10
P00389;G1TV40	1,19E+11	2,92E+10	2,41E+10	5,63E+10	4,58E+10	4,15E+10
P00637;G1TH72	0	3,1E+08	1,58E+08	8,53E+08	7,21E+08	4,82E+08
P00820;A0A0A0MQQ1	1,91E+08	2,34E+08	1,97E+08	7,55E+08	1,02E+09	1,38E+09
P00883	3,46E+10	1,23E+11	1,34E+11	2,21E+10	3,72E+10	2,79E+10
P00919	3,89E+09	1,11E+10	9,24E+09	1,4E+10	1,63E+10	1,11E+10
P00939	1,48E+10	7,51E+10	7,56E+10	3,83E+10	6,4E+10	5,29E+10
P01840	0	0	0	1,41E+09	0	4,41E+08
P01885	1,53E+09	4,44E+08	3,44E+08	1E+09	1,96E+09	1,33E+09
P06140;P01894	0	0	0	0	0	1,5E+08
P01948	1,44E+08	3,37E+08	1E+08	5,27E+09	1,07E+10	2,66E+09
P02057	0	0	0	4E+08	3,16E+08	0
P02251	3,43E+09	5,98E+09	3,93E+09	7,94E+08	9,4E+08	7,32E+08
P02252	1,88E+09	2,75E+09	3,01E+09	1,02E+10	6,62E+09	7,39E+09
P02493	1,59E+08	2,85E+08	2,51E+08	5,23E+09	2,17E+08	1,04E+08
P05176	3,8E+08	0	0	1,71E+08	2,53E+08	0
P06813	4,81E+09	1,01E+10	9,14E+09	3,9E+09	5,27E+09	6,15E+09
P06815	0	61095000	0	0	0	0
P07511	0	6,51E+08	7,11E+08	1,02E+08	0	1,17E+08
P07855	3,25E+08	5,05E+08	7,63E+08	0	0	0
P09451;G1THL2	5,66E+09	8,58E+08	1,29E+09	0	0	0
P10102;G1U7J6;G1THE4	9,61E+08	4,11E+08	5,22E+08	1,71E+09	1,55E+09	1,26E+09
P10160;G1TC37	1,11E+10	5,55E+10	6,66E+10	4,92E+09	5,75E+09	7,52E+09
P11084	0	0	0	0	50276000	0
P11611	0	4,43E+08	2,53E+08	0	2,27E+08	0
P11845;A0A075B6E1	1,62E+08	1,95E+09	1,45E+09	3,55E+08	7,48E+08	2,72E+08
P11909	0	0	4E+08	1,38E+09	1,71E+09	1,43E+09
P80291;P80289;P80292	0	2,64E+08	2,85E+08	0	36354000	0
G1SQD3;U3KNJ4;G1T8G9	4,78E+10	1,90E+11	1,95E+11	2,33E+10	3,24E+10	2,3E+10
P12345	8,1E+10	1,54E+10	1,48E+10	4,06E+10	3,52E+10	4,26E+10
P13280;G1TQZ7	2,93E+08	1,19E+09	1,36E+09	3,51E+08	5,35E+08	3,13E+08
P13491;G1U2T9	1,49E+10	3,5E+10	2,99E+10	2,1E+10	3,2E+10	2,2E+10
P14519	3,98E+10	6,67E+09	6,69E+09	6,62E+09	5,9E+09	8,11E+09
P15122	0	5,35E+08	4,5E+08	0	1,04E+08	1,29E+08
P15253	2,47E+11	9,28E+10	9,58E+10	1,83E+11	2,17E+11	3,08E+11
P15541;G1TE56	3,85E+08	1,44E+08	0	0	0	1,88E+08
P17177;G1SJK7	9,85E+08	2,32E+08	2,05E+08	6,5E+08	2,73E+08	3,71E+08
P80290;P18055	9,18E+08	4,92E+09	3,61E+09	3,52E+08	8,14E+08	3,67E+08
P19943	8,73E+09	1,32E+10	1,17E+10	4,61E+09	6,5E+09	5,93E+09
P20063;G1SDS2	3,02E+08	1,85E+08	1,68E+08	0	0	0
P20614;Z4YNC6	3,14E+08	1,37E+08	2,17E+08	1,24E+08	1,15E+08	1,05E+08
G1TH03;G1SI34;P04191	2E+10	5,19E+09	5,84E+09	1,34E+10	1,15E+10	7,98E+09
P21195	3,98E+11	1,74E+11	1,72E+11	3,92E+11	4,45E+11	6,10E+11
P23612;G1TA78	5,6E+08	6,02E+09	6,37E+09	3,26E+08	4,15E+08	5,83E+08
P24480;G1SNE8	1,43E+10	9,11E+10	9,23E+10	4,14E+09	6,12E+09	8,5E+09
P25704;G1U6X0	0	0	0	0	60760000	0

P27115	2,93E+08	1,64E+08	85037000	1,26E+08	1,07E+08	1,15E+08
P27124	2,79E+09	1,18E+10	1,28E+10	1,13E+09	2,44E+09	1,72E+09
P29694;G1TVL1	9,55E+09	4,12E+10	4,69E+10	5,88E+09	4,96E+09	3,26E+09
P29751	3,88E+11	6,35E+11	6,34E+11	2,11E+11	2,44E+11	2,90E+11
P30801	3,85E+09	4,03E+10	5,82E+10	1,24E+09	1,09E+09	2,38E+09
P30946;A0A0G2JH20	2,39E+10	1,05E+11	1,08E+11	1,47E+10	1,74E+10	1,78E+10
P30947;G1SUV5;G1SWK6	4,99E+10	1,71E+11	2,02E+11	1,72E+10	1,86E+10	1,85E+10
P32185	0	0	0	0	0	1,55E+08
P34032	1,59E+09	5,5E+09	6,03E+09	1,87E+09	2,12E+09	1,16E+09
P35566;G1SW80	5,96E+08	6,15E+08	6,29E+08	2,25E+08	3,16E+08	2,53E+08
P40826	1,68E+09	3,5E+09	3,35E+09	8,39E+08	8,15E+08	9,57E+08
P41035	1,3E+09	2,66E+09	2,66E+09	9,06E+08	1,19E+09	8,47E+08
P41111;G1TYD9	0	0	84296000	0	0	0
P41316;G1T4F9	3,3E+09	4,79E+09	2,75E+09	1,62E+10	1,37E+10	1,43E+10
P41975	8,37E+08	0	0	1,9E+09	1,25E+09	1,86E+09
P41982	1,34E+10	2,33E+09	2,29E+09	5,73E+09	7,31E+09	7,32E+09
P42675;G1SX63	1,24E+09	1,08E+09	9,17E+08	4,71E+08	5,81E+08	4,45E+08
P43236	3,69E+08	1,34E+08	0	0	0	0
P43348;G1TM81	6,28E+09	3,93E+10	4,27E+10	1,54E+09	2,56E+09	2,45E+09
P47845	1,63E+10	5,94E+10	6,11E+10	19422000	43750000	1,03E+08
P51662	1,94E+10	1,07E+11	1,36E+11	1,28E+10	1,8E+10	1,67E+10
P53787	5,69E+09	3,58E+10	3,53E+10	9,61E+09	1,08E+10	1,08E+10
P53815;G1TU86;G1SWH9	4,27E+09	3,79E+08	0	2,37E+09	2,44E+09	4,25E+09
P58772	2,36E+09	6,41E+09	6,01E+09	3,56E+09	3,1E+09	4,81E+09
P58776	0	2,42E+08	2,22E+08	0	0	0
P62139;P62139-2;G1U3X6	4,31E+09	1,11E+10	1,12E+10	3,95E+09	3,77E+09	3,53E+09
P62493	2,1E+10	6,92E+09	7,05E+09	1,75E+10	1,89E+10	1,68E+10
P62497;G1U034	3,87E+08	2,6E+09	2,88E+09	2,36E+08	2,8E+08	2,62E+08
G1SY51;G1T0U7;Q8HYX6	2,42E+09	1,04E+10	1,22E+10	1,64E+09	1,94E+09	2,9E+09
P63148;G1T7C8	0	0	0	0	0	1,56E+09
G1TUP4;G1U0U7;G1T1I6	1,06E+09	3,45E+09	3,52E+09	6,31E+08	1,05E+09	9,81E+08
P63169	3,07E+09	5,44E+09	4,82E+09	1,64E+09	1,95E+09	1,59E+09
P67777;G1TSB1	2,26E+09	6,3E+09	6,7E+09	1,11E+09	1,13E+09	1,01E+09
P67873	1,69E+09	2,44E+09	1,98E+09	1,38E+09	1,53E+09	1,64E+09
P68105;G1U6W1;G1TM98	6,03E+10	1,77E+11	2,12E+11	4,73E+10	5,49E+10	5,12E+10
P79398	9,94E+08	1,26E+09	1,5E+09	1,02E+09	1,03E+09	9,88E+08
P80584;G1SGU9;G1U0S3	8,76E+08	9,24E+09	9,45E+09	1,49E+09	2,18E+09	2,02E+09
P80912;G1SV28	1,75E+09	1,88E+10	1,86E+10	4,56E+09	6,38E+09	6,31E+09
P83468	7,05E+08	4,38E+09	4,5E+09	1,41E+09	2,43E+09	2,56E+09
P98049	1,62E+10	2,95E+09	2,1E+09	1,64E+10	1,38E+10	9,57E+09
Q01059	0	0	1,05E+08	0	0	0
Q01971;G1SIQ3;G1TP21	1,75E+10	6,52E+09	6,25E+09	2,39E+10	2,1E+10	1,35E+10
Q09YN4	1,2E+09	1,95E+09	2,19E+09	1,7E+09	1,93E+09	1,98E+09
Q09YN5	3,81E+08	0	0	94662000	0	0
Q09YN6;G1TZQ2	2,67E+08	2,37E+08	1,97E+08	1,35E+08	4,57E+08	3,29E+08
Q28618	3,49E+09	1,07E+10	1,07E+10	2,64E+09	3,38E+09	4,05E+09
Q28619;G1SFP2	1,19E+08	5,89E+08	4,11E+08	0	1,63E+08	0
Q28685	1,04E+08	0	0	2,79E+08	0	0
Q28709;G1TPC8	5,81E+09	4,26E+08	5,91E+08	3,47E+08	1,99E+08	3,39E+08
Q28717;G1TX59	1,78E+08	8,52E+08	9,91E+08	1,52E+08	1,04E+08	0

Q28740	1,2E+10	1,34E+09	1,64E+09	8,37E+09	8,88E+09	8,32E+09
Q29502;G1TUX8;G1TLL9	3,29E+08	3,66E+09	3,85E+09	4,73E+08	5,31E+08	5,3E+08
Q4PLJ0	6,9E+08	2,32E+09	2,14E+09	5,43E+08	1,01E+09	1,1E+09
Q5UE96	0	0	1,01E+09	2,27E+09	1,6E+09	1,34E+09
Q6SQH4	1,01E+10	3,69E+10	3,25E+10	1,75E+09	6,2E+09	4,01E+09
Q6TYA7	1,16E+09	2,93E+08	3,08E+08	1,63E+09	1,53E+09	1,08E+09
Q71V39	0	1,38E+08	0	0	0	0
Q7YQK3;A0A140TAV7	2,82E+10	3,57E+09	3,51E+09	1,02E+10	8,34E+09	1,08E+10
Q7YQK4	4,45E+08	0	0	0	0	0
Q8HZQ5	0	5,17E+08	0	0	0	0
Q8MI17;G1TNA5	2,77E+10	1,27E+11	1,18E+11	1,48E+11	1,73E+11	1,34E+11
Q8MJF1	5,65E+08	1,9E+09	1,13E+09	3,48E+09	3,56E+09	4,36E+09
Q8MK67	6,12E+09	3,25E+10	3,26E+10	9,82E+09	1,37E+10	1,89E+10
Q8MK68;G1TCG7	1,67E+08	0	0	1,32E+08	1,22E+08	1,16E+08
Q8WMG3	3,29E+08	0	0	3,57E+09	2,42E+09	1,78E+09
Q8WN94	7,17E+09	4,04E+10	4,19E+10	2,16E+10	3,01E+10	3,11E+10
Q95212	0	1,28E+09	1,5E+09	0	0	0
Q95KZ3	2,34E+09	5,92E+08	6,5E+08	1,89E+09	1,39E+09	1,01E+09
Q9BDY9	4,11E+08	4,88E+08	5,46E+08	1,53E+09	1,49E+09	2,13E+09
Q9GJP9	0	0	0	6,47E+08	0	0
Q9GK63;G1TFT0	0	0	0	1,07E+09	4,24E+08	4,07E+08
G1TRJ1;G1TXS8;G1TX79	3,39E+09	9,11E+08	9,14E+08	1,15E+09	1,02E+09	1,09E+09
Q9GLC3;G1SD34;G1TTG6	1,04E+10	2,48E+09	2,68E+09	2,81E+10	2,87E+10	3,12E+10
Q9N0J6;G1SZL4	0	0	0	0	0	63695000
G1SSX6;P27112;G1U418	8,16E+10	2,26E+10	2,15E+10	2,48E+11	2,21E+11	1,83E+11
Q9TT13	2,52E+09	8,36E+08	3,97E+08	8,81E+09	7,43E+09	5,77E+09
Q9TT15	2,52E+10	9,3E+09	7,12E+09	4,66E+10	5,23E+10	4,01E+10
Q9TT37;G1TUE1	1,45E+10	1,62E+09	1,16E+09	5,15E+10	4,78E+10	5,67E+10
G1TC44;G1TX83;G1SU28	6,84E+10	4,38E+11	4,84E+11	8,04E+10	1,24E+11	1,56E+11
Q9TTT8	0	4,15E+08	3,65E+08	2,68E+09	1,93E+09	2,74E+09
Q9TU29	1,69E+08	0	0	0	0	0
Q9XS70	9,32E+08	2,37E+09	2,5E+09	4,3E+08	4,72E+08	4,36E+08
U3KLV2	3,76E+08	2,39E+08	6,92E+08	0	0	0
U3KLX7	4,18E+08	1,13E+09	9,61E+08	0	2,55E+08	2,59E+08
U3KLY7	1,62E+09	6,65E+09	7,07E+09	3,53E+09	4,35E+09	3,65E+09
U3KLZ3	3,84E+10	1,22E+10	1,15E+10	3,28E+10	2,82E+10	3,8E+10
U3KM30	3,83E+09	5,58E+08	4,85E+08	8,89E+09	5,72E+09	6,97E+09
U3KM31;G1SKV7	1,63E+09	6,66E+09	7,89E+09	1,99E+09	2,78E+09	2,71E+09
U3KM64;G1SCE4	5,31E+09	8,35E+09	9,29E+09	2,3E+09	3,98E+09	3,29E+09
U3KM66	0	0	0	0	0	45903000
U3KM71	1,17E+10	1,92E+09	1,69E+09	1,56E+10	1,35E+10	1,46E+10
U3KM78;G1SRB1	8,24E+09	2,64E+09	2,44E+09	8,18E+09	6,49E+09	5,9E+09
U3KM82	0	0	0	1,37E+08	0	0
U3KM83	2,8E+08	1,38E+08	0	0	3,29E+08	2,47E+08
U3KM89;G1SHH1	8,6E+08	2,93E+08	4,21E+08	6,66E+08	7,17E+08	5,04E+08
U3KM96;G1TJ67	5,1E+09	2,2E+09	1,97E+09	5,01E+09	3,58E+09	2,9E+09
U3KMD1	4,06E+08	1,57E+09	1,8E+09	4,31E+08	5,57E+08	3,63E+08
U3KMD4	9,45E+08	0	0	2,74E+08	3,7E+08	0
U3KMI4	0	0	0	0	0	3E+08
U3KMP1	3,87E+09	1,28E+09	1,29E+09	4,25E+09	2,84E+09	1,79E+09

U3KMP2;G1SSS2;P55787	0	1,8E+08	0	3,23E+09	1,59E+09	6,28E+09
U3KMQ6	0	0	0	4,78E+08	0	0
U3KMQ7	7,42E+08	2,22E+08	2,35E+08	1,03E+09	4,12E+08	3,51E+08
U3KMU6	1,09E+10	2,16E+09	2E+09	4,12E+09	5,5E+09	6,43E+09
U3KMU7	4,67E+08	1,44E+09	1,5E+09	1,63E+08	2,88E+08	5,49E+08
U3KMU9	0	0	1,89E+08	0	0	0
U3KMY5	1,95E+09	2,97E+08	1,92E+08	5,8E+09	4,86E+09	5,33E+09
U3KMZ9	0	2,38E+08	2,87E+08	1,51E+08	1,8E+08	1,2E+08
U3KN22	3,85E+09	1,16E+09	7,99E+08	5,03E+09	6,37E+09	1,09E+10
U3KN24	4,48E+08	4,37E+08	2,13E+08	1,58E+08	0	0
U3KN73;G1T286	2,47E+08	1,41E+09	9,9E+08	2,06E+08	3,29E+08	3,49E+08
U3KN87	1,11E+08	6,35E+08	6,77E+08	0	1,03E+08	1,64E+08
U3KNB3	3,35E+08	6,74E+08	1,1E+09	2,54E+09	3,64E+09	3,44E+09
U3KNB6	2,13E+09	6,72E+09	6,01E+09	2,12E+09	2,37E+09	1,97E+09
U3KNB9;G1SE16	2,75E+08	0	2,76E+08	5,33E+08	3,64E+08	4,78E+08
U3KNE2	4,16E+09	4,69E+08	2,87E+08	3,76E+09	5,17E+09	3,92E+09
U3KNI3	0	0	0	0	0	16330000
U3KNK3;G1SW41	2,69E+08	1,47E+09	1,21E+09	1,26E+08	2,04E+08	0
U3KNL7	3,12E+08	1,39E+09	1,13E+09	0	0	0
U3KNP7;G1SL40	0	9,01E+08	8,18E+08	1,37E+09	1,74E+09	1,6E+09
U3KNY1	4,96E+09	6,67E+08	8,03E+08	3,07E+10	2,34E+10	2,09E+10
U3KP42	1,02E+09	0	0	9,87E+08	1,15E+09	1,15E+09
U3KP45	0	0	8,45E+08	0	0	3,87E+08
U3KPA0	2,82E+08	1,32E+09	1,21E+09	2,83E+08	3,51E+08	5,21E+08
U3KPB0;G1SNN6	0	5,41E+08	1,42E+09	2,75E+08	0	2,59E+08
U3KPB2;G1SPE6	1,87E+09	3,93E+08	3,64E+08	2,56E+09	1,25E+09	1,3E+09
U3KPD5	0	2,44E+09	2,27E+09	1,29E+08	4,36E+08	2,05E+08
U3KPG6	5,39E+08	2,58E+08	0	2,55E+08	0	0
U3KPH9	0	0	0	0	1,53E+08	0
U3KPJ0	8E+08	2,09E+08	2,31E+08	1,82E+08	1,45E+08	2,52E+08
U3KPL2	3,06E+09	6,37E+08	6,45E+08	9,82E+08	8,3E+08	6,46E+08

Supplementary Table S2: List of proteins found to be dysregulated in the *ex-vivo* CE vs. rCEnC proteomic comparison, which have been previously reported having a role in CE.

Protein Symbol	Protein name	CE
**Up-regulated in rCEnC**		
AP2B1	AP-2 complex subunit beta	Neural crest marker gene expressed in neural crest–derived progenitor of CEnC[1]
PLA2G4A	Cytosolic phospholipase A2	Up-regulated in HCEnC after CMV infection[2]
**Down-regulated in rCEnC**		
ATP1B1	Sodium/potassium-transporting ATPase subunit beta-1	Novel locus associated with FECD[3]
COL4A3	Collagen alpha-3(IV) chain	Mutations in TCF8 cause Posterior Polymorphous CD and ectopic expression of COL4A3 by CEnC[4]. TCF8 binds the promoter of COL4A3, missense mutations in TCF8 cause Late-Onset FECD [5]
COL4A6	Collagen alpha-6(IV) chain	There is a TCF8 binding site on COL4A6 promoter region, missense mutations in TCF8 cause Late-Onset FECD[5] and Polymorphous CD[4]
ENG	Endoglin	Cultured bCEnC were confirmed to express TGF-β type 1 and type 2 receptors and endoglin[6]
F11R/JAM1/JAMA	Junctional adhesion molecule A	Putative CE marker[7], it is expressed in tight junctions of the human CE[8]
GPX1	Glutathione peroxidase 1	It is was increased in HCEnC of older donors by 3.14-fold over those of young donors[9]
HSPA2	Heat shock-related 70 kDa protein 2	Expressed at higher levels in pediatric HCEnC[10]
ITGB5	Integrin beta-5	ITGB5 was found to be expressed in cultured HCEnC[11] and significantly slightly upregulated (1.4-fold) in FECD compared to healthy control[12]
KTN1	Kinectin	Target of miR-182, which is downregulated in CE of old mice compared to young mice[13]
LOXL3	Lysyl oxidase homolog 3	Found only in CE (not in corneal epithelium and stroma[14])
LRP1/CD91	Prolow-density lipoprotein receptor-related protein 1	Marker expressed significantly more in transformed (elongated) CEnC than in normal (patent 2016[15])
MGARP/C4orf49	MGARP	Expression markers of HCEnC[16]
SPARC/Osteonectin	SPARC	Osteonectin/SPARC and fibrillin-1 were found both on its stromal and CE aspects of DM[17], upregulated in FECD specimens[18]
TF	Serotransferrin	Altered in the aqueous humor proteome of patients with FECD[19]
TMPO/Thymopoietin/ LAP2	Lamina-associated polypeptide 2, isoform alpha	Target of miR-183, which is downregulated in the CE of old mice compared to young mice[13]

References

1. Katikireddy, K. R., Schmedt, T., Price, M. O., Price, F. W. & Jurkunas, U. V. Existence of Neural Crest–Derived Progenitor Cells in Normal and Fuchs Endothelial Dystrophy Corneal Endothelium. *The American journal of pathology* **186**, 2736-2750 (2016).
2. Miyazaki, D. *et al.* Corneal endothelial cells activate innate and acquired arm of anti-viral responses after cytomegalovirus infection. *Experimental eye research* **161**, 143-152 (2017).
3. Afshari, N. A. *et al.* Genome-wide association study identifies three novel loci in Fuchs endothelial corneal dystrophy. *Nature communications* **8**, 14898 (2017).
4. Krafchak, C. M. *et al.* Mutations in TCF8 cause posterior polymorphous corneal dystrophy and ectopic expression of COL4A3 by corneal endothelial cells. *The American Journal of Human Genetics* **77**, 694-708 (2005).
5. Riazuddin, S. A. *et al.* Missense mutations in TCF8 cause late-onset Fuchs corneal dystrophy and interact with FCD4 on chromosome 9p. *The American Journal of Human Genetics* **86**, 45-53 (2010).
6. Motegi, Y., Usui, T., Ishida, K., Kato, S. & Yamashita, H. Regulation of bovine corneal endothelial cell cycle by transforming growth factor-β. *Acta Ophthalmologica Scandinavica* **81**, 517-525 (2003).
7. Foster, J. W. *et al.* Cornea organoids from human induced pluripotent stem cells. *Scientific reports* **7**, 41286 (2017).
8. Mandell, K. J., Berglin, L., Severson, E. A., Edelhauser, H. F. & Parkos, C. A. Expression of JAM-A in the human corneal endothelium and retinal pigment epithelium: localization and evidence for role in barrier function. *Investigative ophthalmology & visual science* **48**, 3928-3936 (2007).
9. Joyce, N. C., Harris, D. L. & Zhu, C. C. Age-related gene response of human corneal endothelium to oxidative stress and DNA damage. *Investigative ophthalmology & visual science* **52**, 1641-1649 (2011).
10. Frausto, R. F., Wang, C. & Aldave, A. J. Transcriptome analysis of the human corneal endothelium. *Investigative ophthalmology & visual science* **55**, 7821-7830 (2014).
11. Chng, Z. *et al.* High throughput gene expression analysis identifies reliable expression markers of human corneal endothelial cells. *PLoS One* **8**, e67546 (2013).
12. Goyer, B. *et al.* Extracellular matrix and integrin expression profiles in Fuchs endothelial corneal dystrophy cells and tissue model. *Tissue Engineering Part A* **24**, 607-615 (2018).
13. Zhao, X. *et al.* MicroRNA profile comparison of the corneal endothelia of young and old mice: implications for senescence of the corneal endothelium. *Molecular vision* **19**, 1815 (2013).
14. Meade, M. L., Shiyanov, P. & Schlager, J. J. Enhanced detection method for corneal protein identification using shotgun proteomics. *Proteome science* **7**, 23 (2009).
15. Koizumi, N., Okumura, N., Hirano, H., Kinoshita, S. & Ueno, M. (Google Patents, 2016).
16. Frausto, R. F. & Aldave, A. J. Comparing the transcriptome of ex vivo endothelium with cultured human corneal endothelial cells. *Investigative Ophthalmology & Visual Science* **55**, 3585-3585 (2014).
17. Schlötzer-Schrehardt, U., Bachmann, B. O., Laaser, K., Cursiefen, C. & Kruse, F. E. Characterization of the cleavage plane in Descemet's membrane endothelial keratoplasty. *Ophthalmology* **118**, 1950-1957 (2011).
18. Weller, J. M. *et al.* Extracellular matrix alterations in late-onset Fuchs' corneal dystrophy. *Investigative ophthalmology & visual science* **55**, 3700-3708 (2014).
19. Richardson, M. R. *et al.* Alterations in the aqueous humor proteome in patients with Fuchs endothelial corneal dystrophy. *Molecular vision* **16**, 2376 (2010).
